# Safety and efficacy of four drug regimens versus standard-of-care for the treatment of symptomatic outpatients with COVID-19: A randomised, open-label, multi-arm, phase 2 clinical trial

**DOI:** 10.1016/j.ebiom.2022.104322

**Published:** 2022-11-01

**Authors:** Nomathemba Chandiwana, Chelsea Kruger, Hilary Johnstone, Mohamed Farouk Chughlay, Chung Ju, Byungsu Kim, Yengiwe Dineka, Sarah Arbe-Barnes, Robert Miller, Andrew Owen, Andrew Hill, Daniel Windgassen, Nada Abla, Anne Claire Marrast, Stephan Duparc, Willem Daniel Francois Venter

**Affiliations:** a Ezintsha, Faculty of Health Sciences, University of the Witwatersrand, Johannesburg, South Africa; b HJ-Clinical Trial Consultancy, George, South Africa; c Medicines for Malaria Venture, Geneva, Switzerland; d Shin Poong Pharm. Co. Ltd., Seoul, Republic of Korea; e Graduate School of Clinical Pharmacy, CHA University, Pocheon-si, Gyeonggi-do, Republic of Korea; f Artemida Pharma, Stevenage, United Kingdom; g Department of Molecular and Clinical Pharmacology, Centre of Excellence in Long-acting Therapeutics (CELT), University of Liverpool, Liverpool, United Kingdom; h DATAMAP, Freiburg, Germany

**Keywords:** SARS-CoV-2, Pyronaridine-artesunate, Artesunate-amodiaquine, Favipiravir + nitazoxanide, Sofosbuvir-daclatasvir, Outpatient

## Abstract

**Background:**

This exploratory study investigated four repurposed anti-infective drug regimens in outpatients with COVID-19.

**Methods:**

This phase 2, single centre, randomised, open-label, clinical trial was conducted in South Africa between 3rd September 2020 and 23rd August 2021. Symptomatic outpatients aged 18–65 years, with RT-PCR confirmed SARS-CoV-2 infection were computer randomised (1:1:1:1:1) to standard-of-care (SOC) with paracetamol, or SOC plus artesunate-amodiaquine (ASAQ), pyronaridine-artesunate (PA), favipiravir plus nitazoxanide (FPV + NTZ), or sofosbuvir-daclatasvir (SOF-DCV). The primary endpoint was the incidence of viral clearance, i.e., the proportion of patients with a negative SARS-CoV-2 RT-PCR on day 7, compared to SOC using a log-binomial model in the modified intention-to-treat (mITT) population.

**Findings:**

The mITT population included 186 patients: mean age (SD) 34.9 (10.3) years, body weight 78.2 (17.1) kg. Day 7 SARS-CoV-2 clearance rates (n/N; risk ratio [95% CI]) were: SOC 34.2% (13/38), ASAQ 38.5% (15/39; 0.80 [0.44, 1.47]), PA 30.3% (10/33; 0.69 [0.37, 1.29]), FPV + NTZ 27.0% (10/37; 0.60 [0.31, 1.18]) and SOF-DCV 23.5% (8/34; 0.47 [0.22, 1.00]). Three lower respiratory tract infections occurred (PA 6.1% [2/33]; SOF-DCV 2.9% [1/34]); two required hospitalisation (PA, SOF-DCV). There were no deaths. Adverse events occurred in 55.3% (105/190) of patients, including one serious adverse event (pancytopenia; FPV + NTZ).

**Interpretation:**

There was no statistical difference in viral clearance for any regimen compared to SOC. All treatments were well tolerated.

**Funding:**

10.13039/501100004167Medicines for Malaria Venture, with funding from the UK Foreign, Commonwealth and Development Office, within the Covid-19 Therapeutics Accelerator in partnership with 10.13039/100004440Wellcome, the 10.13039/100000865Bill and Melinda Gates Foundation, and Mastercard.

**Research in context**

**Evidence before this study**

Before this study was conducted there was little published evidence for therapeutic interventions in outpatients with COVID-19. A PubMed search (3rd September 2020) was conducted using the following search terms: COVID-19 OR severe acute respiratory syndrome coronavirus 2 OR SARS-CoV-2 AND treatment AND outpatient filtered for ‘clinical trial’. Of the six results returned, only two reported therapeutic interventions: a study conducted in the US and Canada found that hydroxychloroquine did not substantially reduce symptom severity in outpatients with early, mild COVID-19; in contrast a study conducted in Iran found that both hydroxychloroquine and febuxostat improved symptoms of fever, cough, and tachypnoea. The weight of evidence has since shown hydroxychloroquine to be ineffective in COVID-19. The same search terms were used for ClinicalTrials.gov, with 88 studies posted before 3rd September 2020, though none had reported results at that time. Notably, since this study was conducted, several drugs have shown efficacy in high-risk non-hospitalised patients with COVID-19 (ritonavir-boosted nirmatrelvir, remdesivir, molnupiravir, sotrovimab, and bebtelovimab). However, there is limited evidence regarding the efficacy of these agents against more recent SARS-CoV-2 variants, and there are still no approved treatments for non-hospitalised COVID-19 patients who are not at high risk.

**Added value of this study**

This exploratory study of outpatient treatment of COVID-19 assessed the antiviral efficacy and safety of the artemisinin-based antimalarial drugs artesunate-amodiaquine and pyronaridine-artesunate, as well the antiviral favipiravir and antiparasitic nitazoxanide in combination. The study also contributes further evidence regarding sofosbuvir-daclatasvir in outpatients with COVID-19. All investigational treatments were evaluated in comparison to standard-of-care (SOC) with paracetamol. Note that the study included the SARS-CoV-2 Alpha (B.1.1.7), Beta (B.1.351), and Delta (B.1.617.2) variants, identified by genome sequencing, but did not evaluate antiviral efficacy against the Omicron (B.1.1.529) variant.

**Implications of all the available evidence**

The antiviral efficacy of the four repurposed anti-infective drugs assessed was not improved over SOC in outpatients with COVID-19, but due to a lack of power the study could not rule out important clinical differences in either direction (benefit or harm). All four drug regimens were well tolerated. Future studies should consider using larger sample sizes, different doses, different populations (e.g., high-risk patients), or alternative endpoints.

## Introduction

Caused by severe acute respiratory syndrome coronavirus 2 (SARS-CoV-2), the COVID-19 pandemic brought significant mortality, morbidity, and economic losses globally.^1^ There was an urgent need for globally accessible, well tolerated, and affordable outpatient treatments to diminish the risk of hospitalisation, reduce symptom duration and severity, prevent post-COVID syndrome,^2^ and inhibit transmission by limiting viral shedding.^3^, ^4^, ^5^ Several countries have since granted emergency approvals for various monoclonal antibodies and antiviral treatments for COVID-19, though access may be limited to those most at risk of poor outcome.^5^, ^6^, ^7^

Drug repurposing is an established route to accelerate the development of new treatments.^8^^,^^9^ By April 2020, a global *in vitro* screening effort had identified numerous candidates for repurposing against SARS-COV-2.^8^ In the absence of a validated pre-clinical SARS-CoV-2 model,^10^ several exploratory and large-scale clinical trials were planned, primarily in hospitalised patients, but also outpatients.

This exploratory study investigated the antiviral efficacy of four repurposed drug regimens versus standard-of-care (SOC) with paracetamol in outpatients with COVID-19. These were the antimalarial drugs artesunate-amodiaquine (ASAQ) and pyronaridine-artesunate (PA)^11^^,^^12^; the combination of the antiviral favipiravir (FPV)^13^ and antiparasitic nitazoxanide (NTZ)^14^; and the fixed-dose combination sofosbuvir-daclatasvir (SOF-DCV), approved for the treatment of hepatitis C.^15^

The choice of investigational regimens was finalised in May 2020. ASAQ, FPV, and NTZ were selected based on *in vitro* efficacy data and pharmacokinetic simulations to predict lung tissue drug concentrations.^8^ PA and ASAQ were selected based on *in vitro* efficacy,^8^ and physiologically based pharmacokinetic simulations indicating that lung concentrations for pyronaridine and desmethylamodiaquine (the major metabolite of amodiaquine) were predicted to exceed the 50% inhibitory concentration for SARS-CoV-2 (manuscript in preparation). No data were available on SOF-DCV activity against SARS-CoV-2, but the combination was included based on SOF activity against a range of other viruses.^16^, ^17^, ^18^, ^19^ When the protocol was finalised (May 2020), published clinical data for the investigational regimens were limited. Two clinical studies, both in hospitalised patients, were available for FPV in COVID-19, showing reduced time to viral clearance and improved chest CT scan findings,^20^ and a significantly reduced the time to relief from pyrexia and cough versus control, though without improvement in the day 7 clinical recovery rate.^21^

The investigational regimens were also considered for their known safety profiles in humans, immediate availability, and ease of delivery to low-resource settings. This study aimed to evaluate these promising COVID-19 outpatient treatments by demonstrating an increase in the proportion of patients with early viral clearance (day 7) versus SOC.

## Methods

### Study design

This exploratory phase 2, single centre, randomised, open-label, clinical trial was conducted in an outpatient setting in Johannesburg, South Africa between 3rd September 2020 and 23rd August 2021 (see Supplementary Appendix Protocol). This clinical trial is registered at ClinicalTrials.gov with the identifier NCT04532931.

### Ethics

All patients provided signed informed consent. The study was conducted according to Good Clinical Practice, the Belmont Report, the Declaration of Helsinki, and South African law. The protocol was approved by the South African Health Products Regulatory Agency and the Human Research Ethics Committee (Medical), University of the Witwatersrand (ref: 200602B). Gauteng Provincial Department of Health provided the approval for the study to recruit from public clinics (ref: GP202008_202). An independent data monitoring committee was convened to monitor safety and efficacy.

### Inclusion/exclusion criteria

Eligible patients were male or female outpatients, aged ≥18 to ≤65 years, body weight ≥45 kg, with a positive SARS-CoV-2 RT-PCR test and symptoms starting ≤96 h prior to randomisation (including fever or chills, cough, myalgia, sore throat, headache, conjunctivitis, shortness of breath, nausea, diarrhoea, new onset anosmia or ageusia), with oxygen saturation (SpO_2_) ≥95%, respiratory rate ≤24 breaths/minute, heart rate <120 beats/minute, and a normal mental state. Key exclusion criteria were pregnancy or lactation, QTc prolongation, or serum potassium <3.5 mmol/L. Patients >65 years of age were excluded to meet the eligibility criteria for mild COVID-19 disease in accordance with South African national guidelines.^22^ See Supplementary Appendix Protocol for full details.

### Randomisation and masking

Eligible patients were randomly allocated (1:1:1:1:1) using a centralised automated randomisation system to one of five arms: SOC (paracetamol), or SOC plus one of ASAQ, PA, FPV + NTZ, or SOF-DCV. Given the complexity of maintaining blinding over the different dosing regimens and durations for the five arms, and the urgent need to identify COVID-19 treatments, all drugs were administered open label.

### Interventions

Drug treatment was started on day 1: paracetamol 1000 mg 6-hourly as needed; ASAQ (Guilin Pharmaceuticals, China) 200/540 mg once daily for 3 days; PA (Shin Poong Pharm. Co. Ltd., Republic of Korea), once daily for 3 days dosed by body weight, 540/180 mg for 45 to <65 kg, and 720/240 for ≥65 kg; FPV (Strides Pharma Science Limited, India), 1600 mg 12-hourly for 1 day, then 600 mg 12-hourly for 6 days; NTZ (Ind-Swift Ltd., India) 1000 mg 12-hourly for 7 days with food; SOF-DCV (Mylan Laboratories, India), 400/60 mg once daily for 7 days. All treatments were given orally. The first dose was supervised, except for SOC which was taken as needed within the daily limit.

### Procedures

Enrolment visits required an in-person consultation and physical examination. To limit the risk of SARS-CoV-2 transmission, follow-up and interim study visits were conducted via telemedicine, telephone, or text/direct messaging. Patients self-quarantined and were transported to the study site for sample collection or visited at home in line with national infection control guidelines. All patients received counselling on infection control.

At screening (day 0), patient eligibility was assessed, demographic characteristics noted, and a blood sample taken for serum potassium. A mid-nasal swab and saliva sample were collected for RT-PCR detection and quantification of SARS-CoV-2, and viral culture. Post-screening patient assessments are shown in Supplementary Data Table S1.

To estimate adherence, drug containers were evaluated at day 28. Blood samples for pharmacokinetic assessments were collected on day 7 for all experimental treatment arms, plus day 3 for the ASAQ and PA arms. Drug plasma or blood levels were determined at a central site (FARMOVS, Bloemfontein, South Africa) using validated protocols (Supplementary Data Table S2).

Mid-nasal swab samples were collected on days 3, 7, 10, 14, and 28 for standardised qualitative and quantitative RT-PCR assays to confirm SARS-CoV-2 infection and investigate the change in viral RNA load (TaqMan and TaqPath assays [Applied Biosystems A47532 and A48067; ThermoFisher Scientific Waltham, MA, USA]). Serology was assessed at baseline (viral N protein SARS-CoV-2 IgG assay [Abbott Diagnostics, IL, USA; reference 06R90]), and repeated at day 28. Viral cultures (Vero E6 cell line) were performed for positive RT-PCR samples using published methods.^23^

Patients reported daily vital signs and SpO_2_ and completed the FLU-PRO® Plus questionnaire and FLU-PRO® Plus Global Additional Diary Items.^24^ Study site personnel assessed the WHO Ordinal Scale for Clinical Improvement.^25^^,^^26^ Adverse events were recorded throughout the study.

### Outcomes

The primary efficacy outcome was the incidence of SARS-CoV-2 clearance, defined as the proportion of patients with a mid-nasal swab negative for SARS-CoV-2 on qualitative RT-PCR on day 7. Secondary efficacy outcomes were: viral clearance at day 7 assessed by viral culture; viral clearance at days 3, 10, 14, 21, and 28 assessed by RT-PCR; time to SARS-CoV-2 clearance; estimated SARS-CoV-2 viral load; hospitalisation incidence to day 28, time to first hospitalisation, and the number of days hospitalised; disease severity as measured by the WHO Ordinal Scale for Clinical Improvement; FLU-PRO® Plus questionnaire responses; time to first zero WHO Ordinal Scale score; the proportion of days with fever, SpO_2_ values <93%, or respiratory symptoms (chest/respiratory FLU-PRO domain score >0); protocol-defined lower respiratory tract infection (LRTI) incidence (resting SpO_2_ <93% at two readings 2 h apart plus subjective dyspnoea and/or cough); mortality incidence.

Safety outcomes were the incidence and severity of adverse events, and changes in vital signs. Exploratory outcomes included SARS-CoV-2 seroconversion and the drug exposure–response relationship for the primary efficacy outcome. See Supplementary Appendix Statistical Analysis Plan.

### Statistics

Based on a viral clearance rate of 20% by day 7 in the control arm, a sample size of 50 patients per arm (250 total) provided ≥80% power to detect an increase in viral clearance to 50% for the investigational arms, assuming a two-sided 5% type 1 error rate and 20% loss to follow-up. The efficacy assumptions were based on published data of 65% viral clearance at day 7 with FPV versus 20% for lopinavir/ritonavir.^20^ Although there was more than one intervention, as this was an exploratory study designed to screen four treatments individually for efficacy in COVID-19 versus SOC, issues of multiplicity were not considered.^27^

The safety population included patients who received at least one dose of randomised treatment. The modified intention-to-treat (mITT) population included patients with a confirmed SARS-CoV-2 infection who received at least one treatment dose. The as-treated population was defined as the patients who completed treatment and had viral load results for day 7 and were analysed for a sensitivity analysis of the primary outcome by what treatment they received. The pharmacokinetic population included patients who received at least one treatment dose with at least one pharmacokinetic result (see Supplementary Appendix Statistical Analysis Plan for full definitions).

The primary efficacy endpoint was assessed in the mITT population using a log-link binomial model adjusted for treatment, age, sex, BMI, comorbidities, viral load category, and days of symptoms at enrolment. Estimates, two-sided 95% confidence intervals (CI) and corresponding *P* values were provided for adjusted risk ratios comparing each experimental arm against SOC. Patients withdrawing from the study before day 7 due to adverse events were considered PCR-positive at day 7. Sensitivity analyses were conducted assuming missing patients were failures and using the as-treated population. Subgroup analyses were planned for all covariates. Secondary efficacy endpoints were assessed in the mITT population, and safety endpoints in the safety population (Supplementary Appendix Statistical Analysis Plan). All statistical analyses were conducted using SAS® (version 9.4 in a Linux environment).

### Role of funders

The study was sponsored by Shin Poong Pharm. Co. Ltd. The study was funded by Medicines for Malaria Venture with grants obtained from UK Aid from the UK Foreign, Commonwealth and Development Office under the provision of the COVID-19 Therapeutics Accelerator in partnership with Wellcome, the Bill and Melinda Gates Foundation, and Mastercard. Additional funding was provided as follows: Shin Poong Pharm. Co. Ltd. funded the Data Safety Monitoring Board for this study; Medicines for Malaria Venture funded Naomi Richardson for writing, editorial, and graphic support services, and Julia Flynn for project management. Shin Poong Pharm. Co. Ltd. and Medicines for Malaria Venture were involved in development and approval of the protocol. Medicines for Malaria Venture provided trial, data management, and monitoring staff, conducted the statistical analysis, and developed the clinical trial report.

## Results

### Patients

**Fig. 1:**
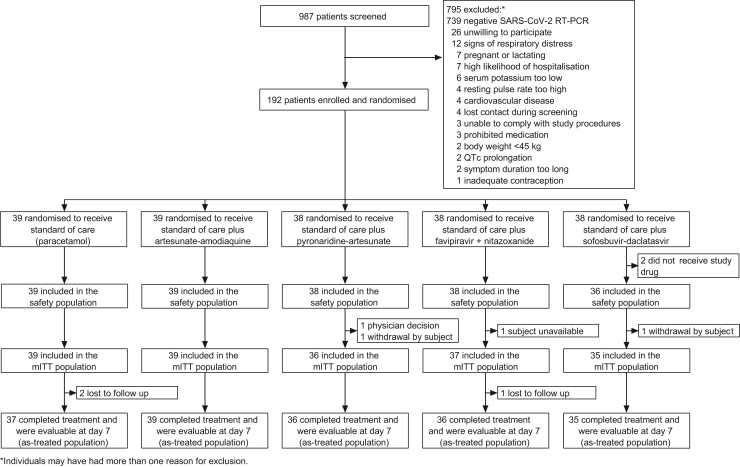
**Patient disposition**.

Of 987 patients screened, 192 were randomised, and 190 received at least one dose of allocated drug (Fig. 1). Four patients had a negative RT-PCR result at baseline but were enrolled before results were available. The mITT population included 186 patients, mean age 34.9 (SD 10.3) years, and mean body mass index 28.4 (6.6) kg/m^2^ (Table 1). One patient had received two doses of COVID-19 vaccine before enrolment. Demographic and clinical characteristics were similar across the study arms, though co-morbidities were less frequent in the SOC arm (Table 1).

**Table 1:** Patient baseline demographic and clinical characteristics (mITT population).

Characteristic	SOC (n = 39)	ASAQ (n = 39)	PA (n = 36)	FPV + NTZ (n = 37)	SOF-DCV (n = 35)
Sex, n (%)					
Female	24 (61.5)	25 (64.1)	20 (55.6)	15 (40.5)	15 (42.9)
Male	15 (38.5)	14 (35.9)	16 (44.4)	22 (59.5)	20 (57.1)
Age, years	33.7 (9.9) [19–60]	36.0 (10.3) [19–61]	35.9 (11.3) [18–61]	34.0 (9.4) [20–59]	34.8 (10.6) [22–62]
Weight, kg	73.9 (15.8) [50.4–117.8]	79.8 (18.3) [54.5–123.5]	79.3 (16.4) [51.4–128.9]	76.0 (16.2) [49.9–111.4]	82.2 (18.3) [50.0–132.2]
Body-mass index, kg/m^2^	27.3 (6.5) [17.7–44.9]	29.0 (6.8) [18.9–47.8]	29.4 (7.6) [18.2–49.1]	27.1 (5.7) [18.0–44.6]	29.1 (6.4) [17.5–43.7]
Race, n (%)					
Black African	36 (92.3)	32 (82.1)	34 (94.4)	32 (86.5)	31 (88.6)
Coloured	0	2 (5.1)	0	2 (5.4)	2 (5.7)
White	1 (2.6)	2 (5.1)	2 (5.6)	2 (5.4)	1 (2.9)
Asian or Indian	2 (5.1)	3 (7.7)	0	1 (2.7)	1 (2.9)
Temperature, °C	36.7 (0.6)	36.7 (0.5)	36.6 (0.5)	36.7 (0.4)	36.7 (0.6)
Pulse rate, beats/min	76.6 (12.6)	81.0 (11.7)	75.6 (12.8)	80.2 (14.2)	77.1 (14.1)
Respiratory rate, breaths/min	18.1 (2.9)	17.8 (2.6)	17.7 (3.1)	17.7 (2.6)	18.0 (2.4)
Oxygen saturation, %	97.2 (1.1)	97.0 (1.3)	97.4 (1.3)	97.0 (1.2)	97.1 (1.4)
Days of symptoms at time of enrolment	2.6 (0.6)	2.7 (0.6)	2.9 (0.5)	2.8 (0.6)	2.4 (0.7)
Nine-point WHO Ordinal Scale for Clinical Improvement score, n (%)					
1	37 (94.9)	39 (100.0)	36 (100.0)	36 (97.3)	35 (100.0)
Missing	2 (5.1)	0	0	1 (2.7)	0
Prior medical history, n (%)	12 (30.8)	8 (20.5)	11 (30.6)	9 (24.3)	7 (20.0)
Comorbidity, n (%)^a^	11 (28.2)	17 (43.6)	15 (41.7)	16 (43.2)	16 (45.7)
HIV infection	2 (5.1)	1 (2.6)	2 (5.6)	3 (8.1)	1 (2.9)
Obesity	10 (25.6)	14 (35.9)	14 (38.9)	13 (35.1)	15 (42.9)
Type 2 diabetes mellitus	2 (5.1)	1 (2.6)	2 (5.6)	1 (2.7)	0
Asthma	0	1 (2.6)	0	0	0
Hypertension	2 (5.1)	5 (12.8)	4 (11.1)	2 (5.4)	6 (17.1)
Log_10_ viral load^b^	5.17 (2.75)	4.94 (2.67)	4.68 (2.72)	5.23 (2.79)	4.55 (2.49)
Serology, n (%)					
Negative	28 (71.8)	28 (71.8)	21 (58.3)	29 (78.4)	22 (62.9)
Positive	11 (28.2)	10 (25.6)	12 (33.3)	8 (21.6)	12 (34.3)
Missing	0	1 (2.6)	3 (8.3)	0	1 (2.9)

SOC, standard-of-care; ASAQ, artesunate-amodiaquine; PA, pyronaridine-artesunate; FPV + NTZ, favipiravir plus nitazoxanide; SOF-DCV, sofosbuvir-daclatasvir; WHO, World Health Organization.

a Patients may have had more than one comorbidity.

b Based on quantitative RT-PCR viral load values (copies/mL), log_10_ transformed. Data are mean (SD) [range] unless otherwise stated.

### Efficacy

For the mITT population, the incidence of SARS-CoV-2 clearance based on qualitative RT-PCR at day 7 versus SOC was similar for ASAQ, PA, or FPV + NTZ with a risk ratio for all investigational arms <1.0 versus SOC (Table 2). For SOF-DCV, when adjusted for covariates, a statistically significantly lower proportion of patients had clearance at day 7 (23.5% [8/34]) compared with SOC (34.2% [13/38] *P* = 0.049) (Table 2). Sensitivity analyses of the primary efficacy outcome supported the primary analysis with none of the experimental arms significantly better than SOC (Supplementary Data Table S3).

**Table 2:** Incidence of SARS-CoV-2 clearance on day 7 based on qualitative RT-PCR (mITT population).

Outcome	SOC (n = 39)	ASAQ (n = 39)	PA (n = 36)	FPV + NTZ (n = 37)	SOF-DCV (n = 35)
Covariate adjusted analysis^a^					
Incidence, n/N (%)^b^	13/38 (34.2)	15/39 (38.5)	10/33 (30.3)	10/37 (27.0)	8/34 (23.5)
Risk ratio (95% CI)^c^	Reference	0.80 (0.44, 1.47)	0.69 (0.37, 1.29)	0.60 (0.31, 1.18)	0.47 (0.22, 0.996)
*P* value	Reference	0.48	0.25	0.14	0.049
Crude analysis					
Incidence, n/N (%)^b^	13/38 (34.2)	15/39 (38.5)	10/34 (29.4)	10/37 (27.0)	8/34 (23.5)
Risk ratio (95% CI)^c^	Reference	1.12 (0.62, 2.04)	0.89 (0.45, 1.75)	0.79 (0.40, 1.57)	0.69 (0.33, 1.46)
*P* value	Reference	0.70	0.73	0.50	0.33

mITT, modified intention-to-treat; SOC, standard-of-care; ASAQ, artesunate-amodiaquine; PA, pyronaridine-artesunate; FPV + NTZ, favipiravir plus nitazoxanide; SOF-DCV, sofosbuvir-daclatasvir.

a The covariate adjusted regression model contained treatment arm, age at baseline (years), sex, baseline BMI, baseline comorbidities, baseline viral load category and days of symptoms at time of enrolment as covariates. The crude analysis repeated the regression model without any covariate adjustment. One patient in the PA arm had missing co-variate values and was excluded from the adjusted analysis.

b n/N is number of patients with clearance/number of patients evaluable at day 7.

c A risk ratio >1 would represent an improvement relative to SOC.

Subgroup analysis of the primary outcome returned risk ratios <1 for all experimental treatment arms versus SOC, except for ASAQ in patients with ≥1 comorbidity (risk ratio 2.4; 95% CI 0.48, 12.0; *P* = 0.2879) (Supplementary Data Table S4). Viral culture results were only available for 16.1% (30/186) of patients, and no conclusions could be drawn (Supplementary Data Table S5).

By day 28, 69.7% (129/185) of evaluable patients had viral clearance assessed by RT-PCR. The proportion of patients with viral clearance at days 3, 10, 14, 21, and 28 was not improved versus SOC for any experimental treatment (Supplementary Data Table S6, Figure S1).

**Fig. 2:**
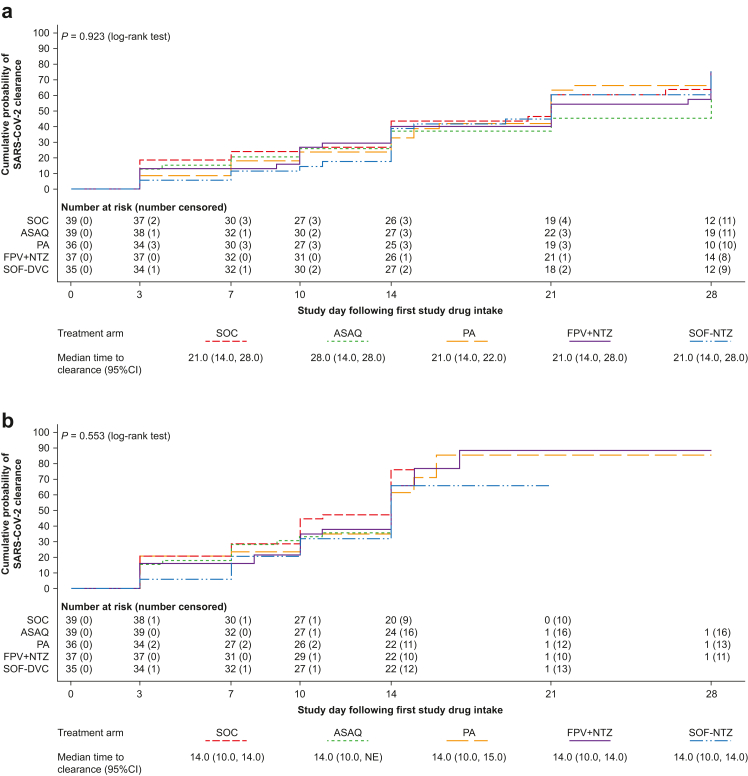
**Time to SARS-CoV-2 clearance (mITT population) based on (a) qualitative RT-PCR and (b) quantitative PCR.** Shown are Kaplan–Meier curves. Time to clearance was defined as the time to the first negative (a) qualitative or (b) quantitative SARS-CoV-2 RT-PCR test (collected post-baseline on days 3, 7, 10, 14, 21, and 28), without any subsequent positive SARS-CoV-2 RT-PCR test. Patients who withdrew from the study were censored on the day of withdrawal; patients with missing data were censored on the day of the last available data; patients without any post-baseline data were censored on day 1. mITT, modified intention-to-treat; ASAQ, artesunate-amodiaquine; PA, pyronaridine-artesunate; FPV + NTZ, favipiravir plus nitazoxanide; SOF-DCV, sofosbuvir-daclatasvir; NE, non-evaluable.

**Fig. 3:**
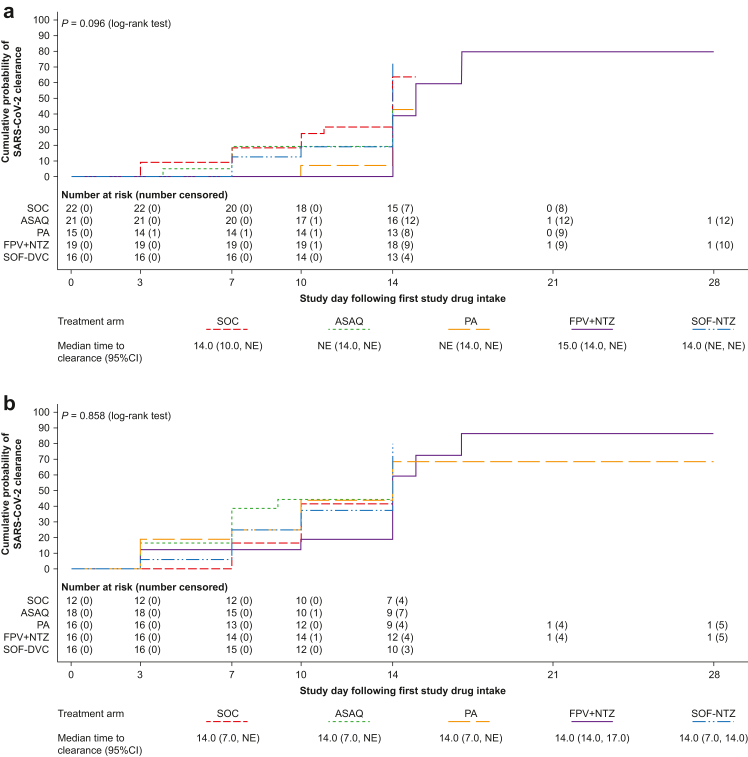
**Time to SARS-CoV-2 clearance based on quantitative RT-PCR in patients with (a) high viral load (mITT population) and (b) high-risk patients.** Shown are Kaplan–Meier curves. Time to clearance was defined as the time to the first negative quantitative SARS-CoV-2 RT-PCR test (collected post-baseline on days 3, 7, 10, 14, 21, and 28), without any subsequent positive quantitative SARS-CoV-2 RT-PCR test. Patients who withdrew from the study were censored on the day of withdrawal; patients with missing data were censored on the day of the last available data; patients without any post-baseline data were censored on day 1. mITT, modified intention-to-treat; NE, non-evaluable; ASAQ, artesunate-amodiaquine; PA, pyronaridine-artesunate; FPV + NTZ, favipiravir plus nitazoxanide; SOF-DCV, sofosbuvir-daclatasvir. High viral load was ≥175,145 copies/mL. High risk was defined as age >60 years or body mass index >30 kg/m^2^ plus the presence of at least one comorbidity for progression to severe disease.

Kaplan–Meier estimated median time to SARS-CoV-2 clearance based on qualitative RT-PCR was 21.0 days (95% CI 14.0, 28.0) for SOC, and was similar across the treatment arms (*P* = 0.923) (Fig. 2a). Using quantitative RT-PCR, the median time to clearance was 14.0 days (95% CI 10.0, 14.0) for SOC and similar for all treatment arms (*P* = 0.553) (Fig. 2b). Subgroup analysis based on quantitative RT-PCR showed no statistically significant differences between treatments in median time to clearance in those with a high baseline viral load (≥176,145 copies/mL; *P* = 0.096; log-rank test) (Fig. 3a) or high-risk patients (*P* = 0.858; log-rank test) (Fig. 3b).

**Fig. 4:**
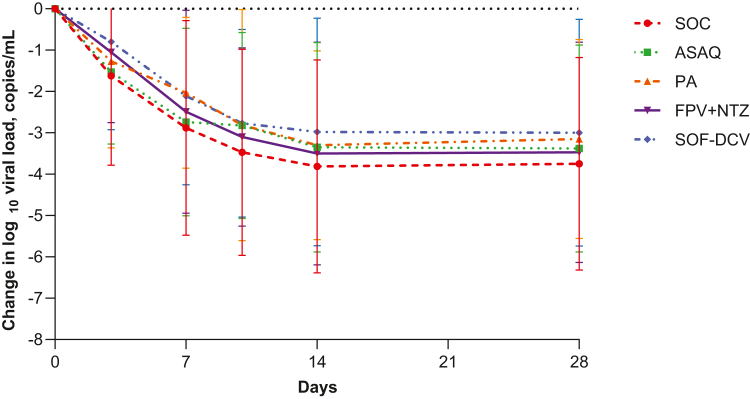
**Change from baseline in log**_**10**_**viral load estimated from quantitative RT-PCR (mITT population).** Shown are mean with SD (bars). Viral load values (log_10_ copies/mL) were calculated across the S, N and ORF-1ab genes. The number of evaluable patients at each timepoint is shown in Supplementary Data Table S7. mITT, modified intention-to-treat; ASAQ, artesunate-amodiaquine; PA, pyronaridine-artesunate; FPV + NTZ, favipiravir plus nitazoxanide; SOF-DCV, sofosbuvir-daclatasvir.

Baseline mean viral load estimated from quantitative RT-PCR was low (4.92; SD 2.67 log_10_ copies/mL) (Table 1) and declined throughout the study at a similar rate in all treatment arms overall (Fig. 4, Supplementary Data Table S7), and in high-risk patients (Supplementary Data Table S8).

There were three cases of protocol-defined LRTI (PA 6.1% [2/33]; SOF-DCV 2.9% [1/34]), two of which required hospitalisation. One 40-year-old female (PA) was hospitalised and received supplemental oxygen for LRTI and respiratory distress (days 6–11); and one 43-year-old female (SOF-DCV) with COVID-19 pneumonia and respiratory distress (days 5–10) was categorised as hospitalised but received supplemental oxygen at home as a hospital bed was not available. All three LRTI cases resolved.

**Fig. 5:**
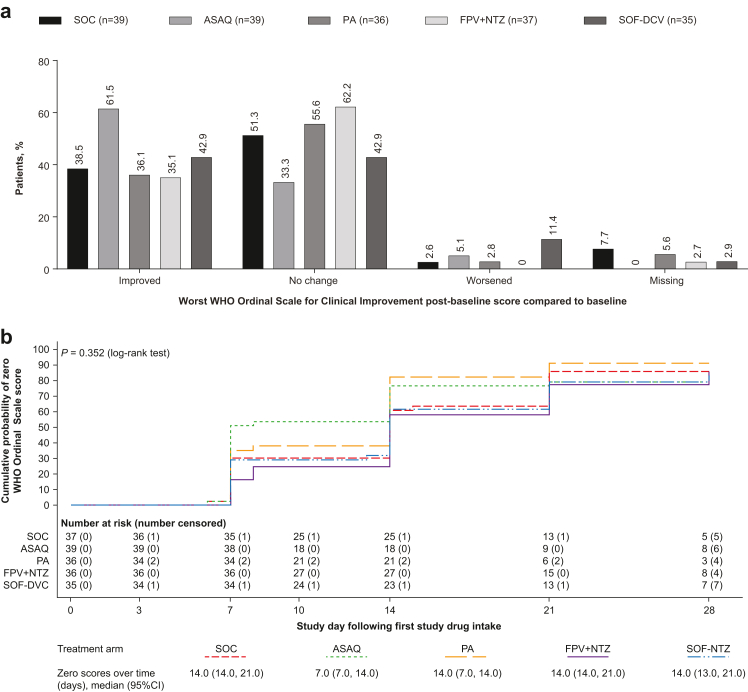
**(a) Change from baseline in WHO Ordinal Scale for Clinical Improvement (b) Time to first zero score on the WHO Ordinal Scale for Clinical Improvement (mITT population).** (a) Change from baseline was taken as the worst score at any time during the study. (b) Zero scores over time presents the time point (in days) by which the estimated cumulative probability of a zero WHO Ordinal Scale score was 50% (median) and the corresponding two-sided 95% confidence interval. Patients who withdrew from the study were censored on the day of withdrawal; patients with missing data were censored on the day of the last available data; patients without any post-baseline data were censored on day 1. mITT, modified intention-to-treat; ASAQ, artesunate-amodiaquine; PA, pyronaridine-artesunate; FPV + NTZ, favipiravir plus nitazoxanide; SOF-DCV, sofosbuvir-daclatasvir.

Based on the WHO Ordinal Scale for Clinical Improvement, there were no statistically significant differences between SOC and the investigational arms in disease severity at days 7, 14, 21, or 28 (Fig. 5a, Supplementary Data Table S9), or time to a score of 0 (Kaplan–Meier; Fig. 5b; Supplementary Data Table S10).

There were no statistically significant differences between treatment arms versus SOC in the number of days with post-baseline fever, SpO_2_ values <93%, or respiratory symptoms, except for the number of days with respiratory symptoms with ASAQ (rate estimate 25.9% [95% CI 22.1, 30.4]) versus SOC (33.0% [95% CI 28.3, 38.5]; rate ratio 0.79 [95% CI 0.64, 0.97]; *P* = 0.026) (Supplementary Data Table S11). Mean total scores for the FLU-PRO Plus questionnaire were low at baseline (0.92; SD 0.58) and improved during the study with no statistically significant differences between investigational arms and SOC (Supplementary Data Figure S2).

### Drug exposure

Mean adherence was estimated in the safety population as ASAQ 100% (n = 39), PA 100% (n = 38), FPV 97.3% (n = 38), NTZ 96.4% (n = 38), and SOF-DCV 94.8% (n = 36). Pharmacokinetic data were sparse, precluding the characterization of the full pharmacokinetic profile. Drug plasma or blood concentrations showed high inter-patient variability (Supplementary Data Table S12), with no apparent relationship between SARS-CoV-2 clearance at day 7 and drug concentrations on day 3 (PA and ASAQ) or day 7 (all investigational drugs) (Supplementary Data Figure S3).

### Safety

**Fig. 6:**
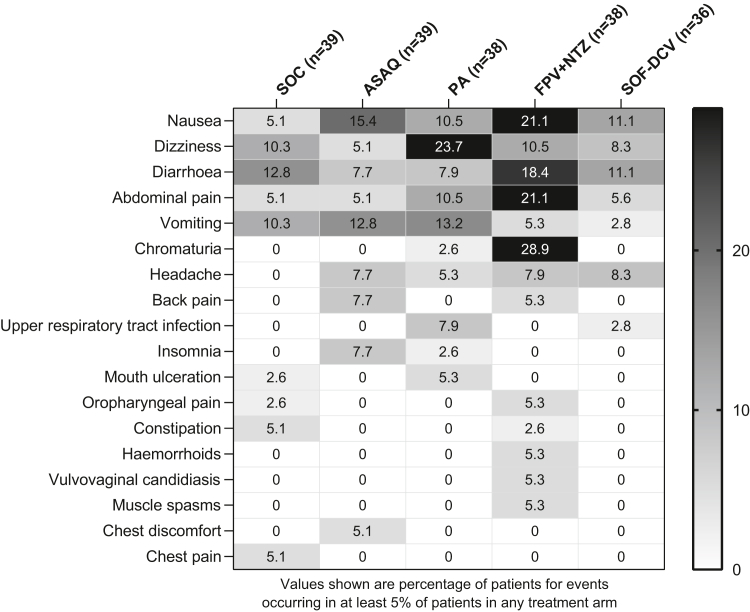
**Frequency of the most common adverse events of any cause (safety population).** Patients could have more than one adverse event. Adverse events were classified using MedDRA version 23.0. ASAQ, artesunate-amodiaquine; PA, pyronaridine-artesunate; FPV + NTZ, favipiravir plus nitazoxanide; SOF-DCV, sofosbuvir-daclatasvir.

There were 238 adverse events of any cause reported in 55.3% (105/190) of patients, with an incidence by treatment arm of SOC 35.9% (14/39), ASAQ 46.2% (18/39), PA 55.3% (21/38), SOF-DCV 58.3% (21/36), and FPV + NTZ 81.6% (31/38). Overall, the most common adverse events were nausea (12.6% [24/190]), dizziness (11.6% [22/190]), and diarrhoea (11.6% [22/190]). Gastrointestinal adverse events (nausea, diarrhoea, and abdominal pain) were particularly frequent with FPV + NTZ (Fig. 6); chromaturia was also reported in 28.9% (11/38) of patients in this arm. Dizziness was most common in the PA arm (23.7% [9/38]) (Fig. 6).

The majority (97.9% [233/238]) of adverse events were grade 1 or grade 2 in severity. There were four grade 3 adverse events: diarrhoea (SOC), suicidal ideation (SOC), and two cases of respiratory distress (PA; SOF-DCV) (Supplementary Data Table S13). None of the grade 3 adverse events were considered drug related. There was one grade 4 serious adverse event of pancytopenia requiring hospitalisation (FPV + NTZ), not considered drug related, but associated with a previously undiagnosed HIV infection. There were no other serious adverse events. Two adverse events led to drug discontinuation: abdominal pain (FPV + NTZ) and dizziness (SOF-DCV). There were no deaths.

Adverse events considered to be drug related were reported in 0% (0/39) of patients in the SOC arm, 27.8% (10/36) for SOF-DCV, 28.2% (11/39) for ASAQ, 31.6% (12/38) for PA, and 55.3% (21/38) for FPV + NTZ (Supplementary Data Table S13). The most common drug-related adverse events within each treatment group were ASAQ nausea 12.8% (5/39), PA dizziness 15.8% (6/38), FPV + NTZ chromaturia 28.9% (11/38), and SOF-DCV nausea 11.1% (4/36).

There were no clinically important changes in vital signs throughout the study, though there was a trend for a lower pulse rate with ASAQ versus other arms (Supplementary Data Figure S4).

### Serology

At baseline, 29.3% (53/181) of patients were positive for SARS CoV-2 serology, probably indicating prior exposure (Table 1). By day 28, 85.1% (148/174) of evaluable patients in the mITT population were positive for SARS CoV-2 serology (Supplementary Data Figure S5). The one patient who had received COVID-19 vaccine before enrolment was unevaluable for serology at baseline and negative at Day 28 (IgG index 1.3).

## Discussion

This study explored the potential of four repurposed drug regimens for the outpatient treatment of COVID-19. The study population was relatively young and at low risk of disease progression.

Notably, there were no cases of protocol defined LRTI in the SOC arm. There was no improvement in efficacy outcomes for any experimental arm versus SOC. Adverse events reported for the investigational regimens were broadly consistent with their known safety profiles,^11^^,^^12^^,^^28^, ^29^, ^30^, ^31^, ^32^ and all treatments were well tolerated.

Due to the epidemiological uncertainty of the timing and duration of waves, recruitment was slow outside the short-lived peaks of transmission, with around 93% of patients failing screening because of a negative RT-PCR test. Hence, recruitment was stopped at 192 patients, below the planned target of 250, but as the drop-out rate was lower (10%) than expected (20%), 38 patients per arm retained 80% power based on original assumptions. Nevertheless, the day 7 clearance rate in the SOC arm (34.2%) was higher than the initial hypothesis of 20%, probably because of prior exposure—29.3% of patients had positive baseline serology. Based on the higher-than-expected rate of viral clearance in the SOC arm, 152 patients per arm would be needed to detect a 50% efficacy rate in the experimental arms with 80% power. Thus, the study was under powered for the primary outcome. Note that sub-group analyses (high-risk patients, high viral load, etc.) were not powered to detect differences between the groups.

Notably, during the period of this study, few of the planned COVID-19 clinical studies in outpatients completed. Our five-arm study was logistically challenging given the extreme pressure on the South African health system. National restrictions to reduce SARS-CoV-2 transmission required a suite of innovative measures, including remote electronic data capture, the engagement, support, and training of patients in conducting and reporting assessments, and the use of mobile clinics. The high completion rate for patients recruited to the study indicates that study procedures were well accepted.

There are limited data on ASAQ or PA, or FPV and NTZ in combination for the treatment of COVID-19. ASAQ and PA were used at doses approved for the treatment of uncomplicated malaria. *In vivo* efficacy of pyronaridine in reducing viral titres and lung pathology was shown in SARS-CoV-2 infected transgenic mice expressing human host receptor hACE2 (K-18-hACE), possibly via inhibition of an essential viral protease, PL ^pro^.^33^ However, there are no published data for artesunate alone or ASAQ in SARS-CoV-2 rodent models, and their pharmacokinetics in COVID-19 patients are unexplored. Thus, there is scope to investigate different dosing regimens for these two drugs for the treatment of COVID-19.

For FPV, two studies in COVID-19 outpatients reported a median time to viral clearance of 10 days (n = 112) versus 8 days with placebo,^34^ and more rapid viral clearance with FPV (6.0 days; n = 83) than with control (14.0 days; n = 44; umifenovir plus intranasal interferon alpha-2b or hydroxychloroquine).^35^ Time to viral clearance was shorter in both studies than the 21.0 days (95% CI 14.0, 28.0) reported for SOC in the current study. FPV is a purine nucleoside analogue, inhibiting the viral RNA-dependent RNA polymerase (RdRp), slowing RNA synthesis.^13^ Thus, it may be more efficacious in early infection when viral replication rates are highest,^36^ and there is evidence for this in hospitalised patients.^37^ In our study, viral loads were generally low even in the SOC group, and this may explain the lack of a statistically significant treatment difference with the FPV + NTZ arm. Additionally, FPV pharmacokinetics display high variability in COVID-19 patients, and higher doses may be necessary.^38^ Similarly, for the NTZ component, we used a dose of 1000 mg BID with food, whereas recent pharmacokinetic modelling predicts an optimal dose of 1400 mg BID with food.^39^ The FPV + NTZ arm had the highest frequency of adverse events in this study, though in healthy volunteers NTZ doses of up to 1500 mg are well tolerated.^40^ However, the safety of high-dose FPV + NTZ would require careful evaluation, particularly in patients with renal or hepatic impairment.^39^

For SOF-DCV, a trial in outpatients reported no effect on hospitalisations versus hydroxychloroquine, but a reduction in fatigue and dyspnoea after 1 month.^41^ In the current study, a lower proportion of patients had viral clearance at day 7 with SOF-DCV than with SOC, and there was no evidence of a virological or clinical benefit. In hospitalised patients, SOV-DCV has not proven efficacious,^42^ though results in combination with ribavirin were more promising.^43^

The main limitations of this study are the use of virological outcomes and its size. Viral clearance was chosen as the primary outcome in May 2020, when there was no consensus regarding the most appropriate study design. This allowed a logistically feasible sample size for the assumed efficacy rates. Also, the use of five very different dosing regimens necessitated an open-label study design, and viral clearance was an objective measure to compare efficacy that would not be influenced by this design. However, there are limitations to using virological endpoints. As neither quantitative nor qualitative RT-PCR differentiate between infectious and replication incompetent virus, viral clearance and viral load assessed through RT-PCR may be inappropriate endpoints to evaluate efficacy.^44^^,^^45^ Although most COVID-19 trials have used a viral clearance endpoint, evaluated with a range of methods, it is still unclear which method is most clinically meaningful. Evaluation of clinical outcomes requires far higher patient numbers, and likely a patient population at greater risk of adverse outcome. For example, the evaluation of molnupiravir in non-hospitalised, high-risk, unvaccinated patients with mild-to-moderate disease required 1433 patients to detect a difference of −6.8% (95% CI, −11.3 to −2.4; *P* = 0.001) in death or hospitalisation.^46^ Similarly, the EPIC-HR study of nirmatrelvir plus ritonavir included 2246 patients at high risk for progression to severe COVID-19 to detect a relative risk reduction in hospitalisation or death of −5.62% (95% CI −7.21 to −4.03) at a rate of 0.8% (8/1039) with nirmatrelvir plus ritonavir and 6.3% (66/1046) for placebo.^47^ Such large studies with primary outcomes of hospitalisation/mortality and sustained symptom resolution are feasible for evaluating single interventions versus SOC but will be difficult to conduct in an era of widespread vaccination. A key strength of this study was its multi-arm design of candidate drugs that were promising at the beginning of the pandemic.

In conclusion, a higher proportion of patients receiving SOC achieved viral clearance at day 7 than expected, and none of the treatment regimens showed a virological efficacy benefit. There was generally no improvement for secondary virological endpoints and symptomatic endpoints. Study participants were young, and 29.3% were seropositive for SARS-CoV-2 at baseline, indicating prior exposure. The combination of this, a limited sample size, and virological endpoints now considered less relevant by current treatment study standards, means that due to a lack of power definitive conclusions related to efficacy cannot be made in either direction (benefit or harm). Since the predictive relationship between the magnitude and timing of viral RNA reduction and viral infectivity or clinical benefit has not been fully established, results should also be interpreted with caution. All treatments were, however, well tolerated. Future studies may consider using larger sample sizes, different drug doses, including patient populations at risk of developing severe disease, and evaluating alternative end points, to demonstrate efficacy.

## Contributors

NC, HJ, MFC, AO, AH, NA, SD, and WDFV contributed to the conception and design of the trial. NC, CK, HJ, MFC, YD, SA-B, RM, and ACM were involved in the acquisition of data. NC, CJ, BK, SA-B, RM, AO, AH, DW, NA, SD, and WDFV were involved in data analysis and interpretation. NC, RM, ACM, and DW verified the underlying data. All authors contributed to the development of the paper, provided critical review, and approved the final version for submission. All authors had access to the primary data and are able to take responsibility for the accuracy and completeness of the results. All authors had final responsibility for the decision to submit the paper.

## Data sharing statement

De-identified participant data are available on reasonable request and with completion of a signed data access agreement from (https://www.mmv.org/about-us/contact-us) referencing this publication. Data will be available for at least five years from publication of this study.

## Declaration of interests

NC declares grants and non-financial support from Shin Poong Pharm. Co. Ltd. during the conduct of the study and grants and non-financial support from ViiV Healthcare and Gilead Sciences, grants, personal fees and non-financial support from Johnson & Johnson, personal fees from Cipla and Frontiers Biotech outside the submitted work. NA, ACM, and SD are employees of Medicines for Malaria Venture. MFC is a former employee of Medicines for Malaria Venture. HJ is a consultant for Medicines for Malaria Venture and was a consultant medical monitor for this study. CJ and BK are employees of Shin Poong Pharm. Co. Ltd. CJ has a patent pharmaceutical composition for COVID-19 treatment pending. SA-B and RM are employees of Artemida Pharma which received funding from Shin Poong Pharm. Co. Ltd. RM consulted for Shin Poong Pharm. Co Ltd. and is the Shin Poong Pharm. Co. Ltd. qualified person for pharmacovigilance, responsible for providing interpretation of the safety data of this study and other studies involving pyronaridine-artesunate. SA-B consulted for Shin Poong Pharm. Co. Ltd. during the study for the operational aspects and in particular all activities related to the Data Monitoring Committee. AO is a director of Tandem Nano Ltd., co-inventor of patents relating to drug delivery, and has received grants and consultancy from AstraZeneca and Janssen, and grants and personal fees from Merck, ViiV healthcare, and Gilead outside of the submitted work. AH received a research grant from the funders for advice on statistical design and analysis during the conduct of the study. DW is an employee of DATAMAP. WDFV reports grants from Unitaid during the conduct of the study; grants from the Bill and Melinda Gates Foundation, the South African Medical Research Council, USAID, National Institutes of Health, FIND, Children's Investment Fund Foundation, personal fees from Virology Education, grants, personal fees and non-financial support from ViiV Healthcare, personal fees and non-financial support from Gilead Healthcare, grants, personal fees and non-financial support from MSD, grants, personal fees and non-financial support from Johnson & Johnson, and personal fees from Mylan/Viatris, Adcock-Ingram, Aspen, Abbott, Roche, and Sanofi outside the submitted work. CK and YD declare no competing interests.

## Appendix A Supplementary data

Supplementary Appendix Protocol

Caption for Supplementary Material

Supplementary Appendix Statistical Analysis Plan
