# Supplementary material for: Safety and efficacy of four drug regimens versus standard-of-care for the treatment of symptomatic outpatients with COVID-19: A randomised, open-label, multi-arm, phase 2 clinical trial
Source: eBioMedicine. 2022 Nov 1;86:104322. doi: 10.1016/j.ebiom.2022.104322 (PMC9624152; doi:10.1016/j.ebiom.2022.104322)
Supplement: Supplementary Appendix Protocol [file mmc1.pdf]

SP-PA-COV-202

A phase 2, exploratory, single center, randomized, open label, adaptive clinical trial to compare the safety and efficacy of four different experimental drug regimens to standard of care for the treatment of symptomatic outpatients with COVID-19

**Short Title:**	COVID-19 Treatment South Africa
**Phase:**	2
**Protocol Version and Approval Date:**	Version 4, 17 Nov 2020
**Sponsor:**	Shin Poong Pharm. Co., Ltd.<br>161 Yoksam-ro, Gangnam-gu<br>Seoul, 06246<br>Republic of Korea
**Funder:**	Medicines for Malaria Venture<br>ICC – Block G, 3rd Floor<br>20, Route de Pré-Bois<br>PO Box 1826<br>1215 Geneva 15<br>Switzerland
**Principal Investigator:**	[redacted] PhD, FCP, DTM&H, Dip HIV<br>Divisional Director: Ezintsha, Wits Reproductive Health & HIV Institute<br>University of the Witwatersrand<br>5th Floor, Building D<br>Sunnyside Office Park<br>32 Princess of Wales Terrace<br>Parktown, Johannesburg

CONFIDENTIAL
Property of Shin Poong Pharm. Co., Ltd.
May not be used, divulged, published, or otherwise disclosed without consent

INVESTIGATOR SIGNATURE PAGE

A phase 2, exploratory, single center, randomized, open label, adaptive clinical trial to compare the safety and efficacy of four different experimental drug regimens to standard of care for the treatment of symptomatic outpatients with COVID-19.

I, the undersigned, am responsible for the conduct of the trial at this site and affirm that:

- I agree to personally conduct or supervise the study.
- I have read the protocol and agree that it contains all necessary details for carrying out the study as described. I will conduct this protocol as outlined therein, as per any approved protocol amendments, as per ICH Good Clinical Practice (GCP) and all applicable Regulatory Authority requirements and national laws.
- I will not deviate from the protocol without prior written permission from the Sponsor and prior review and written approval from the Institutional Review Board or Ethics Committee, and Regulatory Authority, except where necessary to prevent immediate danger to the participant.
- I have read and understand the information in the relevant Summary of Product Characteristics, and I am familiar with the Investigational Medicinal Products (IMPs); I also understand the IMP use, including its potential risks and side effects.
- I agree to inform all participants that the IMPs are being used for investigational purposes and I will ensure that the requirements related to obtaining informed consent are in accordance with ICH Guidelines for Good Clinical Practice (GCP) Section 4.8 and local requirements.
- I agree to report adverse events that occur in the course of the study to the Sponsor, to maintain adequate and accurate records and make those records available, in accordance with ICH Guidelines for Good Clinical Practices (GCP) Section 4.11 and local requirements. I agree to promptly report to the Ethics Committee (EC) all changes in the research activity and all unanticipated problems involving risk to the participants.
- I have sufficient time to properly conduct and complete the trial within the agreed trial period, and I will ensure that any qualified staff at my site(s) who are involved in the trial conduct are adequately trained regarding the IMP, the protocol and their responsibilities for the foreseen duration of the trial to conduct the trial properly and safely. If I delegate any of my trial activities, I will provide the Sponsor with a Delegation of Activities Form. I agree to ensure that all associates, colleagues and employees assisting in the conduct of the study are informed about their obligations in meeting the above commitments.
- I understand that the study may be terminated, or enrolment suspended at any time by the Sponsor, with or without cause, or by me if it becomes necessary to protect the best interest of the participants.

______________________________ Date: __________________________

[redacted]

SPONSOR / FUNDER SIGNATORY APPROVAL PAGE

A phase 2, exploratory, single center, randomized, open label, adaptive clinical trial to compare the safety and efficacy of four different experimental drug regimens to standard of care for the treatment of symptomatic outpatients with COVID-19.

I, the undersigned have read this protocol and I approve the design of this trial:

_______________________________ ____________________
[redacted] Date

_______________________________ ____________________
[redacted] Date

_______________________________ ____________________
[redacted] Date

_______________________________ ____________________
[redacted] Date

TABLE OF CONTENTS

INVESTIGATOR SIGNATURE PAGE **2**
SPONSOR / FUNDER SIGNATORY APPROVAL PAGE **3**
ABBREVIATIONS AND TERMS **6**
SYNOPSIS **8**
1. SCHEDULE OF ACTIVITIES **15**
2. INTRODUCTION **18**
2.1 Background 19
2.2 Study Rationale 19
2.2.1 COVID-19 and antiviral approaches 19
2.2.2 Drug selection 20
2.2.3 Dose selection 22
2.2.4 Study design 23
2.2.5 Pharmacokinetic-pharmacodynamic assessments 23
2.3 Benefit/Risk Assessment 23
3. OBJECTIVES AND ENDPOINTS **24**
4. STUDY DESIGN **25**
4.1 Overall Design 25
4.2 Participant and Study Completion 26
5. STUDY POPULATION **26**
5.1 Inclusion Criteria 27
5.2 Exclusion Criteria 27
5.3 Co-enrollment Guidelines 28
6. TREATMENTS **28**
6.1 Treatments Administered 28
6.2 Risks to the Participants 29
6.2.1 Management of participants to limit risks of SARS-CoV-2 transmission 30
6.3 Dose Modification and Toxicity Management 31
6.4 Method of Treatment Assignment 32
6.5 Blinding 32
6.6 Preparation / Handling / Storage / Accountability 32
6.7 Treatment Compliance 32
6.8 Concomitant Therapy 32
6.8.1 Prohibited medications 32
6.8.2 Precautionary medications 33
6.9 Treatment After the End of the Study 34
7. DISCONTINUATION/WITHDRAWAL CRITERIA **34**
7.1 Discontinuation of Study Treatment 34
7.2 Withdrawal from the Study 34
7.3 Lost to Follow-up 35
8. STUDY ENCOUNTERS **35**
8.1 Screening Evaluation: Day 0 (-1 day window) 35
8.2 Enrollment/Randomization/Start of Treatment: Day 1 36
8.3 Treatment Continuation and Follow-Up: Day 2 to Day 14 (±2) 37
8.4 Late Follow-Up: Day 21 (±2) 39
8.5 End-of-Study: Day 28 39
9. STUDY ASSESSMENTS AND PROCEDURES **40**
9.1 Efficacy Assessments 40
9.1.1 Mid-nasal swab and saliva specimen 40
9.1.2 Participant surveys 41
9.1.3 WHO Ordinal Scale for Clinical Improvement 41
9.2 Adverse Events 41
9.2.1 Definition 42
9.2.2 Serious adverse events 42
9.2.3 Treatment-related AEs and SAEs 43

9.3 Treatment of Overdose 43
9.4 Pregnancy 43
9.5 Other Safety Assessments 43
9.6 Serological and Other Assessments 44
9.7 PK Assessments 44
9.8 Biohazard Containment 45
10. DATA MANAGEMENT 45
11. STATISTICAL CONSIDERATIONS 45
11.1 Sample Size Determination 45
11.2 Populations for Analyses 46
11.3 Statistical Analyses 46
11.3.1 Efficacy analyses 47
11.3.2 Secondary endpoints 47
11.3.3 Exploratory endpoints 48
11.3.4 Combined study analysis 48
12. STUDY MONITORING 48
12.1 Safety Review Team 48
12.2 Independent Monitor 48
12.3 Data Monitoring Committee 49
12.4 Scientific Advisory Committee 49
13. ETHICAL CONSIDERATIONS 49
13.1 Regulatory and Ethical Considerations 49
13.2 Informed Consent Process 50
13.3 Study Records 50
13.4 Confidentiality 50
13.5 Blood Volume 51
14. REFERENCES 52
APPENDICES 56
APPENDIX 1: STUDY GOVERNANCE 56
APPENDIX 2: CONTACT DETAILS 57
APPENDIX 3: THE INFLUENZA PATIENT-REPORTED OUTCOME INSTRUMENT (FLU-PRO© PLUS) 60
APPENDIX 4: DAILY SURVEY 62
APPENDIX 5: WHO ORDINAL SCALE FOR CLINICAL IMPROVEMENT 65
APPENDIX 6: PROHIBITED CONCOMITANT MEDICATIONS 66

ABBREVIATIONS AND TERMS

Term	Definition
ACE2	Angiotensin-converting enzyme 2
ACT	Artemisinin-based combination therapy
AE	Adverse event
ALT	Alanine aminotransferase
AQ	Amodiaquine
ASAQ	Artesunate-amodiaquine
AST	Aspartate aminotransferase
COVID-19	Coronavirus disease
DAIDS	Division of Acquired Immunodeficiency Syndrome
DEAQ	Desethylamodiaquine
DMC	Data Monitoring Committee
EC_{50}	Half-maximal effective concentration
eCRFs	Electronic case report forms
Eligible	Qualified for enrollment into the study based upon inclusion/exclusion criteria
FLU-PRO	Influenza patient-reported outcome
FPV-NTZ	Favipiravir plus nitazoxanide
GCP	Good Clinical Practice
IATA	International Air Transport Association
ICF	Informed consent form
IMP	Investigational medicinal product
IRB	Institutional Review Board
IVRS	Interactive voice response system
IWRS	Interactive web response system
LRTI	Lower respiratory tract infection
MERS-CoV	Middle East respiratory syndrome coronavirus
Mpro	Main protease
NSAID	Non-steroidal anti-inflammatory drug
PA	Pyronaridine-artesunate
Participant(s)	Term used throughout the protocol to denote the enrolled individual(s)
PBPK	Physiologically based pharmacokinetics
PCR	Polymerase chain reaction
PK	Pharmacokinetic(s)
PPE	Personal protection equipment
PY	Pyronaridine
QTcF	QT-interval corrected for heart rate in accordance with Fridericia's formula
PKPD	Pharmacokinetic-pharmacodynamic
RCT	Randomized controlled trial
RdRp	RNA-dependent RNA-polymerase
RNA	Ribonucleic acid
RR	Respiratory rate
RT-PCR	Reverse transcription polymerase chain reaction
SAE	Serious adverse event

SAHPRA	South African Health Products Regulatory Authority
SARS-CoV	Severe acute respiratory syndrome coronavirus
SARS-CoV-2	Severe acute respiratory syndrome coronavirus 2
SmPC	Summary of product characteristics
SoA	Schedule of activities
SOC	Standard of care
SOF/DCV	Sofosbuvir/daclatasvir
SpO2	Oxygen saturation
TdP	Torsades de pointes
ULN	Upper limit of normal
WHO	World Health Organization

SYNOPSIS

**Title**	A phase 2, exploratory, single center, randomized, open label, adaptive clinical trial to compare the safety and efficacy of four different experimental drug regimens to standard of care for the treatment of symptomatic outpatients with COVID-19.
**Short Title**	COVID-19 Treatment South Africa
**Rationale**	Severe acute respiratory syndrome coronavirus 2 (SARS-CoV-2) infection causes mild illness confined to the upper respiratory tract in approximately 80% of people but can cause severe lower respiratory tract infection (LRTI) in 20%, particularly among those in high-risk groups, defined by advanced age (>60 years) and presence of comorbidities (e.g., cardiopulmonary disease, hypertension, diabetes mellitus, obesity, renal disease). Progression to LRTI appears to frequently result in hospitalization for supplemental oxygen therapy and may lead to a need for ventilator respiratory support and ultimately death. Given the rapid spread of the SARS-CoV-2 pandemic, interventions that avert adverse patient outcomes and reduce the strain on the medical system are urgently needed. In addition, prolonged viral shedding has been noted after infection, especially soon after infection; therapeutic strategies that can effectively reduce viral shedding, and potentially onward transmission, have the potential to shift the trajectory of the pandemic.<br><br>As of the beginning of May 2020, no therapeutic agents had been approved for the treatment of COVID-19, although many scientists and researchers have proposed a number of potentially suitable existing drugs based on *in vitro,* pre-clinical, or limited clinical studies. These agents have potential benefits but also have inherent risks and limitations (e.g., adverse reactions and scalability of supply). There is an urgent need for controlled and adequately powered clinical studies in relevant patient populations to guide policy makers, regulators and other clinical trialists on prioritized drugs, including a path toward future evaluations as data become available. This study intends to rapidly provide data to inform early decision-making and/or support combined analysis with other concurrently run studies.<br><br>Our understanding of the viral pathogenesis of SARS-CoV-2 remains limited. However, it appears that the virus cell entry depends on the binding of the viral spike (S) proteins to cellular receptors, and on S protein priming by host cell proteases. SARS-CoV-2, like SARS-CoV, uses the same receptor angiotensin converting enzyme 2 (ACE2) on pulmonary epithelial cells for entry and the transmembrane serine protease 2 for S protein priming [1]. The receptor binding domain of lineage B betacoronaviruses is a single, continuous domain that contains all of the structural information necessary to interact with the host receptor. Fusion is mediated at the cell membrane, delivering the viral nucleocapsid inside the cell for subsequent replication. A recent retrospective analysis of 85 patients with laboratory-confirmed COVID-19 also indicated that SARS-CoV-2 infects human kidney tubules and induces acute tubular damage in some patients [2], and 2–11% of patients exhibit liver comorbidities [3]. COVID-19 has also been associated with multiple direct and indirect cardiovascular complications including acute myocardial injury, myocarditis, arrhythmias, and venous thromboembolism [4,5]. Therefore, therapeutic options that provide antiviral concentrations of drug(s) within the systemic circulation as well as other affected organs are likely to be required.<br><br>Medications to treat and/or prevent SARS-CoV-2 need to inhibit aspects of the viral life cycle, ultimately blocking replication. Already-approved and available

medications are ideal for immediate evaluation for SARS-CoV-2 infection treatment and prevention. Clinical studies are already ongoing for SARS-CoV-2 using various repurposed antiviral medicines (https://www.who.int/emergencies/diseases/novel-coronavirus-2019/global-research-on-novel-coronavirus-2019-ncov).

Drugs were selected for this study on the basis of an analysis of the human pharmacokinetics of all agents reported to have anti-SARS-CoV-2 activity *in vitro*. Specifically, the human maximum plasma concentration (C_{max}) and predicted (calculated) maximum lung concentration after administration of the licensed doses were used to assess whether concentrations would exceed those required to inhibit at least 50% of SARS-CoV-2 replication *in vitro* (EC_{50}) for at least some of the dosing interval. The full analysis has been published [6] and was supplemented by in-house data for 4-aminoquinolines available at MMV [7].

All study drugs, with the exception of pyronaridine, were predicted to provide concentrations with at least some antiviral activity in plasma, with favipiravir and nitazoxanide providing the greatest plasma ratios over their reported activity (see figure beneath).

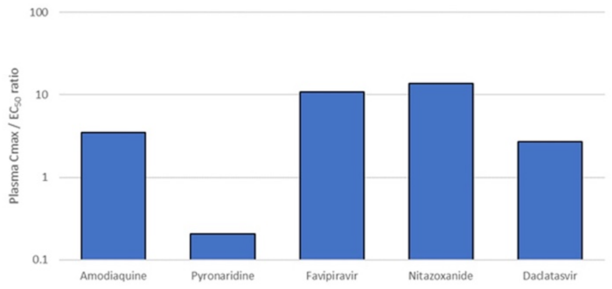

Similarly, based on the calculated lung concentrations, all study drugs were also predicted to reach concentrations with some activity in lung, but predicted lung exposures for amodiaquine and pyronaridine were estimated to be profoundly higher than the reported anti-SARS-CoV-2 EC_{50} (see figure beneath). For amodiaquine, desethylamodiaquine and pyronaridine, this is supported by PBPK simulations which predict a high distribution of the compounds into the lungs.

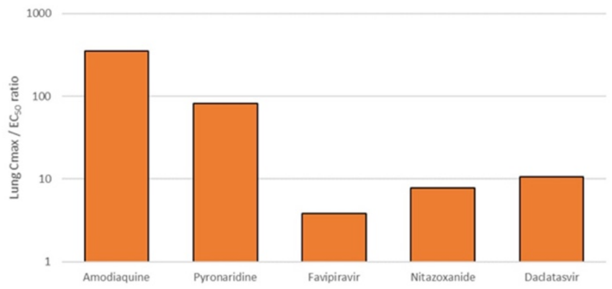

These data alone support the rationale for assessing these agents in combination for COVID-19 therapy.

This phase 2, exploratory study will be an adaptive, randomized, open label, trial for treatment of individuals in an outpatient settings with mild SARS-CoV-2 infection. The primary outcome is focused on the evaluation of efficacy of the proposed experimental drugs in reducing upper respiratory viral shedding, defined as viral clearance (i.e., negative swab) on Day 7. Key secondary outcomes focus on other measures of viral shedding, safety evaluation, progression to LRTI

	(defined by resting blood oxygen saturation level [SpO2] <93% sustained for two readings two hours apart **and** presence of subjective dyspnea or cough), disease severity, clinical resolution rate, and cumulative incidence of hospitalization or mortality at Day 28.<br><br>Assessment of an initial virological impact will inform further evaluation in a subsequent, separate, confirmatory, phase 3 trial to evaluate the efficacy of promising experimental drugs in terms of clinical outcomes.
**Design**	This exploratory study is a randomized, single center, open label study of four different experimental treatment arms versus standard of care for the treatment of SARS-CoV-2 infection in symptomatic outpatients with mild disease at the time of enrolment.
**Population**	Women and men from 18 to 65 years of age, inclusive, with self-reported symptoms of COVID-19 for no more than 72 hours prior to screening AND who test positive for SARS-CoV-2 at screening or during the 2 days prior to screening, will be enrolled. The eligibility criteria have been set to identify participants with mild COVID-19 disease in accordance with national guidelines [8].<br><br>The following eligibility criteria will be used to select study participants.<br><br>Inclusion criteria:<br>1. Age from 18 to 65 years of age, inclusive, at the time of signing the informed consent form.<br>2. Willing and able to provide informed consent.<br>3. Women of reproductive potential must be using a highly effective method of contraception for at least 28 days prior to enrolment and must be able and willing to continue its use throughout the duration of the study.<br>4. Men must agree to use condoms when engaging in heterosexual sex during the study and for the period up to 91 days after the last dose of study medication. Men who are not randomized to a treatment arm including favipiravir (or another arm identified as having teratogenic potential through semen) will no longer need to adhere to this after randomization.<br>5. Laboratory confirmed SARS-CoV-2 infection, and any of the following self-reported symptoms with onset no more than 72 hours prior to screening informed consent, and still present at randomization: fever or chills, cough, myalgia, sore throat, headache, conjunctivitis, shortness of breath, nausea, diarrhea, new onset of anosmia or ageusia, or other symptoms recognized in local and international guidelines as typical of mild COVID-19.<br>6. Body weight ≥45 kg.<br>7. Access to reliable video conference, telephone, direct/text messaging, or other device permitting real-time, reliable information transfer.<br><br>Exclusion criteria:<br>1. Pregnant or lactating women.<br>2. Known hypersensitivity or specific contraindications to the use of any of the active drugs in the treatment arms or similar compounds.<br>3. Duration of self-reported symptoms of COVID-19 for more than 96 hours or four calendar days (whichever is greater) prior to randomisation.<br>4. Signs of respiratory distress prior to randomization, including:<br>o respiratory rate >24 breaths/min<br>o SpO2 <95% in room air.<br>5. Resting pulse rate ≥120 beats/min.<br>6. High likelihood of hospitalization in the opinion of the attending clinician.<br>7. QTcF >470 msec for females, or >450 msec for males, at screening.<br>8. Serum potassium < 3.5 mmol/L at screening.

	9. History of clinically significant cardiovascular disease (including arrhythmias, QT-interval prolongation, torsades de pointes (TdP), history of coronary artery disease with graft or stent procedures/surgery, cardiac failure [class 2 or higher using the New York Heart Association functional classification]).<br>10. Known chronic kidney disease (Stage IV or receiving dialysis).<br>11. Known cirrhosis (Child-Pugh Class B or greater).<br>12. Known macular degeneration, or other known retinal diseases, or 4-aminoquinolone-induced visual impairment.<br>13. Currently receiving, or recently received (within 60 days prior to randomization) treatment with any of the drugs in the experimental treatment arms (Arms B to E).<br>14. Currently receiving, or recently received (within 30 days prior to randomization) treatment with any antimalarial drugs.<br>15. Currently on treatment with drugs with known arrhythmogenic potential, or those known to induce significant QT-interval prolongation or TdP, as detailed in Appendix 6.<br>16. Currently on treatment for tuberculosis (or on treatment with rifampicin for any other indication), or on treatment with a protease inhibitor-based antiretroviral regimen, or efavirenz, or carbamazepine.<br>17. Inability/unlikely to be in the study area for the duration of the 28 day follow-up period.<br>18. Any surgical or medical condition which might significantly alter the absorption, distribution, metabolism, or excretion of drugs, or which may jeopardize the safety of the volunteer or the objectives of the study. The Investigator should make this determination in consideration of the volunteer's medical history.<br>19. Personnel (e.g. investigator, sub-investigator, research assistant, pharmacist, study coordinator or anyone mentioned in the delegation log) directly involved in the conduct of the study.<br>20. Participant is judged by the Investigator to be at significant risk of failing to comply with the provisions of the protocol as to cause harm to self or seriously interfere with the validity of the study results.<br><br>Volunteers may be re-screened if they are found not to have COVID-19 at the time of the initial screening, but develop a further, and separate acute illness suggestive of COVID-19 at a later stage again.<br><br>Participants will be counseled about the preliminary *in vitro* data on the activity of the planned treatment arms against SARS-CoV-2 and equipoise regarding efficacy in humans, given that there are only limited data from an uncontrolled study at this time.
**Interventions**	The following treatment arms are planned:<br>• Arm A: Standard of care (SOC)<br>• Arm B: SOC plus artesunate-amodiaquine (ASAQ)<br>• Arm C: SOC plus pyronaridine-artesunate (PA)<br>• Arm D: SOC plus favipiravir plus nitazoxanide (FPV-NTZ)<br>• Arm E: SOC plus sofosbuvir/daclatasvir (SOF/DCV)<br><br>Based on the availability of the investigational drugs, a decision may be made to proceed with fewer than five arms initially, and to commence the remaining arms as soon as the relevant investigational products are available.<br><br>Current standard of care (SOC) will serve as the control arm and will also be provided to all participants in accordance with local guidelines which are subject

	to change. Any changes to recommended SOC will be discussed by the study scientific advisory committee to determine the impact on existing assessments and treatment arms. At the time of drafting this protocol, SOC comprises paracetamol 1 g 6-hourly taken orally as needed. Other optional symptomatic therapies (e.g., throat lozenges) may also be provided based on availability and need, and participants will be advised to rest.	
**Objectives and Endpoints**	<u>Primary Objective</u>	<u>Primary Endpoint</u>
	• To test the efficacy of each treatment regimen compared to SOC alone in reducing SARS-CoV-2 viral shedding.	• Incidence of SARS-CoV-2 clearance (defined as the proportion of participants with a negative SARS-CoV-2 RT-PCR test) on Day 7.
	<u>Secondary Objectives</u>	<u>Secondary Endpoints</u>
	• To test the efficacy of each treatment regimen compared to SOC alone in reducing SARS-CoV-2 viral shedding as determined by viral culture	• Incidence of SARS-CoV-2 clearance on Day 7 (defined as the proportion of participants with negative SARS-CoV-2 viral cultures on both Day 7 and Day 10 in the subset of participants for whom these assessments are performed and results are available)
	• To test the efficacy of each treatment regimen compared to SOC alone in reducing late SARS-CoV-2 viral shedding	• Incidence of SARS-CoV-2 clearance (defined as the proportion of participants with negative SARS-CoV-2 RT-PCR tests) on Days 10, 14, 21 and 28
	• To test the efficacy of each treatment compared to SOC alone in reducing the duration of SARS-CoV-2 viral shedding	• Time to clearance of SARS-CoV-2, defined as a negative SARS-CoV-2 RT-PCR test (samples collected Days 0, 3, 7, 10, 14, 21 and 28)
	• To test whether each treatment regimen is associated with decreased viral shedding over 14 days compared to SOC alone	• Estimated viral load of SARS-CoV-2 detected by quantitative RT-PCR
	• To test whether each treatment regimen decreases the resolution rate for symptomatic SARS-CoV-2 infection/COVID-19 disease compared to SOC alone	• Proportion of days with fever after randomization<br>• Proportion of days with respiratory symptoms after randomization<br>• FLU-PRO© Plus questionnaire scores and FLU-PRO© Plus Global Additional Daily Diary Items over the first 14 days
	• To test the safety of each treatment regimen compared to SOC alone for treatment of outpatients with SARS-CoV-2 infection	• Serious adverse events (including death and hospitalization)<br>• Adverse events resulting in treatment discontinuation<br>• Adverse events considered related to the investigational products

	• To test the efficacy of each treatment regimen compared to SOC alone to prevent progression to LRTI	• LRTI, defined by resting SpO2<93% sustained for 2 readings 2 hours apart AND presence of subjective dyspnea or cough
	• To test whether each treatment regimen has an effect on disease severity compared to SOC alone	• Maximum score on WHO Ordinal Scale for Clinical Improvement during study participation
	• To test the efficacy of each treatment regimen compared to SOC alone in reducing hospitalization	• Cumulative incidence of hospitalization at Day 28
	• To test whether each treatment regimen has an effect on the duration of hospitalization among persons who become hospitalized with COVID-19 disease, compared to SOC	• Days of hospitalization
	• To test the efficacy of each treatment regimen compared to SOC alone in reducing mortality.	• Cumulative incidence of mortality, measured at Day 28 or later if participant is hospitalized at the time of Day 28.
	Exploratory Objectives	Exploratory Endpoints
	• To investigate drug exposure of the study drugs in patients with COVID-19	• Plasma/whole blood C_{min} concentrations on Day 3 (Arms B and C) and Day 7 (Arms B to E)
	• To explore the relationship between efficacy and plasma or whole blood concentrations of the study drugs for each treatment regimen	• Viral clearance<br>• Categorical secondary endpoints related to efficacy<br>• Plasma/whole blood C_{min} concentrations on Day 7
	• To explore the immune response in symptomatic patients infected with SARS-CoV-2	• Seroconversion as assessed by means of a validated serological assay still to be determined
	• To explore the relationship between sample RT-PCR results, their cycle threshold values, and corresponding viral culture results for SARS-CoV-2.	• SARS CoV-2 qualitative and quantitative RT-PCR results and cycle threshold values from all relevant time points, and viral culture results from screening, Day 7 and Day 10 in the subset of participants for whom viral culture is performed.
**Sample Size**	Assuming that the percentage of participants with viral clearance by Day 7 is 20% in the control arm (Arm A), a sample size of 50 participants per arm will provide at least 80% power to detect an increase in viral clearance to 50% for a new treatment. This assumes a two-sided, 5% type 1 error rate and a loss to follow up rate of 20%. The assumptions for this calculation are supported by a published analysis of a trial of favipiravir (FPV) versus lopinavir/ritonavir (LPV/RTV), where the percentage with viral clearance by Day 7 was 65% for favipiravir versus 20% for LPV/RTV [9]. Issues of multiplicity have not been considered in this exploratory study.	

	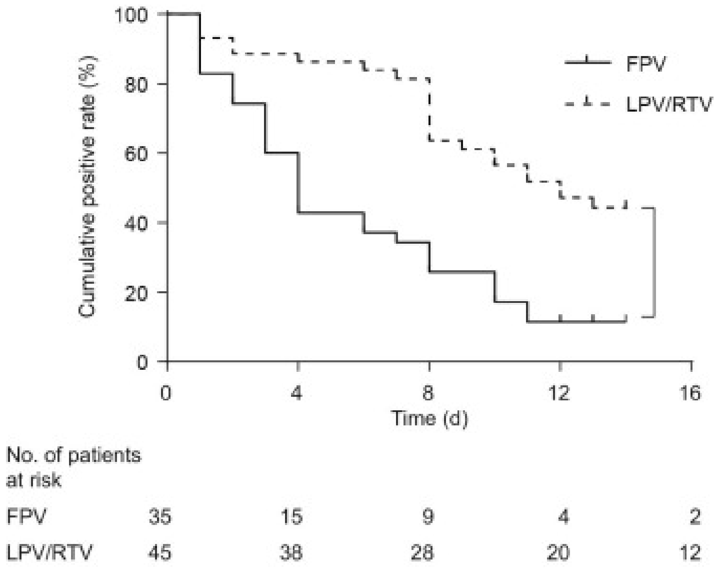 The aim of this exploratory study is to demonstrate similar high proportions of viral clearance for new candidate treatments. Demonstration of these benefits in any study treatment arms would justify taking these forward into a subsequent, separate, confirmatory, phase 3 trial, while dropping those arms which fail to achieve an improved rate of viral clearance versus the control treatment.
**Duration**	Twenty-eight days of clinical follow-up per participant unless follow-up of an unresolved serious adverse event necessitates an extended follow-up period.
**Site**	Administrative Institution:<br>Ezintsha, Wits Reproductive Health & HIV Institute (Wits RHI)<br>University of the Witwatersrand<br>5th Floor, Building D<br>Sunnyside Office Park<br>32 Princess of Wales Terrace<br>Parktown, Johannesburg<br><br>Main Site:<br>Building C<br>Sunnyside Office Park<br>32 Princess of Wales Terrace<br>Parktown, Johannesburg<br><br>Satellite Site:<br>Charlotte Maxeke Johannesburg Academic Hospital<br>Area 585, Level 8, Jubilee Road<br>Johannesburg, 2193<br>South Africa

1. SCHEDULE OF ACTIVITIES

Procedure	Screen-ing	Enroll-ment	Treatment and Follow-Up Period (Including self-quarantine as required in accordance with local guidelines[a])								Late Follow-Up		EOS
	Day 0 (-1)[b]	Day 1	Day 2	Day 3 (+1)	Days 4-6	Day 7 (±1)	Days 8-9	Day 10 (±1)	Days 11-13	Day 14 (±2)	Days 15-20	Day 21 (±2)	Day 28 (±2)[c]
Screening informed consent	X												
Demography	X												
Duration of COVID-19 symptoms	X												
Pregnancy and lactation status and urine pregnancy test[d]	X												
Mid-nasal swab and saliva specimen[e]	X[f]			X[g]		X[g]		X[g]		X[g]		X[g]	X[g]
Blood for serum potassium	X												
Dispense 48-hour "Flu Pack"[h]	X												
Inclusion and exclusion criteria[i]	X	X											
Main informed consent, optional storage consent		X											
Past and current medical conditions		X											
Concomitant medications, including contraception		X											
Physical examination, including weight and height		X											
Vital signs (temperature, pulse, RR, SpO2)		X											
ECG, including assessment of QTcF interval[j]		X											
Randomization		X[k]											
Blood collection for serology[l]		X											X
Optional blood collection for samples for storage[m]		X											X
Participant collected daily vitals (temperature, pulse and respiratory rate)[n]		X	X	X	X	X	X	X	X	X		X	X
Participant collected daily SpO2[o]		X	X	X	X	X	X	X	X	X			
Completion of daily survey[p]		X	X	X	X	X	X	X	X	X		X	X
Completion of FLU-PRO© Plus questionnaire[q]		X	X	X	X	X	X	X	X	X		X	X
WHO Ordinal Scale for Clinical Improvement[r]		X				X				X		X	X
Study therapy		X[s]	X[s]	X[s]	X[s]	X[s]							
PK sampling				X[t]		X[u]							
Contact with study clinician or staff[v]	X	X	X	X	(X[w])	X	(X[w])	X	(X[w])	X	(X[w])	X	X
Adverse event review[x]		X	X	X	X	X	X	X	X	X	X	X	X
Return of unused IMP and empty containers													X[y]

EOS: end of study; PK: pharmacokinetics; SOC: standard of care; SpO2: oxygen saturation; RR: respiratory rate; WHO: World Health Organization.

Note: Participants who stop study product should continue study participation off study product with continued evaluations as per the schedule of activities. The reason for study product discontinuation should be recorded.

[a] Current local guidelines specify a quarantine duration for patients with mild COVID-19 of 10 days from onset of symptoms provided resolution of fever has occurred and other symptoms are improving [8]. Instructions to participants will be updated if the local guidelines are updated without amending the protocol.

[b] Screening will be conducted at the study site. A window period of -1 day may apply as long as COVID-19 symptoms are not present for more than 96 hours or 4 calendar days (whichever is greater) prior to randomization. Study personnel will wear personal protective equipment during all interactions, and participants and study personnel will be instructed to adhere to local guidance for COVID-19-related safety measures. Screening (Day 0) and Day 1 evaluations may occur on the same calendar day depending on the availability of the RT-PCR test results.

[c] For participants who are hospitalized at the time of the scheduled Day 28 visit, the last visit will be approximately 3 days after discharge.

[d] Urine pregnancy test for women of reproductive potential only.

[e] Or other specimen depending on best practice recommendations and the availability of a suitable, validated assay.

[f] Participants that have tested positive for SARS-CoV-2 infection (RT-PCR) in the 2 days prior to screening and have laboratory confirmation of this require a mid-nasal swab and saliva specimen (or other specimen as identified) for RT-PCR quantification and viral culture of SARS-CoV-2 at screening only.

[g] Participants will be visited by laboratory personnel or a study team member or community healthcare worker (CHW), or may report to the site if the quarantine period has been completed, to facilitate this. Laboratory personnel/study personnel/CHWs will wear personal protective equipment during all required interactions, and participants and laboratory personnel/study personnel/CHWs will be instructed to adhere to local guidance for COVID-19-related safety measures. Qualitative RT-PCR for SARS-CoV-2 will be performed at all time points. Quantitative RT-PCR will be performed at all time points up to and including Day 14. Viral culture will be performed at Day 7 and Day 10 only.

[h] 48-Hour “flu pack” to be dispensed to all participants in accordance with local standard operating procedures and current national treatment guideline.

[i] Eligibility criteria related to age, duration of COVID-19 symptoms, pregnancy status, qualitative RT-PCR and potassium results will be assessed on Day 0 or when the Day 0 results are available; all other eligibility criteria will be assessed on Day 1 after completion of the relevant assessments.

[j] Using a validated method.

[k] Participants who meet all eligibility criteria will be randomized to receive study treatment. Study treatment packs will be dispensed or delivered to participants, or collected by participants from the site.

[l] Blood samples (5 mL at each time point) will be collected for serological testing in accordance with emerging, validated assays from randomized participants.

[m] Blood samples (up to 20 mL at each time point) will be collected from randomized participants providing optional consent for this. Samples will be stored at -70 °C for a period of up to 2 years. The samples will be used for future testing in accordance with emerging disease-related data, and suitable, validated assays.

[n] The Day 1 assessments will be performed by randomized participants under observation of the site personnel. Assessments will be recorded utilizing a mobile phone/tablet application, or other device, or diary, or during a telephonic or in person interaction (once the quarantine period has completed) with the site personnel. Assessments may be performed by study personnel if an in person visit is conducted after completion of the quarantine period.

[o] The Day 1 assessment will be performed by randomized participants under observation of the site personnel. To be measured after at least 5 minutes resting in a sitting or supine position. Participants with an SpO2 <93% will repeat the assessment 2 hours later. If the repeat assessment is <93%, they will contact the site immediately to report this. All results will be recorded utilizing a mobile phone/tablet application, or other device, or diary, or during a telephonic or in person interaction (once the quarantine period has completed) with the site personnel.

[p] To be recorded during a telephonic or in person (once the quarantine period has completed) interaction with the site personnel, or utilizing a mobile phone/tablet application, or other device, or diary. The daily survey will consist of the FLU-PRO© Plus Global Additional Daily Diary Items (related to general well-being, overall severity of symptoms, and

ability to perform usual daily activies) as well as other general daily diary questions related to study drug administration, recording of vital signs and SpO2 measurements, review of concomitant medications, and information related to possible adverse events.

[q] The Influenza Patient-Reported Outcome instrument (FLU-PRO© Plus). To be recorded during a telephonic or in person (once the quarantine period has completed) interaction with the site personnel, or utilizing a mobile phone/tablet application, or other device, or diary.

[r] To be performed by study team personnel. The Day 1 assessment should be performed prior to the first dose of IMP.

[s] To be taken as prescribed, for up to 7 days depending on the treatment arm to which the participant is randomized. Includes SOC for all treatment arms.

[t] A Day 3 PK sample will be drawn from participants in the ASAQ and PA arms in accordance with the timing in Section 9.7.

[u] A Day 7 PK sample will be drawn from all participants in the experimental arms (Arms B to E) in accordance with the timing in Section 9.7.

[v] To be performed utilizing a web-conference, telephone call or text/direct messaging, or in person if the quarantine period has been completed.

[w] These evaluations will be as needed/requested by study participant.

[x] Serious adverse events, adverse events resulting in treatment discontinuation, and all other AEs, including those judged by the site Investigator to be related to the administered treatment regimens.

[y] Or anytime from Day 7 if an earlier contact visit takes place.

2. INTRODUCTION

This is a randomized, single center, open label, adaptive, exploratory trial for the treatment of severe acute respiratory syndrome coronavirus 2 (SARS-CoV-2) infection in adults with mild disease not requiring hospital admission. The trial will compare four different experimental treatment arms to SOC.

Assessments will be conducted to assess the effect of each dosing regimen in decreasing viral shedding. Evaluations include SARS-CoV-2 viral shedding, progression to lower respiratory tract infection (LRTI), and safety and tolerability. A plasma or whole blood sample will be collected from each participant on Day 7 in order to assess plasma concentrations at the primary endpoint, and on Day 3 from participants in the artesunate-amodiaquine and pyronaridine-artesunate arms.

Enrolment to each of the planned, currently identified study arms (Arms A to E) will commence as soon as the relevant drugs are available after study initiation (i.e., different treatment arms may be opened up at different times depending on the availability of the IMPs).

Up to 250 eligible adults (18 to 65 years of age) will be randomized in an equal allocation ratio to receive one of the following therapies:

- Arm A: Standard of care (SOC)
- Arm B: SOC plus artesunate-amodiaquine (ASAQ)
- Arm C: SOC plus pyronaridine-artesunate (PA)
- Arm D: SOC plus favipiravir plus nitazoxanide (FPV-NTZ)
- Arm E: SOC plus sofosbuvir/daclatasvir (SOF/DCV).

Current SOC will be provided to all participants in accordance with local guidelines which are subject to change. Any changes to recommended SOC will be discussed by the study Scientific Advisory Committee to determine the impact on existing assessments and treatment arms. At the time of drafting this protocol, SOC comprises paracetamol 1 g 6-hourly taken orally as needed. Other optional symptomatic therapies (e.g., throat lozenges) may also be provided based on availability and need, and participants will be advised to rest.

During the study, participants will take the IMP associated with the treatment arm to which they are randomized, and will perform the following:

- Complete daily vital signs assessments (temperature, pulse rate and respiratory rate). Participants will also measure SpO2 daily and evaluate themselves for symptoms and signs of LRTI.
- Complete surveys that will include questions about general well-being, respiratory and systemic symptoms, adherence to the study medication dosing schedule, review of adverse events (AEs) and concomitant medications, and other issues which may emerge as important to track on a daily basis.

Additional assessments (collection of mid-nasal swabs and saliva samples [or other specimen depending on best practice recommendations and the availability of suitable, validated assays] for qualitative and quantitative reverse transcription polymerase chain reaction [RT-PCR] and viral culture detection of SARS-CoV-2, AE evaluation, PK sampling and clinical improvement assessment) will be performed by study team personnel or laboratory personnel.

2.1 Background

SARS-CoV-2 is a coronavirus novel to the human population and discovered in December 2019; it is currently the cause of a global pandemic [11,12,13]. The World Health Organization (WHO) named the novel coronavirus SARS-CoV-2 and the disease caused by the virus COVID-19.

SARS-CoV-2 has spread rapidly across the world, with the first case confirmed in South Africa on 5 March 2020. As of 30 June 2020, there were 151,209 confirmed cases in South Africa, with 2,657 deaths [14]. Despite scale up in screening and testing, accurate reporting is limited by availability of diagnostic testing. WHO declared the COVID-19 pandemic a Public Health Emergency of International Concern on 30 January 2020 [15], and a pandemic on 11 March 2020 [16]. The United States declared a national emergency on 13 March 2020 [17], and South Africa declared a national state of disaster on 15 March 2020 [18].

Severe pneumonitis and most deaths have occurred in the elderly or in persons with underlying pulmonary or cardiac comorbidities, hypertension or diabetes. In healthy adults, including pregnant women, infection with SARS-CoV-2 can be asymptomatic, or cause a febrile, self-limited respiratory infection in the majority of cases. The disease can be severe, however, in a small percentage of patients. Infection appears less symptomatic in children and younger adults [19]. Manifestations of COVID-19 in persons with comorbidities, such as HIV infection, tuberculosis or chronic malaria, are not well-characterized. Nevertheless, the burden of this pandemic to the global health and economic systems is expected to be substantial. No acquired immunity to this novel viral infection appears to exist in the human population globally, and no effective treatment or preventative agent is licensed at this time.

As with many infectious epidemics, household contacts, first responders, caregivers, and medical personnel attending persons with COVID-19 are at high risk of infection. The incubation time requires 14 days of quarantine for exposed individuals not wearing personal protective equipment [20], and on 3 March 2020, WHO declared a global shortage of personal protective equipment, leaving doctors, nurses, and other frontline workers dangerously ill-equipped to care for COVID-19 patients [21]. Extensive absences from the care network and health system will degrade the ability to care not only for those with COVID-19 but also for routine healthcare issues as well. At the height of local epidemic, the already stretched health care system is expected to become overburdened with patients with respiratory illness. To date, rigorous self-isolation and lockdown have been required to contain SARS-CoV-2, leading entire societies to abruptly stop normal life. Interventions are urgently needed to stop viral spread and to decrease the morbidity and mortality associated with the infection. The ability to stop viral replication to prevent transmission of the virus and to prevent LRTI, which is associated with need for hospitalization and possibly mechanical ventilatory support, will be of benefit to the individual, the hospital system, and the health of the public.

2.2 Study Rationale

2.2.1 COVID-19 and antiviral approaches

SARS-CoV-2 is a novel betacoronavirus of zoonotic origin, similar to the coronaviruses severe acute respiratory syndrome coronavirus (SARS-CoV) and Middle East respiratory syndrome coronavirus (MERS-CoV). Based on current evidence, case fatality rate for SARS-CoV-2 is about 3%, which is significantly lower than that of SARS-CoV (10%) and MERS-CoV (40%) [22]. However, SARS-CoV-2 has potentially higher transmissibility (R_0: 1.4 to 5.5) than both SARS-CoV (R_0: 2 to 5) and MERS-CoV (R_0: <1).

Our understanding of the viral pathogenesis of SARS-CoV-2 remains limited. However, it appears that the virus cell entry depends on the binding of the viral spike (S) proteins to cellular receptors, and on S protein

priming by host cell proteases. SARS-CoV-2, like SARS-CoV, uses the same receptor angiotensin converting enzyme 2 (ACE2) on pulmonary epithelial cells for entry and the transmembrane serine protease 2 for S protein priming [1]. The receptor binding domain of lineage B betacoronaviruses is a single, continuous domain that contains all of the structural information necessary to interact with the host receptor. Fusion is mediated at the cell membrane, delivering the viral nucleocapsid inside the cell for subsequent replication. A recent retrospective analysis of 85 patients with laboratory-confirmed COVID-19 also indicated that SARS-CoV-2 infects human kidney tubules and induces acute tubular damage in some patients [2], and 2–11% of patients exhibit liver comorbidities [3]. COVID-19 has also been associated with multiple direct and indirect cardiovascular complications including acute myocardial injury, myocarditis, arrhythmias, and venous thromboembolism [4,5]. Therefore, therapeutic options that provide antiviral concentrations of drug(s) within the systemic circulation as well as other affected organs are likely to be required.

Medications to treat and/or prevent SARS-CoV-2 need to inhibit aspects of the viral life cycle, ultimately blocking replication. Already-approved and available medications are ideal for immediate evaluation for SARS-CoV-2 infection treatment and prevention. Clinical studies are already ongoing for SARS-CoV-2 using various repurposed antiviral medicines (https://www.who.int/emergencies/diseases/novel-coronavirus-2019/global-research-on-novel-coronavirus-2019-ncov).

2.2.2 Drug selection

Drugs were selected for this study on the basis of an analysis of the human pharmacokinetics of all agents reported to have anti-SARS-CoV-2 activity *in vitro*. Specifically, for all drugs considered with the exception of SOF/DCV, the human maximum plasma concentration (C_{max}) and predicted maximum lung concentration after administration of the licensed doses were used to assess whether concentrations would exceed those required to inhibit at least 50% of SARS-CoV-2 replication *in vitro* (EC_{50}). The full analysis has been published [6] and was supplemented by in-house data for 4-aminoquinolines available at MMV [7].

a) **b)**

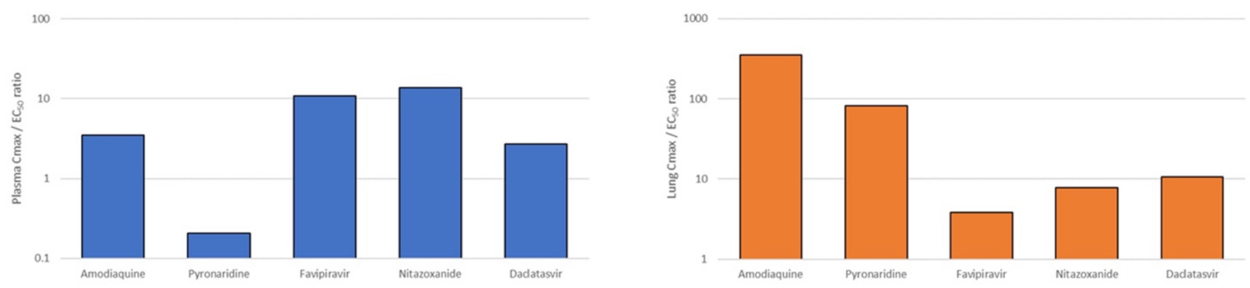

Figure 1: Anti-SARS-CoV-2 activity in plasma (a) and lung (b) for various drug candidates

These drugs, with the exception of pyronaridine, were predicted to provide concentrations with at least some antiviral activity in plasma, with favipiravir and nitazoxanide providing the greatest plasma ratios over their reported activity (Figure 1a). Similarly, based on the calculated lung concentrations, these drugs were also predicted to reach concentrations with some activity in lung, but predicted lung exposures for

amodiaquine and pyronaridine were estimated to be profoundly higher than the reported anti-SARS-CoV-2 EC_{50} (Figure 1b)

These data alone support the rationale for assessing these agents in combination for COVID-19 therapy. Additional rationale is as follows.

2.2.2.1 Artesunate-amodiaquine (Arm B) and pyronaridine-artesunate (Arm C)

Amodiaquine (AQ), desethylamodiaquine (DEAQ, primary active metabolite of AQ), and pyronaridine (PY) are active against SARS-CoV-2 (Vero76 cell assay) *in vitro* with plasma/blood EC_{50} of 1.8 µM, <0.3 µM and 0.7 µM, respectively [7]. Moreover, PBPK modelling of lung penetration indicates AQ, DEAQ and PY to be well-distributed to lungs, with a C_{max} lung/plasma ratio of 13 for AQ and 40 for DEAQ, and C_{max} lung/blood ratio of 44 for PY, supporting the selection of these compounds for this trial [7].

In contrast with AQ and PY, the available data on the activity of artesunate against SARS-CoV-2 are limited [23,24]. The inclusion of artesunate in treatment arms B and C is therefore largely driven by the availability of AQ and PY as existing fixed-dose combinations with artesunate (ASAQ and PA) with established safety, tolerability and efficacy in malaria.

2.2.2.2 Favipiravir and nitazoxanide (Arm D)

Favipiravir has already demonstrated benefits as a therapeutic agent for COVID-19 in a randomized controlled trial [10].

Nitazoxanide, is a thiazolide antiparasitic medicine used for the treatment of cryptosporidiosis and giardiasis that cause diarrhea [25,26], and also has reported activity against anaerobic bacteria, protozoa and other viruses [27]. Importantly, rapid deacetylation of nitazoxanide in blood means that the major systemic species of the drug *in vivo* is tizoxanide, which has not yet been studied for anti-SARS-CoV-2 activity. Notwithstanding, tizoxanide has been shown to exhibit similar *in vitro* inhibitory activity to nitazoxanide for rotaviruses [28], hepatitis B and C viruses [29,30], other coronaviruses, noroviruses [31] and influenza viruses [32,33]. As another respiratory virus, previous work on influenza may be useful to gain insight into the expected impact of nitazoxanide for SARS-CoV-2. Accordingly, the drug has been shown to selectively block the maturation of the influenza hemagglutinin glycoprotein at the post-translational stage [33,34] and a previous phase 2b/3 trial demonstrated a reduction in symptoms and viral shedding at a dose of 600 mg twice daily compared to placebo in patients with uncomplicated influenza [35]. Other potential benefits of nitazoxanide in COVID-19 may derive from its impact upon the innate immune response that potentiates the production of type 1 interferons [33,36] and bronchodilation of the airways through inhibition of TMEM16A ion channels [37].

No significant adverse drug-drug interactions are expected when favipiravir and nitazoxanide are administered together (https://www.covid19-druginteractions.org/).

2.2.2.3 Sofosbuvir / daclatasvir (Arm E)

The combination of sofosbuvir 400mg once daily and daclatasvir 60g once daily is normally used to treat hepatitis C and has an excellent safety profile.

SARS-CoV-2 replication and transcription depends on a number of key enzymes, notably RNA-dependent RNA-polymerase (RdRp), Main Protease (Mpro) and Helicase. The structure of SARS-CoV-2 RdRp and Mpro have been analyzed and published in high resolution and are attractive targets against which to model antiviral drugs [38,39]. Some *in silico* [40,41] and *in vitro* [42,43] studies of sofosbuvir and daclatasvir have

predicted that these and other nucleoside/nucleotide analogues will bind strongly to the SARS-CoV-2 RdRp enzyme and inhibit its function [40,43]. This is the same proposed mechanism of action of both remdesivir and favipiravir.

One study predicts sofosbuvir and remdesivir both would have equal binding energy to SARS-CoV-2 RdRp of -7.5Kcal/mol. Another study predicts molecular docking score to SARS-CoV-2 RdRp of -4.41Kcal/mol and -5.16Kcal/mol, respectively with a binding energy of -34.1Kcal/mol and -36.3Kcal/mol, respectively [44].

However, *in silico* results are not always concurrent, as computer models use different formulas and make different assumptions, and *in vitro* analyses use different cell lines and have different laboratory conditions. One deep learning model suggested that daclatasvir would have a binding strength to SARS-CoV-2 RdRp of 23.31K_d which is similar to remdesivir (20.17K_d) [45]. However, another modelling study comparing the binding strength of 88 antiviral drugs with SARS-CoV-2 Mpro, found daclatasvir to be one of the weakest binders to the enzyme (MolDock Score -45.44) [46].

<u>Pharmacokinetics</u>

Whilst the IC_{50} of remdesivir is thought to be 0.85 +/- 0.41 µM [47] the IC_{50} of sofosbuvir and daclatasvir in SARS-Cov-2 has not yet been published. One study found that sofosbuvir failed to prevent COVID-19-induced cellular death *in vitro* [48]. Sofosbuvir and daclatasvir are both licensed for use in hepatitis C virus (HCV) [48]. The IC_{50} of sofosbuvir across HCV genotypes is thought to range from 0.7 to 2.6 µM [49]. Knowledge of sofosbuvir pharmacokinetics in other viruses is crucial in guiding sufficient dosing regimens against SARS-CoV-2 and ensuring the success of repurposed antivirals. Many studies *in silico, in vitro* and in animals have shown sofosbuvir to have action against other members of the Flaviviridae and Togaviridae family (other positive-strand RNA viruses) such as yellow fever, Zika, dengue and Chikungunya (CHIKV) viruses [50,51,52,53]. Sofosbuvir inhibited yellow fever viral replication *in vitro* with an EC_{50} of around 5 µM [50]. In another study, sofosbuvir showed inhibition of three Zika virus strains in Huh-7 and Jar cells, with EC_{50} of 1 µM to 5 µM [51]. Furthermore, sofosbuvir possesses anti-dengue activity and demonstrated aggressive *in vitro* reduction of viral yield with an EC_{90} of 0.4 µM in HepG2 cells [52]. Ferreira *et. al.* [53] investigated the pharmacology of sofosbuvir on CHIKV-infected astrocytes and Huh-7 hepatoma cells *in vitro*, demonstrating that sofosbuvir inhibits CHIKV replication (EC_{50} 17 ± 5 µM and 2.7 ± 0.5 µM, respectively). Results from these studies highlight sofosbuvir's broad potential antiviral activity.

In the light of the mentioned evidence it is reasonable to believe that sofosbuvir could be effective in treating COVID-19. In Iran, there are currently eight registered randomized clinical trials looking at the effectiveness of sofosbuvir in combination with daclatasvir or ledipasvir for treatment against COVID-19, with a total sample size of 510. Results from these are expected from May 2020 onwards [54]. Six out of the eight trials are assessing the combination of sofosbuvir/daclatasvir.

2.2.3 Dose selection

Assessment of human maximum plasma concentrations and modelling of predicted maximum lung concentrations of candidate drugs was done based on the doses for which these drugs are currently licensed (with respect to their known therapeutic indications). Therefore, the safety and tolerability are well understood at these doses and they are deemed appropriate for this study.

The adult dose of nitazoxanide for diarrhea in immunocompetent patients with amebiasis, cryptosporidiosis, or giardiasis is 500 mg twice daily with food. However, for diarrhea in AIDS patients, 1000 mg twice daily with food can be used for 14 days or until diarrhea resolves. This 1000 mg twice daily

dose has been selected for this study on the basis that modelling suggests a higher proportion of patients will achieve plasma and lung exposures above the EC_{90} target at this dose [55].

2.2.4 Study design

In the absence of approved drug therapies for the treatment of COVID-19, this phase 2, exploratory trial aims to efficiently evaluate and prioritize multiple candidate therapeutics selected for clinical evaluation based on their known safety in humans, available pre-clinical activity against SARS-CoV-2, pharmacokinetic properties, and potential ease of delivery to low-resource settings.

The study offers an approach where an initial assessment of potential benefit can be made before enrolling a large number of participants in a confirmatory clinical trial with selected treatment(s). The overarching goal of the proposed study is to evaluate the effect of the proposed experimental drugs on viral shedding. Assessment of an initial virological impact will inform further evaluation in a subsequent, confirmatory, phase 3 trial to evaluate the efficacy of promising experimental drugs on clinical outcomes such as prevention of lower respiratory tract infection, duration of hospitalization, and mortality.

2.2.5 Pharmacokinetic-pharmacodynamic assessments

PK assessments in initial trials are critical to garner essential information about the exposure-response relationship for COVID-19 treatment and prevention. Given ethical concerns regarding the exposure of study personnel to highly infectious patients, intense PK sampling is not possible in the context of this study. Minimum plasma (or whole blood in the case of pyronaridine) concentrations (C_{min}) are well understood to be the best PK surrogate parameters for assessing PKPD effects in viral infections due to the speed at which viruses replicate in the presence of subtherapeutic drug concentrations. The sampling strategy in this study has therefore been developed to maximize the value of isolated samples. Minimum concentration (C_{min}) on Day 7 will be assessed in all participants in the experimental arms to coincide with the primary endpoint, and on Day 3 in participants in the ASAQ and PA arms.

2.3 Benefit/Risk Assessment

COVID-19 disease can be unpredictable in its severity. An 18.7% mortality rate has been observed among hospitalized cases in South Africa [56]. The elderly (>60 years of age) and those with medical comorbidities (e.g., cardiopulmonary disease, hypertension, diabetes mellitus and renal disease) are at highest risk of poor outcomes [11,12,13]. Transmission in younger persons, however, amplifies infection in communities, putting susceptible persons at risk. There is no proven drug for treatment of those with COVID-19 disease, although preliminary data for remdesivir is promising.

All of the planned interventions have been demonstrated to be tolerable and safe at the proposed, clinically tested doses. Participants will also be provided with standard of care treatment in accordance with local, existing guidelines, which will also serve as a control arm.

QT-interval prolongation is associated with the 4-aminoquinolone compounds. For this reason, patients at high risk of developing QT-interval prolongation (those with a history of arrhythmias or other significant cardiovascular disease, those with QT-interval prolongation at baseline, those with low serum potassium levels at baseline, and those on treatment with other drugs known to cause QT-interval prolongation) will be excluded at screening. Concomitant administration of other drugs known to cause significant QT-interval prolongation is also prohibited during the study.

Thus, the potential benefit-to-risk ratio for testing the proposed interventions as treatment, is favorable in this population.

3. OBJECTIVES AND ENDPOINTS

Objectives	Endpoints
Primary	
• To test the efficacy of each treatment regimen compared to SOC alone in reducing SARS-CoV-2 viral shedding.	• Incidence of SARS-CoV-2 clearance (defined as the proportion of participants with a negative SARS-CoV-2 RT-PCR test) on Day 7.
Secondary	
• To test the efficacy of each treatment regimen compared to SOC alone in reducing SARS-CoV-2 viral shedding determined by viral culture	• Incidence of SARS-CoV-2 clearance on Day 7 (defined as the proportion of participants with negative SARS-CoV-2 viral cultures on both Day 7 and Day 10 in the subset of participants for whom these assessments are performed and results are available)
• To test the efficacy of each treatment regimen compared to SOC alone in reducing late SARS-CoV-2 viral shedding	• Incidence of SARS-CoV-2 clearance (defined as the proportion of participants with negative SARS-CoV-2 RT-PCR tests) on Days 10, 14, 21 and 28
• To test the efficacy of each treatment compared to SOC alone in reducing the duration of SARS-CoV-2 viral shedding	• Time to clearance of SARS-CoV-2, defined as a negative SARS-CoV-2 RT-PCR test (samples collected Days 0, 3, 7, 10, 14, 21 and 28)
• To test whether each treatment regimen is associated with decreased viral shedding over 14 days compared to SOC alone	• Estimated viral load of SARS-CoV-2 detected by quantitative RT-PCR
• To test whether each treatment regimen decreases the resolution rate for symptomatic SARS-CoV-2 infection/COVID-19 disease compared to SOC alone	• Proportion of days with fever after randomization<br>• Proportion of days with respiratory symptoms after randomization<br>• FLU-PRO© Plus questionnaire scores and FLU-PRO© Plus Global Additional Daily Diary Items over the first 14 days
• To test the safety of each treatment regimen compared to SOC alone for treatment of outpatients with SARS-CoV-2 infection	• Serious adverse events (including death and hospitalization)<br>• Adverse events resulting in treatment discontinuation<br>• Adverse events considered related to the investigational products
• To test the efficacy of each treatment regimen compared to SOC alone to prevent progression to LRTI	• LRTI, defined by resting SpO2<93% sustained for 2 readings 2 hours apart AND presence of subjective dyspnea or cough
• To test whether each treatment regimen has an effect on disease severity compared to SOC alone	• Maximum score on WHO Ordinal Scale for Clinical Improvement during study participation
• To test the efficacy of each treatment regimen compared to SOC alone in reducing hospitalization	• Cumulative incidence of hospitalization at Day 28

• To test whether each treatment regimen has an effect on the duration of hospitalization among persons who become hospitalized with COVID-19 disease, compared to SOC	• Days of hospitalization
• To test the efficacy of each treatment regimen compared to SOC alone in reducing mortality.	• Cumulative incidence of mortality, measured at Day 28 or later if participant is hospitalized at the time of Day 28.
Exploratory	
• To investigate drug exposure of the study drugs in patients with COVID-19	• Plasma/whole blood C_{min} concentrations on Day 3 (Arms B and C) and Day 7 (Arms B to E)
• To explore the relationship between efficacy and plasma or whole blood concentrations of the study drugs for each treatment regimen	• Viral clearance<br>• Categorical secondary endpoints related to efficacy<br>• Plasma/whole blood C_{min} concentrations on Day 7
• To explore the immune response in symptomatic patients infected with SARS-CoV-2	• Seroconversion as assessed by means of a validated serological assay still to be determined
• To explore the relationship between sample RT-PCR results, their cycle threshold values, and corresponding viral culture results for SARS-CoV-2.	• SARS CoV-2 qualitative and quantitative RT-PCR results and cycle threshold values from all relevant time points, and viral culture results from screening, Day 7 and Day 10 in the subset of participants for whom viral culture is performed.

4. STUDY DESIGN

4.1 Overall Design

The overarching goal of this study is to assess the efficacy of interventions to reduce virus shedding among adult outpatients with mild SARS-CoV-2 infection. Confirmation of the initial virological impact on the clinical progression of COVID-19 should be obtained in a subsequent, separate, phase 3 trial focused on prevention of LRTI, and reduction of hospitalization and mortality.

This is a randomized, single center, open label, exploratory, adaptive trial. The trial will commence four experimental interventions (Arms B to E) to assess the efficacy as measured by a reduction in viral shedding compared to SOC (Arm A). Participants will be randomized to one of these treatment arms in a 1:1:1:1:1 ratio.

Initially, this study will enroll up to 250 eligible adults (18 to 65 years of age inclusive) who have RT-PCR-confirmed SARS-CoV-2 infection and self-reported symptoms of COVID-19 (50 per arm). The sample size calculations and statistical power are detailed in Section 11.1). Eligible participants will be enrolled and randomized in an equal allocation ratio to study treatment arms. The recruitment rate will be assessed on a weekly basis starting at the end of the second week after the first eligible participant is recruited and randomized. The DMC may advise increasing the sample size target, should a higher dropout rate than expected be observed. This would be based on a blind review of the pooled, observed recruitment and dropout rates, and the final sample size target, if applicable. The interim monitoring plan (written by the study Statistician) will define the strategy for potential sample size re-assessment (based on the the observed overall dropout rate) in order to preserve the pre-specified power of 80%. If this study demonstrates high rates of viral clearance for new candidate treatments, demonstration of these benefits

would then justify taking selected treatments into a subsequent, separate, phase 3, confirmatory trial with a larger sample size, powered on a new endpoint of clinical recovery.

Other unspecified additional treatment modalities may be incorporated into this protocol, based on emerging data. These will be added through protocol amendments, which will define additional sample size needs and any potential change to randomization (e.g., changing the randomization ratio, continuation/discontinuation of any of the existing arms, etc.). Details regarding sample size calculation, statistical power, and statistical methods are provided in Section 10.

Participants will be counseled about the preliminary *in vitro* data on the activity of the experimental interventions against SARS-CoV-2, and equipoise regarding efficacy in humans, given that there are only limited data available at this time. This counselling will be serially updated as new, clear data become available.

Blood samples will be drawn from participants for SARS-CoV-2 antibody testing using emerging, validated assays. Blood samples will also be collected from participants offering optional consent for storage and future testing defined by emerging disease-related data and hypotheses and suitable, validated assays. An amendment to this protocol will be drafted to describe the assessment of other outcomes based on emerging data in this rapidly developing field.

An independent DMC will be convened for this study with expertise in COVID-19 or respiratory viruses, emerging epidemics, as well as biostatistics. The purpose of the DMC is to monitor the study for social harms, safety and efficacy. The DMC will also review the blinded sample size re-assessment plan and proposed sample size changes in a blinded manner if required.

4.2 Participant and Study Completion

As an initial target, 250 participants will be randomly assigned to one of the five study treatment arms (at least 50 participants per treatment arm). This sample size target may increase based on blinded sample size re-assessment.

A participant is considered to have completed the study if he/she has completed all phases of the study including the last scheduled procedure shown in the Schedule of Activities (SoA) in Section 1.

The study will be considered complete when sufficient number of participants have completed the study to enable appropriate evaluation of the primary endpoint.

5. STUDY POPULATION

Women and men from 18 to 65 years of age, inclusive, with self-reported symptoms of COVID-19 for no more than 72 hours prior to screening informed consent **and** who test positive for SARS-CoV-2 at screening or during the two days prior to screening, will be enrolled. The eligibility criteria have been set to identify participants with mild COVID-19 disease in accordance with national guidelines [8].

Potential participants will be recruited from surrounding COVID-19 testing stations and from patients presenting to local healthcare facilities. Volunteers who are interested in participating in the trial may be consented for pre-screening to assess potential eligibility. Those eligible for screening, will be invited to participate in the informed consent process as described in Section 13.2. Written or electronic informed consent will be obtained prior to any screening procedures.

Prospective approval of protocol deviations to recruitment and enrollment criteria (also known as protocol waivers or exemptions) is not permitted.

The following eligibility criteria will be used to select study participants.

5.1 Inclusion Criteria

Participants are eligible to be included in the study if all of the following criteria apply:

1. Age from 18 to 65 years of age, inclusive, at the time of signing the informed consent.
2. Willing and able to provide informed consent.
3. Women of reproductive potential must be using a highly effective method of contraception for at least 28 days prior to enrolment and must be able and willing to continue its use throughout the duration of the study.
4. Men must agree to use condoms when engaging in heterosexual sex during the study and for the period up to 91 days after the last dose of study medication. Men who are not randomized to a treatment arm including favipiravir (or another arm identified as having teratogenic potential through semen) will no longer need to adhere to this after randomization.
5. Laboratory confirmed SARS-CoV-2 infection, and any of the following self-reported symptoms with onset no more than 72 hours prior to screening informed consent, and still present at randomization: fever or chills, cough, myalgia, sore throat, headache, conjunctivitis, shortness of breath, nausea, diarrhea, new onset of anosmia or ageusia, or other symptoms recognized in local and international guidelines as typical of mild COVID-19.
6. Body weight ≥45 kg.
7. Access to reliable video conference, telephone, direct/text messaging, or other device permitting real-time, reliable information transfer.

5.2 Exclusion Criteria

Participants are excluded from the study if any of the following criteria are met:

1. Pregnant or lactating women.
2. Known hypersensitivity or specific contraindications to the use of any of the active drugs in the treatment arms, or similar compounds.
3. Duration of self-reported symptoms of COVID-19 for more than 96 hours or four calendar days (whichever is greater) prior to randomisation.
4. Signs of respiratory distress prior to randomization, including:
   - respiratory rate >24 breaths/min
   - SpO_2 <95% in room air.
5. Resting pulse rate ≥120 beats/min.
6. High likelihood of hospitalization in the opinion of the attending clinician.
7. QTcF >470 msec for females, or >450 msec for males, at screening.
8. Serum potassium <3.5 mmol/L at screening.
9. History of clinically significant cardiovascular disease (including arrhythmias, QT-interval prolongation, torsades de pointes (TdP), history of coronary artery disease with graft or stent procedures/surgery, cardiac failure [class 2 or higher using the New York Heart Association functional classification]).
10. Known chronic kidney disease (Stage IV or receiving dialysis).
11. Known cirrhosis (Child-Pugh Class B or greater).
12. Known macular degeneration, or other known retinal diseases, or 4-aminoquinolone-induced visual impairment.

13. Currently receiving, or recently received (within 60 days prior to randomization) treatment with any of the drugs in the experimental treatment arms (Arms B to E).
14. Currently receiving, or recently received (within 30 days prior to randomization) treatment with any antimalarial drugs.
15. Currently on treatment with drugs with known arrhythmogenic potential, or those known to induce significant QT-interval prolongation or TdP, as detailed in Appendix 6.
16. Currently on treatment for tuberculosis (or on treatment with rifampicin for any other indication), or on treatment with a protease inhibitor-based antiretroviral regimen, or efavirenz, or carbamazepine.
17. Inability/unlikely to be in the study area for the duration of the 28 day follow-up period.
18. Any surgical or medical condition which might significantly alter the absorption, distribution, metabolism, or excretion of drugs, or which may jeopardize the safety of the volunteer or the objectives of the study. The Investigator should make this determination in consideration of the volunteer's medical history.
19. Personnel (e.g. investigator, sub-investigator, research assistant, pharmacist, study coordinator or anyone mentioned in the delegation log) directly involved in the conduct of the study.
20. Participant is judged by the Investigator to be at significant risk of failing to comply with the provisions of the protocol as to cause harm to self or seriously interfere with the validity of the study results.

Volunteers may be re-screened if they are found not to have COVID-19 at the time of the initial screening, but develop a further, and separate acute illness suggestive of COVID-19 at a later stage again.

5.3 Co-enrollment Guidelines

Participants may be co-enrolled in other research studies, provided that these are observational studies where no interventions are offered only. Any other exception requires approval of the Principal Investigator; if a participant clinically worsens, such as requiring hospitalization, such an exception will be automatically granted for participation in other offered treatment studies. The study team should be consulted for co-enrollment in studies that do not meet this guidance or if there are questions about eligibility for co-enrollment. For any co-enrolled study, the total volume of blood samples collected across the two studies should not exceed 480 mL over any eight week period.

6. TREATMENTS

Study treatment is defined as any investigational treatment or marketed product to be administered to a study participant according to the study protocol.

6.1 Treatments Administered

The following treatments are planned:

Arm A:

Study Treatment Name	Paracetamol (SOC)
Dosage Formulation	Tablet 500 mg
Route of Administration	Oral
Dosing Instructions	2 tablets (1000 mg) to be taken 6-hourly as needed

Arm B:

SOC as described in Arm A plus experimental treatment as follows:

Study Treatment Name	Artesunate-amodiaquine (ASAQ)
Dosage Formulation	100/270 mg artesunate/amodiaquine fixed dose combination, uncoated tablets
Route of Administration	Oral
Dosing Instructions	2 tablets (200/540 mg artesunate/amodiaquine) daily for 3 days

Arm C:

SOC as described in Arm A plus experimental treatment as follows:

Study Treatment Name	Pyronaridine-artesunate (PA)	
Dosage Formulation	180/60 mg pyronaridine tetraphosphate/artesunate fixed dose combination, film-coated tablets	
Route of Administration	Oral	
Dosing Instructions	For participants with body weight 45 to <65 kg:	3 tablets (540/180 mg pyronaridine/artesunate) daily for 3 days
	For participants with body weight ≥65 kg	4 tablets (720/240 mg pyronaridine/artesunate) daily for 3 days

Arm D:

SOC as described in Arm A plus experimental treatment as follows:

Study Treatment Name	Drug 1: Favipiravir	Drug 2: Nitazoxanide
Dosage Formulation	200 mg and 400 mg film-coated tablets	500 mg tablets
Route of Administration	Oral	Oral
Dosing Instructions	1600 mg 12-hourly for 1 day,<br>then<br>600 mg 12-hourly for 6 days	2 tablets (1000 mg) 12-hourly for 7 days;<br>to be taken with food.

Arm E:

SOC as described in Arm A plus experimental treatment as follows:

Study Treatment Name	Sofosbuvir/daclatasvir
Dosage Formulation	400mg/60 mg sofosbuvir/daclatasvir fixed dose combination, film coated tablets
Route of Administration	Oral
Dosing Instructions	1 tablet (400 mg/60 mg sofosbuvir/daclatasvir) daily for 7 days

6.2 Risks to the Participants

Administration of IMP in this study will be for a short duration (up to 7 days) at doses that are well-described and have been shown to be well-tolerated in clinical studies. Risks to participants are therefore expected to be well-controlled.

Since both COVID-19 and many drugs are known to be associated with liver injury, all participants will be advised of the clinical signs and symptoms of hepatotoxicity and will be requested to report the emergence of any of these urgently.

In evaluating individual risks to participants, the relevant Summaries of Product Characteristics (SmPCs) for each IMP should be reviewed. Documented risks related to different components of the treatment arms include, but are not limited to, the following.

Artesunate-amodiaquine:

Amodiaquine has been shown to prolong the QT-interval in some patients.

Pyronaridine-artesunate:

PA has been associated with a transient increase in transaminases in a small number of patients treated for acute malaria and in Caucasian, healthy volunteers. No associated clinical signs and symptoms of hepatotoxicity have been observed or reported.

Favipiravir:

Early embryonic deaths and teratogenicity have been observed in pre-clinical studies with favipiravir. Participants will be counselled with regard to this prior to study participation and will be required to agree to use of suitable contraception (Section 5.1). Women of reproductive potential will require a negative urine pregnancy test to be eligible for study participation.

Nitazoxanide:

No specific risks to participants are predicted at the proposed dose to be administered during this study.

Sofosbuvir:

Cases of severe bradycardia and heart block have been observed when sofosbuvir-containing regimens are used in combination with amiodarone, which is an exclusion criterion for participation in this study.

Daclatasvir:

Cases of severe bradycardia and heart block have been observed when daclatasvir-containing regimens are used in combination with amiodarone, which is an exclusion criterion for participation in this study. Diabetics may experience improved glucose control, potentially resulting in symptomatic hypoglycemia, after initiating treatment.

6.2.1 Management of participants to limit risks of SARS-CoV-2 transmission

To limit the transmission of SARS-CoV-2, the following measures will be employed:

- Participants will engage in visits and site personnel contact using telemedicine, telephone or text/direct messaging as far as is possible.
- For any necessary physical interactions, study and laboratory personnel will be instructed to wear personal protection equipment (PPE) at all times, and participants and laboratory/study personnel will be instructed to adhere to national and regional guidance for COVID-19-related safety measures.
- The organization of each study site will be in accordance with national and regional guidelines for limiting the spread of COVID-19, and will be such that contact between persons with potential SARS-CoV-2 infection and other patients is restricted.

- To limit exposure in waiting rooms and pharmacies, daily vital signs will be self-monitored by participants and medications will be delivered to participants at homes where possible.
- To limit community exposure to trial participants infected with SARS-CoV-2, participants will be required to self-quarantine in accordance with national and regional guidelines for the required duration and transported to study site with a study vehicle in line with national infection control guidelines.

6.3 Dose Modification and Toxicity Management

If any study therapy dose is missed, it should be taken as soon as possible. If it is less than 4 hours before the next dose is scheduled, the missed dose should be skipped and documented as such in the participant daily survey.

Modification for toxicities is discussed below. Only toxicities related to study medications provided through the study will be considered in this section.

Grade 1 or 2

Participants who develop Grade 1 or 2 toxicity (per Division of Acquired Immunodeficiency Syndrome [DAIDS] Table for Grading the Severity of Adult and Pediatric Adverse Events Corrected Version 2.1, July 2017 [https://rsc.niaid.nih.gov/sites/default/files/daidsgradingcorrectedv21.pdf]) that is considered by the site Investigator to be related to the study medication, may continue study treatment at the discretion of the site Investigator with close follow up. If a participant chooses to discontinue study treatment, the site should notify the Safety Review Team within 7 days. These participants will remain on study, off study treatment, and have all evaluations performed.

Grade 3

- Participants who develop a Grade 3 toxicity thought by the site Investigator to be related to study drug should have study product withheld, and the site should consult with the Safety Review Team via the Medical Monitor. The participant should be re-evaluated every 2 days until the AE returns to Grade ≤2, at which time study drug may be reintroduced at the discretion of the site Investigator in consultation with the Safety Review Team.
- Participants experiencing a Grade 3 toxicity requiring permanent discontinuation of study product should be followed up weekly until resolution of the toxicity. These participants will remain on study, off study treatment, and have all evaluations performed per the SoA.

Grade 4

- Participants who develop a Grade 4 toxicity will have study product permanently discontinued, and the site should notify the Principal Investigator and the Safety Review Team via the Medical Monitor within 72 hours.
- Participants experiencing Grade 4 toxicity requiring permanent discontinuation of study product should be followed up weekly until resolution of the AE or return to baseline. These participants will remain on study, off study treatment, and have all evaluations performed per the SoA.

Specific Management of Toxicities Related to Study Drugs

All possible drug toxicities will be managed on a case-by-case basis. Grade 2 and above toxicities will be discussed with one of the study physicians in their role as a site Investigator. All Grade 3 and above

toxicities will be reported to the Principal Investigator and Medical Monitor. Case management and follow-up will, at a minimum, be in accordance with the above-mentioned guidelines.

All AEs (whether or not considered drug-related) will be reported during the study, and all serious adverse events (SAEs) and suspected, unexpected, serious adverse reactions (SUSARs) will be reported in accordance with applicable regulations and requirements (Section 9.2.2).

6.4 Method of Treatment Assignment

Participants will be randomized using a centralized, automated randomization system (IVRS/IWRS) to one of the five treatment arms (Arms A to E) in a 1:1:1:1:1 ratio. The randomization plan will be adaptive based on treatment arms available for participant allocation and will be overseen by the study Statistician.

6.5 Blinding

No blinding will be performed to conceal treatment identification for the study. The study will be open-label to participants and study personnel.

6.6 Preparation / Handling / Storage / Accountability

Drugs should be stored at room temperature, as per package insert. Records must be maintained that document receipt, release for dosing, disposal, or return to the Sponsor.

Details of all drug-related procedures surrounding shipping, receipt, storage, handling, preparation, dispensing and accountability will be documented in the Pharmacy Manual.

6.7 Treatment Compliance

Study drug (and home monitoring kits) will either be dispensed to participants at the site or delivered to participants at their homes. In cases where study supplies are delivered, participants will be contacted to ensure that they received the box of study supplies and took their first dose of medication. All participants will be asked to complete a daily survey to gather information regarding treatment administration. Medication errors, including under-dosing, will be recorded in the survey.

Consultation via tele-medicine, text/direct messaging, or telephone will be available to provide support to the participant to complete study procedures.

6.8 Concomitant Therapy

Participants will be asked about concomitant medications at the screening/baseline evaluation visit. During the study, participants will be asked to complete a daily survey that includes information regarding any medication or vaccine (including over-the-counter or prescription medicines, vitamins, and/or herbal supplements) that the participant receives during the study. At each contact, the Investigator should question the participant about any medication taken.

6.8.1 Prohibited medications

Participants will be advised to consult the study clinician before commencing any new concomitant medications during the study.

The use of the following drugs is prohibited during the study (Appendix 6):

- Drugs with known arrhythmogenic potential, or known to induce significant QT-interval prolongation or TdP (detailed in Appendix 6)
- Protease inhibitor-based antiretroviral regimens, or efavirenz
- Rifampicin
- Carbamazepine.

6.8.2 Precautionary medications

Use of medications classified as precautionary is not study prohibitory but will be discussed with the study clinician and the participant. The study clinician will review all previous and concomitant medications taken by study participants to evaluate possible drug-drug interactions and ensure that unacceptable risks do not result from these. The relevant SmPCs for the applicable study drugs and previous/concomitant medications will be used as a reference.

Known interactions to be considered include, but are not limited to, the following:

Artesunate-amodiaquine:

Drugs known to prolong the QT interval should be used with caution. In the absence of clinical data, ASAQ is not recommended to be administered concomitantly with drugs known to inhibit CYP2A6 (e.g., methoxsalen, pilocarpine, tranylcypromine) and/or CYP2C8 (e.g., trimethoprim, ketoconazole, ritonavir, saquinavir, lopinavir, gemfibrozil, montelukast).

Pyronaridine-artesunate:

Caution is advised when co-administering PA with metoprolol given in cardiac failure. A possible dose adjustment of metoprolol may be required. Dihydroartemisinin administration may result in a slight decrease in CYP1A2 activity. Caution is therefore, advised when PA is administered concomitantly with medicinal products metabolized by this enzyme that have a narrow therapeutic index, such as theophylline.

Favipiravir:

Favipiravir should be used with caution when administered together with pyrazinamide, repaglinide, theophylline, and famciclovir.

Nitazoxanide:

Caution should be used when administering nitazoxanide concurrently with other highly plasma protein-bound drugs with narrow therapeutic indices.

Sofosbuvir:

Medicinal products that are strong (e.g., carbamazepine, phenobarbital, phenytoin, rifampicin and St John's wort) or moderate (e.g., modafinil, oxcarbazepine and rifapentine) P-glycoprotein (P-gp) inducers in the intestine may decrease sofosbuvir plasma concentration leading to reduced therapeutic effect. Co-administration with such medicinal products is not recommended. The concomitant administration of protease inhibitors can result in increased plasma concentrations of sofosbuvir and is not recommended.

Daclatasvir:

Strong inducers of CYP3A4 and P-gp (e.g., phenytoin, carbamazepine, oxcarbazepine, phenobarbital, rifampicin, rifabutin, rifapentine, systemic dexamethasone, and St John's wort) may lead to lower exposure and loss of efficacy of daclatasvir. The concomitant administration of protease inhibitors can result in increased plasma concentrations of daclatasvir and is not recommended.

Daclatasvir is an inhibitor of P-gp, organic anion transporting polypeptide (OATP) 1B1, OCT1 and breast cancer resistance protein (BCRP). Administration of daclatasvir may increase systemic exposure to medicinal products that are substrates of P-gp, OATP 1B1, OCT1 or BCRP, which could increase or prolong their therapeutic effect and adverse reactions. Caution should be used if the medicinal product has a narrow therapeutic range.

6.9 Treatment After the End of the Study

No additional treatment will be provided at the end of the study.

7. DISCONTINUATION/WITHDRAWAL CRITERIA

7.1 Discontinuation of Study Treatment

Study treatment will be discontinued for the following reasons:

- Hospitalization (at the discretion of the inpatient provider, hospitalized participants may continue to receive study treatment if maintained on the originally randomized treatment regimen)
- Requirement for prohibited concomitant medications or other contraindication to study product
- Occurrence of an AE requiring discontinuation of study product
- Request by participant to terminate study treatment
- Clinical reasons believed by the physician to be life-threatening or place the participant at undue risk, even if not addressed in Section 6.3.

Participants who stop study product should continue study participation off study product with continued evaluations as per the SoA. The reason for study product discontinuation should be recorded.

Hospitalized participants will be followed through hospitalization and the last visit will be within 3 days of discharge if this occurs after Day 28.

7.2 Withdrawal from the Study

- A participant may withdraw from the study at any time at his/her own request or may be withdrawn at any time for the following reasons:
    - At the request of the primary care provider if he/she thinks the study is no longer in the best interest of the participant
    - Participant is judged by the Investigator to be at significant risk of failing to comply with the provisions of the protocol as to cause harm to self or seriously interfere with the validity of the study results
    - At the discretion of the Institutional Review Board/Ethics Committee or government agencies as part of their duties, Investigator, or industry supporter.

- If the participant withdraws consent for disclosure of future information, the Sponsor may retain and continue to use any data collected before such withdrawal of consent.
- If a participant withdraws from the study, he/she may request destruction of any samples taken and not tested. This must be documented by the Investigator in the site study records.
- If possible and agreed to by the participant, assessments detailed for the EOS visit on Day 28 (SoA) should be completed at the time of study discontinuation if a participant withdraws from the study.

7.3 Lost to Follow-up

A participant will be considered lost to follow-up if he/she is unable to be contacted by the study site.

The following actions must be taken if a participant fails to comply with required study procedures:

- The site must attempt to contact the participant as soon as possible and counsel the participant on the importance of maintaining the assigned procedure schedule and ascertain whether or not the participant wishes to and/or should continue in the study.
- Before a participant is deemed lost to follow-up, the Investigator or designee must make every effort to regain contact with the participant (where possible, 3 telephone calls and, if necessary, a visit to the participant's place of residence by a member of the study team). These contact attempts should be documented in the participant's medical record.
- Should the participant continue to be unreachable, he/she will be considered to have withdrawn from the study with a primary reason of lost to follow-up.

8. STUDY ENCOUNTERS

The current COVID-19 pandemic has placed a significant burden on the healthcare system. Contact between study participants and study personnel will be limited as far as is possible. Study personnel will be instructed to wear PPE during all interactions, and participant and study personnel will be instructed to adhere to national and regional guidance for COVID-19-related safety measures.

For this study, data collection will be self-conducted where possible to minimize the impact of non-ill participants within the healthcare system and the risks of SARS-CoV-2 transmission. If the participant is assessed as eligible during the screening visit, contact between study participants and study personnel will occur via tele-medicine, text/direct messaging or telephone as far as is possible.

Participants will be instructed to seek clinical care should they manifest any signs or symptoms of LRTI requiring medical intervention and notify their physician about trial participation. Any participant who's clinical condition deteriorates during the study, will be hospitalised if required in accordance with the national guidelines for clinical management of patients with COVID-19 [8].

8.1 Screening Evaluation: Day 0 (-1 day window)

Presenting patients will be assessed for study eligibility through screening conducted at the site.

Day 0 evaluations and procedures are as follows:

- Screening informed consent for all eligibility assessments to be conducted on Day 0 (may be taken electronically)
- Collection of demographic information

- Collection of information on the duration of COVID-19 symptoms
- Pregnancy and lactation status, and a urine pregnancy test for women of reproductive potential (women will be considered to be of reproductive potential unless they have been surgically sterilized [by hysterectomy and/or bilateral oophorectomy and/or bilateral salpingectomy or tubal ligation], or are post-menopausal [age-appropriate spontaneous amenorrhea for ≥12 months in the absence of oral contractive use for >12 months)
- Collection of a mid-nasal swab and saliva sample (or other sample depending on best practice recommendations) for RT-PCR detection and quantification of SARS-CoV-2 and viral culture
- Collection of blood sample for serum potassium concentration
- Dispensing of a 48-hour "flu pack" for symptomatic management of COVID-19 symptoms (paracetamol 1 g 6-hourly to be taken orally as needed and other optional symptomatic therapies [e.g., throat lozenges] based on availability and need).

Once available, participants will be contacted to inform them of their test results, and those with a positive SARS-CoV-2 RT-PCR test result, a serum potassium ≥ 3.5 mmol/L, and otherwise eligible in terms of assessments conducted on Day 0, will be invited to return for an enrollment visit on Day 1.

8.2 Enrollment/Randomization/Start of Treatment: Day 1

Patients who present for the Day 1 visit will be invited to take part in the study (pending additional confirmation of eligibility), and to provide informed consent for this and the remaining eligibility assessments in accordance with the main study informed consent form (ICF) prior to any further procedures being conducted. The main study informed consent may be taken electronically.

Eligibility will continue to be assessed at the Enrollment Visit on Day 1 in accordance with the following assessments:

- Collection of past and current medical conditions
- Collection of concomitant medication information, including contraception usage
- Weight and height assessment
- Abbreviated, symptom-directed, physical examination to include as least the following:
    - General examination
    - Heart and lungs
- Vital signs (temperature, pulse, and respiratory rate)
- SpO2 assessment
- ECG, including assessment of QTcF-interval
- Check of inclusion and exclusion criteria.

Thereafter, the following will be conducted for patients meeting all eligibility criteria:

- Participants will be randomized to one of the treatment arms in accordance with the study randomization schedule.
- Each participant will receive a monitoring kit containing a thermometer and an SpO2 monitor.

- Participants will be instructed as to how to:
    - Measure daily vital signs (temperature, pulse and respiratory rate)
    - Measure daily SpO2 (and repeat SpO2 if the scheduled reading is <93%)
    - Complete the FLU-PRO© Plus questionnaire
    - Complete the daily survey (comprising the FLU-PRO© Plus Global Additional Daily Diary Items and other general daily diary questions).
- A mobile data collection application will be installed on the participant's mobile device (for participants that have their own, compatible, mobile device).
- A blood sample will be collected for:
    - SARS-CoV-2 serology
    - Storage for future testing (only in participants providing optional consent for this).
- Participants will perform the following assessments under observation of the site personnel (and record data using telemedicine or relay this to the site personnel for transcribing):
    - Vital signs (temperature, pulse and respiratory rate)
    - SpO2 measurement
    - The FLU-PRO© Plus questionnaire
    - The daily survey (comprising the FLU-PRO© Plus Global Additional Daily Diary Items and other general daily diary questions).
- A study team member will complete the WHO Ordinal Scale for Clinical Improvement survey.
- IMP for the full course of treatment will be dispensed to participants (either at the study site, or via courier or study personnel delivery). Participants will be instructed to commence their study treatment on the same day.

Prior to completion of the visit, the following will be done:

- Participants will be instructed to seek clinical care at any stage throughout the study should they develop signs or symptoms of respiratory distress. If needed, direct hospital referral may be facilitated by the site personnel in accordance with the national guidelines for clinical management of patients with COVID-19 [8].
- Participants will be advised that, at any stage during the study, they may request additional contact, including via telemedicine or telephone, with the study clinician or staff to discuss developing symptoms or AEs, or if they need to clarify study procedures.

Screening, enrollment and randomization procedures (Day 0 and Day 1) can take place on the same calendar day in the unlikely event that the RT-PCR result is available rapidly.

8.3 Treatment Continuation and Follow-Up: Day 2 to Day 14 (±2)

A study team member will be available via telemedicine, text/direct messaging, or telephone to provide support for completion of study procedures.

On Relevant Study Treatment Days:

- Participants will take their study treatment until completion as per the dosing schedule described in Section 6.1.

On Days 2 to 14 (±2) inclusive:

- Participants will complete the following assessments (and record data using telemedicine, telephonically or via text/direct messaging):
  - Vital signs (temperature, pulse and respiratory rate)
  - SpO2 measurement
  - The FLU-PRO© Plus questionnaire
  - The daily survey (comprising the FLU-PRO© Plus Global Additional Daily Diary Items and other general daily diary questions).

On Day 2:

- Contact with the study clinician or staff will be conducted via telemedicine or telephone. Participants will be clinically assessed for signs and symptoms of respiratory distress and will have in person assessment of AEs.

On Days 3 (±1), 7 (±1), 10 (±1), and 14 (±2):

- Contact with the study clinician or staff will be conducted via telemedicine or telephone, or may be in person if the relevant quarantine period has been completed in accordance with local guidelines. Participants will be clinically assessed for signs and symptoms of respiratory distress and will have in person assessment of AEs.
- Participants will be visited by a study team member, laboratory personnel or a CHW, or may report to the site if the relevant quarantine period has been completed, for collection of a mid-nasal swab and saliva sample (or other sample depending on best practice recommendations) for RT-PCR detection and quantification of SARS-CoV-2 (Days 3, 7, 10 and 14) and viral culture (Days 7 and 10).

On Days 3 and 7:

- Participants in the ASAQ and PA treatment arms will have a plasma and/or whole blood PK sample collected on Day 3 prior to the last dose of IMP and approximately 24 hours after the previous IMP dose.
- Participants in the experimental treatment arms (Arms B to E) will have a single plasma and/or whole blood PK sample collected on Day 7 prior to their last dose of IMP (if relevant) and relative to the previous dose of IMP administered as follows:
  - 24 (±2) hours post-dose for SOF/DCV
  - 96 (±2) hours post dose for ASAQ, PA
  - 12 (±1) hours post dose for FPV-NTZ.

On Days 7 (±1) and 14 (±2):

- A study team member will complete the WHO Ordinal Scale for Clinical Improvement survey.

8.4 Late Follow-Up: Day 21 (±2)

The following will be performed:

- Contact with the study clinician or staff will be conducted via telemedicine or telephone. Participants will be clinically assessed for signs and symptoms of respiratory distress and will have in person assessment of AEs. As needed, additional contact with the study clinician or staff will be conducted at the request of the participant (e.g., if developing concerning symptoms or an AE) or if needed to clarify study procedures or follow-up symptoms.
- Participants will complete the following assessments (and record data using telemedicine, telephonically or via text messaging):
  - Vital signs (temperature, pulse and respiratory rate)
  - The FLU-PRO© Plus questionnaire
  - The daily survey (comprising the FLU-PRO© Plus Global Additional Daily Diary Items and other general daily diary questions).
- Participants will report to the site, or be visited by a study team member, laboratory personnel or a CHW for collection of a mid-nasal swab and saliva sample (or other sample depending on best practice recommendations) for RT-PCR detection of SARS-CoV-2.
- A study team member will complete the WHO Ordinal Scale for Clinical Improvement survey.

A study team member will be available via telemedicine, text/direct messaging, or telephone to provide support for completion of study procedures.

8.5 End-of-Study: Day 28

The following will be performed:

- Contact with the study clinician or staff will be conducted via telemedicine or telephone or in person. Participants will be clinically assessed for signs and symptoms of respiratory distress and will have in person assessment of AEs.
- Participants will complete the following assessments (and record data using telemedicine, telephonically, via text messaging or in person):
  - Vital signs (temperature, pulse and respiratory rate); these may be assessed by the site personnel if an in person visit is conducted
  - The FLU-PRO© Plus questionnaire
  - The daily survey (comprising the FLU-PRO© Plus Global Additional Daily Diary Items and other general daily diary questions).
- Participants will report to the site or be visited by a study team member or CHW for the following:
  - Collection of a mid-nasal swab and saliva sample (or other sample depending on best practice recommendations) for RT-PCR detection of SARS-CoV-2
  - Collection of blood for SARS-CoV-2 serology
  - Collection of blood for storage for future testing (only in participants providing optional consent for this).

- A study team member will complete the WHO Ordinal Scale for Clinical Improvement survey.
- Unused IMP, empty IMP containers and SpO2 monitors will either be returned to the site by the participant, or collected by courier or a member of the study team (if not already returned at an earlier time point).

For participants who are hospitalized on Day 28, the Day 28 procedures will be performed within 3 days after discharge from the hospital.

A study team member will be available via telemedicine, text/direct messaging, or telephone to provide support for completion of study procedures.

Clinical outcomes will be confirmed through appropriate health records, if possible.

Participant reimbursement may be performed in accordance with local standards.

9. STUDY ASSESSMENTS AND PROCEDURES

- Study procedures and their timing are summarized in the SoA.
- Protocol waivers or exemptions are not allowed.
- Immediate safety concerns should be discussed with the Sponsor (via the Medical Monitor or chairperson of the SRT) immediately upon occurrence or awareness to determine if the participant should continue or discontinue study treatment.
- All screening and enrollment evaluations (demographic data, duration of COVID-19 symptoms, pregnancy and lactation status, mid-nasal swab and saliva sample, confirmation of positive SARS-CoV-2 result, serum potassium, medical history, previous and concomitant medication usage, weight and height, abbreviated, symptom-directed physical examination, study personnal assessment of vital signs and SpO2, and ECG and QTcF evaluation) must be completed prior to randomization, and reviewed to confirm that potential participants meet all eligibility criteria. The Investigator will maintain a screening log to record details of all participants screened and to confirm eligibility or record reasons for screening failure, as applicable.
- Procedures conducted as part of the participant's routine clinical management and obtained before signing of the informed consent form (ICF) may be utilized for screening or baseline purposes provided that the procedures meet the protocol-specified criteria and were performed within the time frame defined in the SoA.
- Blood samples will only be collected for SARS-CoV-2 serological assays (in accordance with emerging, suitable and validated assays), for a screening serum potassium assessment, for a maximum of two PK samples, and for storage for future testing (in participants offering optional consent for this only). The maximum amount of blood collected from any participant over the duration of the study is expected to be 71 mL but should not exceed 480 mL even if additional safety assessments are required.

9.1 Efficacy Assessments

9.1.1 Mid-nasal swab and saliva specimen

Mid-nasal swabs and saliva specimens (or other site depending on best practice recommendations and the availability of suitable, validated assays) will be collected from all potentially eligible volunteers at

screening on Day 0 for viral detection (and quantification and viral culture if the participant is subsequently enrolled). Additional mid-nasal swabs and saliva samples will be collected on Days 3, 7, 10, 14, 21 and 28. Participants will report to the site or be visited by a study team member, laboratory personnel or a CHW to facilitate swab collection.

Swab kits may contain either viral transport media, 0.9% saline, or phosphate-buffered saline, which have been shown to be equivalent for storage of SARS-CoV-2 samples based on validated assays [57]. Saliva samples will be collected in suitable sterile containers. Previous testing has demonstrated that respiratory viral ribonucleic acid (RNA) is stable in room temperature for up to 1 week.

Swabs will be subjected to RNA amplification and tested for SARS-CoV-2. Qualitative assays will be performed at all time points. Additional quantitative assays for viral load will be performed at screening and on Days 3, 7, 10 and 14. Viral culture will be performed on samples from enrolled participants collected at screening, Day 7 and Day 10.

Further details regarding mid-nasal swab and saliva sample (or other biological samples depending on the availability of suitable, validated assays) collection, handling and processing, as well as the testing laboratory, assays and methods to be used, will be documented in the Laboratory Manual.

9.1.2 Participant surveys

Participants will be asked to complete the the following surveys during the study:

- The FLU-PRO© Plus questionnaire [58,59,60] to assess the symptomatic extent of their illness (Appendix 3)
- The daily survey (Appendix 4) that includes:
  - The FLU-PRO© Plus Global Additional Daily Diary Items (related to general well-being, overall severity of symptoms, and ability to perform usual daily activies)
  - General daily diary items (related to study drug administration, recording of vital signs and SpO2 measurements, review of concomitant medications, and information related to possible adverse events).

These surveys will be completed electronically using telemedicine, or telephonically during an interview with a study team member.

9.1.3 WHO Ordinal Scale for Clinical Improvement

A study team member will complete the WHO Ordinal Scale for Clinical Improvement (Appendix 5) after contact (via telemedicine or telephone) on relevant study days.

9.2 Adverse Events

Participants will be asked to complete the daily survey (Appendix 4) that includes information on any symptoms that they are experiencing. In addition, AE review by a staff member (via telemedicine, telephone, or text/direct messaging) will be performed.

All AEs must be recorded on the electronic case report forms (eCRFs).

AEs will be recorded from the time of informed consent until the end-of-study visit (Day 28). SAEs occurring after the reporting period that the Investigator becomes aware of must be reported as described in Section 9.2.2 if the Investigator considers the event to be related to the IMP administered.

9.2.1 Definition

An AE is any untoward medical occurrence that occurs in a participant or clinical investigation participant administered a pharmaceutical product, which does not necessarily have a causal relationship with this treatment. An AE can therefore be any unfavorable and unintended sign (including abnormal laboratory findings), symptom, or disease temporally associated with the use of an investigational product, whether or not considered related to the product.

AEs may include the onset of new illnesses and the exacerbation of pre-existing medical conditions. An AE can include an undesirable medical condition occurring at any time, including baseline or washout periods, even if no study treatment has been administered.

9.2.2 Serious adverse events

An SAE is defined as any untoward medical occurrence that:

- Results in death
- Is life-threatening (immediate risk of death)
- Requires inpatient hospitalization or prolongation of existing hospitalization (with the exception of hospitalization due to progressive COVID-19 disease)
- Results in persistent or significant disability/incapacity
- Results in a congenital anomaly/birth defect
- Is an important medical event that may not be immediately life-threatening or result in death or hospitalization but may jeopardize the participant or may require intervention to prevent one of the other outcomes listed in the definition above.

All AEs that are recorded must have their severity graded. To grade AEs, sites should refer to the DAIDS AE Grading Table, corrected Version 2.1, July 2017, which can be found on the DAIDS Regulatory Support Center website at https://rsc.niaid.nih.gov/sites/default/files/daidsgradingcorrectedv21.pdf.

The Investigator will report all SAEs to the Sponsor through the Qualified Person for Pharmacovigilance (QPPV) within 24 hours of the site personnel becoming aware of the event. The report should be in writing by email or fax with the Medical Monitor on copy and documented on a standard SAE Reporting Form. In addition to this, fatal or life-threatening SAEs must be reported immediately to the QPPV, irrespective of the extent of available AE information.

Qualified Person Responsible for Pharmacovigilance [redacted]
Email:
Tel/Fax:

For all SAEs, the Investigator is obligated to pursue and provide information in accordance with the timeframes for reporting specified above. In addition, an Investigator may be requested to obtain specific additional follow-up information in an expedited fashion. This information may be more detailed than that captured on the AE case report form. In general, this will include a description of the AE in sufficient detail to allow for a complete medical assessment of the case and independent determination of possible causality. Information on other possible causes of the event, such as concomitant medications and illnesses must be provided. In the case of a participant death, a summary of autopsy findings (if available) must be submitted as soon as possible to the QPPV.

The Local Ethics Committee and Regulatory Authority will be notified of all SAEs in accordance with their requirements.

Reporting and follow-up procedures are described in the Safety Management Plan.

9.2.3 Treatment-related AEs and SAEs

A treatment-related AE is defined as any new AE that begins, or any preexisting condition that worsens in severity, after at least 1 dose of study treatment has been administered and is considered by the Investigator to be related to the study medication.

All AEs and SAEs should have attribution recorded as treatment- or not treatment-related, in the judgment of the site Investigator.

9.3 Treatment of Overdose

Overdose of any of the study interventions (including SOC) should be managed according to the product labeling information. Study drug overdose, including misuse or abuse of the product and medication errors, should be reported in the eCRF in the clinician notes.

9.4 Pregnancy

All women of reproductive potential will be offered effective contraception for the duration of the study. While all efforts will be undertaken to prevent pregnancy among participants, or pregnancy in partners of male participants in Arm D, should a participant or partner of a participant from any study arm become pregnant during the study, she will be counselled (or invited for counselling if she is a participant's partner) regarding the risks associated with known or potentially teratogenic study drugs, and undergo an informed consent process to confirm her continued participation in the study (if appropriate), and willingness to be followed through the pregnancy.

Pregnancies occurring in participants enrolled in this study, or their partners, must be reported and followed to outcome. Reporting should be performed using a standard Pregnancy Reporting Form. The reporting procedure follows that described for SAEs in Section 9.2.2.

Pregnancy alone is not regarded as an AE unless there is a possibility that the study drug may have interfered with the effectiveness of a contraceptive medication. Elective abortions without complications should not be considered AEs unless they were therapeutic abortions. Hospitalization for normal delivery of a healthy newborn should not be considered an SAE. Pregnancy is not considered an SAE unless there is an associated outcome that meets one or more of the SAE criteria. Spontaneous abortions should always be reported as SAEs.

Any pregnancy outcome considered to be an SAE should be reported within the timelines and using the procedures described in Section 9.2.2.

9.5 Other Safety Assessments

ECG, including assessment of QTcF-interval (using a suitable, validated method) and serum potassium will be performed at screening to exclude patients at high risk of QT-interval prolongation from participation in the study.

During the study, safety will be assessed through vital signs monitoring, SpO2 monitoring, and participant surveys, as shown in the SoA.

Participants will be asked to complete the FLU-PRO© Plus questionnaire and the daily survey (which includes the FLU-PRO© Plus Global Additional Daily Diary Items). Events qualifying as AEs will be recorded on the eCRF and reported as described in Section 9.2.

9.6 Serological and Other Assessments

Blood samples will be collected from all randomized participants at baseline on Day 1, and on Day 28 for serological testing in accordance with emerging, suitable and validated assays.

An additional blood sample will also be collected from participants offering optional consent for storage of samples at -70 °C for a period of up to 2 years. These samples will be used for future testing in accordance with emerging, disease-related data and suitable, validated assays.

Additional details regarding sample collection, processing and storage will be described in the Laboratory Manual.

9.7 PK Assessments

The aim of the PK assessments is to gather essential information about the exposure-response relationship for COVID-19 treatment and prevention. The sampling strategy in this study has been developed to maximize the value of isolated samples. Minimum concentration (C_{min}) on Day 7 will be assessed in all participants in the experimental arms (Arms B to E) to coincide with the primary endpoint, and on Day 3 in participants in the ASAQ and PA arms (Arms B and C).

A single plasma and/or whole blood PK sample will be collected on these days. The timing of the sample collections will be as follows:

- Day 3 samples for participants in the ASAQ and PA arms will be collected prior to the last dose of IMP and approximately 24 hours after the previous IMP dose.
- Day 7 samples for participants in all experimental arms (Arms B to E) will be collected prior to the last dose of IMP (if IMP administration is still scheduled for Day 7) and relative to the previous dose of IMP administered as follows:
  - 24 (±2) hours post-dose for SOF/DCV
  - 96 (±2) hours post dose for ASAQ, PA
  - 12 (±1) hours post dose for FPV-NTZ.

The basic requirements for PK sampling are as follows:

1. Accurate record of the time of IMP administration (for the dose administered prior to the blood sampling) (dd:mm:yy; hh:mm)
2. Accurate recording of the time of blood sampling (dd:mm:yy; hh:mm).

Blood samples will be collected and processed as outlined in the study-specific Laboratory Manual. Additional details regarding storage and shipment will be also be described in the Laboratory Manual.

Details of PK analytical procedures will be described in the Analytical Plan.

9.8 Biohazard Containment

As the transmission of SARS-CoV-2 and other respiratory droplet pathogens can occur through contact with respiratory droplets and contaminated surfaces, precautions will be employed by all personnel in the handling of all specimens for this study.

All dangerous goods and materials, including diagnostic specimens and infectious substances, must be transported using packaging mandated by national and regional regulations.

Details of these procedures will be described in the Laboratory Manual and will comply with relevant IATA Dangerous Goods Regulations.

10. DATA MANAGEMENT

The Investigator will maintain paper or electronic source documentation for all study participants. Participant information will be captured in an eCRF. The Clinical Data Management System will comply with regulatory guidelines and requirements for electronic systems.

Data validation and quality control procedures will be detailed in the Data Management Plan.

Medical history and AE data will be coded using the Medical Dictionary for Regulatory Activities (MedDRA) version 22.1 or later. Concomitant medications will be coded using the latest 2020 release of the WHO Drug Dictionary in effect at the time of study start.

11. STATISTICAL CONSIDERATIONS

11.1 Sample Size Determination

Primary Endpoint of Viral Clearance

Assuming that the percentage of participants with viral clearance by Day 7 is 20% in the control arm (Arm A), a sample size of 50 participants per arm will provide at least 80% power to detect an increase in viral clearance to 50% for a new treatment regimen. This assumes a two-sided, 5% type 1 error rate and a loss to follow up rate of 20%. The assumptions for this calculation are supported by a published analysis of a trial of favipiravir versus lopinavir/ritonavir, where the percentage with viral clearance by Day 7 was 65% for favipiravir (FPV) versus 20% for lopinavir/ritonavir (LPV/RTV) [9]. Issues of multiplicity have not been considered in this exploratory study.

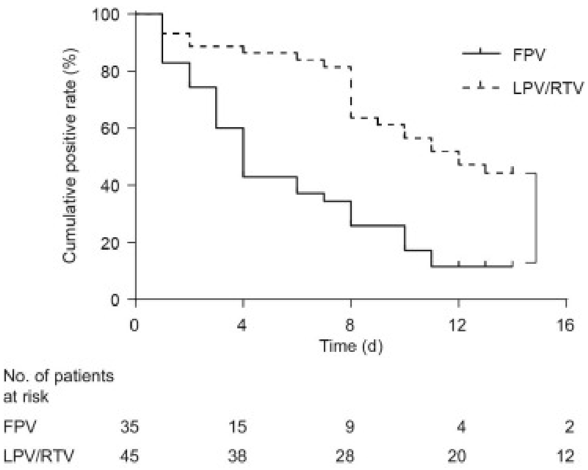

Figure 2: Kaplan-Meier survival curves for time to viral clearance per treatment

The aim of this exploratory study is to demonstrate similar high proportions of viral clearance for new candidate treatments. Demonstration of these benefits in any study treatment arms would justify taking these specific arms forward into a subsequent, separate, confirmatory, phase 3 trial, with a new endpoint of clinical recovery.

11.2 Populations for Analyses

For analysis purposes, the following populations are defined and will be described in greater detail in the trial Statistical Analysis Plan:

Population	Description
Safety Analysis Set	All enrolled participants who receive at least one dose of randomized treatment
Modified Intention to Treat (mITT)	All enrolled participants with confirmed SARS-CoV-2 infection who receive at least one dose of randomized treatment
As Treated	All enrolled participants who complete the course of randomized treatment and have viral load results available on Day 7

11.3 Statistical Analyses

The Statistical Analysis Plan will be developed and finalized before database lock and will describe the participant populations to be included in the analyses, the detailed analytical plans with endpoints and procedures for accounting for missing, unused, and spurious data.

An Interim Monitoring Plan will also be developed to describe approaches for re-estimation of sample size in order to preserve the pre-specified power of 80%.

This section presents a brief summary of the planned statistical analyses of the primary and secondary endpoints (Section 3).

11.3.1 Efficacy analyses

Demographic characteristics (age, sex, race) of each study group will be tabulated.

The mean age (plus range and standard deviation) by sex of the enrolled participants, as a whole and per group, will be calculated.

Primary analyses: The primary analyses will be conducted on the mITT population overall. Participants randomized to each active treatment will be compared to participants randomized to SOC (Arm A). The primary analysis will make use of all participants randomized to SOC, whether contemporaneously enrolled or not. Due to anticipated heterogeneity in risk of disease progression, pre-specified baseline variables, including age, body mass index (BMI), and days of symptoms at time of enrollment will be included in the model to increase precision.

Sensitivity analysis: The primary analyses will be repeated, replacing randomization arm by actual treatment to account for possible off-label use of and noncompliance to the investigational products.

Subgroup analyses: All subgroup analyses will be pre-specified in the Statistical Analysis Plan. Any further subgroup analyses will be considered *ad hoc*.

Missing Data: Due to the design of the study and retention activities, measurable outcomes are expected for all participants. However, in the unlikely event of a missing test result, the missing data will be imputed.

11.3.2 Secondary endpoints

All secondary endpoints will be assessed in the mITT population overall and by treatment arm.

Endpoints related to SpO2 will be calculated for all participants that had SpO2 ≥95% on Day 1.

11.3.2.1 Safety analyses

All safety analyses will be performed on the Safety Analysis Set. SAEs, AEs, treatment-related AEs, and discontinuations due to AEs will be compared by study group.

11.3.2.2 Hospitalization

Hospitalization rates between the groups will be compared using logistic regression stratified by site. Number of days hospitalized will be described graphically and by median and interquartile rage.

11.3.2.3 Disease severity

Disease severity, as measured by the WHO Ordinal Scale for Clinical Improvement (Appendix 5), will be compared between the groups using a proportional odds model.

11.3.2.4 Symptom resolution

Days with fever after randomization, respiratory symptoms after randomization, and Sp02<93% after randomization will be modeled using Poisson regression stratified by site with an offset for number of days of observation.

Data from the FLU-PRO© Plus questionnaire (Appendix 3) and the FLU-PRO© Plus Global Additional Daily Diary Items (Appendix 4) will be analysed in accordance with guidance from Leidos Biomedical Research, Inc. Details will be described in the Statistical Analysis Plan.

11.3.3 Exploratory endpoints

Limited sparse PK sampling will be conducted in all participants in the experimental arms of the study using C_{min} plasma and/or whole blood concentrations tailored to each of the main study drugs. For the majority of drugs this will be conducted on Day 7 only, to minimize exposure of healthcare staff to infectious patients through site visits. For long half-life drugs such as pyronaridine and amodiaquine that will only be dosed during the first days of the study, an additional plasma and/or whole blood C_{min} sample will be collected pre-dose on Day 3 to ensure robust quantification of drugs and/or their major circulating active metabolite.

A number of potential analyses are possible with these plasma or whole blood concentration data. Correlations of C_{min} with time to undetectable virus will be analyzed by Cox regression and the difference in proportions for other endpoints can be evaluated using Pearson's chi-square or Fisher's exact test (as appropriate). If warranted, differences in plasma and whole blood concentrations between individuals defined as "responders" versus those defined as "non-responders" to therapy will be assessed using a ttest, in the case that differences in response to therapy between participants is evident.

11.3.4 Combined study analysis

This protocol is based on a model protocol for consideration by multiple institutions as they evaluate the efficacy of experimental treatments for SARS-CoV-2 infection. Individual participant data from similar studies may be pooled into a combined study analysis. De-identified data from the present study will be made available for these purposes in accordance with the funder's open access policy (https://www.gatesfoundation.org/how-we-work/general-information/open-access-policy).

12. STUDY MONITORING

12.1 Safety Review Team

The Safety Review Team (SRT) will monitor the conduct of the study through monthly summary reports of arms of accrual, baseline characteristics, AEs, laboratory results and protocol deviations. On a weekly basis, the Medical Monitor (one of the members of the SRT) will review by-arm summaries of premature study discontinuations and premature study treatment discontinuations (and reasons) and AEs.

A detailed description of the operational aspects of the Safety Review Team will be documented in the Safety Review Team Charter.

12.2 Independent Monitor

Study conduct will be monitored by an independent monitor. Monitors will conduct remote monitoring and will visit the sites as required. Review of individual participant records, including consent forms, eCRFs, supporting data, laboratory specimen records, and endpoints through laboratory and medical records (physicians' progress notes, nurses' notes, individuals' hospital charts) will be performed as detailed in the Clinical Monitoring Plan, to ensure protection of study participants, compliance with the protocol, and accuracy and completeness of records. The monitors also will inspect the sites' regulatory files to ensure that regulatory requirements are being followed, and the sites' pharmacies to review product storage and management.

The Clinical Monitoring Plan will describe these activities and will take into consideration necessary adaptations to ensure the safety of the monitor with regard to infection control and the fact that physical access to sites may be limited.

12.3 Data Monitoring Committee

An independent DMC will be convened for this study with expertise in respiratory viruses, antiviral therapies and shedding, and emerging epidemics. A biostatistician will also form part of the DMC. The purpose of the DMC is to monitor the study for safety, social harms, and efficacy. The DMC will evaluate the progress of the project, including periodic assessments of accrual, retention, safety, performance and variation of the project site, and other factors that can affect project implementation.

The DMC will review and approve modifications to the overall enrollment target based on the observed drop-out rate. Due to the anticipated rapid speed of enrollment and the short duration of the study, no formal interim analyses for futility will be performed. The DMC will review severe acute respiratory syndrome coronavirus 2 (SARS-CoV-2) endpoints.

The DMC will conduct interim reviews as specified in the DMC Charter when adequate data have been accrued. Meetings will be convened by teleconference. Semi-blinded reports containing participant characteristics, AEs, SAEs, and other safety and efficacy data will be sent to the protocol team and DMC members the week prior to the DMC meeting. The DMC members will also receive pooled reports of accrual and retention rates to enable them to provide recommendations regarding a possible sample size increase in an un-biased way.

The objectives and responsibilities of the DMC, and the DMC operational procedures will be detailed in the DMC Charter which will be ratified by the DMC members prior to the study start.

12.4 Scientific Advisory Committee

A scientific advisory committee (SAC) will be established for this study. The scientific advisory committee will be consulted in the event of new evidence emerging that could impact the scientific rationale of the trial and might require:

- changes to treatment arm drugs or doses or duration of IMP administration, or
- changes to other aspects of the study design.

13. ETHICAL CONSIDERATIONS

13.1 Regulatory and Ethical Considerations

The study will be conducted according to GCP, the Belmont Report, the Declaration of Helsinki, and South Africa legal requirements regarding clinical trials. The study protocol and relevant supporting documents will be submitted for review and approval by the SAHPRA and the Human Research Ethics Committee (HREC) responsible for oversight of research conducted at the study site. The study protocol will be registered with the South African National Clinical Trial Registry (www.sanctr.gov.za) / National Human Research Ethics Committee (www.ethicsapp.co.za) and www.ClinicalTrial.gov. Six-monthly progress reports will be submitted to SAHPRA and HREC for the duration of the study, and as requested. Upon completion or premature termination of the study, the Investigator will provide HREC and SAHPRA with a summary of the study’s outcome, and any reports required.

13.2 Informed Consent Process

The principles of informed consent in the current edition of the Declaration of Helsinki will be implemented before any protocol-specified procedures or interventions are carried out. The pre-screening consent form will describe any information that will be gathered or procedures that will be followed during pre-screening. The separate screening and main study electronic consent form(s) will describe the purpose of the study, the procedures to be followed, and the risks and benefits of participation. Consent for storage of samples for future testing will also be requested, but will be optional. Failure to provide consent for sample storage will not exclude a volunteer from study participation. In the event of a female participant becoming pregnant, consent for follow-up of the pregnancy and outcome thereof will be requested. Potential participants will have the opportunity to have any questions answered before and after signing either the pre-screening consent form or the ICF. The informed consent process and all questions raised will be documented.

The study staff who conduct the informed consent process will also sign the ICF(s). A copy of the consent form(s) will be given to the participant, and this fact will be documented in the participant's record.

A participant who is rescreened will sign new ICF(s); eligibility for the study must be re-checked prior to enrollment with regard to all other screening procedures.

13.3 Study Records

The study site will establish a standard operating procedure for confidentiality protection. The site will ensure that study records including administrative documentation and regulatory documentation as well as documentation related to each participant enrolled in the study, including ICFs, locator forms, case report forms, notations of all contacts with the participant, and all other source documents are stored in a secure manner.

13.4 Confidentiality

All laboratory specimens, evaluation forms, reports, and other records will be identified in a manner designed to maintain participant confidentiality. The exceptions are SARS-CoV-2 testing results, which are subject to local and national reporting. This may be name-based depending on local requirements. Local public health may contact participants diagnosed with SARS-CoV-2 for the purpose of surveillance and contact notification. Participants will be informed prior to SARS-CoV-2 testing that results are reportable and may lead to contact by local public health if results are positive for infection.

All records will be kept in a secure storage area with limited access. Clinical information will not be released without the written permission of the participant, except as necessary for monitoring, and auditing by the regulatory authorities or the Human Research Ethics Committee (HREC).

The Principal Investigator or designee and all employees and co-workers involved with this study may not disclose or use for any purpose other than performance of the study, any data, record, or other unpublished confidential information disclosed to those individuals for the purpose of the study. All computers are password-protected and records can only be accessed by authorized study staff.

13.5 Blood Volume

Anticipated blood draws for participants in the experimental treatment arms will amount to the following total blood volumes:

Assessment	Maximum volume per sample	Maximum no. of samples	Total volume
Serum potassium	5 mL	1	5 mL
PK	8 mL	2	16 mL
Serology	5 mL	2	10 mL
Storage for future testing	20 mL	2	40 mL
		**TOTAL**	**71 mL**

For participants in the SOC arm (Arm A), no PK samples will be drawn and the anticipated total blood volume drawn will therefore be 55 mL.

Therefore, for all participants, even if the situation arises where multiple additional blood draws for unanticipated safety monitoring are required, the total volume drawn should not exceed 480 mL over an 8 week period.

14. REFERENCES

1. Hoffmann M, Kleine-Weber H, Schroeder S, et al. SARS-CoV-2 cell entry depends on ACE2 and TMPRSS2 and is blocked by a clinically proven protease inhibitor. 2020 Mar; Cell. pii: S0092-8674(20)30229-4 (pre-publication).
2. Diao B, Wang C, Wang R, et al. Human Kidney is a Target for Novel Severe Acute Respiratory Syndrome Coronavirus 2 (SARS-CoV-2) Infection. 2020:doi: 10.1101/2020.03.04.20031120 J medRxiv.
3. Zhang C, Shi L, Wang FS. Liver injury in COVID-19: management and challenges. Lancet Gastroenterol Hepatol. 2020:doi: 10.1016/S2468-1253(20)30057-1.
4. Driggin E, Madhavan MV, Bikdeli B, et al. Cardiovascular considerations for patients, health care workers, and health systems during the COVID-19 pandemic. J Am Coll Cardiol. 2020;75:2352-71.
5. Wu CI, Postema PG, Arbelo E, et al. SARS-CoV-2, COVID-19, and inherited arrhythmia syndromes. Heart Rhythm. 2020:https://doi.org/10.1016/j.hrthm.2020.03.024.
6. Arshad U, Pertinez H, Box H, Tatham L, Rajoli R, Curley P, Neary M, Sharp J, Liptrott N, Valentijn A, David C, Rannard S, O'Neill P, Aljayyoussi G, Pennignton S, Ward S, Hill A, Back D, Khoo S, Bray P, Biagini G, Owen A. Prioritisation of COVID-19 Drug Repurposing Opportunities Based on Plasma and Lung Concentrations Derived from Established Human Pharmacokinetics. Clinical Pharmacology & Therapeutics. In Press
7. Internal communication, Medicines for Malaria Venture. May 2020.
8. National Institute for Communicable Diseases. Clinical management of suspected or confirmed COVID-19 disease. Version 5, 24 Aug 2020.
9. Cai Q, Yang M, Liu D, et al. Experimental Treatment with Favipiravir for COVID-19: An Open-Label Control Study, Engineering. 2020:doi.org/10.1016/j.eng.2020.03.007
10. Chen C, Zhang Y, Huang J, et al. Favipiravir versus Arbidol for COVID-19: A Randomized Clinical Trial. medRxiv 2020.03.17.20037432; doi: https://doi.org/10.1101/2020.03.17.20037432
11. Huang C, Wang Y, Li X, et al. Clinical features of patients infected with 2019 novel coronavirus in Wuhan, China. Lancet. 2020 Feb;395(10223):497-506.
12. Zhu N, Zhang D, Wang W, et al. A novel coronavirus from patients with pneumonia in China, 2019. N Engl J Med. 2020 Feb;382(8):727-733.
13. Chen N, Zhou M, Dong X, et al. Epidemiological and clinical characteristics of 99 cases of 2019 novel coronavirus pneumonia in Wuhan, China: a descriptive study. Lancet. 2020 Feb;395(10223):507-513.
14. https://sacoronavirus.co.za/2020/06/29/update-on-covid-19-29th-june-2020/
15. WHO News Release. Statement on the second meeting of the International Health Regulations (2005) Emergency Committee regarding the outbreak of novel coronavirus (2019-nCoV). 2020 Jan 30.
16. WHO Director-General’s opening remarks at the media briefing on COVID-19 – 11 March 2020. World Health Organisation. 11 March 2020.
17. White House Press Release. https://www.whitehouse.gov/presidential-actions/proclamation-declaring-national-emergency-concerning-novel-coronavirus-disease-covid-19-outbreak. 2020 Mar 13.

18. Disaster Management Act 57 of 2002, South African Government. www.gov.za
19. Cai J, Xu J, Lin D, et al. A case series of children with 2019 novel coronavirus infection: clinical and epidemiological features. Clin Infect Dis. 2020 Feb. pii: ciaa198.
20. Linton NM, Kobayashi T, Yang Y, et al. Incubation period and other epidemiological characteristics of 2019 novel coronavirus infections with right truncation: a statistical analysis of publicly available case data. J Clin Med. 2020 Feb;9(2). pii: E538.
21. WHO News Release. Shortage of personal protective equipment endangering health workers worldwide. 2020 Mar 03.
22. Chen J. Pathogenicity and transmissibility of 2019-nCoV-a quick overview and comparison with other emerging viruses. Microbes Infect. 2020 Mar;22(2);69-71.
23. Uzun T, Toptas O. Artesunate: could be an alternative drug to chloroquine in COVID-19 treatment? Chin Med 2020; 15:54. https://dio.org/10.1186/s13020-020-00336-8.
24. Investigator's Brochure. Pyramax Tablets and Granules (pyronaridine-artesunate). Edition no. 10, Sep 2018.
25. Wilson CM. 296 - Antiparasitic Agents. In: Long SS, ed. Principles and Practice of Pediatric Infectious Diseases (Fourth Edition). London 2012:1518-45.e3.
26. DrugBank. Nitazoxanide. Available at: https://www.drugbank.ca/drugs/DB00507. Accessed 17/04/2020 2020.
27. Keiser J, Utzinger J. Chapter 8 - The Drugs We Have and the Drugs We Need Against Major Helminth Infections. In: Zhou X-N, Bergquist R, Olveda R, Utzinger J, eds. Advances in Parasitology. Vol. 73: Academic Press, 2010:197-230.
28. Rossignol JF, El-Gohary YM. Nitazoxanide in the treatment of viral gastroenteritis: a randomized double-blind placebo-controlled clinical trial. Alimentary Pharmacology & Therapeutics 2006; 24:1423-30.
29. Korba BE, Elazar M, Lui P, Rossignol JF, Glenn JS. Potential for Hepatitis C Virus Resistance to Nitazoxanide or Tizoxanide. Antimicrobial Agents and Chemotherapy 2008; 52:4069-71.
30. Korba BE, Montero AB, Farrar K, et al. Nitazoxanide, tizoxanide and other thiazolides are potent inhibitors of hepatitis B virus and hepatitis C virus replication. Antiviral Research 2008; 77:56-63.
31. Dang W, Xu L, Ma B, et al. Nitazoxanide Inhibits Human Norovirus Replication and Synergizes with Ribavirin by Activation of Cellular Antiviral Response. Antimicrobial Agents and Chemotherapy 2018; 62:e00707-18.
32. Koszalka P, Tilmanis D, Hurt AC. Influenza antivirals currently in late-phase clinical trial. Influenza Other Respir Viruses 2017; 11:240-6.
33. Rossignol J-F. Nitazoxanide: A first-in-class broad-spectrum antiviral agent. Antiviral Research 2014; 110:94-103.
34. Pizzorno A, Padey B, Terrier O, Rosa-Calatrava M. Drug Repurposing Approaches for the Treatment of Influenza Viral Infection: Reviving Old Drugs to Fight Against a Long-Lived Enemy. Frontiers in Immunology 2019; 10.
35. Haffizulla J, Hartman A, Hoppers M, et al. Effect of nitazoxanide in adults and adolescents with acute uncomplicated influenza: a double-blind, randomized, placebo-controlled, phase 2b/3 trial. Lancet Infect Dis 2014; 14:609-18.
36. Clerici M, Trabattoni D, Pacei M, Biasin M, Rossignol J-F. The anti-infective Nitazoxanide shows strong immumodulating effects (155.21). 2011; 186:155.21-.21.

37. Miner K, Labitzke K, Liu B, et al. Drug Repurposing: The Anthelmintics Niclosamide and Nitazoxanide Are Potent TMEM16A Antagonists That Fully Bronchodilate Airways. Front Pharmacol 2019; 10:51-.
38. Gao et al, 2020. Structure of the RNA-dependent RNA polymerase from COVID-19 virus
39. Jin, Z. et al. Structure of Mpro from COVID-19 virus and discovery of its inhibitors. Nature https://doi.org/10.1038/ s41586-020-2223-y (2019).
40. Elfiky et al 2020 Anti-HCV, nucleotide inhibitors, repurposing against COVID-19.
41. Elfiky et al 2020, Ribavirin, Remdesivir, Sofosbuvir, Galidesivir, and Tenofovir against SARSCoV-2 RNA dependent RNA polymerase (RdRp): A molecular docking study.
42. Ju et al. Nucleotide Analogues as Inhibitors of SARS-CoV Polymerase.
43. Chien et al. Nucleotide Analogues as Inhibitors of SARS-CoV-2 Polymerase
44. Ahmad et al, 2020 Prediction of small molecule inhibitors targeting the novel coronavirus (SARS-CoV-2) RNA-dependent RNA polymerase
45. Beck et al, 2020 Predicting commercially available antiviral drugs that may act on the novel coronavirus (SARS-CoV-2) through a drug-target interaction deep learning model
46. Alabboud et al, 2020. In silico study of various antiviral drugs, vitamins, and natural substances as potential binding compounds with SARS-CoV-2 main protease.
47. Rodon et al, Search for SARS-CoV-2 inhibitors in currently approved drugs to tackle COVID-19 pandemia. bioRxiv 2020.04.23.055756; doi: https://doi.org/10.1101/2020.04.23.055756.
48. Liu et al. Evaluation of 19 antiviral drugs against SARS-CoV-2 Infection. bioRxiv 2020.04.29.067983; doi: https://doi.org/10.1101/2020.04.29.067983.
49. Sovaldi® (sofosbuvir) tablets for oral use. FDA. FDA AccessData. Available from:https://www.accessdata.fda.gov/drugsatfda_docs/label/2015/204671s002lbl.pdf
50. De Freitas et al. (2019) Yellow fever virus is susceptible to sofosbuvir both in vitro and in vivo. PLoS Negl Trop Dis 13(1): e0007072. https://doi.org/10.1371/journal. pntd.0007072
51. Bullard-Feibelman, K., Govero, J., Zhu, Z., Salazar, V., Veselinovic, M., Diamond, M. and Geiss, B., 2017. The FDA-approved drug sofosbuvir inhibits Zika virus infection. Antiviral Research, 137, pp.134-140.
52. Gan, C., Lim, S., Chee, C., Yusof, R. and Heh, C., 2017. Sofosbuvir as treatment against dengue?. Chemical Biology & Drug Design, 91(2), pp.448-455.
53. Ferreira A, Reis P, de Freitas C, et al. Beyond Members of the Flaviviridae Family, Sofosbuvir Also Inhibits Chikungunya Virus Replication. Antimicrobial Agents and Chemotherapy 2018; 63(2):pp.e01389-18.
54. Iranian Clinical Trial Registry. 2020 [online] Available at: https://www.irct.ir/
55. Rajoli R, Pertinez H, Arshad U, et al. Dose prediction for repurposing nitazoxanide in SARS-CoV-2 treatment or chemoprophylaxis. medRxiv 2020.05.01.20087130; doi: https://doi.org/10.1101/2020.05.01.20087130.
56. NICD COVID-19 Surveillance in Selected Hospitals, 14 May 2020.
57. Rodino KG, Espy MJ, Buckwalter SP, et al. Evaluation of saline, phosphate buffered saline and minimum essential medium as potential 2 alternatives to viral transport media for SARS-CoV-2 testing. J Clin Microbiol. 2020. 2020 Mar 30.
58. Powers JH, Guerrero ML, Leidy NK, et al. Development of the Flu-PRO: a patient-reported outcome (PRO) instrument to evaluate the symptoms of influenza. BMC Infect Dis. 2016;16:1.

59. Powers JH, Bacci ED, Guerrero ML, et al. Evaluation of the performance properties of the InFLUenza Patient-Reported Outcome (FLU-PRO©) instrument. Value in Health. 2018 Feb;21(2):210-218.
60. Powers JH, Bacci ED, Leidy NK, et al. Performance of the inFLUenza Patient-Reported Outcome (FLU-PRO) diary in patients with influenza-like illness (ILI). PloS One. 2018 Mar;13(3):e0194180.

APPENDICES

APPENDIX 1: STUDY GOVERNANCE

The following Investigators and Institutional Affiliations are established. Designees may be provided, as appropriate.

Name	Role	Institution
[redacted]	Principal Investigator	Ezintsha, Wits Reproductive Health & HIV Institute<br>University of the Witwatersrand<br>5th Floor, Building D<br>Sunnyside Office Park<br>32 Princess of Wales Terrace<br>Parktown, Johannesburg<br>South Africa
[redacted]	Lead Investigator	<u>Main Site:</u><br>Building C<br>Sunnyside Office Park<br>32 Princess of Wales Terrace<br>Parktown, Johannesburg<br><br><u>Satellite Site:</u><br>Charlotte Maxeke Johannesburg Academic Hospital<br>Area 585, Level 8<br>Jubilee Road<br>Johannesburg, 2193<br>South Africa

APPENDIX 2: CONTACT DETAILS

Site	
Institution:<br>Address:	Ezintsha, Wits Reproductive Health & HIV Institute<br>University of the Witwatersrand<br>5th Floor, Building D<br>Sunnyside Office Park<br>32 Princess of Wales Terrace<br>Parktown, Johannesburg
Main Site:	Building C<br>Sunnyside Office Park<br>32 Princess of Wales Terrace<br>Parktown, Johannesburg<br>South Africa
Principal Investigator:<br>Email:<br>Telephone:	[redacted]
Lead Investigator:<br>Email:<br>Mobile:	[redacted]
Research Pharmacist:<br>[redacted]<br>Telephone:	[redacted]
IRB/Ethics Committee:<br>Research Administrator:<br>Telephone:	Human Research Ethics Committee<br>[redacted]

Satellite Site	
Satellite Site:	Charlotte Maxeke Johannesburg Academic Hospital<br>Area 585, Level 8<br>Jubilee Road<br>Johannesburg, 2193<br>South Africa

Sponsor	
Institution:	Shin Poong Pharm. Co., Ltd.
Address:	161 Yoksam-Ro, Gangnam-Gu<br>Seoul, 06246<br>Republic of Korea
Project Director:<br>Email:<br>Telephone:	[redacted]

Funder	
Institution:	Medicines for Malaria Venture
Address:	ICC – Block G, 3rd Floor<br>20, Route de Pré-Bois<br>PO Box 1826<br>1215 Geneva 15<br>Switzerland
Chief Medical Officer:<br>Email:<br>Telephone:	[redacted]
Project Director:<br>Email:<br>Telephone:	[redacted]
Clinical Trial Manager:<br>Email:<br>Telephone:	[redacted]

Cinical Research Organisation	
Institution:	Triclinium Clinical Development (TCD)
Address:	Design House<br>121 Amkor Road<br>Lyttleton Manor<br>Centurion, 0157<br>South Africa
Project Manager:<br>Email:<br>Telephone:	[redacted]

Independent Medical Monitor	
Institution:	HJ-Clinical Trial Consultancy
Address:	15 Oaklands, Fancourt<br>George, 6529<br>South Africa
Medical Monitor:<br>Email:<br>Telephone:	[redacted]

Pharmacovigilance	
Institution:	Quanticate
Address:	Bevan House 9-11 Bancroft Court<br>Hitchin<br>Hertfordshire<br>SG5 1LH<br>United Kingdom
QPPV:<br>Email:<br>Telephone:	[redacted]

APPENDIX 3: THE INFLUENZA PATIENT-REPORTED OUTCOME INSTRUMENT (FLU-PRO© Plus)

To be completed on D1-14, D21 and D28:

[redacted]

Copyright © 2014- 2020 Leidos Biomedical Research, Inc. All Rights Reserved

[redacted]

Copyright © 2014- 2020 Leidos Biomedical Research, Inc. All Rights Reserved

APPENDIX 4: DAILY SURVEY

To be completed on D1-14, D21 and D28

[redacted]

[redacted]

[redacted]

APPENDIX 5: WHO ORDINAL SCALE FOR CLINICAL IMPROVEMENT

Patient State	Descriptor	Score
Uninfected	No clinical or virological evidence of infection	0
Ambulatory	No limitation of activities	1
	Limitation of activities	2
Hospitalized: mild disease	Hospitalized, no oxygen therapy	3
	Oxygen by mask or nasal prongs	4
Hospitalized: severe disease	Non-invasive ventilation or high-flow oxygen	5
	Intubation and mechanical ventilation	6
	Ventilation + additional organ support (pressors, RRT, ECMO)	7
Dead	Death	8

WHO COVID-19 Core Protocol
SOLIDARITY Trial
Version 10.0, March 22, 2020

APPENDIX 6: PROHIBITED CONCOMITANT MEDICATIONS

The following drugs are known to induce prolongation of the QT-interval. Their use is not permitted in conjunction with the study drugs. Participants using these drugs at baseline, or those anticipated to require these during the study, should not be enrolled in the study.

- antiarrhythmics (e.g., amiodarone, disopyramide, dofetilide, ibutilide, procainamide, quinidine, hydroquinidine, sotalol)
- neuroleptics (e.g., phenothiazines, sertindole, sultopride, chlorpromazine, haloperidol, mesoridazine, pimozide, or thioridazine) and antidepressant agents known to cause significant QT-interval prolongation (e.g., citalopram, escitalopram)
- certain antimicrobial agents, including agents of the following classes:
  - macrolides (e.g,. erythromycin, clarithromycin)
  - fluroquinolones (e.g., moxifloxacin, sparfloxacin)
  - imidazole and triazole antifungal agents
  - pentamidine
  - saquinavir
- certain non-sedating antihistamines (e.g., terfenadine, astemizole, mizolastine)
- cisapride
- droperidol
- domperidone
- bepridil
- diphemanil
- probucol
- levomethadyl
- methadone
- vinca alkaloids
- arsenic trioxide.

Use of the following drugs is not permitted during the study due to potential drug-drug interactions. Participants using these drugs at baseline, or those anticipated to require these during the study, should not be enrolled in the study.

- protease inhibitor-based antiretroviral regimens
- efavirenz
- rifampicin
- carmabazepine.
