# Supplementary material for: Safety and efficacy of four drug regimens versus standard-of-care for the treatment of symptomatic outpatients with COVID-19: A randomised, open-label, multi-arm, phase 2 clinical trial
Source: eBioMedicine. 2022 Nov 1;86:104322. doi: 10.1016/j.ebiom.2022.104322 (PMC9624152; doi:10.1016/j.ebiom.2022.104322)
Supplement: Caption for Supplementary Material [file mmc2.docx]

**Supplementary appendix**

Table S1 Post-screening schedule of patient assessments.

Table S2 Bioanalytical methods to determine drug concentrations.

Table S3 Sensitivity analysis of the primary endpoint: incidence of SARS-CoV-2 clearance on day 7 based on qualitative RT-PCR.

Table S4 Primary analysis of the incidence of SARS-CoV-2 clearance on day 7 based on RT-PCR by subgroup (mITT population). See also Figure S1.

Table S5 Incidence of SARS-CoV-2 clearance on day 7 based on viral culture (mITT population).

Table S6 Incidence of SARS-CoV-2 clearance on days 3, 10, 14, 21, and 28 based on RT-PCR (mITT population). See also Figure S1.

Figure S1 Incidence of SARS-CoV-2 clearance on days 3, 10, 14, 21, and 28 based on RT-PCR (mITT population). See also Table S6.

Table S7 Repeated measure analysis of log_10_ viral load of SARS-CoV-2 change from baseline (mITT population).

Table S8 Repeated measure analysis of log_10_ viral load of SARS-CoV-2 change from baseline in high-risk patients (mITT population).

Table S9 Proportional odds model for disease progression for day 7, 14, 21, and 28 (mITT population).

Table S10 Cox regression analysis of time to first zero WHO Ordinal Scale score for Clinical Improvement (mITT population).

Table S11 Poisson regression analysis for the proportion of days with fever, SpO_2_ values <93%, or respiratory symptoms after randomisation (mITT population).

Figure S2 FLU-PRO Plus questionnaire scores and changes from baseline (mITT population).

Table S12 Investigational drug blood or plasma concentrations (pharmacokinetic population).

Figure S3 Drug plasma or blood concentrations at day 3 and day 7 in patients with or without SARS-CoV-2 clearance based on RT-PCR at day 7 (pharmacokinetic population).

Table S13 Treatment emergent adverse events of any cause and maximum severity (safety population).

Table S13 Treatment emergent adverse events considered to be study drug related (safety population).

Figure S4 Change in vital signs from baseline (safety population).

Figure S5 Shift from baseline to day 28 in serology (mITT population).
