# Supplementary material for: Safety and efficacy of four drug regimens versus standard-of-care for the treatment of symptomatic outpatients with COVID-19: A randomised, open-label, multi-arm, phase 2 clinical trial
Source: eBioMedicine. 2022 Nov 1;86:104322. doi: 10.1016/j.ebiom.2022.104322 (PMC9624152; doi:10.1016/j.ebiom.2022.104322)
Supplement: Supplementary Appendix Statistical Analysis Plan [file mmc3.pdf]

DATAMAP

A PHASE 2, EXPLORATORY, SINGLE CENTER, RANDOMIZED, OPEN LABEL, ADAPTIVE CLINICAL TRIAL TO COMPARE THE SAFETY AND EFFICACY OF FOUR DIFFERENT EXPERIMENTAL DRUG REGIMENS TO STANDARD OF CARE FOR THE TREATMENT OF SYMPTOMATIC OUTPATIENTS WITH COVID-19

SP-PA-COV-202

Statistical Analysis Plan

Author	[redacted]
Version	Version 1, Final amendment 2
Date of version	01-Mar-2022
Approved by	[redacted] Deputy Head of Research, Shin Poong Pharm. Co., Ltd.<br>[redacted] Chief Medical Officer, MMV

Signatures

Author:<br>DATAMAP GmbH	(Date)	(Signature)

Approved by:

[redacted]<br>Deputy Head of Research<br>Shin Poong Pharm. Co., Ltd.	(Date)	(Signature)
[redacted]<br>Chief Medical Officer<br>MMV	(Date)	(Signature)

Document - History

Version	Date	Responsible	Change information
Version 1, Draft 1	20-Nov-2020	[redacted]	Initial draft version
Version 1, Draft 2	10-Dec-2020		Comments from draft 1 incorporated and outputs shells added.
Version 1, Draft 3	15-Dec-2020		Comments from wider team for draft 2 incorporated
Version 1, Draft 4	30-Jun-2021		Comments from wider team for draft 3 incorporated
Version 1, Draft 5	23-Jul-2021		Comments from wider team for draft 4 incorporated
Version 1, final	05-Aug-2021		Last comments incorporated. Track change mode removed.
Version 1, amendment 1	15-Oct-2021		Minor corrections and clarifications in text and TFL shells.<br>Added programming notes where necessary.<br>Added imputation rule for PK concentration and for viral load data.<br>Added clarification on calculation of days of symptoms at time of enrolment.<br>Aligned viral load endpoint with protocol, i.e. removed Day 21 and 28.<br>Clarified exploratory PK analysis: removed corresponding cox regression. Removed visit windows for PK as concentrations outside of visit windows should not be excluded from the analysis. Added corresponding footnotes to PK outputs.
Version 1, amendment 2	01-Mar-2022		For the subgroups of sero-negative participants and for high-risk participants, the following post-hoc analyses were added respectively:<br>• Summary of WHO Ordinal Scale for Clinical Improvement<br>• Kaplan Meier analysis of time to first zero WHO Ordinal Scale score for Clinical Improvement<br>• Cox regression analysis of time to first zero WHO Ordinal Scale score for Clinical Improvement

Table of Contents

Abbreviations 5
1. Introduction 6
2. Definitions and Data Conventions 6
3. Analysis Sets 9
4. General analysis considerations 9
5. Subject Disposition 10
6. Demographic Data and Baseline Characteristics 10
7. Prior/ Concomitant Medications 10
8. Efficacy Analysis 10
8.1 Primary variable(s) 10
8.2 Secondary and other variable(s) 11
8.2.1 Incidence of SARS-CoV-2 clearance 11
8.2.2 Time to clearance of SARS-CoV-2 12
8.2.3 Viral load of SARS-CoV-2 12
8.2.4 Hospitalization 13
8.2.5 Disease severity 13
8.2.6 Symptom resolution 15
8.2.7 Lower respiratory tract infection 15
8.2.8 All-cause Mortality 15
9. Exposure to Study Drug 16
10. Safety Analysis 16
10.1 Adverse events 16
10.2 Vital signs 17
11. Exploratory Analyses 17
12. Interim Analyses 18
13. Change to Protocol Specified Analyses 18
14. List of Tables and Figures 19
15. List of Data Listings and Statistical Tabulations 21
16. Output Shells 23
17. Appendices 104
17.1 FLU-PRO total and domain scoring 104

Abbreviations

AE	Adverse event
ASAQ	Artesunate-amodiaquine
ATC	Anatomical Therapeutic Chemical
BMI	Body mass index
CT	Cycle threshold
FLU-PRO	Influenza patient-reported outcome
FPV-NTZ	Favipiravir plus nitazoxanide
MAR	Missing at random
MedDRA	Medical Dictionary for Regulatory Activities
mITT	Modified Intention to Treat
LRTI	Lower respiratory tract infection
LSM	Least Square Means
PA	Pyronaridine-artesunate
PK	Pharmacokinetic
RR	Respiratory rate
RT-PCR	Reverse transcription polymerase chain reaction
SARS-CoV-2	Severe acute respiratory syndrome coronavirus 2
SE	Standard Error
SOC	Standard of care
SOF/DCV	Sofosbuvir/daclatasvir
SpO2	Oxygen saturation
TEAEs	Treatment emergent adverse event
WHO	World Health Organization

1. Introduction

This analysis plan is based on the study protocol SP-PA-COV-202 version 4, dated 17-November-2020. All analyses will be performed by DATAMAP GmbH, Freiburg, Germany using SAS®, version 9.4 in a Linux environment.

This is a randomized, single center, open label, adaptive, exploratory trial. The trial is comprising four experimental interventions (Arms B to E) to assess the efficacy of each intervention as measured by a reduction in viral shedding compared to standard of care (SOC) (Arm A). Participants are randomized to one of these treatment arms in a 1:1:1:1:1 ratio. The overarching goal of this study is to assess the efficacy of interventions to reduce virus shedding among adult outpatients with mild SARS-CoV-2 infection. Additional objectives include clinical outcomes related to SARS-CoV-2 infection and tolerability of the interventions.

2. Definitions and Data Conventions

Study days

Study days will be calculated as actual date minus date of study drug intake + 1 if the actual date is greater or equal to the date of study drug intake. If the actual date is earlier than the date of study drug intake the study day will be calculated as actual date minus date of study drug intake + 1. Hence the day of the study drug intake will be defined as Day 1.

Baseline

Baseline for analysis purposes if not otherwise defined will be the last measurement prior to the first study drug administration, or where this is not available, the earliest measurement performed on the first day of study drug administration.

Fever

Fever is defined as oral body temperature ≥38°C.

Baseline comorbidities

Baseline comorbidities will be defined by the participants having none, 1 or more than 1 comorbidity as defined in the pre-specified medical history terms in the eCRF.

Baseline viral load category

A baseline viral load category will be determined by quantitative RT-PCR results (copies/mL) of the N gene. Participants will be assigned to the low/high baseline viral load category based on the median baseline viral load of all participants in the mITT Analysis Set. Participants with viral load below the median are assigned to the low category, participants with viral load equal to or higher than the median are assigned to the high category.

High-risk participants

Participants will be assigned in the high-risk category if they have at least one of the following risk factors: BMI > 30, baseline comorbidities of at least one or more or aged > 60 years. Otherwise participants are assigned to the low risk category.

Time windows for analysis

To account for potential deviations from the visit schedule and unscheduled assessments, all "by time point" summaries will be based on analysis visits, which will be assigned based on a time window around the scheduled time point. Please note that the window is only used for the summaries by time point. In all participant-specific displays and time to event analyses the exact timing of the assessments will be used. If more than one assessment falls into a time window the one closest to the target time point will be used for summarization. If two assessments are equally close the later will be chosen. The following windows will be used:

Mid-nasal swab data

Scheduled time point	Window
Baseline	last assessment prior to or on the day of first study drug administration
Day 3	Day 3 - 4
Day 7	Day 6 - 8
Day 10	Day 9 - 11
Day 14	Day 12 - 16
Day 21	Day 19 - 23
Day 28	Day 26 - 30
End of study	Overall last available assessment

Viral culture data

Scheduled time point	Window
Baseline	last assessment prior to or on the day of first study drug administration
Day 7	Day 6 - 8
Day 10	Day 9 - 11

Vital signs data

Scheduled time point	Window
Baseline	Screening/enrollment assessment by study personnel prior to first study drug administration. If it is not available, the last assessment prior to or on the day of first study drug administration will be used.
Day 2	Day 2
Day 3	Day 3
Day 4	Day 4
Day 5	Day 5
Day 6	Day 6
Day 7	Day 7
Day 8	Day 8
Day 9	Day 9
Day 10	Day 10
Day 11	Day 11
Day 12	Day 12
Day 13	Day 13
Day 14	Day 14 - 16
Day 21	Day 19 - 23

Day 28	Day 26 - 30
End of study	Overall last available assessment

FLU-PRO Plus© questionnaire data

Scheduled time point	Window
Baseline	last assessment prior to or on the day of first study drug administration
Day 2	Day 2
Day 3	Day 3
Day 4	Day 4
Day 5	Day 5
Day 6	Day 6
Day 7	Day 7
Day 8	Day 8
Day 9	Day 9
Day 10	Day 10
Day 11	Day 11
Day 12	Day 12
Day 13	Day 13
Day 14	Day 14 - 16
Day 21	Day 19 - 23
Day 28	Day 26 - 30
End of study	Overall last available assessment

WHO Ordinal Scale for Clinical Improvement

Scheduled time point	Window
Baseline	last assessment prior to or on the day of first study drug administration
Day 7	Day 6 – 8
Day 14	Day 12 - 16
Day 21	Day19 – 23
Day 28	Day 26 – 30
End of study	Overall last available assessment

Serology

Scheduled time point	Window
Baseline	last assessment before dosing
Day 28	Day 26 – 30

3. Analysis Sets

The following analysis sets are defined:

Randomized Analysis Set

All enrolled participants who were randomized. Analyses will be performed by using randomized treatment.

Safety Analysis Set

All enrolled participants who receive at least one dose of randomized treatment will be included in the Safety Analysis Set. Analyses will be performed by using actual treatment.

Modified Intention to Treat (mITT) Analysis Set

All enrolled participants with confirmed SARS-CoV-2 infection (based on qualitative RT-PCR, either mid-nasal swab or saliva/combination sample) who receive at least one dose of randomized treatment will be included in the Modified Intention to Treat Analysis Set. Only randomized participants are included and randomized treatment will be used in the analysis. The primary analyses and all secondary endpoints except for safety analyses will be assessed using the mITT Analysis Set.

As Treated Analysis Set

All enrolled participants who complete the course of randomized treatment and have qualitative SARS-CoV-2 RT-PCR results (either mid-nasal swab or saliva/combination sample) for Day 7 (+/- 1 Day). Analyses will be performed by using actual treatment instead of randomized treatment. The As Treated analysis set will be used for a sensitivity analysis of the primary analysis. A participant will be considered as having completed the course of treatment if he/she took all scheduled experimental treatment doses (ASAQ or PA) or if he/she took at least 6 out of 7 scheduled experimental treatment doses (SOF/DCV) or 12 out of 14 scheduled experimental treatment doses (FPV-NTZ).

For FPV-NTZ arm both study drugs, FPV and NTZ, must reach at least 85% compliance. For NTZ the rules are based on tablet counts (as detailed above). For FPV the compliance calculated is based on the total dose, see section 9. Additionally, for FPV the intake of the loading dose (two times 1600 mg) on day 1 must be complete.

Pharmacokinetic (PK) Analysis Set

All enrolled participants who were randomized and received at least one dose of treatment and with at least one PK result available.

4. General analysis considerations

Continuous data will be summarised by treatment arm with the number of observations, mean, standard deviation, median, minimum and maximum, and quartiles. Categorical data will be summarised by number and percentage of participants per treatment arm and overall.

There will five treatment arms (Arm A to Arm E) with Arm A being standard of care (reference arm) and Arm B to Arm E being four different experimental drug regimens. The following treatment arm labels will be used in the outputs:

- Label used for treatment arm A: SOC
- Label used for treatment arm B: ASAQ
- Label used for treatment arm C: PA
- Label used for treatment arm D: FPV-NTZ
- Label used for treatment arm E: SOF/DCV

As treatment for Arm A is to be administered on an as needed basis only, all participants in this arm will be considered compliant and to have completed the course of treatment. Since this is an exploratory, screening study, multiplicity issues will not be considered.

The following variables will be used in covariate adjusted analyses: age at baseline (years), sex, BMI at baseline, baseline comorbidities, baseline viral load category and days of symptoms at time of enrollment. Of these variables age at baseline, sex and BMI at baseline will be non-missing for randomized participants. If a participant has no baseline comorbidities recorded, he will be counted in the 'None' category. If a patient's CRF item days of symptoms at time of enrolment is missing then it will be calculated as the duration from the medical history start date of Covid-19 to the informed consent date.

All RT-PCR-specific analyses will be using mid-nasal swab results only.

All analyses of quantitative SARS-CoV-2 RT-PCR (viral load) are based on the N gene.

5. Subject Disposition

Summary tables of participant disposition will be provided for the Randomized Analysis Set by treatment arm and overall. The following data will be displayed:

- The number and percentage of participants randomized, treated, completed, prematurely withdrawn.
- The number and percentage by reasons for withdrawal.

The number and percentage of participants in each analysis set will be summarized by treatment arm and overall. The reasons leading to exclusion from any analysis set will be presented as well.

All protocol deviations will be summarized by treatment arm and overall.

6. Demographic Data and Baseline Characteristics

Participant demographic data and baseline characteristics will be summarised by treatment arm and overall for the mITT Analysis Set. Standard summary statistics will be used, as appropriate. The following summaries will be generated:

- Participant demographics: sex, age (additionally split by sex), race, body weight, height, BMI.
- Baseline characteristics:
    - Temperature, pulse rate, respiratory rate (RR), SpO2, number of days with symptoms at time of enrollment and WHO Ordinal Scale score for Clinical Improvement.
- Medical and surgical history terms will be coded according to the Medical Dictionary for Regulatory Activities (MedDRA) version 23.0 or later and will be summarised by primary system organ class and preferred term by treatment regimen and overall. Medical histories of COVID-19 will not be included in this analysis. Additionally, a table containing only comorbidities pre-specified in the CRF will be displayed.

Physical examination findings will be listed.

7. Prior/ Concomitant Medications

Prior medications will be those that were taken prior to study entry and were stopped prior to the day of first study drug intake. Concomitant medications will be those that were given from the day of first study drug intake until study termination, irrespective of whether they started before the first study drug intake and continued into the study or whether they were newly started on or after first study drug intake. Prior and concomitant medications will be coded according to the latest release of the WHO drug dictionary in effect at the time of study start.

Prior and concomitant medications will be summarized separately by Anatomical / Therapeutical / Chemical (ATC) class 3 and preferred term.

All prior and concomitant medications will be listed by participant. These will include ATC classification and WHO preferred terms.

Prior and concomitant medication will be summarized for the Safety Analysis Set.

8. Efficacy Analysis

8.1 Primary variable(s)

The mITT Analysis Set will be used for the primary analysis.

The primary efficacy variable is incidence of SARS-CoV-2 clearance defined as the proportion of participants with a negative SARS-CoV-2 RT-PCR test on Day 7. The analysis will be performed using a log-binomial model. SARS-CoV-2 RT-PCR test on Day 7 (yes/no) will be the response variable. Treatment, age at baseline (years), sex, BMI at baseline, baseline comorbidities, baseline viral load category and days of symptoms at time of enrollment will be used as covariates. The analysis will be

repeated using a crude log-binomial regression model without any covariate adjustment. The main conclusions will be drawn from the adjusted analysis.

If the log-binomial model specified above does not converge a generalized linear regression with a log link, Poisson distribution, instead of Binomial distribution, will be used. If neither model converges a Normal distribution instead of a Poisson distribution will be used.

Estimates, two-sided 95% confidence intervals and corresponding p-values will be provided for the adjusted risk ratios that compare each experimental arm against the standard of care arm.

Participants who discontinue the study prior to the day 7 assessment of SARS-CoV-2 RT-PCR test because of an adverse event are counted as having a positive SARS-CoV-2 RT-PCR test on Day 7. Values from participants who discontinue the study prior to the day 7 assessment of SARS-CoV-2 RT-PCR test due to other reasons remain missing. Other participants with a missing Day 7 assessment will be imputed as positive. A time window from Day 5 to Day 9 will be used for the day 7 assessment.

In the first sensitivity analysis all participants who discontinue prior to the day 7 SARS-CoV-2 RT-PCR test are imputed as having a positive of SARS-CoV-2 RT-PCR test on Day 7. As a second sensitivity analysis the primary analysis will be repeated using the As Treated Analysis Set. Actual treatment received instead of randomized treatment will be utilized in the analysis.

Additionally, the number and percentage of participants with negative SARS-CoV-2 RT-PCR test at Day 7 will be displayed.

Subgroup analyses will be done for all covariates. The covariates age and days of symptoms at time of enrollment will be categorized according to the respective median of the covariate based on all patients in the mITT Analysis Set. The following subgroup analysis will be performed

- Age group at baseline (low: age (years) <= median, high: age (years) > median)
- Sex (male, female)
- BMI at baseline (low: BMI <= 30, high: BMI > 30)
- Baseline comorbidities (none, one or more)
- Baseline viral load category (low, high)
- Days of symptoms at time of enrollment (low: days of symptoms at time of enrollment <= median, high: days of symptoms at time of enrollment > median).
- Baseline risk category (low, high)

The primary regression model will be calculated for each subgroup variable by adding an interaction between the treatment arm and the respective subgroup variable. The p-value of the interaction will be displayed as well as the overall and the by-subgroup risk ratios comparing each experimental arm against the standard of care arm and the respective 95% CIs. In case the respective subgroup variable was defined by categorizing a continuous variable, the continuous variable will be replaced by the categorized version in the model.

8.2 Secondary and other variable(s)

All secondary endpoints will be assessed in the mITT Analysis Set. All analyses will be done by treatment arm, and overall if applicable.

8.2.1 Incidence of SARS-CoV-2 clearance

Analyses based on viral culture

The incidence of SARS-CoV-2 clearance on Day 7 (defined as the proportion of participants with negative SARS-CoV-2 viral cultures on both Day 7 and Day 10 in the subset of participants for whom these assessments are performed and results are available) will be analyzed using a log-binomial regression model in which the response variable will be SARS-CoV-2 clearance on Day 7 (yes/no). Treatment, age at baseline (years), sex, BMI at baseline, baseline comorbidities, baseline viral load category, and days of symptoms at time of enrollment will be used as covariates. Similar to the primary analysis, if the log-binomial model specified above does not converge a generalized linear regression with a log link, Poisson distribution, instead of binomial distribution, will be used. If neither model converges a normal distribution instead of a Poission distribution will be used.

Participants who discontinue the study prior to Day 7 will be excluded from the analysis. If a positive result is present either at Day 7 or Day 10 while the other assessment is missing, the outcome will be

classified as positive overall. If a negative result is present at either Day 7 or Day 10 while the other assessment is missing (or both or missing), the participant will be excluded from the analysis.

Additionally, the number and percentage of participants with SARS-CoV-2 clearance on Day 7 determined by viral culture will be displayed.

Only patients with positive viral culture at baseline are included in the analyses above.

Analyses based on SARS-CoV-2 RT-PCR test

The incidence of SARS-CoV-2 clearance (defined as the proportion of participants with negative of SARS-CoV-2 RT-PCR test) on Days 3, 10, 14, 21 and 28 will be analyzed similarly to the primary efficacy variable.

Separate log-binomial regressions will be used to analyze the categorical variable SARS-CoV-2 clearance (yes/no) at each time point. Treatment, age at baseline (years), sex, BMI at baseline, baseline comorbidities, baseline viral load category and days of symptoms at time of enrollment will be used as covariates. In case of non-convergence a poisson regression, similar to the primary analysis, will be used. If the Poisson regression does not converge, a Normal distribution will be used instead.

If the result at Day X (3, 10, 14 21, or 28) is missing then the result at Day X will be classified as positive if the test at the preceding visit was positive or if any subsequent test was positive. However, if the preceding test was negative and there were no subsequent positive tests, then the result will be classified as negative.

Additionally, the number and percentage of participants with negative of SARS-CoV-2 RT-PCR test will be displayed for Days 3, 10, 14, 21 and 28.

8.2.2 Time to clearance of SARS-CoV-2

Kaplan Meier analysis will be performed for the time to clearance of SARS-CoV-2, defined as the time to first negative qualitative SARS-CoV-2 RT-PCR test (collected post-baseline on Days 3, 7, 10, 14, 21 and 28) without any subsequent positive qualitative SARS-CoV-2 RT-PCR test.

Data from participants who do not experience the defined event will be censored as follows:

- Participants who withdraw from the study early due to any reason will be censored on the day of the last available of SARS-CoV-2 RT-PCR test measurement
- Participants who continue in the study but have all remaining SARS-CoV-2 RT-PCR tests missing from a given point in time onward will be censored on the day of the last available SARS-CoV-2 RT-PCR test measurement
- Participants without any post-baseline SARS-CoV-2 RT-PCR data will be censored at Day 1 (duration of 1 day)

Kaplan-Meier estimates and corresponding 2-sided 95% confidence intervals will be provided for time to clearance of SARS-CoV-2 until Day 7, Day 14, Day 21 and Day 28. The 25th, 50th and 75th percentiles of time to clearance and corresponding 2-sided 95% confidence intervals will be presented.

Kaplan-Meier curves for time to clearance of SARS-CoV-2 will be generated including the p-value of the log-rank test.

Time to first negative qualitative SARS-CoV-2 RT-PCR test without any subsequent positive qualitative SARS-CoV-2 RT-PCR test will also be analysed using Cox proportional hazards regression model. Covariates will be the same as in the regression model for the primary analysis. Hazard ratios, comparing each experimental arm against the standard of care arm, and the associated 95% confidence intervals will be presented.

The Kaplan Meier analysis and the Cox regression analysis will be repeated using quantitative RT-PCR based on the N gene instead of qualitative RT-PCR. The time to first quantitative SARS-CoV-2 RT-PCR test with zero count without any subsequent count above zero will be analysed.

The Kaplan-Meier analyses using quantitative RT-PCR will be repeated in the subset of participants with high viral load at baseline and high-risk participants respectively.

8.2.3 Viral load of SARS-CoV-2

Descriptive statistics for the log10 transformed viral load of SARS-CoV-2 detected by quantitative RT-PCR based on the N gene and corresponding change from baseline will be presented by time point

(Baseline, Day 3, Day 7, Day 10, Day 14). For the descriptive analysis and the mixed-effect linear model viral load values of 0 will be imputed with half of the lower limit of quantification (LLOQ) before log10 transformation. The lower limit of quantification is 20 copies/ml.

The covariate adjusted mean change from baseline for log10 transformed viral loads for each treatment arm will be obtained from a mixed-effect linear model for repeated measures (MMRM) with treatment, age at baseline (years), sex, BMI at baseline, baseline comorbidities, baseline viral load category, days of symptoms at time of enrollment and treatment-by-visit interaction as fixed effects. An unstructured covariance matrix will be assumed for the within subject repeated measurements, and Kenward-Rogers type degrees of freedom will be used. If the model fails to converge with unstructured covariance matrix, either a compound symmetry (first choice) or first order autoregressive (AR1) (second choice) covariance structure will be used.

The SAS procedure PROC MIXED will be used for analysis. Results will be presented with least-square mean (LSM) change, standard error (SE) and associated two-sided 95% confidence interval for treatment effects and LSM, SE, associated two-sided 95% confidence interval, and two-sided p value for treatment contrasts of each experimental arm versus the standard of care arm.

The model above will be repeated using the subset of high-risk participants.

A spaghetti plot which displays the individual log10 transformed viral loads of SARS-CoV-2 (detected by quantitative RT-PCR) of each participant from baseline until Day 28 by treatment arm will be presented.

8.2.4 Hospitalization

The response variable hospitalization up to day 28 (yes/no) will be analyzed using logistic regression. Treatment, age at baseline (years), sex, BMI at baseline, baseline comorbidities, baseline viral load category and days of symptoms at time of enrollment will be used as covariates. Estimates, two-sided 95% confidence intervals and corresponding p-values will be provided for the adjusted odds ratios that compare each experimental arm against the standard of care arm.

Time to first hospitalization (in days) will be analysed using Kaplan-Meier methodology.

Data from participants who do not experience the defined event will be censored as follows:

- Participants who withdraw from the study early due to any reason will be censored on the day of study termination.

Kaplan-Meier estimates and corresponding 2-sided 95% confidence intervals will be for Day 7, Day 14, Day 21 and Day 28. The 25th, 50th and 75th percentiles of time to first hospitalization and corresponding 2-sided 95% confidence intervals will be presented.

Kaplan-Meier curves for time to clearance of SARS-CoV-2 will be generated including the p-value of the log-rank test.

Time to first hospitalization will also be analysed using Cox proportional hazards regression model. Covariates will be the same as in the regression model for the primary analysis. Hazard ratios, comparing each experimental arm against the standard of care arm, and the associated 95% confidence intervals will be presented.

The cumulative incidence of hospitalization at Day 28, defined by the number of participants hospitalized up to and including Day 28 divided by the number of participants in the mITT Analysis Set, will be presented. The p-value of fisher's exact test will be provided for comparison of each experimental arm against the standard of care arm.

Number of days hospitalized will be described graphically using a bar chart and will be summarized using standard statistics for continuous data as well as interquartile range.

In general, only hospitalizations related to COVID-19 are analysed. Patients hospitalized within the first 24h after first study drug intake will be excluded from the analysis.

8.2.5 Disease severity

Disease severity as measured by the WHO Ordinal Scale for Clinical Improvement (Appendix 5) will be compared between the treatment arms using a longitudinal proportional odds model.

The response variable WHO Ordinal Scale score for Clinical Improvement measured at the post-baseline time points Day 7, Day 14, Day 21 and Day 28 is ordered from score 0 to 8 (0 < 1 < 2 < … < 8). Time point, treatment and treatment-by-time point interaction will be included as fixed-effect factors. Baseline age (in years), sex, baseline BMI, baseline WHO Ordinal Scale score, baseline comorbidities, baseline viral load category and days of symptoms at time of enrollment will be included as covariates. Participant-specific intercepts will be included as random effects. The estimated adjusted odds ratios comparing each experimental arm against the standard of care arm at each time point (Day 7, Day 14, Day 21 and Day 28) together with the associated two-sided 95% confidence intervals will be provided. The analysis will be performed with an assumption of missing at random (MAR) for missing data in the response variable. The proportional odds assumption will be investigated. If the assumption does not hold the results of the analysis have to be interpreted with caution.

If convergence problems arise due to the high number of categories of the response variable then a condensed response variable, patient state, will be used instead of the WHO Ordinal Scale score for Clinical Improvement. The score categories would be condensed as follows:

WHO Ordinal Scale score for Clinical Improvement	Patient state
0	0
1, 2	1
3, 4	2
5, 6, 7	3
8	4

If convergence problems persist for condensed response variables then patients will be classified into two categories (any post-baseline worsening by at least 1 category of the WHO ordinal scale score compared to baseline, no worsening of at least 1 category at any time point post-baseline compared to baseline). Using this classification, a logistic regression model, using the same covariates as in the primary analysis, will be calculated. Estimates, two-sided 95% confidence intervals and corresponding p-values will be provided for the adjusted odds ratios that compare each experimental arm against the standard of care arm.

Summary statistics for the maximum score on WHO Ordinal Scale for Clinical Improvement during study participation will be presented and results for each participant will be listed. The proportion of patients whose worst post-baseline WHO Ordinal Scale score improved, stayed equal or worsened compared to baseline will be provided.

Time to first zero WHO Ordinal Scale score (in days) defined as time to first zero score without any subsequent non-zero score will be analysed using Kaplan-Meier methodology. Participants with a WHO Ordinal Scale score of zero at baseline will be excluded from the analysis.

Data from participants who do not experience the defined event will be censored as follows:

- Participants who withdraw from the study early due to any reason will be censored on the day of last available WHO Ordinal Scale for Clinical Improvement assessment.
- Participants who continue in the study but have all remaining WHO Ordinal Scale for Clinical Improvement assessments missing from a given point in time onward will be censored on the day of the last available WHO Ordinal Scale for Clinical Improvement assessment.
- Participants without any post-baseline WHO Ordinal Scale for Clinical Improvement assessment data will be censored at Day 1 (duration of 1 day).

Kaplan-Meier estimates and corresponding 2-sided 95% confidence intervals will be for Day 7, Day 14, Day 21 and Day 28. The 25th, 50th and 75th percentiles of time to first zero score and corresponding 2-sided 95% confidence intervals will be presented.

Kaplan-Meier curves will be generated including the p-value of the log-rank test.

Time to first zero WHO Ordinal Scale Score (as defined above) will also be analysed using Cox proportional hazards regression model. Covariates will be the same as in the regression model for the primary analysis. Hazard ratios, comparing each experimental arm against the standard of care arm, and the associated 95% confidence intervals will be presented.

8.2.6 Symptom resolution

The number of days with fever after randomization, the number of days with respiratory symptoms (chest/respiratory FLU-PRO domain score >0) after randomization, and the number of days with Sp02<93% after randomization will be modeled using Poisson regression with an offset for number of days of observation (i.e. number of days with corresponding daily vitals/chest/respiratory FLU-PRO domain score data non-missing after randomization). Treatment, baseline viral load category and baseline comorbidities will be included as factor, age at baseline (years), sex, BMI at baseline and days of symptoms at time of enrollment will be included as covariates. Estimates of incidence rates (number of events per 100 person-days) and corresponding two-sided 95% confidence intervals will be presented for each experimental arm and the standard of care arm. Additionally, the incidence rate ratios (IRR) and corresponding two-sided 95% confidence intervals will be provided that compare each experimental arm against the standard of care arm.

The endpoint relating to SpO2 will be calculated for all participants that had SpO2 ≥95% at baseline. SpO2 assessments <93% are only counted if they are confirmed by a repeat assessment which must be between 1 and 12 hours after the initial SpO2 reading. The analysis for SpO2 will be done once only for plausible SpO2 values and once for all available SpO2 values. Implausible SpO2 values are defined as values below 87% with no associated difficulty in breathing or chest tightness (as determined by medical review of FLU-PRO and other eCRF data).

The FLU-PRO© Plus questionnaire will be measured daily during the first 14 days, at Day 21 and at Day 28. The questionnaire is composed of 32 items. From these items six domain scores for the body areas Nose, Throat, Eyes, Chest/Respiratory, Gastrointestinal, and Body/Systemic can be computed ranging from 0 (symptom free) to 4 (very severe symptoms). A total score is calculated as well. The domain scores and the total score will be calculated for each time point. The derivation rules for the domain score and the total score can be found in Section 17.1. The domain scores and the total score and their corresponding changes from baseline will be displayed by time point with the number of observations, mean, standard deviation, median, minimum and maximum, and quartiles.

Both the FLU-PRO© Plus questionnaire and the FLU-PRO© Plus Global Additional Daily Diary Items, including daily survey items not related to vital signs, will be listed for each participant.

8.2.7 Lower respiratory tract infection

Lower respiratory tract infection (LRTI) is defined by resting SpO2<93% sustained for 2 readings 2 hours apart and presence of either subjective dyspnea or cough (or both). The repeat SpO2 assessment must be between 1 and 12 hours after the initial SpO2 reading. In order to identify the LRTIs all possible cases are first determined programmatically using only the SpO2 readings. Medical review will then review these possible cases in conjunction with clinical symptoms and decide whether the LRTI definition is fulfilled or not.

The cumulative incidence of LRTI at Day 28, defined by the number of participants developing LRTI up to and including Day 28 divided by the number of participants in the mITT Analysis Set that had SpO2 ≥95% at baseline, will be presented. Corresponding two-sided 95% CIs, calculated according to the Wilson method without continuity correction, will be displayed.

The above analysis will be done once only for plausible SpO2 values and once for all available SpO2 values. Plausible values are defined in Section 8.2.6.

8.2.8 All-cause Mortality

The cumulative incidence of all-cause mortality at Day 28 defined by the number of participants who died up to and including Day 28 divided by the number of participants in the mITT Analysis Set will be presented. Participants who are hospitalized at the time of Day 28 and die later also count to the numerator. Corresponding two-sided 95% CIs, calculated according to the Wilson method without continuity correction, will be displayed.

9. Exposure to Study Drug

The study drug compliance will be presented using standard summary statistics for each experimental treatment arm for the Safety Analysis Set. Additionally, compliance will be categorized using <85% and >=85% and the corresponding number and percentage will be displayed. The compliance will only be calculated and displayed for the experimental treatments, as Paracetamol follows no clear dosing regimen and is taken as needed to the maximum allowed daily dose.

Compliance is calculated by using the total number of tablets actually taken divided by the total scheduled number of tablets based on the participant's randomized treatment arm multiplied with 100. The total number of tablets actually taken is derived as the total number of tablets dispensed minus the total number of tablets returned. The total scheduled number of tablets for each experimental treatment arm can be found in the table 1 below. For experimental arm D the compliance for NTZ is calculated as detailed above. For FPV the compliance is calculated as the total dose actually taken divided by the total planned dose multiplied with 100. The total dose actually taken is derived as the sum of the total number of tablets actually taken (see derivation above) multiplied with corresponding dosage formulation (200 mg or 400 mg). The total planned dose for FPV is 10400 mg. The compliance for each drug is presented separately.

Table 1

Treatment arm	Study drug	Total daily number of scheduled tablets
Arm B	ASAQ	6
Arm C	PA	9 (body weight >=45 kg - < 65 kg)<br>12 (body weight >= 65 kg)
Arm D	FPV	Based on total planned dose (10400 mg).
	NTZ	28
Arm E	SOF/DCV	7

Details on the administration of the investigational drug, including number of tablets dispensed, number of tablets returned, date, time and compliance of dosing will be listed by participant. The listing will be done for the Safety Analysis Set.

PK analyses with respect to exposure to study drug are detailed in section 11.

10. Safety Analysis

All safety analyses will be performed on the Safety Analysis Set by treatment arm and overall.

10.1 Adverse events

All treatment emergent adverse events (TEAEs), i.e., adverse events (AEs) which newly started or increased in intensity on or after the day of first study drug administration will be considered for summarization. Adverse events will be coded according to the Medical Dictionary for Regulatory Activities (MedDRA) version 23.0 or later. The severity grading of AEs will be done according to the DAIDS AE Grading Table, corrected Version 2.1, July 2017.

TEAEs will be summarized by presenting the number and percentage of participants having at least one TEAE, having a TEAE in each primary system organ class and having each individual TEAE (preferred term).

The number and percentage of participants and the numbers of events will be presented by primary system organ class and preferred term for the following summaries:

- TEAEs.
- Serious TEAEs.
- Study drug related TEAEs/serious TEAEs.
- TEAEs/serious TEAEs leading to study drug discontinuation.
- TEAEs leading to death.
- TEAEs leading to hospitalization.

Additionally, TEAEs will be summarized with number and percentage of participants and the numbers of events by primary system organ class, preferred term and maximum severity. For summaries by maximum severity the following applies: if a participant reported more than one adverse event with the same preferred term, the adverse event with the greatest severity will be presented. If a participant reported more than one adverse event within the same primary system organ class, the participant will be counted only once with the greatest severity at the system organ class level.

For the legal requirements of ClinicalTrials.gov a summary of treatment emergent adverse events which are not serious adverse events with an incidence greater than 5% in at least one treatment arm will be provided by primary system organ class and preferred term on the Safety Analysis Set.

All information obtained on AEs will be displayed in appropriate data listings by treatment arm and participant.

10.2 Vital signs

Absolute values and changes from baseline in vital signs (temperature (°C), pulse (beats/min), respiratory rate (breaths/min) and SpO2 (%)) will be summarized by time point with the number of observations, mean, standard deviation, median, minimum and maximum, and quartiles.

11. Exploratory Analyses

All exploratory analyses relating to PK will be performed using the PK Analysis Set. All other exploratory analyses will be performed using the mITT Analysis Set.

Drug exposure

Plasma/whole blood $C_{day\ 3}$ and $C_{day\ 7}$ concentrations on Day 3 (Arms B and C) and Day 7 (Arms B to E) will be displayed by treatment arm for each component using summary statistics. Concentrations below the lower limit of quantification (LLOQ) will be imputed with LLOQ/2.

Relationship between efficacy and plasma or whole blood concentrations of the study drugs

Plasma or blood concentrations of each component at Day 3 ($C_{day\ 3}$, for ASAQ and PA arms) and/or Day 7 ($C_{day\ 7}$, for all participants in the experimental arms), will be displayed using a scatter plot. Participants will be displayed by SARS-CoV-2 clearance on Day 7 (as defined for the primary endpoint) and exploratory arm. Concentrations below the lower limit of quantification (LLOQ) will be imputed with LLOQ/2.

Immune response

Seroconversion as assessed by means of a validated serological assay will be a qualitative outcome (positive, negative). A shift table comparing baseline and Day 28 results will be displayed.

Relationship between sample RT-PCR results, their cycle threshold values, and corresponding viral culture results for SARS-CoV-2

The proportion of participants with low viral load (log10 viral load < 6) on Day 7 will be analyzed similarly to the primary analysis using a log-binomial regression adjusted for the same covariates. If the log-binomial model specified above does not converge a generalized linear regression with a log link, poisson distribution, instead of binomial distribution, will be used. If neither model converges a Normal distribution instead of a Poisson distribution will be used.

Estimates, two-sided 95% confidence intervals and corresponding p-values will be provided for the adjusted risk ratios that compare each experimental arm against the standard of care arm.

A shift table of the baseline qualitative SARS-CoV-2 RT-PCR results versus each post-baseline time point by treatment arm will be generated.

Additionally, corresponding viral culture results from screening, Day 7 and Day 10 in the subset of participants for whom viral culture is performed will be displayed.

Descriptive statistics for the log10 transformed mean viral load of SARS-CoV-2 and corresponding change from baseline will be presented by time point (Baseline, Day 3, Day 7, Day 10, Day 14, Day 21, Day 28). The mean viral load result will be calculated as the mean viral load result detected by quantitative RT-PCR of the S-gene, N-gene and ORF1ab gene. Only results based on nasal-swabs will be used. Mean viral load values of 0 will be imputed with half of the lower limit of quantification (LLOQ) before log10 transformation, i.e. the imputed value will be 10 copies/ml, before log10 transformation.

The mixed-effect linear model for repeated measures described in section 8.2.3 will be repeated using the above derivation of log10 transformed mean viral load.

The incidence of SARS-CoV-2 clearance on Day 10 (defined as the proportion of participants with negative SARS-CoV-2 viral cultures on Day 10 in the subset of participants for whom this assessment is performed and results are available) will be analyzed using a log-binomial regression model in which the response variable will be SARS-CoV-2 clearance on Day 10 (yes/no). Treatment, age at baseline (years), sex, BMI at baseline, baseline comorbidities, baseline viral load category, and days of symptoms at time of enrollment will be used as covariates. Similar to the primary analysis, if the log-binomial model specified above does not converge a generalized linear regression with a log link, Poisson distribution, instead of Binomial distribution, will be used. If neither model converges a Normal distribution instead of a Poission distribution will be used.

Participants who discontinue the study prior to Day 10 will be excluded from the analysis. If a result is missing at Day 10 the participant will be excluded from the analysis.

Only patients with positive viral culture at baseline are included in the analyses above.

12. Interim Analyses

No formal interim analyses for futility will be performed.

An independent DMC will be convened for this study after approximately 75 and 150 patients have reached Day 7 with expertise in respiratory viruses, antiviral therapies and shedding, emerging epidemics and medical statistics. Details regarding the objectives and responsibilities of the DMC, and the DMC operational procedures are described in the DMC Charter.

There may be a potential sample size adjustment based on the dropout rate after approximately 150 participants have been recruited and have safety and viral clearance data for Day 7. The sample size adjustment will be performed by the trial statistician and verified by the DMC statistician. The details of the sample size adjustment are described in the Interim Monitoring Plan.

Processing of data and reports to be generated for the DMC, except for the sample size adjustment, are not described in the SAP.

13. Change to Protocol Specified Analyses

Due to the now well-described varying sensitivity of SARS-CoV-2 RT-PCR assays and the high incidence of false negative results, the analysis of time to clearance of SARS-CoV-2 will only consider viral clearance to be present if all subsequent RT-PCR results remain negative.

For the subgroups of sero-negative participants and for high-risk participants, the following post-hoc analyses were added:

- Summary of WHO Ordinal Scale for Clinical Improvement
- Kaplan Meier analysis of time to first zero WHO Ordinal Scale score for Clinical Improvement
- Cox regression analysis of time to first zero WHO Ordinal Scale score for Clinical Improvement.

14. List of Tables and Figures

Table 14.1.1 Participant disposition by treatment arm and overall (Randomized Analysis Set)........... 24
Table 14.1.2 Analysis sets by treatment arm and overall (Randomized Analysis Set)......................... 25
Table 14.1.3 Summary of participants excluded from analysis sets by treatment arm and overall (Randomized Analysis Set) 26
Table 14.1.4 Demographic characteristics by treatment arm and overall (mITT Analysis Set) 27
Table 14.1.5 Baseline characteristics by treatment arm and overall (mITT Analysis Set).................... 31
Table 14.1.6 Medical and surgical history by treatment arm and overall, MedDRA primary system organ class and preferred term (mITT Analysis Set) 34
Table 14.1.7 Comorbidities of interest for SARS-CoV-2 infection by treatment arm and overall, MedDRA primary system organ class and preferred term (mITT Analysis Set) 35
Table 14.1.8 Prior medications by ATC class and WHO preferred term by treatment arm and overall, ATC class and preferred term (Safety Analysis Set)........ 36
Table 14.2.1.1 Primary analysis of incidence of SARS-CoV-2 clearance on Day 7 based on RT-PCR (mITT Analysis Set)........ 37
Table 14.2.1.2 Sensitivity analysis #1 for incidence of SARS-CoV-2 clearance on Day 7 based on RT-PCR (mITT Analysis set)........ 37
Table 14.2.1.3 Sensitivity analysis #2 for incidence of SARS-CoV-2 clearance on Day 7 based on RT-PCR (As Treated Analysis set)........ 37
Table 14.2.1.4 Primary analysis of incidence of SARS-CoV-2 clearance on Day 7 based on RT-PCR by subgroup (mITT Analysis Set) 38
Table 14.2.1.5 Incidence of SARS-CoV-2 clearance on Day 7 based on viral culture (mITT Analysis Set) 39
Table 14.2.1.6 Incidence of SARS-CoV-2 clearance on Day 3, 10, 14, 21 and 28 based on RT-PCR (mITT Analysis Set)........ 40
Table 14.2.2.1 Kaplan Meier analysis of time to SARS-CoV-2 clearance based on qualitative RT-PCR (mITT Analysis Set)........ 41
Table 14.2.2.2 Kaplan Meier analysis of time to SARS-CoV-2 clearance based on quantitative RT-PCR (mITT Analysis Set) 43
Table 14.2.2.3 Kaplan Meier analysis of time to SARS-CoV-2 clearance based on quantitative RT-PCR (mITT Analysis Set - high-risk participants)........ 44
Table 14.2.2.4 Kaplan Meier analysis of time to SARS-CoV-2 clearance based on quantitative RT-PCR (mITT Analysis Set – high baseline viral load) 44
Table 14.2.2.5 Cox regression analysis of time to SARS-CoV-2 clearance based on qualitative RT-PCR (mITT Analysis Set) 45
Table 14.2.2.6 Cox regression analysis of time to SARS-CoV-2 clearance based on quantitative RT-PCR (mITT Analysis Set) 45
Table 14.2.2.7 Cox regression analysis of time to SARS-CoV-2 clearance based on quantitative RT-PCR (mITT Analysis Set – high-risk participants) 46
Table 14.2.2.8 Cox regression analysis of time to SARS-CoV-2 clearance based on quantitative RT-PCR (mITT Analysis Set – high baseline viral load) 46
Figure 14.2.2.1 Kaplan-Meier curves for time to SARS-CoV-2 clearance based on qualitative RT-PCR (m-ITT Analysis Set)........ 47
Figure 14.2.2.2 Kaplan-Meier curves for time to SARS-CoV-2 clearance based on quantitative RT-PCR (m-ITT Analysis Set) 48
Figure 14.2.2.3 Kaplan-Meier curves for time to SARS-CoV-2 clearance based on quantitative RT-PCR (m-ITT Analysis Set – high-risk participants)........ 48
Figure 14.2.2.4 Kaplan-Meier curves for time to SARS-CoV-2 clearance based on quantitative RT-PCR (m-ITT Analysis Set – high baseline viral load) 48
Table 14.2.3.1 Summary of log10 viral load of SARS-CoV-2 by treatment arm and overall and time point (mITT Analysis Set) 49

Table 14.2.3.2 Repeated measure analysis of log10 viral load of SARS-CoV-2 change from baseline (mITT Analysis Set) 51
Table 14.2.3.3 Repeated measure analysis of log10 viral load of SARS-CoV-2 change from baseline (mITT Analysis Set – high-risk participants) 51
Figure 14.2.3.1 Spaghetti plot of log10 viral load of SARS-CoV-2 based quantitative RT-PCR by treatment arm (mITT Analysis Set) 52
Table 14.2.4.1 Logistic regression for hospitalization up to Day 28 (mITT Analysis Set) 53
Table 14.2.4.2 Summary of hospitalization data by treatment arm and overall (mITT Analysis Set) ... 54
Table 14.2.4.3 Kaplan Meier analysis of time to first hospitalization (mITT Analysis Set) 55
Table 14.2.4.4 Cox regression analysis of time to first hospitalization (mITT Analysis Set) 56
Figure 14.2.4.1 Kaplan-Meier curves for time to first hospitalization (mITT Analysis Set) 56
Table 14.2.5.1 Summary of WHO Ordinal Scale for Clinical Improvement by treatment arm and overall (mITT Analysis Set) 57
Table 14.2.5.2 Proportional odds model for disease severity for Day 7, 14, 21, and 28 (mITT Analysis Set) 58
Table 14.2.5.3 Logistic regression for disease severity (mITT Analysis Set) 59
Table 14.2.5.4 Kaplan Meier analysis of time to first zero WHO Ordinal Scale score for Clinical Improvement (mITT Analysis Set) 60
Table 14.2.5.5 Cox regression analysis of time to first zero WHO Ordinal Scale score for Clinical Improvement (mITT Analysis Set) 61
Table 14.2.5.6 Summary of WHO Ordinal Scale for Clinical Improvement by treatment arm and overall (mITT Analysis Set – sero-negative participants) 61
Table 14.2.5.7 Summary of WHO Ordinal Scale for Clinical Improvement by treatment arm and overall (mITT Analysis Set – high-risk participants) 61
Table 14.2.5.8 Kaplan Meier analysis of time to first zero WHO Ordinal Scale score for Clinical Improvement (mITT Analysis Set – sero-negative participants) 61
Table 14.2.5.9 Kaplan Meier analysis of time to first zero WHO Ordinal Scale score for Clinical Improvement (mITT Analysis Set – high-risk participants) 62
Table 14.2.5.10 Cox regression analysis of time to first zero WHO Ordinal Scale score for Clinical Improvement (mITT Analysis Set – sero-negative participants) 62
Table 14.2.5.11 Cox regression analysis of time to first zero WHO Ordinal Scale score for Clinical Improvement (mITT Analysis Set – high-risk participants) 62
Figure 14.2.5.1 Kaplan-Meier curves for time to first zero WHO Ordinal Scale score for Clinical Improvement (mITT Analysis set) 62
Table 14.2.6.1 Poisson regression for proportion of days with fever after randomization (mITT Analysis Set) 63
Table 14.2.6.2 Poisson regression for proportion of days with respiratory symptoms after randomization (mITT Analysis Set) 64
Table 14.2.6.3 Poisson regression for proportion of days with SpO2<93% after randomization (mITT Analysis Set) 65
Table 14.2.6.4 Summary of FLU-PRO Plus questionnaire scores and changes from baseline by treatment arm and overall (mITT Analysis Set) 66
Table 14.2.7.1 Cumulative incidence of lower respiratory tract infection by treatment arm and overall (mITT Analysis Set) 68
Table 14.2.8.1 Cumulative incidence of all-cause mortality by treatment arm and overall (mITT Analysis Set) 69
Table 14.3.1.1 Compliance by treatment arm (Safety Analysis Set) 70
Table 14.3.2.1 Concomitant medications by treatment arm and overall, ATC class and WHO preferred term (Safety Analysis Set) 71
Table 14.3.3.1 Summary of treatment emergent adverse events by MedDRA primary system organ class and preferred term by treatment arm and overall (Safety Analysis Set) 72

Table 14.3.3.2 Summary of serious treatment emergent adverse events by MedDRA primary system organ class and preferred term by treatment arm and overall (Safety Analysis Set) 73
Table 14.3.3.3 Summary of treatment emergent adverse events considered to be study drug related by MedDRA primary system organ class and preferred term by treatment arm and overall (Safety Analysis Set) 73
Table 14.3.3.4 Summary of serious treatment emergent adverse events considered to be study drug related by MedDRA primary system organ class and preferred term by treatment arm and overall (Safety Analysis Set) 73
Table 14.3.3.5 Summary of treatment emergent adverse events leading to study drug discontinuation by MedDRA primary system organ class and preferred term by treatment arm and overall (Safety Analysis Set) 73
Table 14.3.3.6 Summary of serious treatment emergent adverse events leading to study drug discontinuation by MedDRA primary system organ class and preferred term by treatment arm and overall (Safety Analysis Set) 73
Table 14.3.3.7 Summary of treatment emergent adverse events leading to death by MedDRA primary system organ class and preferred term by treatment arm and overall (Safety Analysis Set) 73
Table 14.3.3.8 Summary of treatment emergent adverse events leading to hospitalization by MedDRA primary system organ class and preferred term by treatment arm and overall (Safety Analysis Set) .. 73
Table 14.3.3.9 Summary of treatment emergent adverse events by MedDRA primary system organ class, preferred term, maximal severity and treatment arm and overall (Safety Analysis Set) 74
Table 14.3.3.10 Non-serious adverse events (threshold >5%) by MedDRA primary system organ class, preferred term and treatment arm (Safety Analysis Set) 75
Table 14.3.4.1 Summary of vital signs including changes from baseline by treatment arm and overall and time point (Safety Analysis Set) 76
Table 14.3.5.1 Shift table of serology by treatment arm (mITT Analysis Set) 78
Table 14.3.5.2 Summary of PK parameter by treatment arm and overall (PK Analysis Set) 79
Table 14.3.5.3 Incidence of low viral load on Day 7 (mITT Analysis Set) 80
Table 14.3.5.4 Shift table of viral culture data by treatment arm, overall and time point (mITT Analysis Set) 81
Table 14.3.5.5 Shift table of qualitative SARS-CoV-2 RT-PCR by treatment arm (mITT Analysis Set) 82
Table 14.3.5.6 Summary of log10 mean viral load of SARS-CoV-2 by treatment arm and overall, time point (mITT Analysis Set) 83
Table 14.3.5.7 Repeated measure analysis of log10 mean viral load of SARS-CoV-2 change from baseline (mITT Analysis Set) 83
Table 14.3.5.8 Incidence of SARS-CoV-2 clearance on Day 10 based on viral culture (mITT Analysis Set) 83
Figure 14.3.5.1 Scatter plot of plasma or blood concentrations at Day 3 and/or Day 7 by SARS-CoV-2 clearance on Day 7 and exploratory arm (PK Analysis Set) 84
Listing 14.3.1.1 Listing of adverse events leading to death (Safety Analysis Set) 85
Listing 14.3.1.2 Listing of serious treatment-emergent adverse events (Safety Analysis Set) 85
Listing 14.3.1.3 Listing of treatment-emergent adverse events leading to study discontinuation (Safety Analysis Set) 85
Listing 14.3.1.4 Listing of treatment-emergent adverse events with action taken related to study treatment (Safety Analysis Set) 85

15. List of Data Listings and Statistical Tabulations

Appendix 16.2.1.1 Study completion (Randomized Analysis Set) 86
Appendix 16.2.1.2 Screening failures (All screened subjects) 87
Appendix 16.2.2.1 Protocol deviation (Randomized Analysis Set) 88
Appendix 16.2.3.1 Analysis Sets (Randomized Analysis Set) 89

Appendix 16.2.4.1 Demographic data and informed consent (Randomized Analysis Set) 90
Appendix 16.2.4.2 Medical and surgical history (Randomized Analysis Set) 91
Appendix 16.2.4.3 Physical examination (Randomized Analysis Set)... 92
Appendix 16.2.4.4 Randomization (Randomized Analysis Set)... 93
Appendix 16.2.5.1 Drug accountability (Safety Analysis Set) 94
Appendix 16.2.5.2 Pharmacokinetic concentration data (PK Analysis Set)... 95
Appendix 16.2.6.1 Mid-nasal swab and saliva specimen data, viral culture and SARS-CoV-2 serology (Randomized Analysis Set) 96
Appendix 16.2.6.2 Hospitalizations (Randomized Analysis Set).. 97
Appendix 16.2.6.3 FLU-PRO Plus (Randomized Analysis Set) 98
Appendix 16.2.6.4 FLU-PRO Plus Global additional daily diary items (Randomized Analysis Set)..... 99
Appendix 16.2.6.5 WHO Ordinal Scale for Clinical Improvement (Randomized Analysis Set) 100
Appendix 16.2.7.1 Treatment emergent adverse events (Safety Analysis Set).................................. 101
Appendix 16.2.7.2 Vital signs (Safety Analysis Set) 102
Appendix 16.2.7.3 Prior and concomitant medications (Safety Analysis Set) 103

16. Output Shells

Table 14.1.1 Participant disposition by treatment arm and overall (Randomized Analysis Set)
(Page x of y)

	SOC (N=xx) n (%)	ASAQ (N=xx) n (%)	PA (N=xx) n (%)	FPV-NTZ (N=xx) n (%)	SOF/DCV (N=xx) n (%)	Overall (N=xx) n (%)
Participants randomized	xx (100.0)	xx (100.0)	xx (100.0)	xx (100.0)	xx (100.0)	xx (100.0)
Participants randomized but not treated	xx (xx.x)	xx (xx.x)	xx (xx.x)	xx (xx.x)	xx (xx.x)	xx (xx.x)
Primary reason for discontinuation						
Reason 1	xx (xx.x)	xx (xx.x)	xx (xx.x)	xx (xx.x)	xx (xx.x)	xx (xx.x)
Reason 2	xx (xx.x)	xx (xx.x)	xx (xx.x)	xx (xx.x)	xx (xx.x)	xx (xx.x)
...						
Participants treated	xx (xx.x)	xx (xx.x)	xx (xx.x)	xx (xx.x)	xx (xx.x)	xx (xx.x)
Participants treated but discontinued prior to study end						
Primary reason for discontinuation						
Reason 1	xx (xx.x)	xx (xx.x)	xx (xx.x)	xx (xx.x)	xx (xx.x)	xx (xx.x)
Reason 2	xx (xx.x)	xx (xx.x)	xx (xx.x)	xx (xx.x)	xx (xx.x)	xx (xx.x)
...						
Participants completed	xx (xx.x)	xx (xx.x)	xx (xx.x)	xx (xx.x)	xx (xx.x)	xx (xx.x)

Percentages are based on the number participants in the Randomized Analysis Set.

Programming note: Display only reasons for discontinuation with non-zero counts.

Table 14.1.2 Analysis sets by treatment arm and overall (Randomized Analysis Set)
(Page x of y)

	SOC<br>n (%)	ASAQ<br>n (%)	PA<br>n (%)	FPV-NTZ<br>n (%)	SOF/DCV<br>n (%)	Overall<br>n (%)
Randomized Analysis Set	xx (100.0)	xx (100.0)	xx (100.0)	xx (100.0)	xx (100.0)	xx (100.0)
Safety Analysis Set	xx (xx.x)	xx (xx.x)	xx (xx.x)	xx (xx.x)	xx (xx.x)	xx (xx.x)
mITT Analysis Set	xx (xx.x)	xx (xx.x)	xx (xx.x)	xx (xx.x)	xx (xx.x)	xx (xx.x)
As Treated Analysis Set	xx (xx.x)	xx (xx.x)	xx (xx.x)	xx (xx.x)	xx (xx.x)	xx (xx.x)
PK Analysis Set	xx (xx.x)	xx (xx.x)	xx (xx.x)	xx (xx.x)	xx (xx.x)	xx (xx.x)

Percentages are based on the number of participants in the Randomized Analysis Set.

Table 14.1.3 Summary of participants excluded from analysis sets by treatment arm and overall (Randomized Analysis Set)
(Page x of y)

	SOC<br>(N=xx)<br>n (%)	ASAQ<br>(N=xx)<br>n (%)	PA<br>(N=xx)<br>n (%)	FPV-NTZ<br>(N=xx)<br>n (%)	SOF/DCV<br>(N=xx)<br>n (%)	Overall<br>(N=xx)<br>n (%)
Excluded from the Safety Analysis Set (*)	xxx (xx.x)	xxx (xx.x)	xxx (xx.x)	xxx (xx.x)	xxx (xx.x)	xxx (xx.x)
Excluded from the mITT Analysis Set (**)	xxx (xx.x)	xxx (xx.x)	xxx (xx.x)	xxx (xx.x)	xxx (xx.x)	xxx (xx.x)
Excluded from the As Treated Analysis Set	xxx (xx.x)	xxx (xx.x)	xxx (xx.x)	xxx (xx.x)	xxx (xx.x)	xxx (xx.x)
Reasons for exclusion (***)						
Reason 1	xxx (xx.x)	xxx (xx.x)	xxx (xx.x)	xxx (xx.x)	xxx (xx.x)	xxx (xx.x)
Reason 2	xxx (xx.x)	xxx (xx.x)	xxx (xx.x)	xxx (xx.x)	xxx (xx.x)	xxx (xx.x)
Reason 3	xxx (xx.x)	xxx (xx.x)	xxx (xx.x)	xxx (xx.x)	xxx (xx.x)	xxx (xx.x)
Participants with more than one reason	xxx (xx.x)	xxx (xx.x)	xxx (xx.x)	xxx (xx.x)	xxx (xx.x)	xxx (xx.x)
Excluded from the PK Analysis Set (****)	xxx (xx.x)	xxx (xx.x)	xxx (xx.x)	xxx (xx.x)	xxx (xx.x)	xxx (xx.x)

Percentages are based on the number of participants in the Randomized Analysis Set.
(*) Enrolled, randomized participants who did not receive study drug.
(**) Enrolled, randomized participants who did not receive study drug or did not have confirmed SARS-CoV-2 infection at baseline.
(***) Reasons are not mutually exclusive.
(****) Enrolled, randomized participants who received study drug who had no PK data.

Programming note: Sort reasons by descending frequency in the total column.

Table 14.1.4 Demographic characteristics by treatment arm and overall (mITT Analysis Set)
(Page x of y)

Variable/<br>Statistic/Category	SOC<br>(N=xx)	ASAQ<br>(N=xx)	PA<br>(N=xx)	FPV-NTZ<br>(N=xx)	SOF/DCV<br>(N=xx)	Overall<br>(N=xx)
Sex, n (%)						
Male	xx (xx.x)	xx (xx.x)	xx (xx.x)	xx (xx.x)	xx (xx.x)	xx (xx.x)
Female	xx (xx.x)	xx (xx.x)	xx (xx.x)	xx (xx.x)	xx (xx.x)	xx (xx.x)
Race						
Black African	xx (xx.x)	xx (xx.x)	xx (xx.x)	xx (xx.x)	xx (xx.x)	xx (xx.x)
Coloured	xx (xx.x)	xx (xx.x)	xx (xx.x)	xx (xx.x)	xx (xx.x)	xx (xx.x)
White	xx (xx.x)	xx (xx.x)	xx (xx.x)	xx (xx.x)	xx (xx.x)	xx (xx.x)
Asian or Indian	xx (xx.x)	xx (xx.x)	xx (xx.x)	xx (xx.x)	xx (xx.x)	xx (xx.x)
Other	xx (xx.x)	xx (xx.x)	xx (xx.x)	xx (xx.x)	xx (xx.x)	xx (xx.x)
Age (years)						
n	xx	xx	xx	xx	xx	xx
Mean	xx.x	xx.x	xx.x	xx.x	xx.x	xx.x
Standard deviation	x.xx	x.xx	x.xx	x.xx	x.xx	x.xx
Minimum	xx	xx	xx	xx	xx	xx
Q1	xx.x	xx.x	xx.x	xx.x	xx.x	xx.x
Median	xx.x	xx	xx	xx	xx	xx
Q3	xx.x	xx.x	xx.x	xx.x	xx.x	xx.x
Maximum	xx	xx	xx	xx	xx	xx

In case of missing values for categorical variables a missing category will be displayed.
Body mass index = weight in kg / (height in m)**2

Table 14.1.4 Demographic characteristics by treatment arm and overall (mITT Analysis Set)
(Page x of y)

Variable/ Statistic/Category	SOC (N=xx)	ASAQ (N=xx)	PA (N=xx)	FPV-NTZ (N=xx)	SOF/DCV (N=xx)	Overall (N=xx)
Age (years) by sex, n (%)						
Male						
n	xx	xx	xx	xx	xx	xx
Mean	xx.x	xx.x	xx.x	xx.x	xx.x	xx.x
Standard deviation	x.xx	x.xx	x.xx	x.xx	x.xx	x.xx
Minimum	xx	xx	xx	xx	xx	xx
Q1	xx.x	xx.x	xx.x	xx.x	xx.x	xx.x
Median	xx.x	xx	xx	xx	xx	xx
Q3	xx.x	xx.x	xx.x	xx.x	xx.x	xx.x
Maximum	xx	xx	xx	xx	xx	xx
Female						
n	xx	xx	xx	xx	xx	xx
Mean	xx.x	xx.x	xx.x	xx.x	xx.x	xx.x
Standard deviation	x.xx	x.xx	x.xx	x.xx	x.xx	x.xx
Minimum	xx	xx	xx	xx	xx	xx
Q1	xx.x	xx.x	xx.x	xx.x	xx.x	xx.x
Median	xx.x	xx	xx	xx	xx	xx
Q3	xx.x	xx.x	xx.x	xx.x	xx.x	xx.x
Maximum	xx	xx	xx	xx	xx	xx

In case of missing values for categorical variables a missing category will be displayed.
Body mass index = weight in kg / (height in m)**2

Table 14.1.4 Demographic characteristics by treatment arm and overall (mITT Analysis Set)
(Page x of y)

Variable/ Statistic/Category	SOC (N=xx)	ASAQ (N=xx)	PA (N=xx)	FPV-NTZ (N=xx)	SOF/DCV (N=xx)	Overall (N=xx)
Body weight (kg)						
n	xx	xx	xx	xx	xx	xx
Mean	xx.x	xx.x	xx.x	xx.x	xx.x	xx.x
Standard deviation	x.xx	x.xx	x.xx	x.xx	x.xx	x.xx
Minimum	xx	xx	xx	xx	xx	xx
Q1	xx.x	xx.x	xx.x	xx.x	xx.x	xx.x
Median	xx.x	xx	xx	xx	xx	xx
Q3	xx.x	xx.x	xx.x	xx.x	xx.x	xx.x
Maximum	xx	xx	xx	xx	xx	xx
Height (cm)						
n	xx	xx	xx	xx	xx	xx
Mean	xx.x	xx.x	xx.x	xx.x	xx.x	xx.x
Standard deviation	x.xx	x.xx	x.xx	x.xx	x.xx	x.xx
Minimum	xx	xx	xx	xx	xx	xx
Q1	xx.x	xx.x	xx.x	xx.x	xx.x	xx.x
Median	xx.x	xx	xx	xx	xx	xx
Q3	xx.x	xx.x	xx.x	xx.x	xx.x	xx.x
Maximum	xx	xx	xx	xx	xx	xx
BMI (kg/m**2)						
n	xx	xx	xx	xx	xx	xx
Mean	xx.x	xx.x	xx.x	xx.x	xx.x	xx.x
Standard deviation	x.xx	x.xx	x.xx	x.xx	x.xx	x.xx
Minimum	xx	xx	xx	xx	xx	xx
Q1	xx.x	xx.x	xx.x	xx.x	xx.x	xx.x
Median	xx.x	xx	xx	xx	xx	xx
Q3	xx.x	xx.x	xx.x	xx.x	xx.x	xx.x
Maximum	xx	xx	xx	xx	xx	xx

```
In case of missing values for categorical variables a missing category will be displayed.
Body mass index = weight in kg / (height in m)**2
```

Table 14.1.5 Baseline characteristics by treatment arm and overall (mITT Analysis Set)
(Page x of y)

Variable/ Statistic/Category	SOC (N=xx)	ASAQ (N=xx)	PA (N=xx)	FPV-NTZ (N=xx)	SOF/DCV (N=xx)	Overall (N=xx)
Temperature (degree C)						
n	xx	xx	xx	xx	xx	xx
Mean	xx.x	xx.x	xx.x	xx.x	xx.x	xx.x
Standard deviation	x.xx	x.xx	x.xx	x.xx	x.xx	x.xx
Minimum	xx	xx	xx	xx	xx	xx
Q1	xx.x	xx.x	xx.x	xx.x	xx.x	xx.x
Median	xx.x	xx	xx	xx	xx	xx
Q3	xx.x	xx.x	xx.x	xx.x	xx.x	xx.x
Maximum	xx	xx	xx	xx	xx	xx
Pulse rate (beats/min)						
n	xx	xx	xx	xx	xx	xx
Mean	xx.x	xx.x	xx.x	xx.x	xx.x	xx.x
Standard deviation	x.xx	x.xx	x.xx	x.xx	x.xx	x.xx
Minimum	xx	xx	xx	xx	xx	xx
Q1	xx.x	xx.x	xx.x	xx.x	xx.x	xx.x
Median	xx.x	xx	xx	xx	xx	xx
Q3	xx.x	xx.x	xx.x	xx.x	xx.x	xx.x
Maximum	xx	xx	xx	xx	xx	xx

Table 14.1.5 Baseline characteristics by treatment arm and overall (mITT Analysis Set)
(Page x of y)

Variable/<br>Statistic/Category	SOC<br>(N=xx)	ASAQ<br>(N=xx)	PA<br>(N=xx)	FPV-NTZ<br>(N=xx)	SOF/DCV<br>(N=xx)	Overall<br>(N=xx)
Respiratory rate (breaths/min)						
n	xx	xx	xx	xx	xx	xx
Mean	xx.x	xx.x	xx.x	xx.x	xx.x	xx.x
Standard deviation	x.xx	x.xx	x.xx	x.xx	x.xx	x.xx
Minimum	xx	xx	xx	xx	xx	xx
Q1	xx.x	xx.x	xx.x	xx.x	xx.x	xx.x
Median	xx.x	xx	xx	xx	xx	xx
Q3	xx.x	xx.x	xx.x	xx.x	xx.x	xx.x
Maximum	xx	xx	xx	xx	xx	xx
SpO2 (%)						
n	xx	xx	xx	xx	xx	xx
Mean	xx.x	xx.x	xx.x	xx.x	xx.x	xx.x
Standard deviation	x.xx	x.xx	x.xx	x.xx	x.xx	x.xx
Minimum	xx	xx	xx	xx	xx	xx
Q1	xx.x	xx.x	xx.x	xx.x	xx.x	xx.x
Median	xx.x	xx	xx	xx	xx	xx
Q3	xx.x	xx.x	xx.x	xx.x	xx.x	xx.x
Maximum	xx	xx	xx	xx	xx	xx

Table 14.1.5 Baseline characteristics by treatment arm and overall (mITT Analysis Set)
(Page x of y)

Variable/ Statistic/Category	SOC (N=xx)	ASAQ (N=xx)	PA (N=xx)	FPV-NTZ (N=xx)	SOF/DCV (N=xx)	Overall (N=xx)
Days of symptoms at time of enrollment						
n	xx	xx	xx	xx	xx	xx
Mean	xx.x	xx.x	xx.x	xx.x	xx.x	xx.x
Standard deviation	x.xx	x.xx	x.xx	x.xx	x.xx	x.xx
Minimum	xx	xx	xx	xx	xx	xx
Q1	xx.x	xx.x	xx.x	xx.x	xx.x	xx.x
Median	xx.x	xx	xx	xx	xx	xx
Q3	xx.x	xx.x	xx.x	xx.x	xx.x	xx.x
Maximum	xx	xx	xx	xx	xx	xx
WHO Ordinal Scale for Clinical Improvement, n (%)						
0	xx (xx.x)	xx (xx.x)	xx (xx.x)	xx (xx.x)	xx (xx.x)	xx (xx.x)
1	xx (xx.x)	xx (xx.x)	xx (xx.x)	xx (xx.x)	xx (xx.x)	xx (xx.x)
2	xx (xx.x)	xx (xx.x)	xx (xx.x)	xx (xx.x)	xx (xx.x)	xx (xx.x)
3	xx (xx.x)	xx (xx.x)	xx (xx.x)	xx (xx.x)	xx (xx.x)	xx (xx.x)
4	xx (xx.x)	xx (xx.x)	xx (xx.x)	xx (xx.x)	xx (xx.x)	xx (xx.x)
5	xx (xx.x)	xx (xx.x)	xx (xx.x)	xx (xx.x)	xx (xx.x)	xx (xx.x)
6	xx (xx.x)	xx (xx.x)	xx (xx.x)	xx (xx.x)	xx (xx.x)	xx (xx.x)
7	xx (xx.x)	xx (xx.x)	xx (xx.x)	xx (xx.x)	xx (xx.x)	xx (xx.x)
8	xx (xx.x)	xx (xx.x)	xx (xx.x)	xx (xx.x)	xx (xx.x)	xx (xx.x)

Programming note: For WHO ordinal scale present only categories with count > 0.

Table 14.1.6 Medical and surgical history by treatment arm and overall, MedDRA primary system organ class and preferred term (mITT Analysis Set)
(Page x of y)

Primary system organ class<br>Preferred term	SOC<br>(N=xx)<br>n (%)	ASAQ<br>(N=xx)<br>n (%)	PA<br>(N=xx)<br>n (%)	FPV-NTZ<br>(N=xx)<br>n (%)	SOF/DCV<br>(N=xx)<br>n (%)	Overall<br>(N=xx)<br>n (%)
At least one medical history	xx (xx.x)	xx (xx.x)	xx (xx.x)	xx (xx.x)	xx (xx.x)	xx (xx.x)
Primary system organ class 1	xx (xx.x)	xx (xx.x)	xx (xx.x)	xx (xx.x)	xx (xx.x)	xx (xx.x)
Preferred term 1	xx (xx.x)	xx (xx.x)	xx (xx.x)	xx (xx.x)	xx (xx.x)	xx (xx.x)
Preferred term 2	xx (xx.x)	xx (xx.x)	xx (xx.x)	xx (xx.x)	xx (xx.x)	xx (xx.x)
Primary system organ class 2	xx (xx.x)	xx (xx.x)	xx (xx.x)	xx (xx.x)	xx (xx.x)	xx (xx.x)
Preferred term 1	xx (xx.x)	xx (xx.x)	xx (xx.x)	xx (xx.x)	xx (xx.x)	xx (xx.x)
Preferred term 2	xx (xx.x)	xx (xx.x)	xx (xx.x)	xx (xx.x)	xx (xx.x)	xx (xx.x)
etc.						

A participant with multiple events within a primary system organ class is counted only once for that system organ class.
A participant with multiple events within a preferred term is counted only once for that preferred term.
Medical history related to COVID-19 disease are not reported in this table.
MedDRA version xx.x has been used for coding.

Programming note: Sort primary system organ classes alphabetically. Sort preferred terms within system organ classes by descending frequency in the "overall" column. The total n within each cohort will be used as denominator.

Table 14.1.7 Comorbidities of interest for SARS-CoV-2 infection by treatment arm and overall, MedDRA primary system organ class and preferred term (mITT Analysis Set)
(Page x of y)

Primary system organ class<br>Preferred term	SOC<br>(N=xx)<br>n (%)	ASAQ<br>(N=xx)<br>n (%)	PA<br>(N=xx)<br>n (%)	FPV-NTZ<br>(N=xx)<br>n (%)	SOF/DCV<br>(N=xx)<br>n (%)	Overall<br>(N=xx)<br>n (%)
At least one comorbitity	xx (xx.x)	xx (xx.x)	xx (xx.x)	xx (xx.x)	xx (xx.x)	xx (xx.x)
Primary system organ class 1	xx (xx.x)	xx (xx.x)	xx (xx.x)	xx (xx.x)	xx (xx.x)	xx (xx.x)
Preferred term 1	xx (xx.x)	xx (xx.x)	xx (xx.x)	xx (xx.x)	xx (xx.x)	xx (xx.x)
Preferred term 2	xx (xx.x)	xx (xx.x)	xx (xx.x)	xx (xx.x)	xx (xx.x)	xx (xx.x)
Primary system organ class 2	xx (xx.x)	xx (xx.x)	xx (xx.x)	xx (xx.x)	xx (xx.x)	xx (xx.x)
Preferred term 1	xx (xx.x)	xx (xx.x)	xx (xx.x)	xx (xx.x)	xx (xx.x)	xx (xx.x)
Preferred term 2	xx (xx.x)	xx (xx.x)	xx (xx.x)	xx (xx.x)	xx (xx.x)	xx (xx.x)
etc.						

A participant with multiple events within a primary system organ class is counted only once for that system organ class.
A participant with multiple events within a preferred term is counted only once for that preferred term.
Only comorbidities pre-specified on the CRF are presented.
MedDRA version xx.x has been used for coding.

Programming note: Sort primary system organ classes alphabetically. Sort preferred terms within system organ classes by descending frequency in the "overall" column. The total n within each cohort will be used as denominator.

Table 14.1.8 Prior medications by ATC class and WHO preferred term by treatment arm and overall, ATC class and preferred term (Safety Analysis Set)
(Page x of y)

ATC class/<br>Preferred term	SOC<br>(N=xx)<br>n (%)	ASAQ<br>(N=xx)<br>n (%)	PA<br>(N=xx)<br>n (%)	FPV-NTZ<br>(N=xx)<br>n (%)	SOF/DCV<br>(N=xx)<br>n (%)	Overall<br>(N=xx)<br>n (%)
At least one medication	xx (xx.x)	xx (xx.x)	xx (xx.x)	xx (xx.x)	xx (xx.x)	xx (xx.x)
ATC class 1	xx (xx.x)	xx (xx.x)	xx (xx.x)	xx (xx.x)	xx (xx.x)	xx (xx.x)
Preferred term 1	xx (xx.x)	xx (xx.x)	xx (xx.x)	xx (xx.x)	xx (xx.x)	xx (xx.x)
Preferred term 2	xx (xx.x)	xx (xx.x)	xx (xx.x)	xx (xx.x)	xx (xx.x)	xx (xx.x)
ATC class 2	xx (xx.x)	xx (xx.x)	xx (xx.x)	xx (xx.x)	xx (xx.x)	xx (xx.x)
Preferred term 1	xx (xx.x)	xx (xx.x)	xx (xx.x)	xx (xx.x)	xx (xx.x)	xx (xx.x)
Preferred term 2	xx (xx.x)	xx (xx.x)	xx (xx.x)	xx (xx.x)	xx (xx.x)	xx (xx.x)
etc.						

A participant can have more than one prior medication.
Medications were coded using the World Health Organizational-Drug Dictionary (WHO-DD) version xx.

Programming note: Use ATC class 3. Sort ATC classes alphabetically. Sort preferred terms within ATC classes by descending frequency in the overall column. The total n within each cohort will be used as denominator.

Table 14.2.1.1 Primary analysis of incidence of SARS-CoV-2 clearance on Day 7 based on RT-PCR (mITT Analysis Set)

				Covariate adjusted analysis*			Crude analysis*		
Treatment arm	N	n/M (%)	Comparison	Risk ratio	95% CI	p-value	Risk ratio	95% CI	p-value
SOC	xx	xx/xx (xx.x)							
ASAQ	xx	xx/xx (xx.x)	ASAQ / SOC	xx.x	(xx.x, xx.x)	x.xxx	xx.x	(xx.x, xx.x)	x.xxx
PA	xx	xx/xx (xx.x)	PA / SOC	xx.x	(xx.x, xx.x)	x.xxx	xx.x	(xx.x, xx.x)	x.xxx
FPV-NTZ	xx	xx/xx (xx.x)	FPV-NTZ / SOC	xx.x	(xx.x, xx.x)	x.xxx	xx.x	(xx.x, xx.x)	x.xxx
SOF/DCV	xx	xx/xx (xx.x)	SOF/DCV / SOC	xx.x	(xx.x, xx.x)	x.xxx	xx.x	(xx.x, xx.x)	x.xxx

N = number of participants in the respective treatment arm.
n = number of participants with SARS-CoV-2 clearance on Day 7 based on RT-PCR test.
M = number of evaluable participants for Day 7.
The covariate adjusted regression model contains treatment arm, age at baseline (years), sex, baseline BMI, baseline comorbidities, baseline viral load category and days of symptoms at time of enrollment as covariates.
The crude analysis repeats the regression model without any covariate adjustment.
A risk ratio > 1 favors the treatment arm in the numerator of the ratio (= higher proportion of SARS-CoV-2 clearance).
Model specification: * = log link and binomial distribution, # = log link and poisson distribution, § = log link and normal distribution.

Table 14.2.1.2 Sensitivity analysis #1 for incidence of SARS-CoV-2 clearance on Day 7 based on RT-PCR (mITT Analysis set)

Programming note: Same layout as table 14.2.1.1.
All participants who discontinue prior to the day 7 SARS-CoV-2 RT-PCR test are imputed as having a positive of SARS-CoV-2 RT-PCR test on Day 7.

Table 14.2.1.3 Sensitivity analysis #2 for incidence of SARS-CoV-2 clearance on Day 7 based on RT-PCR (As Treated Analysis set)

Programming note: Same layout as table 14.2.1.1.

Table 14.2.1.4 Primary analysis of incidence of SARS-CoV-2 clearance on Day 7 based on RT-PCR by subgroup (mITT Analysis Set)

Subgroup		Treatment arm	N	n/M (%)	Comparison	Risk ratio	95% CI	p-value
Age group at baseline*	Overall	SOC	xx	xx/xx (xx.x)				
		ASAQ	xx	xx/xx (xx.x)	ASAQ / SOC	xx.x	(xx.x, xx.x)	x.xxx
		PA	xx	xx/xx (xx.x)	PA / SOC	xx.x	(xx.x, xx.x)	x.xxx
		FPV-NTZ	xx	xx/xx (xx.x)	FPV-NTZ / SOC	xx.x	(xx.x, xx.x)	x.xxx
		SOF/DCV	xx	xx/xx (xx.x)	SOF/DCV / SOC	xx.x	(xx.x, xx.x)	x.xxx
					Interaction effect			x.xxx
	Low (<= median)	SOC	xx	xx/xx (xx.x)				
		ASAQ	xx	xx/xx (xx.x)	ASAQ / SOC	xx.x	(xx.x, xx.x)	x.xxx
		PA	xx	xx/xx (xx.x)	PA / SOC	xx.x	(xx.x, xx.x)	x.xxx
		FPV-NTZ	xx	xx/xx (xx.x)	FPV-NTZ / SOC	xx.x	(xx.x, xx.x)	x.xxx
		SOF/DCV	xx	xx/xx (xx.x)	SOF/DCV / SOC	xx.x	(xx.x, xx.x)	x.xxx
	High (> median)	SOC	xx	xx/xx (xx.x)				
		ASAQ	xx	xx/xx (xx.x)	ASAQ / SOC	xx.x	(xx.x, xx.x)	x.xxx
		PA	xx	xx/xx (xx.x)	PA / SOC	xx.x	(xx.x, xx.x)	x.xxx
		FPV-NTZ	xx	xx/xx (xx.x)	FPV-NTZ / SOC	xx.x	(xx.x, xx.x)	x.xxx
		SOF/DCV	xx	xx/xx (xx.x)	SOF/DCV / SOC	xx.x	(xx.x, xx.x)	x.xxx

N = number of participants in the respective treatment arm.
n = number of participants with SARS-CoV-2 clearance on Day 7 based on RT-PCR.
M = number of evaluable participants for Day 7.
The regression model for each subgroup contains all covariates from the primary analysis and the interaction between the respective subgroup variable and the treatment arm.
A risk ratio > 1 favors the treatment arm in the numerator of the ratio (= higher proportion of SARS-CoV-2 clearance).
Model specification: * = log link and binomial distribution, # = log link and poisson distribution, § = log link and normal distribution.

Programming note: Display subgroup analysis for all other subgroup variables (sex, BMI at baseline, baseline comorbidities, baseline viral load category, days of symptoms at time of enrollment, baseline risk category) on the following pages. Use new page for each subgroup. For subgroup variables categorized by median, display median value in brackets. For the subgroup analysis of baseline risk category this variable is also used as covariate. In general use the same covariates as in the primary efficacy endpoint model. In case a subgroup analysis is done for a categorized version of a continuous variable the categorized version is used as covariate (instead of the continuous version).

Table 14.2.1.5 Incidence of SARS-CoV-2 clearance on Day 7 based on viral culture (mITT Analysis Set)

Treatment arm	N	n/M (%)	Comparison*	Risk ratio	95% CI	p-value
SOC	xx	xx/xx (xx.x)				
ASAQ	xx	xx/xx (xx.x)	ASAQ / SOC	xx.x	(xx.x, xx.x)	x.xxx
PA	xx	xx/xx (xx.x)	PA / SOC	xx.x	(xx.x, xx.x)	x.xxx
FPV-NTZ	xx	xx/xx (xx.x)	FPV-NTZ / SOC	xx.x	(xx.x, xx.x)	x.xxx
SOF/DCV	xx	xx/xx (xx.x)	SOF/DCV / SOC	xx.x	(xx.x, xx.x)	x.xxx

N = number of participants in the respective treatment arm.
n = number of participants with SARS-CoV-2 clearance on Day 7 based on viral culture.
M = number of evaluable participants for Day 7.
The regression model contains treatment arm, age at baseline (years), sex, baseline BMI, baseline comorbidities, baseline viral load category and days of symptoms at time of enrollment as covariates.
A risk ratio > 1 favors the treatment arm in the numerator of the ratio (= higher proportion of SARS-CoV-2 clearance).
Model specification: * = log link and binomial distribution, # = log link and poisson distribution, § = log link and normal distribution.

Table 14.2.1.6 Incidence of SARS-CoV-2 clearance on Day 3, 10, 14, 21 and 28 based on RT-PCR (mITT Analysis Set)

Time point	Treatment arm	N	n/M (%)	Comparison	Risk ratio	95% CI	p-value
Day 3*							
	SOC	xx	xx/xx (xx.x)				
	ASAQ	xx	xx/xx (xx.x)	ASAQ / SOC	xx.x	(xx.x, xx.x)	x.xxx
	PA	xx	xx/xx (xx.x)	PA / SOC	xx.x	(xx.x, xx.x)	x.xxx
	FPV-NTZ	xx	xx/xx (xx.x)	FPV-NTZ / SOC	xx.x	(xx.x, xx.x)	x.xxx
	SOF/DCV	xx	xx/xx (xx.x)	SOF/DCV / SOC	xx.x	(xx.x, xx.x)	x.xxx
Day 10							
	SOC	xx	xx/xx (xx.x)				
	ASAQ	xx	xx/xx (xx.x)	ASAQ / SOC	xx.x	(xx.x, xx.x)	x.xxx
	PA	xx	xx/xx (xx.x)	PA / SOC	xx.x	(xx.x, xx.x)	x.xxx
	FPV-NTZ	xx	xx/xx (xx.x)	FPV-NTZ / SOC	xx.x	(xx.x, xx.x)	x.xxx
	SOF/DCV	xx	xx/xx (xx.x)	SOF/DCV / SOC	xx.x	(xx.x, xx.x)	x.xxx
Etc...							

N = number of participants in the respective treatment arm.
n = number of participants with SARS-CoV-2 clearance at respective time point based on RT-PCR test.
M = number of evaluable participants for respective time point.
The regression model contains treatment arm, age at baseline (years), sex, baseline BMI, baseline comorbidities, baseline viral load category and days of symptoms at time of enrollment as covariates.
A risk ratio > 1 favors the treatment arm in the numerator of the ratio (= higher proportion of SARS-CoV-2 clearance).
Model specification: * = log link and binomial distribution, # = log link and poisson distribution, § = log link and normal distribution.

Programming note: Display table for time points Day 3, Day 10, Day 14, Day 21 and Day 28.

Table 14.2.2.1 Kaplan Meier analysis of time to SARS-CoV-2 clearance based on qualitative RT-PCR (mITT Analysis Set)
(Page x of y)

Statistic	SOC (N=xx)	ASAQ (N=xx)	PA (N=xx)	FPV-NTZ (N=xx)	SOF/DCV (N=xx)
Available observations	xx	xx	xx	xx	xx
Number of participants with SARS-CoV-2 clearance	xx	xx	xx	xx	xx
Number of censored observations	xx	xx	xx	xx	xx
SARS-CoV-2 clearances over time (days)					
25th percentile (Two-sided 95% CI)	xx.x (xx.x, xx.x)	xx.x (xx.x, xx.x)	xx.x (xx.x, xx.x)	xx.x (xx.x, xx.x)	xx.x (xx.x, xx.x)
Median (Two-sided 95% CI)	xx.x (xx.x, xx.x)	xx.x (xx.x, xx.x)	xx.x (xx.x, xx.x)	xx.x (xx.x, xx.x)	xx.x (xx.x, xx.x)
75th percentile (Two-sided 95% CI)	xx.x (xx.x, xx.x)	xx.x (xx.x, xx.x)	xx.x (xx.x, xx.x)	xx.x (xx.x, xx.x)	xx.x (xx.x, xx.x)
% SARS-CoV-2 clearance estimate (95% CI)					
Day 7	xx.x (xx.x, xx.x)	xx.x (xx.x, xx.x)	xx.x (xx.x, xx.x)	xx.x (xx.x, xx.x)	xx.x (xx.x, xx.x)
Day 14	xx.x (xx.x, xx.x)	xx.x (xx.x, xx.x)	xx.x (xx.x, xx.x)	xx.x (xx.x, xx.x)	xx.x (xx.x, xx.x)
Day 21	xx.x (xx.x, xx.x)	xx.x (xx.x, xx.x)	xx.x (xx.x, xx.x)	xx.x (xx.x, xx.x)	xx.x (xx.x, xx.x)
Day 28	xx.x (xx.x, xx.x)	xx.x (xx.x, xx.x)	xx.x (xx.x, xx.x)	xx.x (xx.x, xx.x)	xx.x (xx.x, xx.x)

Estimates are based on Kaplan-Meier methodology.

Participants who did not experience the event of interest are considered censored according to the censoring rules detailed in Appendix 16.1.9.

Time to SARS-CoV-2 clearance is defined as the time to first negative qualitative SARS-CoV-2 RT-PCR test without any subsequent positive qualitative SARS-CoV-2 RT-PCR test.

SARS-Cov-2 clearance over time: presents the time point (in days) by which the estimated cumulative probability of SARS-CoV-2 is 25%, 50% and 75% and the corresponding two-sided 95% confidence interval.

% SARS-CoV-2 clearance estimate: presents the estimated cumulative probability of SARS-CoV-2 clearance by Day 7/14/21/28 and the corresponding two-sided 95% confidence interval.

n.e. = not estimable.

Table 14.2.2.2 Kaplan Meier analysis of time to SARS-CoV-2 clearance based on quantitative RT-PCR (mITT Analysis Set)
(Page x of y)

Statistic	SOC (N=xx)	ASAQ (N=xx)	PA (N=xx)	FPV-NTZ (N=xx)	SOF/DCV (N=xx)
Available observations	xx	xx	xx	xx	xx
Number of participants with SARS-CoV-2 clearance	xx	xx	xx	xx	xx
Number of censored observations	xx	xx	xx	xx	xx
SARS-CoV-2 clearances over time (days)					
25th percentile (Two-sided 95% CI)	xx.x (xx.x, xx.x)	xx.x (xx.x, xx.x)	xx.x (xx.x, xx.x)	xx.x (xx.x, xx.x)	xx.x (xx.x, xx.x)
Median (Two-sided 95% CI)	xx.x (xx.x, xx.x)	xx.x (xx.x, xx.x)	xx.x (xx.x, xx.x)	xx.x (xx.x, xx.x)	xx.x (xx.x, xx.x)
75th percentile (Two-sided 95% CI)	xx.x (xx.x, xx.x)	xx.x (xx.x, xx.x)	xx.x (xx.x, xx.x)	xx.x (xx.x, xx.x)	xx.x (xx.x, xx.x)
% SARS-CoV-2 clearance estimate (95% CI)					
Day 7	xx.x (xx.x, xx.x)	xx.x (xx.x, xx.x)	xx.x (xx.x, xx.x)	xx.x (xx.x, xx.x)	xx.x (xx.x, xx.x)
Day 14	xx.x (xx.x, xx.x)	xx.x (xx.x, xx.x)	xx.x (xx.x, xx.x)	xx.x (xx.x, xx.x)	xx.x (xx.x, xx.x)
Day 21	xx.x (xx.x, xx.x)	xx.x (xx.x, xx.x)	xx.x (xx.x, xx.x)	xx.x (xx.x, xx.x)	xx.x (xx.x, xx.x)
Day 28	xx.x (xx.x, xx.x)	xx.x (xx.x, xx.x)	xx.x (xx.x, xx.x)	xx.x (xx.x, xx.x)	xx.x (xx.x, xx.x)

Estimates are based on Kaplan-Meier methodology.
Participants who did not experience the event of interest are considered censored according to the censoring rules detailed in Appendix 16.1.9.
Time to SARS-CoV-2 clearance is defined as the time to first quantitative SARS-CoV-2 RT-PCR test with zero count without any subsequent count above zero.
SARS-Cov-2 clearance over time: presents the time point (in days) by which the estimated cumulative probability of SARS-CoV-2 is 25%, 50% and 75% and the corresponding two-sided 95% confidence interval.
% SARS-CoV-2 clearance estimate: presents the estimated cumulative probability of SARS-CoV-2 clearance by Day 7/14/21/28 and the corresponding two-sided 95% confidence interval.

n.e. = not estimable.

Table 14.2.2.3 Kaplan Meier analysis of time to SARS-CoV-2 clearance based on quantitative RT-PCR (mITT Analysis Set - high-risk participants)
(Page x of y)

Programming note: Use same layout as table 14.2.2.2. Use mITT Analysis Set restricted to the subset of high-risk participants.

Table 14.2.2.4 Kaplan Meier analysis of time to SARS-CoV-2 clearance based on quantitative RT-PCR (mITT Analysis Set – high baseline viral load)
(Page x of y)

Programming note: Use same layout as table 14.2.2.2. Use mITT Analysis Set restricted to the subset of participants with high viral load at baseline.

Table 14.2.2.5 Cox regression analysis of time to SARS-CoV-2 clearance based on qualitative RT-PCR (mITT Analysis Set)

Treatment arm	N	n/M (%)	Comparison	Hazard ratio	95% CI	p-value
SOC	xx	xx/xx (xx.x)				
ASAQ	xx	xx/xx (xx.x)	ASAQ / SOC	xx.x	(xx.x, xx.x)	x.xxx
PA	xx	xx/xx (xx.x)	PA / SOC	xx.x	(xx.x, xx.x)	x.xxx
FPV-NTZ	xx	xx/xx (xx.x)	FPV-NTZ / SOC	xx.x	(xx.x, xx.x)	x.xxx
SOF/DCV	xx	xx/xx (xx.x)	SOF/DCV / SOC	xx.x	(xx.x, xx.x)	x.xxx

N = number of participants in the respective treatment arm.
n = number of participants with SARS-CoV-2 clearance based on RT-PCR test.
M = number of evaluable participants.
Time to SARS-CoV-2 clearance is defined as the time to first negative qualitative SARS-CoV-2 RT-PCR test without any subsequent positive qualitative SARS-CoV-2 RT-PCR test.
The cox regression model contains treatment arm, age at baseline (years), sex, baseline BMI, baseline comorbidities, baseline viral load category and days of symptoms at time of enrollment as covariates.
A hazard ratio > 1 favors the treatment arm in the numerator of the ratio (= higher probability of SARS-CoV-2 clearance).

Table 14.2.2.6 Cox regression analysis of time to SARS-CoV-2 clearance based on quantitative RT-PCR (mITT Analysis Set)

Programming note: Use same layout as table 14.2.2.5.

Footnotes:
N = number of participants in the respective treatment arm.
n = number of participants with SARS-CoV-2 clearance based on RT-PCR test.
M = number of evaluable participants.
Time to SARS-CoV-2 clearance is defined as the time to first quantitative SARS-CoV-2 RT-PCR test with zero count without any subsequent count above zero.
The cox regression model contains treatment arm, age at baseline (years), sex, baseline BMI, baseline comorbidities, baseline viral load category and days of symptoms at time of enrollment as covariates.
A hazard ratio > 1 favors the treatment arm in the numerator of the ratio (= higher probability of SARS-CoV-2 clearance).

Table 14.2.2.7 Cox regression analysis of time to SARS-CoV-2 clearance based on quantitative RT-PCR (mITT Analysis Set – high-risk participants)

Programming note: Use same layout as table 14.2.2.6. Use mITT Analysis Set restricted to the subset of high-risk participants.

Table 14.2.2.8 Cox regression analysis of time to SARS-CoV-2 clearance based on quantitative RT-PCR (mITT Analysis Set – high baseline viral load)

Programming note: Use same layout as table 14.2.2.6. Use mITT Analysis Set restricted to the subset of participants with high viral load at baseline.

Figure 14.2.2.1 Kaplan-Meier curves for time to SARS-CoV-2 clearance based on qualitative RT-PCR (m-ITT Analysis Set)

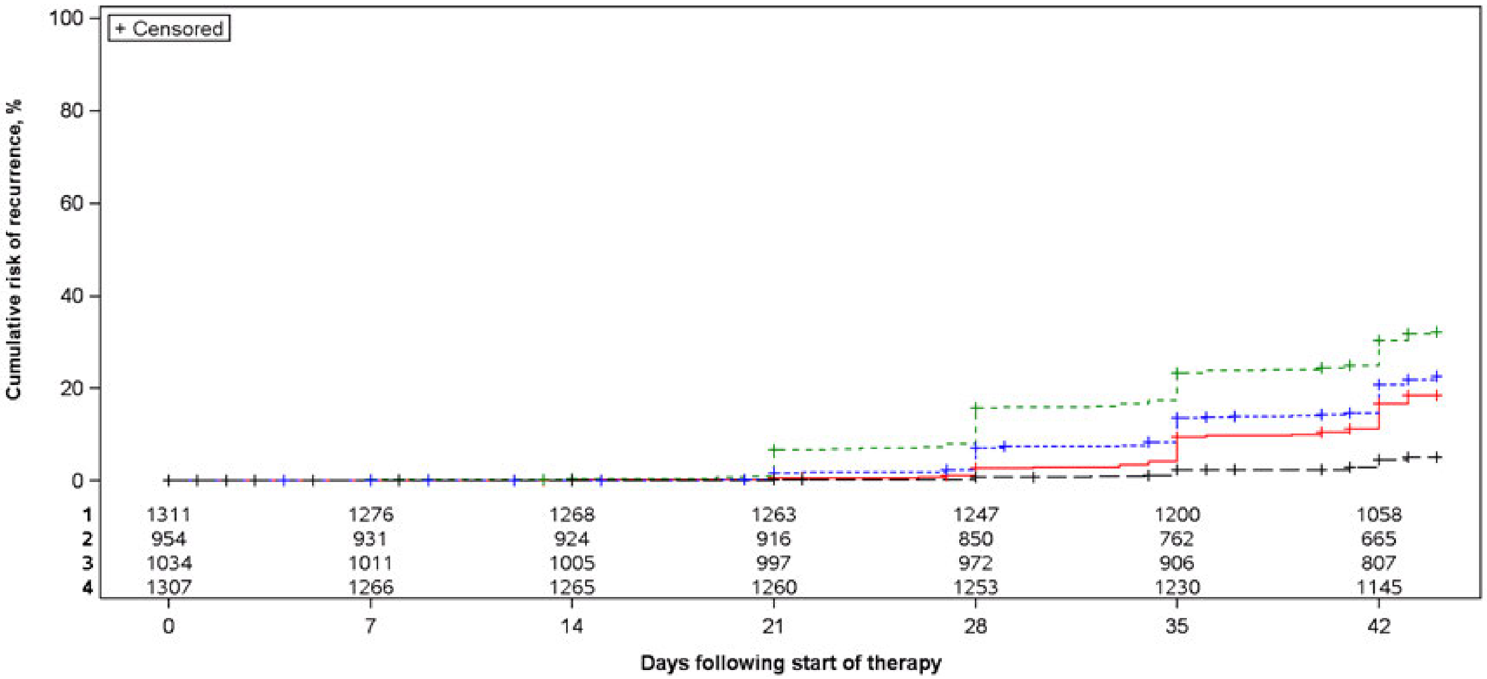

Programming note: Figure layout will be similar to the above. Display the following treatment arms: SOC, ASAQ, PA, FPV-NTZ, SOF/DCV. Use different colors and line styles to differentiate between survival curves. Use x-axis label: Study day following first study drug intake. Use y-axis label: Cumulative probability of SARS-CoV-2 clearance. Display x-axis until Day 28. Time until SARS-CoV-2 clearance will be truncated after Day 28 in order to avoid a sudden drop in number of participants at risk. Also display the p-value of the log-rank test.

Footnotes:

Time to SARS-CoV-2 clearance is defined as the time to first negative qualitative SARS-CoV-2 RT-PCR test without any subsequent positive SARS-CoV-2 RT-PCR test.

Participants who did not experience the event of interest are considered censored according to the censoring rules detailed in the SAP.

Figure 14.2.2.2 Kaplan-Meier curves for time to SARS-CoV-2 clearance based on quantitative RT-PCR (m-ITT Analysis Set)

Programming note: Same layout as figure 14.2.2.1.

Footnotes:
Time to SARS-CoV-2 clearance is defined as the time to first quantitative SARS-CoV-2 RT-PCR test with zero count without any subsequent count above zero.
Participants who did not experience the event of interest are considered censored according to the censoring rules detailed in the SAP.

Figure 14.2.2.3 Kaplan-Meier curves for time to SARS-CoV-2 clearance based on quantitative RT-PCR (m-ITT Analysis Set – high-risk participants)

Programming note: Use same layout as figure 14.2.2.2. Use mITT Analysis Set restricted to the subset of high-risk participants.

Figure 14.2.2.4 Kaplan-Meier curves for time to SARS-CoV-2 clearance based on quantitative RT-PCR (m-ITT Analysis Set – high baseline viral load)

Programming note: Use same layout as figure 14.2.2.2. Use mITT Analysis Set restricted to the subset of participants with high viral load at baseline.

Table 14.2.3.1 Summary of log10 viral load of SARS-CoV-2 by treatment arm and overall and time point (mITT Analysis Set)
(Page x of y)

Time point<br>Statistic	SOC<br>(N=xx)			ASAQ<br>(N=xx)			PA<br>(N=xx)		
	Base	Post	Change	Base	Post	Change	Base	Post	Change
Baseline									
n (%)	xx<br>(xx.x)			xx (xx.x)			xx (xx.x)		
Mean	xx.xx			xx.xx			xx.xx		
SD	xx.xxx			xx.xxx			xx.xxx		
Minimum	xx.x			xx.x			xx.x		
Q1	xx.xx			xx.xx			xx.xx		
Median	xx.xx			xx.xx			xx.xx		
Q3	xx.xx			xx.xx			xx.xx		
Maximum	xx.x			xx.x			xx.x		
Day 3									
n (%)	xx<br>(xx.x)	xx (xx.x)	xx xx.x)	xx (xx.x)	xx (xx.x)	xx (xx.x)	xx (xx.x)	xx (xx.x)	xx (xx.x)
Mean	xx.xx	xx.xx	xx.xx	xx.xx	xx.xx	xx.xx	xx.xx	xx.xx	xx.xx
SD	xx.xxx	xx.xxx	xx.xxx	xx.xxx	xx.xxx	xx.xxx	xx.xxx	xx.xxx	xx.xxx
Minimum	xx.x	xx.x	xx.x	xx.x	xx.x	xx.x	xx.x	xx.x	xx.x
Q1	xx.xx	xx.xx	xx.xx	xx.xx	xx.xx	xx.xx	xx.xx	xx.xx	xx.xx
Median	xx.xx	xx.xx	xx.xx	xx.xx	xx.xx	xx.xx	xx.xx	xx.xx	xx.xx
Q3	xx.xx	xx.xx	xx.xx	xx.xx	xx.xx	xx.xx	xx.xx	xx.xx	xx.xx
Maximum	xx.x	xx.x	xx.x	xx.x	xx.x	xx.x	xx.x	xx.x	xx.x
Day 7									
etc...									

Base = Baseline, Post = Post-baseline, Change = Post-baseline - baseline.
At each visit, only participants with a value at both baseline and the respective day are included.
Analysis is based on quantitative RT-PCR.
Viral load values (copies/mL) are log10 transformed.

Table 14.2.3.1 Summary of log10 viral load of SARS-CoV-2 by treatment arm and overall and time point (mITT Analysis Set)
(Page x of y)

Time point	FPV-NTZ (N=xx)			SOF/DCF (N=xx)			Overall (N=xx)		
Statistic	Base	Post	Change	Base	Post	Change	Base	Post	Change
Baseline									
n (%)	xx (xx.x)			xx (xx.x)			xx (xx.x)		
Mean	xx.xx			xx.xx			xx.xx		
SD	xx.xxx			xx.xxx			xx.xxx		
Minimum	xx.x			xx.x			xx.x		
Q1	xx.xx			xx.xx			xx.xx		
Median	xx.xx			xx.xx			xx.xx		
Q3	xx.xx			xx.xx			xx.xx		
Maximum	xx.x			xx.x			xx.x		
Day 3									
n (%)	xx (xx.x)	xx (xx.x)	xx (xx.x)	xx (xx.x)	xx (xx.x)	xx (xx.x)	xx (xx.x)	xx (xx.x)	xx (xx.x)
Mean	xx.xx	xx.xx	xx.xx	xx.xx	xx.xx	xx.xx	xx.xx	xx.xx	xx.xx
SD	xx.xxx	xx.xxx	xx.xxx	xx.xxx	xx.xxx	xx.xxx	xx.xxx	xx.xxx	xx.xxx
Minimum	xx.x	xx.x	xx.x	xx.x	xx.x	xx.x	xx.x	xx.x	xx.x
Q1	xx.xx	xx.xx	xx.xx	xx.xx	xx.xx	xx.xx	xx.xx	xx.xx	xx.xx
Median	xx.xx	xx.xx	xx.xx	xx.xx	xx.xx	xx.xx	xx.xx	xx.xx	xx.xx
Q3	xx.xx	xx.xx	xx.xx	xx.xx	xx.xx	xx.xx	xx.xx	xx.xx	xx.xx
Maximum	xx.x	xx.x	xx.x	xx.x	xx.x	xx.x	xx.x	xx.x	xx.x
Day 7									
Etc...									

Base = Baseline, Post = Post-baseline, Change = Post-baseline - baseline.
At each visit, only participants with a value at both baseline and the respective day are included.
Analysis is based on quantitative RT-PCR.
Viral load values (copies/mL) are log10 transformed.
Programming note: Present this table for time points Baseline, Day 3, Day 7, Day 10, Day 14and End of study.

Table 14.2.3.2 Repeated measure analysis of log10 viral load of SARS-CoV-2 change from baseline (mITT Analysis Set)
(Page x of y)

		Test			Reference			Comparison of LSmean change		
Test vs Ref. (Comparison)	Time point	n	LSmean change (SE)	95% CI	n	LSmean change (SE)	95% CI	Diff. Test- Ref. (SE)	95% CI	2-sided p-value
ASAQ (N=xx) vs SOC (N=xx)	Day 3	xx	xx.x (xx.xx)	(xx.x, xx.x)	xx	xx.x (xx.xx)	(xx.x, xx.x)	xx.x (xx.xx)	(xx.x, xx.x)	x.xxx
	Day 7	xx	xx.x (xx.xx)	(xx.x, xx.x)	xx	xx.x (xx.xx)	(xx.x, xx.x)	xx.x (xx.xx)	(xx.x, xx.x)	x.xxx
	Day 10	xx	xx.x (xx.xx)	(xx.x, xx.x)	xx	xx.x (xx.xx)	(xx.x, xx.x)	xx.x (xx.xx)	(xx.x, xx.x)	x.xxx
	Day 14	xx	xx.x (xx.xx)	(xx.x, xx.x)	xx	xx.x (xx.xx)	(xx.x, xx.x)	xx.x (xx.xx)	(xx.x, xx.x)	x.xxx

N = number of participants in the respective treatment arm.
n = number of participants with available data on respective time point.
The mixed-effect linear model for repeated measures includes treatment, age at baseline (years), sex, BMI at baseline, baseline comorbidities, baseline viral load category, days of symptoms at time of enrollment and treatment-by-visit interaction as fixed effects.

Programming note: Continue table on next pages for comparison of PA vs SOC, FPV-NTZ vs SOC and SOF/DCV vs SOC.

Table 14.2.3.3 Repeated measure analysis of log10 viral load of SARS-CoV-2 change from baseline (mITT Analysis Set – high-risk participants)
(Page x of y)

Programming note: Use same layout as table 14.2.3.2. Produce table for mITT Analysis Set in the subset of high-risk participants.

Figure 14.2.3.1 Spaghetti plot of log10 viral load of SARS-CoV-2 based quantitative RT-PCR by treatment arm (mITT Analysis Set)

Programming note: Display the individual log10 transformed viral load of SARS-CoV-2 (detected by quantitative RT-PCR based on N gene) of each participant from baseline until Day 28. Display a separate panel for each treatment arm. No visit windowing approach is applied. Viral load values are log10 transformed for the analysis. Use "Viral load (log10 copies/mL)" as y-axis label.

Table 14.2.4.1 Logistic regression for hospitalization up to Day 28 (mITT Analysis Set)
(Page x of y)

Treatment arm	N	n/M (%)	Comparison	Odds ratio	95% CI	p-value
SOC	xx	xx/xx (xx.x)				
ASAQ	xx	xx/xx (xx.x)	ASAQ / SOC	xx.x	(xx.x, xx.x)	x.xxx
PA	xx	xx/xx (xx.x)	PA / SOC	xx.x	(xx.x, xx.x)	x.xxx
FPV-NTZ	xx	xx/xx (xx.x)	FPV-NTZ / SOC	xx.x	(xx.x, xx.x)	x.xxx
SOF/DCV	xx	xx/xx (xx.x)	SOF/DCV / SOC	xx.x	(xx.x, xx.x)	x.xxx

N = number of participants in the respective treatment arm.
n = number of participants hospitalized up to Day 28.
M = number of evaluable participants.
The logistic regression model contains treatment arm, age at baseline (years), sex, baseline BMI, baseline comorbidities, baseline viral load category and days of symptoms at time of enrollment as covariates.
An odds ratio > 1 suggests a higher chance of hospitalization in the treatment arm of the numerator of the ratio.
Only hospitalizations related to COVID-19 are analysed.

Table 14.2.4.2 Summary of hospitalization data by treatment arm and overall (mITT Analysis Set)
(Page x of y)

Variable<br>Statistic/Category	SOC<br>(N=xx)	ASAQ<br>(N=xx)	PA<br>(N=xx)	FPV-NTZ<br>(N=xx)	SOF/DCV<br>(N=xx)	Overall<br>(N=xx)
Cumulative incidence of hospitalization at Day 28, n (%)	xx (xx.x)	xx (xx.x)	xx (xx.x)	xx (xx.x)	xx (xx.x)	xx (xx.x)
p-value (Fisher's exact test)		x.xxx	x.xxx	x.xxx	x.xxx	
Number of days hospitalized						
n (%)	xx (xx.x)	xx (xx.x)	xx (xx.x)	xx (xx.x)	xx (xx.x)	xx (xx.x)
Mean	xx.xx	xx.xx	xx.xx	xx.xx	xx.xx	xx.xx
SD	xx.xxx	xx.xxx	xx.xxx	xx.xxx	xx.xxx	xx.xxx
Minimum	xx.x	xx.x	xx.x	xx.x	xx.x	xx.x
Q1	xx.xx	xx.xx	xx.xx	xx.xx	xx.xx	xx.xx
Median	xx.xx	xx.xx	xx.xx	xx.xx	xx.xx	xx.xx
Q3	xx.xx	xx.xx	xx.xx	xx.xx	xx.xx	xx.xx
Maximum	xx.x	xx.x	xx.x	xx.x	xx.x	xx.x
Interquartile range	xx.xx	xx.xx	xx.xx	xx.xx	xx.xx	xx.xx

Interquartile range is defined as Q3-Q1.
Cumulative incidence of hospitalization at Day 28 is defined as the number of participants hospitalized up to and including Day 28 divided by the number of participants in the mITT Analysis Set.
Only hospitalizations related to COVID-19 are analysed.
p-values of fisher's exact test comparing cumulative incidence of each experimental arm against the standard of care arm is displayed.

Table 14.2.4.3 Kaplan Meier analysis of time to first hospitalization (mITT Analysis Set)
(Page x of y)

Statistic	SOC (N=xx)	ASAQ (N=xx)	PA (N=xx)	FPV-NTZ (N=xx)	SOF/DCV (N=xx)
Available observations	xx	xx	xx	xx	xx
Number of participants with hospitalization	xx	xx	xx	xx	xx
Number of censored observations	xx	xx	xx	xx	xx
Hospitalizations over time (days)					
25th percentile (Two-sided 95% CI)	xx.x (xx.x, xx.x)	xx.x (xx.x, xx.x)	xx.x (xx.x, xx.x)	xx.x (xx.x, xx.x)	xx.x (xx.x, xx.x)
Median (Two-sided 95% CI)	xx.x (xx.x, xx.x)	xx.x (xx.x, xx.x)	xx.x (xx.x, xx.x)	xx.x (xx.x, xx.x)	xx.x (xx.x, xx.x)
75th percentile (Two-sided 95% CI)	xx.x (xx.x, xx.x)	xx.x (xx.x, xx.x)	xx.x (xx.x, xx.x)	xx.x (xx.x, xx.x)	xx.x (xx.x, xx.x)
% Hospitalization estimate (95% CI)					
Day 7	xx.x (xx.x, xx.x)	xx.x (xx.x, xx.x)	xx.x (xx.x, xx.x)	xx.x (xx.x, xx.x)	xx.x (xx.x, xx.x)
Day 14	xx.x (xx.x, xx.x)	xx.x (xx.x, xx.x)	xx.x (xx.x, xx.x)	xx.x (xx.x, xx.x)	xx.x (xx.x, xx.x)
Day 21	xx.x (xx.x, xx.x)	xx.x (xx.x, xx.x)	xx.x (xx.x, xx.x)	xx.x (xx.x, xx.x)	xx.x (xx.x, xx.x)
Day 28	xx.x (xx.x, xx.x)	xx.x (xx.x, xx.x)	xx.x (xx.x, xx.x)	xx.x (xx.x, xx.x)	xx.x (xx.x, xx.x)

Estimates are based on Kaplan-Meier methodology.
Participants who did not experience the event of interest are considered censored according to the censoring rules detailed in Appendix 16.1.9.
Hospitalizations over time: presents the time point (in days) by which the estimated cumulative probability of hospitalization is 25%, 50% and 75% and the corresponding two-sided 95% confidence interval.
% SARS-CoV-2 clearance estimate: presents the estimated cumulative probability of hospitalization by Day 7/14/21/28 and the corresponding two-sided 95% confidence interval.
n.e. = not estimable.

Table 14.2.4.4 Cox regression analysis of time to first hospitalization (mITT Analysis Set)

Programming note: Use same layout as table 14.2.2.3.

Footnotes:
N = number of participants in the respective treatment arm.
n = number of participants hospitalized up to Day 28.
M = number of evaluable participants
The cox regression model contains treatment arm, age at baseline (years), sex, baseline BMI, baseline comorbidities, baseline viral load category and days of symptoms at time of enrollment as covariates.
A hazard ratio > 1 favors the treatment arm in the denominator of the ratio (= lower probability of hospitalization).

Figure 14.2.4.1 Kaplan-Meier curves for time to first hospitalization (mITT Analysis Set)

Programming note: Use same figure layout as figure 14.2.2.1. Display the following treatment arms: SOC, ASAQ, PA, FPV-NTZ, SOF/DCV. Use different colors and line styles to differentiate between survival curves. Use x-axis label: Days following first study drug intake. Use y-axis label: Cumulative probability of hospitalization. Display x-axis until Day 28. Time to hospitalization will be truncated after Day 28 in order to avoid a sudden drop in number of participants at risk. Also display the p-value of the log-rank test.

Footnotes:
Participants who did not experience the event of interest are considered censored according to the censoring rules detailed in the SAP.

Table 14.2.5.1 Summary of WHO Ordinal Scale for Clinical Improvement by treatment arm and overall (mITT Analysis Set)
(Page x of y)

Variable<br>Statistic	SOC<br>(N=xx)	ASAQ<br>(N=xx)	PA<br>(N=xx)	FPV-NTZ<br>(N=xx)	SOF/DCV<br>(N=xx)	Overall<br>(N=xx)
Maximum score of WHO Ordinal Scale for Clinical Improvement post-baseline						
n (%)	xx (xx.x)	xx (xx.x)	xx (xx.x)	xx (xx.x)	xx (xx.x)	xx (xx.x)
Mean	xx.xx	xx.xx	xx.xx	xx.xx	xx.xx	xx.xx
SD	xx.xxx	xx.xxx	xx.xxx	xx.xxx	xx.xxx	xx.xxx
Minimum	xx.x	xx.x	xx.x	xx.x	xx.x	xx.x
Q1	xx.xx	xx.xx	xx.xx	xx.xx	xx.xx	xx.xx
Median	xx.xx	xx.xx	xx.xx	xx.xx	xx.xx	xx.xx
Q3	xx.xx	xx.xx	xx.xx	xx.xx	xx.xx	xx.xx
Maximum	xx.x	xx.x	xx.x	xx.x	xx.x	xx.x
Worst WHO Ordinal Scale for Clinical Improvement post-baseline compared to baseline						
Improved	xx (xx.x)	xx (xx.x)	xx (xx.x)	xx (xx.x)	xx (xx.x)	xx (xx.x)
No change	xx (xx.x)	xx (xx.x)	xx (xx.x)	xx (xx.x)	xx (xx.x)	xx (xx.x)
Worsened	xx (xx.x)	xx (xx.x)	xx (xx.x)	xx (xx.x)	xx (xx.x)	xx (xx.x)
Missing	xx (xx.x)	xx (xx.x)	xx (xx.x)	xx (xx.x)	xx (xx.x)	xx (xx.x)

Percentages are based on the total number of participants in the corresponding treatment arm.

Table 14.2.5.2 Proportional odds model for disease severity for Day 7, 14, 21, and 28 (mITT Analysis Set)
(Page x of y)

Time point	Treatment arm	N	Comparison	Adjusted odds ratio	95% CI	p-value
Day 7						
	SOC	xx				
	ASAQ	xx	ASAQ / SOC	xx.x	(xx.x, xx.x)	x.xxx
	PA	xx	PA / SOC	xx.x	(xx.x, xx.x)	x.xxx
	FPV-NTZ	xx	FPV-NTZ / SOC	xx.x	(xx.x, xx.x)	x.xxx
	SOF/DCV	xx	SOF/DCV / SOC	xx.x	(xx.x, xx.x)	x.xxx
Day 14						
	SOC	xx				
	ASAQ	xx	ASAQ / SOC	xx.x	(xx.x, xx.x)	x.xxx
	PA	xx	PA / SOC	xx.x	(xx.x, xx.x)	x.xxx
	FPV-NTZ	xx	FPV-NTZ / SOC	xx.x	(xx.x, xx.x)	x.xxx
	SOF/DCV	xx	SOF/DCV / SOC	xx.x	(xx.x, xx.x)	x.xxx
Etc...						

N = number of participants in the respective treatment arm.
Disease severity is measured by WHO Ordinal Scale score for Clinical Improvement (ordered from 0<1<2<...<8).
The longitudinal proportional odds model includes time point, treatment and treatment-by-time point interaction as fixed-effect factors. Baseline age (in years), sex, baseline BMI, baseline WHO Ordinal Scale score, baseline comorbidities, baseline viral load category and days of symptoms at time of enrollment are included as covariates. Participant-specific intercepts will be included as random effects.
An adjusted odds ratio > 1 suggests a higher chance of more severe disease at the corresponding time point in the treatment arm of the numerator of the ratio.

Programming note: Display table for time points Day 7, Day 14, Day 21 and Day 28. If the condensed response variable needs to be used due to convergence issues of the original model, change footnote " Disease severity is measured by WHO Ordinal Scale score for Clinical Improvement (ordered from 0<1<2<...<8).*" to " Disease severity is measured by patient state (0<1<2<3<4).". , derived from WHO Ordinal Scale score for Clinical Improvement.".*

Table 14.2.5.3 Logistic regression for disease severity (mITT Analysis Set)
(Page x of y)

Treatment arm	N	n/M (%)	Comparison	Odds ratio	95% CI	p-value
SOC	xx	xx/xx (xx.x)				
ASAQ	xx	xx/xx (xx.x)	ASAQ / SOC	xx.x	(xx.x, xx.x)	x.xxx
PA	xx	xx/xx (xx.x)	PA / SOC	xx.x	(xx.x, xx.x)	x.xxx
FPV-NTZ	xx	xx/xx (xx.x)	FPV-NTZ / SOC	xx.x	(xx.x, xx.x)	x.xxx
SOF/DCV	xx	xx/xx (xx.x)	SOF/DCV / SOC	xx.x	(xx.x, xx.x)	x.xxx

N = number of participants in the respective treatment arm.

n = number of participants with WHO ordinal scale score worsening at any post-baseline time point by at least 1 category compared to baseline.

M = number of evaluable participants.

The logistic regression model contains treatment arm, age at baseline (years), sex, baseline BMI, baseline comorbidities, baseline viral load category and days of symptoms at time of enrollment as covariates.

An odds ratio > 1 suggests a higher chance of severe disease in the treatment arm of the numerator of the ratio.

Programming note: This analysis is only required if the proportional odds model from table 14.2.5.2 does not converge.

Table 14.2.5.4 Kaplan Meier analysis of time to first zero WHO Ordinal Scale score for Clinical Improvement (mITT Analysis Set)
(Page x of y)

Statistic	SOC (N=xx)	ASAQ (N=xx)	PA (N=xx)	FPV-NTZ (N=xx)	SOF/DCV (N=xx)
Available observations	xx	xx	xx	xx	xx
Number of participants with zero WHO Scale score	xx	xx	xx	xx	xx
Number of censored observations	xx	xx	xx	xx	xx
Zero scores over time (days)					
25th percentile (Two-sided 95% CI)	xx.x (xx.x, xx.x)	xx.x (xx.x, xx.x)	xx.x (xx.x, xx.x)	xx.x (xx.x, xx.x)	xx.x (xx.x, xx.x)
Median (Two-sided 95% CI)	xx.x (xx.x, xx.x)	xx.x (xx.x, xx.x)	xx.x (xx.x, xx.x)	xx.x (xx.x, xx.x)	xx.x (xx.x, xx.x)
75th percentile (Two-sided 95% CI)	xx.x (xx.x, xx.x)	xx.x (xx.x, xx.x)	xx.x (xx.x, xx.x)	xx.x (xx.x, xx.x)	xx.x (xx.x, xx.x)
% zero scores estimate (95% CI)					
Day 7	xx.x (xx.x, xx.x)	xx.x (xx.x, xx.x)	xx.x (xx.x, xx.x)	xx.x (xx.x, xx.x)	xx.x (xx.x, xx.x)
Day 14	xx.x (xx.x, xx.x)	xx.x (xx.x, xx.x)	xx.x (xx.x, xx.x)	xx.x (xx.x, xx.x)	xx.x (xx.x, xx.x)
Day 21	xx.x (xx.x, xx.x)	xx.x (xx.x, xx.x)	xx.x (xx.x, xx.x)	xx.x (xx.x, xx.x)	xx.x (xx.x, xx.x)
Day 28	xx.x (xx.x, xx.x)	xx.x (xx.x, xx.x)	xx.x (xx.x, xx.x)	xx.x (xx.x, xx.x)	xx.x (xx.x, xx.x)

Estimates are based on Kaplan-Meier methodology.
Participants who did not experience the event of interest are considered censored according to the censoring rules detailed in Appendix 16.1.9.
Time to first zero WHO Ordinal Scale score is defined as time to first zero score without any subsequent non-zero score.
Zero scores over time: presents the time point (in days) by which the estimated cumulative probability of a zero WHO Ordinal Scale score is 25%, 50% and 75% and the corresponding two-sided 95% confidence interval.
% zero score estimate: presents the estimated cumulative probability of a zero WHO Ordinal Scale score by Day 7/14/21/28 and the corresponding two-sided 95% confidence interval.
n.e. = not estimable.

Table 14.2.5.5 Cox regression analysis of time to first zero WHO Ordinal Scale score for Clinical Improvement (mITT Analysis Set)

Programming note: Use same layout as table 14.2.2.5.

Footnotes:
N = number of participants in the respective treatment arm.
n = number of participants with WHO Ordinal Scale score.
M = number of evaluable participants.
The cox regression model contains treatment arm, age at baseline (years), sex, baseline BMI, baseline comorbidities, baseline viral load category and days of symptoms at time of enrollment as covariates.
A hazard ratio > 1 favors the treatment arm in the numerator of the ratio (= higher probability of having zero WHO Ordinal Scale score).

Table 14.2.5.6 Summary of WHO Ordinal Scale for Clinical Improvement by treatment arm and overall (mITT Analysis Set – sero-negative participants)
(Page x of y)

Programming note: Use same layout as table 14.2.5.1. Produce table for mITT Analysis Set in the subset of sero-negative participants. Sero-negative participants can be selected using ADMB.PARAMCD='COVSER' and ADMB.ABLFL='Y' and ADMB.MITTFL='Y' and ADMB.AVAL=0.

Table 14.2.5.7 Summary of WHO Ordinal Scale for Clinical Improvement by treatment arm and overall (mITT Analysis Set – high-risk participants)
(Page x of y)

Programming note: Use same layout as table 14.2.5.1. Produce table for mITT Analysis Set in the subset of high-risk participants. High-risk participants can be selected using ADSL.HGRKCATN=2.

Table 14.2.5.8 Kaplan Meier analysis of time to first zero WHO Ordinal Scale score for Clinical Improvement (mITT Analysis Set – sero-negative participants)
(Page x of y)

Programming note: Use same layout as table 14.2.5.4. Produce table for mITT Analysis Set in the subset of sero-negative participants. Sero-negative participants can be selected using ADMB.PARAMCD='COVSER' and ADMB.ABLFL='Y' and ADMB.MITTFL='Y' and ADMB.AVAL=0.

Table 14.2.5.9 Kaplan Meier analysis of time to first zero WHO Ordinal Scale score for Clinical Improvement (mITT Analysis Set – high-risk participants)
(Page x of y)

Programming note: Use same layout as table 14.2.5.4. Produce table for mITT Analysis Set in the subset of high-risk participants. High-risk participants can be selected using ADSL.HGRKCATN=2.

Table 14.2.5.10 Cox regression analysis of time to first zero WHO Ordinal Scale score for Clinical Improvement (mITT Analysis Set – sero-negative participants)

Programming note: Use same layout as table 14.2.5.5. Produce table for mITT Analysis Set in the subset of sero-negative participants. Sero-negative participants can be selected using ADMB.PARAMCD='COVSER' and ADMB.ABLFL='Y' and ADMB.MITTFL='Y' and ADMB.AVAL=0.

Table 14.2.5.11 Cox regression analysis of time to first zero WHO Ordinal Scale score for Clinical Improvement (mITT Analysis Set – high-risk participants)

Programming note: Use same layout as table 14.2.5.5. Produce table for mITT Analysis Set in the subset of high-risk participants. High-risk participants can be selected using ADSL.HGRKCATN=2.

Figure 14.2.5.1 Kaplan-Meier curves for time to first zero WHO Ordinal Scale score for Clinical Improvement (mITT Analysis set)

Programming note: Use same figure layout as figure 14.2.2.1. Display the following treatment arms: SOC, ASAQ, PA, FPV-NTZ, SOF/DCV. Use different colors and line styles to differentiate between survival curves. Use x-axis label: Days following first study drug intake. Use y-axis label: Cumulative probability of zero WHO Ordinal Scale score. Display x-axis until Day 28. Time to first zero WHO Ordinal Scale score will be truncated after Day 28 in order to avoid a sudden drop in number of participants at risk. Also display the p-value of the log-rank test.

Footnotes:
Participants who did not experience the event of interest are considered censored according to the censoring rules detailed in the SAP.

Table 14.2.6.1 Poisson regression for proportion of days with fever after randomization (mITT Analysis Set)
(Page x of y)

Treatment arm	N	No. of days with fever	No. of days observation	Raw rate	Rate estimate	95% CI	Comparison	Rate ratio	95% CI	p-value
SOC	xx	xx	xx	xx.x	xx.x	(xx.x, xx.x)				
ASAQ	xx	xx	xx	xx.x	xx.x	(xx.x, xx.x)	ASAQ / SOC	xx.x	(xx.x, xx.x)	x.xxx
PA	xx	xx	xx	xx.x	xx.x	(xx.x, xx.x)	PA / SOC	xx.x	(xx.x, xx.x)	x.xxx
FPV-NTZ	xx	xx	xx	xx.x	xx.x	(xx.x, xx.x)	FPV-NTZ / SOC	xx.x	(xx.x, xx.x)	x.xxx
SOF/DCV	xx	xx	xx	xx.x	xx.x	(xx.x, xx.x)	SOF/DCV / SOC	xx.x	(xx.x, xx.x)	x.xxx

N = number of participants in the respective treatment arm.
Raw rate is calculated as the number of days with fever / number of days of observation x 100.
Rate estimate is derived by means of a Poisson regression model, adjusted to a 100 day period.
The Poisson regression model contains treatment arm, age at baseline (years), sex, baseline BMI, baseline comorbidities, baseline viral load category and days of symptoms at time of enrollment as covariates. For each participant the number of days of observation (i.e. days where temperature is non-missing) is used as offset.

Table 14.2.6.2 Poisson regression for proportion of days with respiratory symptoms after randomization (mITT Analysis Set)
(Page x of y)

Treatment arm	N	No. of days with respiratory symptoms	No. of days observation	Raw rate	Rate estimate	95% CI	Comparison	Rate ratio	95% CI	p-value
SOC	xx	xx	xx	xx.x	xx.x	(xx.x, xx.x)				
ASAQ	xx	xx	xx	xx.x	xx.x	(xx.x, xx.x)	ASAQ / SOC	xx.x	(xx.x, xx.x)	x.xxx
PA	xx	xx	xx	xx.x	xx.x	(xx.x, xx.x)	PA / SOC	xx.x	(xx.x, xx.x)	x.xxx
FPV-NTZ	xx	xx	xx	xx.x	xx.x	(xx.x, xx.x)	FPV-NTZ / SOC	xx.x	(xx.x, xx.x)	x.xxx
SOF/DCV	xx	xx	xx	xx.x	xx.x	(xx.x, xx.x)	SOF/DCV / SOC	xx.x	(xx.x, xx.x)	x.xxx

N = number of participants in the respective treatment arm.
Raw rate is calculated as the number of days with respiratory symptoms / number of days of observation x 100.
Rate estimate is derived by means of a Poisson regression model, adjusted to a 100 day period.
The Poisson regression model contains treatment arm, age at baseline (years), sex, baseline BMI, baseline comorbidities, baseline viral load category and days of symptoms at time of enrollment as covariates. For each participant the number of days of observation (i.e. days where chest/respiratory FLU-PRO domain score is non-missing) is used as offset.
Respiratory symptom is defined as a chest/respiratory FLU-PRO domain score >0.

Table 14.2.6.3 Poisson regression for proportion of days with SpO2<93% after randomization (mITT Analysis Set)
(Page x of y)

Type of analysis	Treatment arm	N	m	No. of days with SpO2<93%	No. of days observation	Raw rate	Rate estimate	95% CI	Comparison	Rate ratio	95% CI	p-value
All SpO2 values												
	SOC	xx	xx	xx	xx	xx.x	xx.x	(xx.x, xx.x)				
	ASAQ	xx	xx	xx	xx	xx.x	xx.x	(xx.x, xx.x)	ASAQ / SOC	xx.x	(xx.x, xx.x)	x.xxx
	PA	xx	xx	xx	xx	xx.x	xx.x	(xx.x, xx.x)	PA / SOC	xx.x	(xx.x, xx.x)	x.xxx
	FPV-NTZ	xx	xx	xx	xx	xx.x	xx.x	(xx.x, xx.x)	FPV-NTZ / SOC	xx.x	(xx.x, xx.x)	x.xxx
	SOF/DCV	xx	xx	xx	xx	xx.x	xx.x	(xx.x, xx.x)	SOF/DCV / SOC	xx.x	(xx.x, xx.x)	x.xxx
Restricted to plausible SpO2 values*												
	SOC	xx	xx	xx	xx	xx.x	xx.x	(xx.x, xx.x)				
	ASAQ	xx	xx	xx	xx	xx.x	xx.x	(xx.x, xx.x)	ASAQ / SOC	xx.x	(xx.x, xx.x)	x.xxx
	PA	xx	xx	xx	xx	xx.x	xx.x	(xx.x, xx.x)	PA / SOC	xx.x	(xx.x, xx.x)	x.xxx
	FPV-NTZ	xx	xx	xx	xx	xx.x	xx.x	(xx.x, xx.x)	FPV-NTZ / SOC	xx.x	(xx.x, xx.x)	x.xxx
	SOF/DCV	xx	xx	xx	xx	xx.x	xx.x	(xx.x, xx.x)	SOF/DCV / SOC	xx.x	(xx.x, xx.x)	x.xxx

N = number of participants in the respective treatment arm.
m is the number of participants in the mITT Analysis Set of the corresponding treatment arm that have SpO2 >=95% on baseline.
Raw rate is calculated as the number of days with SpO2<93% / number of days of observation x 100.
Rate estimate is derived by means of a Poisson regression model, adjusted to a 100 day period.
The Poisson regression model contains treatment arm, age at baseline (years), sex, baseline BMI, baseline comorbidities, baseline viral load category and days of symptoms at time of enrollment as covariates. For each participant the number of days of observation (i.e. days where SpO2<93% is non-missing) is used as offset.
The analysis includes only participants that had SpO2 >=95% on baseline.
*Only plausible SpO2 values are used in the analysis. Days without any plausible SpO2 value are not counted towards no. of days of observation.

Table 14.2.6.4 Summary of FLU-PRO Plus questionnaire scores and changes from baseline by treatment arm and overall (mITT Analysis Set)
(Page x of y)

Score: Domain score - Nose

Time point<br>Statistic	SOC (N=xx)			ASAQ (N=xx)			PA (N=xx)		
	Base	Post	Change	Base	Post	Change	Base	Post	Change
Baseline									
n (%)	xx (xx.x)			xx (xx.x)			xx (xx.x)		
Mean	xx.xx			xx.xx			xx.xx		
SD	xx.xxx			xx.xxx			xx.xxx		
Minimum	xx.x			xx.x			xx.x		
Q1	xx.xx			xx.xx			xx.xx		
Median	xx.xx			xx.xx			xx.xx		
Q3	xx.xx			xx.xx			xx.xx		
Maximum	xx.x			xx.x			xx.x		
Day 2									
n (%)	xx (xx.x)	xx (xx.x)	xx xx.x)	xx (xx.x)	xx (xx.x)	xx (xx.x)	xx (xx.x)	xx (xx.x)	xx (xx.x)
Mean	xx.xx	xx.xx	xx.xx	xx.xx	xx.xx	xx.xx	xx.xx	xx.xx	xx.xx
SD	xx.xxx	xx.xxx	xx.xxx	xx.xxx	xx.xxx	xx.xxx	xx.xxx	xx.xxx	xx.xxx
Minimum	xx.x	xx.x	xx.x	xx.x	xx.x	xx.x	xx.x	xx.x	xx.x
Q1	xx.xx	xx.xx	xx.xx	xx.xx	xx.xx	xx.xx	xx.xx	xx.xx	xx.xx
Median	xx.xx	xx.xx	xx.xx	xx.xx	xx.xx	xx.xx	xx.xx	xx.xx	xx.xx
Q3	xx.xx	xx.xx	xx.xx	xx.xx	xx.xx	xx.xx	xx.xx	xx.xx	xx.xx
Maximum	xx.x	xx.x	xx.x	xx.x	xx.x	xx.x	xx.x	xx.x	xx.x
Day 3									
etc...									

Base = Baseline, Post = Post-baseline, Change = Post-baseline - baseline.
At each visit, only participants with a value at both baseline and the respective day are included.

Table 14.2.6.4 Summary of FLU-PRO Plus questionnaire scores and changes from baseline by treatment arm and overall (mITT Analysis Set)

(Page x of y)

Score: Domain score - Nose

Time point<br>Statistic	FPV-NTZ (N=xx)			SOF/DCV (N=xx)			Overall (N=xx)		
	Base	Post	Change	Base	Post	Change	Base	Post	Change
Baseline									
n (%)	xx (xx.x)			xx (xx.x)			xx (xx.x)		
Mean	xx.xx			xx.xx			xx.xx		
SD	xx.xxx			xx.xxx			xx.xxx		
Minimum	xx.x			xx.x			xx.x		
Q1	xx.xx			xx.xx			xx.xx		
Median	xx.xx			xx.xx			xx.xx		
Q3	xx.xx			xx.xx			xx.xx		
Maximum	xx.x			xx.x			xx.x		
Day 2									
n (%)	xx (xx.x)	xx (xx.x)	xx xx.x)	xx (xx.x)	xx (xx.x)	xx (xx.x)	xx (xx.x)	xx (xx.x)	xx (xx.x)
Mean	xx.xx	xx.xx	xx.xx	xx.xx	xx.xx	xx.xx	xx.xx	xx.xx	xx.xx
SD	xx.xxx	xx.xxx	xx.xxx	xx.xxx	xx.xxx	xx.xxx	xx.xxx	xx.xxx	xx.xxx
Minimum	xx.x	xx.x	xx.x	xx.x	xx.x	xx.x	xx.x	xx.x	xx.x
Q1	xx.xx	xx.xx	xx.xx	xx.xx	xx.xx	xx.xx	xx.xx	xx.xx	xx.xx
Median	xx.xx	xx.xx	xx.xx	xx.xx	xx.xx	xx.xx	xx.xx	xx.xx	xx.xx
Q3	xx.xx	xx.xx	xx.xx	xx.xx	xx.xx	xx.xx	xx.xx	xx.xx	xx.xx
Maximum	xx.x	xx.x	xx.x	xx.x	xx.x	xx.x	xx.x	xx.x	xx.x
Day 3									
etc...									

Base = Baseline, Post = Post-baseline, Change = Post-baseline - baseline.
At each visit, only participants with a value at both baseline and the respective day are included.

Programming note: Present this table for time points Baseline, Day 2, Day 3, ..., Day 13, Day 14, Day 21, Day 28 and End of study and for each domain score and the overall score, i.e. Domain score – Nose, Domain score – Throat, Domain score – Eyes, Domain score – Chest/Respiratory, Domain score – Gastrointestinal, Domain Score – Body/Systemic and Total score.

Table 14.2.7.1 Cumulative incidence of lower respiratory tract infection by treatment arm and overall (mITT Analysis Set)
(Page x of y)

Type of analysis<br>Variable	SOC (N=xx)		ASAQ (N=xx)		PA (N=xx)		FPV-NTZ (N=xx)		SOF/DCV (N=xx)		Overall (N=xx)	
	n/m (%)	95% CI	n/m (%)	95% CI	n/m (%)	95% CI	n/m (%)	95% CI	n/m (%)	95% CI	n/m (%)	95% CI
All SpO2 values												
Cumulative incidence of LRTI at Day 28	xx/xx (xx.x)	(xx.x, xx.x)	xx/xx (xx.x)	(xx.x, xx.x)	xx/xx (xx.x)	(xx.x, xx.x)	xx/xx (xx.x)	(xx.x, xx.x)	xx/xx (xx.x)	(xx.x, xx.x)	xx/xx (xx.x)	(xx.x, xx.x)
Restricted to plausible SpO2 values*												
Cumulative incidence of LRTI at Day 28	xx/xx (xx.x)	(xx.x, xx.x)	xx/xx (xx.x)	(xx.x, xx.x)	xx/xx (xx.x)	(xx.x, xx.x)	xx/xx (xx.x)	(xx.x, xx.x)	xx/xx (xx.x)	(xx.x, xx.x)	xx/xx (xx.x)	(xx.x, xx.x)

Lower respiratory tract infection (LRTI) is defined by resting SpO2<93% sustained for 2 readings 2 hours apart and presence of either subjective dyspnea or cough (or both). It is only defined for participants with SpO2 ≥95% on Day 1.
m is the number of participants in the mITT Analysis Set of the corresponding treatment arm that have SpO2 ≥95% on Day 1.
The cumulative incidence of LRTI at Day 28 is defined by the number of participants developing LRTI up to and including Day 28 divided by the number of participants in the mITT Analysis Set that had SpO2 ≥95% on Day 1.
Two-sided 95% confidence intervals are calculated according to the Wilson method without continuity correction.
*Only plausible SpO2 values are used in the analysis.

Table 14.2.8.1 Cumulative incidence of all-cause mortality by treatment arm and overall (mITT Analysis Set)
(Page x of y)

Variable	SOC (N=xx)		ASAQ (N=xx)		PA (N=xx)		FPV-NTZ (N=xx)		SOF/DCV (N=xx)		Overall (N=xx)	
	n (%)	95% CI	n (%)	95% CI	n (%)	95% CI	n (%)	95% CI	n (%)	95% CI	n (%)	95% CI
Cumulative incidence of all-cause mortality at Day 28	xx (xx.x)	(xx.x, xx.x)	xx (xx.x)	(xx.x, xx.x)	xx (xx.x)	(xx.x, xx.x)	xx (xx.x)	(xx.x, xx.x)	xx (xx.x)	(xx.x, xx.x)	xx (xx.x)	(xx.x, xx.x)

The cumulative incidence of all-cause mortality at Day 28 defined by the number of participants who died up to and including Day 28 divided by the number of participants in the mITT Analysis Set will be presented. Participants who are hospitalized at the time of Day 28 and die later also count to the numerator.
The two-sided 95% confidence intervals are calculated according to the Wilson method without continuity correction.

Table 14.3.1.1 Compliance by treatment arm (Safety Analysis Set)
(Page x of y)

Variable<br>Statistic	ASAQ<br>(N=xx)	PA<br>(N=xx)	FPV-NTZ<br>(N=xx)		SOF/DCV<br>(N=xx)
			FPV	NTZ	
Compliance					
n (%)	xx (xx.x)	xx (xx.x)	xx (xx.x)	xx (xx.x)	xx (xx.x)
Mean	xx.xx	xx.xx	xx.xx	xx.xx	xx.xx
SD	xx.xxx	xx.xxx	xx.xxx	xx.xxx	xx.xxx
Minimum	xx.x	xx.x	xx.x	xx.x	xx.x
Q1	xx.xx	xx.xx	xx.xx	xx.xx	xx.xx
Median	xx.xx	xx.xx	xx.xx	xx.xx	xx.xx
Q3	xx.xx	xx.xx	xx.xx	xx.xx	xx.xx
Maximum	xx.x	xx.x	xx.x	xx.x	xx.x
Compliance category, n (%)					
<85%	xx (xx.x)	xx (xx.x)	xx (xx.x)	xx (xx.x)	xx (xx.x)
>=85%	xx (xx.x)	xx (xx.x)	xx (xx.x)	xx (xx.x)	xx (xx.x)
>=85% and loading dose complete*			xx (xx.x)		

The compliance is only calculated and displayed for the experimental treatments.
* Only applicable to FPV. Refers to the participant taking the full loading dose on Day 1.
Analyses is performed according to actual treatment.

Table 14.3.2.1 Concomitant medications by treatment arm and overall, ATC class and WHO preferred term (Safety Analysis Set)

Programming note: Same layout as table 14.1.8. Replace prior with concomitant in the footnotes. Use ATC class 3.

Table 14.3.3.1 Summary of treatment emergent adverse events by MedDRA primary system organ class and preferred term by treatment arm and overall (Safety Analysis Set)
(Page x of y)

Primary system organ class<br>Preferred term	SOC<br>(N=xx)<br>n (%) /# events	ASAQ<br>(N=xx)<br>n (%) /# events	PA<br>(N=xx)<br>n (%) /# events	FPV-NTZ<br>(N=xxx)<br>n (%) /# events	SOF/DCV<br>(N=xxx)<br>n (%) /# events	Overall<br>(N=xxx)<br>n (%) /# events
At least one adverse event	xx (xx.x) / xx	xx (xx.x) / xx	xx (xx.x) / xx	xx (xx.x) / xx	xx (xx.x) / xx	xx (xx.x) / xx
Primary system organ class 1	xx (xx.x) / xx	xx (xx.x) / xx	xx (xx.x) / xx	xx (xx.x) / xx	xx (xx.x) / xx	xx (xx.x) / xx
Preferred term 1	xx (xx.x) / xx	xx (xx.x) / xx	xx (xx.x) / xx	xx (xx.x) / xx	xx (xx.x) / xx	xx (xx.x) / xx
Preferred term 2	xx (xx.x) / xx	xx (xx.x) / xx	xx (xx.x) / xx	xx (xx.x) / xx	xx (xx.x) / xx	xx (xx.x) / xx
Primary system organ class 2	xx (xx.x) / xx	xx (xx.x) / xx	xx (xx.x) / xx	xx (xx.x) / xx	xx (xx.x) / xx	xx (xx.x) / xx
Preferred term 1	xx (xx.x) / xx	xx (xx.x) / xx	xx (xx.x) / xx	xx (xx.x) / xx	xx (xx.x) / xx	xx (xx.x) / xx
Preferred term 2	xx (xx.x) / xx	xx (xx.x) / xx	xx (xx.x) / xx	xx (xx.x) / xx	xx (xx.x) / xx	xx (xx.x) / xx
etc.						

N = number of participants in the Safety Analysis set.
n = number of participants with at least one event.
Analyses is performed according to actual treatment.
A participant with more than one adverse events within a primary system organ class is counted only once for that class.
events: number of events
MedDRA version xx.x has been used for coding.

Programming note: Sort primary system organ classes alphabetically. Sort preferred terms within system organ classes by descending frequency in the overall group.

Table 14.3.3.2 Summary of serious treatment emergent adverse events by MedDRA primary system organ class and preferred term by treatment arm and overall (Safety Analysis Set)
(Page x of y)

Programming note: Same layout as table 14.3.3.1.

Table 14.3.3.3 Summary of treatment emergent adverse events considered to be study drug related by MedDRA primary system organ class and preferred term by treatment arm and overall (Safety Analysis Set)
(Page x of y)

Programming note: Same layout as table 14.3.3.1.

Table 14.3.3.4 Summary of serious treatment emergent adverse events considered to be study drug related by MedDRA primary system organ class and preferred term by treatment arm and overall (Safety Analysis Set)
(Page x of y)

Programming note: Same layout as table 14.3.3.1.

Table 14.3.3.5 Summary of treatment emergent adverse events leading to study drug discontinuation by MedDRA primary system organ class and preferred term by treatment arm and overall (Safety Analysis Set)
(Page x of y)

Programming note: Same layout as table 14.3.3.1.

Table 14.3.3.6 Summary of serious treatment emergent adverse events leading to study drug discontinuation by MedDRA primary system organ class and preferred term by treatment arm and overall (Safety Analysis Set)
(Page x of y)

Programming note: Same layout as table 14.3.3.1.

Table 14.3.3.7 Summary of treatment emergent adverse events leading to death by MedDRA primary system organ class and preferred term by treatment arm and overall (Safety Analysis Set)
(Page x of y)

Programming note: Same layout as table 14.3.3.1.

Table 14.3.3.8 Summary of treatment emergent adverse events leading to hospitalization by MedDRA primary system organ class and preferred term by treatment arm and overall (Safety Analysis Set)
(Page x of y)

Programming note: Same layout as table 14.3.3.1.

Table 14.3.3.9 Summary of treatment emergent adverse events by MedDRA primary system organ class, preferred term, maximal severity and treatment arm and overall (Safety Analysis Set)
(Page x of y)

Primary system organ class	Preferred term	Maximal severity	SOC (N=xx) n (%) /# events	ASAQ (N=xx) n (%)/# events	PA (N=xx) n (%)/# events	FPV-NTZ (N=xx) n (%)/# events	SOF/DCV (N=xx) n (%)/# events	Overall (N=xx) n (%)/# events
At least one AE	All PTs	Any grade	xxx (xx.x) / xx	xxx (xx.x) / xx	xxx (xx.x) / xx	xxx (xx.x) / xx	xxx (xx.x) / xx	xxx (xx.x) / xx
		Grade 1	xxx (xx.x) / xx	xxx (xx.x) / xx	xxx (xx.x) / xx	xxx (xx.x) / xx	xxx (xx.x) / xx	xxx (xx.x) / xx
		Grade 2	xxx (xx.x) / xx	xxx (xx.x) / xx	xxx (xx.x) / xx	xxx (xx.x) / xx	xxx (xx.x) / xx	xxx (xx.x) / xx
		Grade 3	xxx (xx.x) / xx	xxx (xx.x) / xx	xxx (xx.x) / xx	xxx (xx.x) / xx	xxx (xx.x) / xx	xxx (xx.x) / xx
		Grade 4	xxx (xx.x) / xx	xxx (xx.x) / xx	xxx (xx.x) / xx	xxx (xx.x) / xx	xxx (xx.x) / xx	xxx (xx.x) / xx
		Grade 5	xxx (xx.x) / xx	xxx (xx.x) / xx	xxx (xx.x) / xx	xxx (xx.x) / xx	xxx (xx.x) / xx	xxx (xx.x) / xx
Primary SOC	All PTs	Any grade	xxx (xx.x) / xx	xxx (xx.x) / xx	xxx (xx.x) / xx	xxx (xx.x) / xx	xxx (xx.x) / xx	xxx (xx.x) / xx
		Grade 1	xxx (xx.x) / xx	xxx (xx.x) / xx	xxx (xx.x) / xx	xxx (xx.x) / xx	xxx (xx.x) / xx	xxx (xx.x) / xx
	Preferred term 1	Total	xxx (xx.x) / xx	xxx (xx.x) / xx	xxx (xx.x) / xx	xxx (xx.x) / xx	xxx (xx.x) / xx	xxx (xx.x) / xx
		etc.						

N: number of participants in the Safety Analysis Set.
n: number of participants with at least one event.
If a participant reported more than one adverse event within the same category, the worst severity was summarized. A participant with more than one adverse event within a primary system organ class is counted only once for that class.
events: For Grade 1 to 5 only the events with the worst severity per patient are counted. For Any Grade all events are counted.
MedDRA version xx.x has been used for coding.

Programming note: Sort primary system organ classes alphabetically. Sort preferred terms within system organ classes by descending frequency in the overall group.

Table 14.3.3.10 Non-serious adverse events (threshold >5%) by MedDRA primary system organ class, preferred term and treatment arm (Safety Analysis Set)
(Page x of y)

MedDRA version xx.x has been used for coding.

Total number of patients affected by non-serious AE are those patients who had at least one PT that met the threshold criteria. Preferred terms with a frequency greater than 5% in any treatment arm were printed.

Only non-serious treatment emergent adverse events are included.

Programming note: Same layout as table 14.3.3.1.

Table 14.3.4.1 Summary of vital signs including changes from baseline by treatment arm and overall and time point (Safety Analysis Set)
(Page x of y)

Parameter: Oral temperature (degrees C)

Time point<br>Statistic	SOC<br>(N=xx)			ASAQ<br>(N=xx)			PA<br>(N=xx)		
	Base	Post	Change	Base	Post	Change	Base	Post	Change
Baseline									
n (%)	xx (xx.x)			xx (xx.x)			xx (xx.x)		
Mean	xx.xx			xx.xx			xx.xx		
SD	xx.xxx			xx.xxx			xx.xxx		
Minimum	xx.x			xx.x			xx.x		
Q1	xx.xx			xx.xx			xx.xx		
Median	xx.xx			xx.xx			xx.xx		
Q3	xx.xx			xx.xx			xx.xx		
Maximum	xx.x			xx.x			xx.x		
Day 2									
n (%)	xx (xx.x)	xx (xx.x)	xx xx.x)	xx (xx.x)	xx (xx.x)	xx (xx.x)	xx (xx.x)	xx (xx.x)	xx (xx.x)
Mean	xx.xx	xx.xx	xx.xx	xx.xx	xx.xx	xx.xx	xx.xx	xx.xx	xx.xx
SD	xx.xxx	xx.xxx	xx.xxx	xx.xxx	xx.xxx	xx.xxx	xx.xxx	xx.xxx	xx.xxx
Minimum	xx.x	xx.x	xx.x	xx.x	xx.x	xx.x	xx.x	xx.x	xx.x
Q1	xx.xx	xx.xx	xx.xx	xx.xx	xx.xx	xx.xx	xx.xx	xx.xx	xx.xx
Median	xx.xx	xx.xx	xx.xx	xx.xx	xx.xx	xx.xx	xx.xx	xx.xx	xx.xx
Q3	xx.xx	xx.xx	xx.xx	xx.xx	xx.xx	xx.xx	xx.xx	xx.xx	xx.xx
Maximum	xx.x	xx.x	xx.x	xx.x	xx.x	xx.x	xx.x	xx.x	xx.x
Day 3									
etc...									

Base = Baseline, Post = Post-baseline, Change = Post-baseline - baseline.
At each visit, only participants with a value at both baseline and the respective day are included.

Table 14.3.4.1 Summary of vital signs including changes from baseline by treatment arm and overall and time point (Safety Analysis Set)

(Page x of y)

Parameter: Oral temperature (degrees C)

Time point<br>Statistic	FPV-NTZ (N=xx)			SOF/DCV (N=xx)			Overall (N=xx)		
	Base	Post	Change	Base	Post	Change	Base	Post	Change
Baseline									
n (%)	xx (xx.x)			xx (xx.x)			xx (xx.x)		
Mean	xx.xx			xx.xx			xx.xx		
SD	xx.xxx			xx.xxx			xx.xxx		
Minimum	xx.x			xx.x			xx.x		
Q1	xx.xx			xx.xx			xx.xx		
Median	xx.xx			xx.xx			xx.xx		
Q3	xx.xx			xx.xx			xx.xx		
Maximum	xx.x			xx.x			xx.x		
Day 2									
n (%)	xx (xx.x)	xx (xx.x)	xx xx.x)	xx (xx.x)	xx (xx.x)	xx (xx.x)	xx (xx.x)	xx (xx.x)	xx (xx.x)
Mean	xx.xx	xx.xx	xx.xx	xx.xx	xx.xx	xx.xx	xx.xx	xx.xx	xx.xx
SD	xx.xxx	xx.xxx	xx.xxx	xx.xxx	xx.xxx	xx.xxx	xx.xxx	xx.xxx	xx.xxx
Minimum	xx.x	xx.x	xx.x	xx.x	xx.x	xx.x	xx.x	xx.x	xx.x
Q1	xx.xx	xx.xx	xx.xx	xx.xx	xx.xx	xx.xx	xx.xx	xx.xx	xx.xx
Median	xx.xx	xx.xx	xx.xx	xx.xx	xx.xx	xx.xx	xx.xx	xx.xx	xx.xx
Q3	xx.xx	xx.xx	xx.xx	xx.xx	xx.xx	xx.xx	xx.xx	xx.xx	xx.xx
Maximum	xx.x	xx.x	xx.x	xx.x	xx.x	xx.x	xx.x	xx.x	xx.x
Day 3									
etc...									

Base = Baseline, Post = Post-baseline, Change = Post-baseline - baseline.
At each visit, only participants with a value at both baseline and the respective day are included.

Programming note: Present table for time points Day 1, Day 2, Day 3, ..., Day 13, Day 14, Day 21, Day 28 and End of study. Display table for parameter Oral temperature (degrees C), Pulse (beats/min), Respiratory rate (breaths/min) and SpO2 (%).

Table 14.3.5.1 Shift table of serology by treatment arm (mITT Analysis Set)
(Page x of y)

Treatment	Baseline			Day 28 Negative		Day 28 Positive		Day 28 Missing		Day 28 Total	
	Result	n	(%)	n	(%)	n	(%)	n	(%)	n	(%)
SOC (N=xx)	Negative	xx	(xx.x)	xx	(xx.x)	xx	(xx.x)	xx	(xx.x)	xx	(xx.x)
	Positive	xx	(xx.x)	xx	(xx.x)	xx	(xx.x)	xx	(xx.x)	xx	(xx.x)
	Missing	xx	(xx.x)	xx	(xx.x)	xx	(xx.x)	xx	(xx.x)	xx	(xx.x)
	Total	xx	(xx.x)	xx	(xx.x)	xx	(xx.x)	xx	(xx.x)	xx	(xx.x)
ASAQ (N=xx)	Negative	xx	(xx.x)	xx	(xx.x)	xx	(xx.x)	xx	(xx.x)	xx	(xx.x)
	Positive	xx	(xx.x)	xx	(xx.x)	xx	(xx.x)	xx	(xx.x)	xx	(xx.x)
	Missing	xx	(xx.x)	xx	(xx.x)	xx	(xx.x)	xx	(xx.x)	xx	(xx.x)
	Total	xx	(xx.x)	xx	(xx.x)	xx	(xx.x)	xx	(xx.x)	xx	(xx.x)
PA (N=xx)	Negative	xx	(xx.x)	xx	(xx.x)	xx	(xx.x)	xx	(xx.x)	xx	(xx.x)
	Positive	xx	(xx.x)	xx	(xx.x)	xx	(xx.x)	xx	(xx.x)	xx	(xx.x)
	Missing	xx	(xx.x)	xx	(xx.x)	xx	(xx.x)	xx	(xx.x)	xx	(xx.x)
	Total	xx	(xx.x)	xx	(xx.x)	xx	(xx.x)	xx	(xx.x)	xx	(xx.x)
FPV-NTZ (N=xx)	Negative	xx	(xx.x)	xx	(xx.x)	xx	(xx.x)	xx	(xx.x)	xx	(xx.x)
	Positive	xx	(xx.x)	xx	(xx.x)	xx	(xx.x)	xx	(xx.x)	xx	(xx.x)
	Missing	xx	(xx.x)	xx	(xx.x)	xx	(xx.x)	xx	(xx.x)	xx	(xx.x)
	Total	xx	(xx.x)	xx	(xx.x)	xx	(xx.x)	xx	(xx.x)	xx	(xx.x)
SOF/DCV (N=xx)	Negative	xx	(xx.x)	xx	(xx.x)	xx	(xx.x)	xx	(xx.x)	xx	(xx.x)
	Positive	xx	(xx.x)	xx	(xx.x)	xx	(xx.x)	xx	(xx.x)	xx	(xx.x)
	Missing	xx	(xx.x)	xx	(xx.x)	xx	(xx.x)	xx	(xx.x)	xx	(xx.x)
	Total	xx	(xx.x)	xx	(xx.x)	xx	(xx.x)	xx	(xx.x)	xx	(xx.x)

Percentage is based on baseline total N for each treatment arm.

Table 14.3.5.2 Summary of PK parameter by treatment arm and overall (PK Analysis Set)
(Page x of y)

Parameter<br>Statistic	ASAQ<br>(N=xx)				PA<br>(N=xx)		
	Artesunate	Dihydroarte misinin	Amodiaquine	N-Desethyl Amodiaquine	Pyronaridine	Artesunate	Dihydroartem isinin
Cday3 (ng/mL)							
n (%)	xx (xx.x)	xx (xx.x)	xx (xx.x)	xx (xx.x)	xx (xx.x)	xx (xx.x)	xx (xx.x)
Mean	xx.xx	xx.xx	xx.xx	xx.xx	xx.xx	xx.xx	xx.xx
Geometric mean	xx.xx	xx.xx	xx.xx	xx.xx	xx.xx	xx.xx	xx.xx
SD	xx.xxx	xx.xxx	xx.xxx	xx.xxx	xx.xxx	xx.xxx	xx.xxx
Minimum	xx.x	xx.x	xx.x	xx.x	xx.x	xx.x	xx.x
Q1	xx.xx	xx.xx	xx.xx	xx.xx	xx.xx	xx.xx	xx.xx
Median	xx.xx	xx.xx	xx.xx	xx.xx	xx.xx	xx.xx	xx.xx
Q3	xx.xx	xx.xx	xx.xx	xx.xx	xx.xx	xx.xx	xx.xx
Maximum	xx.x	xx.x	xx.x	xx.x	xx.x	xx.x	xx.x
Cday7 (ng/mL)							
n (%)	xx (xx.x)	xx (xx.x)	xx (xx.x)	xx (xx.x)	xx xx.x)	xx (xx.x)	xx (xx.x)
Mean	xx.xx	xx.xx	xx.xx	xx.xx	xx.xx	xx.xx	xx.xx
Geometric mean	xx.xx	xx.xx	xx.xx	xx.xx	xx.xx	xx.xx	xx.xx
SD	xx.xxx	xx.xxx	xx.xxx	xx.xxx	xx.xxx	xx.xxx	xx.xxx
Minimum	xx.x	xx.x	xx.x	xx.x	xx.x	xx.x	xx.x
Q1	xx.xx	xx.xx	xx.xx	xx.xx	xx.xx	xx.xx	xx.xx
Median	xx.xx	xx.xx	xx.xx	xx.xx	xx.xx	xx.xx	xx.xx
Q3	xx.xx	xx.xx	xx.xx	xx.xx	xx.xx	xx.xx	xx.xx
Maximum	xx.x	xx.x	xx.x	xx.x	xx.x	xx.x	xx.x

PK analysis is only done for experimental arms, hence the SOC arm is not displayed.
Only for ASAQ and PA arms PK assessments were done at Day 3 additionally.
Concentrations below the lower limit of quantification (LLOQ) were imputed with LLOQ/2.
No visit windowing approach was applied.

Table 14.3.5.2 Summary of PK parameter by treatment arm and overall (PK Analysis Set)
(Page x of y)

Parameter<br>Statistic	FPV-NTZ (N=xx)			SOF/DCV (N=xx)	
	Favipiravir	Nitazoxanide	Tizoxanide	Sofosbuvir	Daclatasvir
Cday3 (ng/mL)					
n (%)					
Mean					
Geometric mean					
SD					
Minimum					
Q1					
Median					
Q3					
Maximum					
Cday7 (ng/mL)					
n (%)	xx (xx.x)	xx (xx.x)	xx (xx.x)	xx xx.x)	xx (xx.x)
Mean	xx.xx	xx.xx	xx.xx	xx.xx	xx.xx
Geometric mean	xx.xx	xx.xx	xx.xx	xx.xx	xx.xx
SD	xx.xxx	xx.xxx	xx.xxx	xx.xxx	xx.xxx
Minimum	xx.x	xx.x	xx.x	xx.x	xx.x
Q1	xx.xx	xx.xx	xx.xx	xx.xx	xx.xx
Median	xx.xx	xx.xx	xx.xx	xx.xx	xx.xx
Q3	xx.xx	xx.xx	xx.xx	xx.xx	xx.xx
Maximum	xx.x	xx.x	xx.x	xx.x	xx.x

PK analysis is only done for experimental arms, hence the SOC arm is not displayed.
Only for ASAQ and PA arms PK assessments were done at Day 3 additionally.
Concentrations below the lower limit of quantification (LLOQ) were imputed with LLOQ/2.
No visit windowing approach was applied.

Table 14.3.5.3 Incidence of low viral load on Day 7 (mITT Analysis Set)

Treatment arm	N	n/M (%)	Comparison*	Risk ratio	95% CI	p-value
SOC	xx	xx/xx (xx.x)				
ASAQ	xx	xx/xx (xx.x)	ASAQ / SOC	xx.x	(xx.x, xx.x)	x.xxx
PA	xx	xx/xx (xx.x)	PA / SOC	xx.x	(xx.x, xx.x)	x.xxx
FPV-NTZ	xx	xx/xx (xx.x)	FPV-NTZ / SOC	xx.x	(xx.x, xx.x)	x.xxx
SOF/DCV	xx	xx/xx (xx.x)	SOF/DCV / SOC	xx.x	(xx.x, xx.x)	x.xxx

N = number of participants in the respective treatment arm.
n = number of participants with low viral load on Day 7.
M = number of evaluable participants for Day 7.
Low viral load is defined as log10 viral load < 6.
The regression model contains treatment arm, age at baseline (years), sex, baseline BMI, baseline comorbidities, baseline viral load category and days of symptoms at time of enrollment as covariates.
A risk ratio > 1 favors the treatment arm in the numerator of the ratio (= higher proportion of low viral load on Day 7).
Model specification: * = log link and binomial distribution, # = log link and poisson distribution, § = log link and normal distribution.

Table 14.3.5.4 Shift table of viral culture data by treatment arm, overall and time point (mITT Analysis Set)
(Page x of y)

Programming note: Use same layout as table 14.3.5.5. Present time points Baseline, Day 7 and Day 10.

Footnotes:

Base = Baseline, Post = Post-baseline, Change = Post-baseline - baseline.

At each visit, only participants with a value at both baseline and the respective day are included.

Analysis is based on viral culture data.

Table 14.3.5.5 Shift table of qualitative SARS-CoV-2 RT-PCR by treatment arm (mITT Analysis Set)
(Page x of y)

Time point: Day 3

Treatment	Baseline			Not detected		Detected		Missing		Total	
	Result	n	(%)	n	(%)	n	(%)	n	(%)	n	(%)
SOC (N=xx)	Not detected	xx	(xx.x)	xx	(xx.x)	xx	(xx.x)	xx	(xx.x)	xx	(xx.x)
	Detected	xx	(xx.x)	xx	(xx.x)	xx	(xx.x)	xx	(xx.x)	xx	(xx.x)
	Missing	xx	(xx.x)	xx	(xx.x)	xx	(xx.x)	xx	(xx.x)	xx	(xx.x)
	Total	xx	(xx.x)	xx	(xx.x)	xx	(xx.x)	xx	(xx.x)	xx	(xx.x)
ASAQ (N=xx)	Not detected	xx	(xx.x)	xx	(xx.x)	xx	(xx.x)	xx	(xx.x)	xx	(xx.x)
	Detected	xx	(xx.x)	xx	(xx.x)	xx	(xx.x)	xx	(xx.x)	xx	(xx.x)
	Missing	xx	(xx.x)	xx	(xx.x)	xx	(xx.x)	xx	(xx.x)	xx	(xx.x)
	Total	xx	(xx.x)	xx	(xx.x)	xx	(xx.x)	xx	(xx.x)	xx	(xx.x)
PA (N=xx)	Not detected	xx	(xx.x)	xx	(xx.x)	xx	(xx.x)	xx	(xx.x)	xx	(xx.x)
	Detected	xx	(xx.x)	xx	(xx.x)	xx	(xx.x)	xx	(xx.x)	xx	(xx.x)
	Missing	xx	(xx.x)	xx	(xx.x)	xx	(xx.x)	xx	(xx.x)	xx	(xx.x)
	Total	xx	(xx.x)	xx	(xx.x)	xx	(xx.x)	xx	(xx.x)	xx	(xx.x)
FPV-NTZ (N=xx)	Not detected	xx	(xx.x)	xx	(xx.x)	xx	(xx.x)	xx	(xx.x)	xx	(xx.x)
	Detected	xx	(xx.x)	xx	(xx.x)	xx	(xx.x)	xx	(xx.x)	xx	(xx.x)
	Missing	xx	(xx.x)	xx	(xx.x)	xx	(xx.x)	xx	(xx.x)	xx	(xx.x)
	Total	xx	(xx.x)	xx	(xx.x)	xx	(xx.x)	xx	(xx.x)	xx	(xx.x)
SOF/DCV (N=xx)	Not detected	xx	(xx.x)	xx	(xx.x)	xx	(xx.x)	xx	(xx.x)	xx	(xx.x)
	Detected	xx	(xx.x)	xx	(xx.x)	xx	(xx.x)	xx	(xx.x)	xx	(xx.x)
	Missing	xx	(xx.x)	xx	(xx.x)	xx	(xx.x)	xx	(xx.x)	xx	(xx.x)
	Total	xx	(xx.x)	xx	(xx.x)	xx	(xx.x)	xx	(xx.x)	xx	(xx.x)

Percentage is based on baseline total N for each treatment arm.

Programming note: Present the table for time points Day 3, 10, 14, 21 and 28.

Table 14.3.5.6 Summary of log10 mean viral load of SARS-CoV-2 by treatment arm and overall, time point (mITT Analysis Set)

Base = Baseline, Post = Post-baseline, Change = Post-baseline - baseline.
At each visit, only participants with a value at both baseline and the respective day are included.
Analysis is based on RT-PCR.

Mean viral load values (copies/mL) are calculated across the S-gene, N-gene and ORF1ab gene. Mean viral load values are then log10 transformed.

Programming note: Use layout similar to table 14.2.3.1.

Table 14.3.5.7 Repeated measure analysis of log10 mean viral load of SARS-CoV-2 change from baseline (mITT Analysis Set)
(Page x of y)

Programming note: Use layout similar to table 14.2.3.2.

Table 14.3.5.8 Incidence of SARS-CoV-2 clearance on Day 10 based on viral culture (mITT Analysis Set)

Treatment arm	N	n/M (%)	Comparison*	Risk ratio	95% CI	p-value
SOC	xx	xx/xx (xx.x)				
ASAQ	xx	xx/xx (xx.x)	ASAQ / SOC	xx.x	(xx.x, xx.x)	x.xxx
PA	xx	xx/xx (xx.x)	PA / SOC	xx.x	(xx.x, xx.x)	x.xxx
FPV-NTZ	xx	xx/xx (xx.x)	FPV-NTZ / SOC	xx.x	(xx.x, xx.x)	x.xxx
SOF/DCV	xx	xx/xx (xx.x)	SOF/DCV / SOC	xx.x	(xx.x, xx.x)	x.xxx

N = number of participants in the respective treatment arm.
n = number of participants with SARS-CoV-2 clearance on Day 10 based on viral culture.
M = number of evaluable participants for Day 10.
The regression model contains treatment arm, age at baseline (years), sex, baseline BMI, baseline comorbidities, baseline viral load category and days of symptoms at time of enrollment as covariates.
A risk ratio > 1 favors the treatment arm in the numerator of the ratio (= higher proportion of SARS-CoV-2 clearance).
Model specification: * = log link and binomial distribution, # = log link and poisson distribution, § = log link and normal distribution.

Figure 14.3.5.1 Scatter plot of plasma or blood concentrations at Day 3 and/or Day 7 by SARS-CoV-2 clearance on Day 7 and exploratory arm (PK Analysis Set)
(Page x of y)

Programming note: Present plasma or blood concentrations on y-axis. Separate x-axis by "SARS-CoV-2 D7 clearance" and "No SARS-CoV-2 D7 clearance". Create a separate scatter plot panel for each experimental arm. Use different symbols for different PK components. For ASAQ and PA display data for Cday3 and Cday7 either next to each other on the x-axis or use different symbols. For other experimental arms only display Cday7.

Footnotes:

Patients with missing Cday3 and/or Cday7 concentrations are excluded.

PK analysis is only done for experimental arms, hence participants from SOC arm are not included.

No visit windowing approach was applied.

Concentrations below the lower limit of quantification (LLOQ) were imputed with LLOQ/2. LLOQ(Amodiaquine)=0.156 ng/mL, LLOQ(Artesunate, N-Desethyl Amodiaquine)=1.56 ng/mL, LLOQ(Daclatasvir)= 15.6 ng/mL, LLOQ(Dihydroartemisinin)= 3.91 ng/mL, LLOQ(Favipiravir)= 391 ng/mL, LLOQ(Nitazoxanide, Tizoxanide)= 100 ng/mL, LLOQ(Pyronaridine)=0.977 ng/mL, LLOQ(Sofosbuvir)=2.5 ng/mL.

Listing 14.3.1.1 Listing of adverse events leading to death (Safety Analysis Set)
Programming note: Use shell of Appendix 16.2.7.1

Listing 14.3.1.2 Listing of serious treatment-emergent adverse events (Safety Analysis Set)
Programming note: Use shell of Appendix 16.2.1.7

Listing 14.3.1.3 Listing of treatment-emergent adverse events leading to study discontinuation (Safety Analysis Set)
Programming note: Use shell of Appendix 16.2.7.1

Listing 14.3.1.4 Listing of treatment-emergent adverse events with action taken related to study treatment (Safety Analysis Set)
Programming note: Action taken related to study treatment is either "Dose reduced", "Drug interrupted" or "Drug withdrawn". Use shell of Appendix 16.2.7.1

Appendix 16.2.1.1 Study completion (Randomized Analysis Set)
(Page x of y)

Treatment arm: SOC

Participant ID	Age/ Sex/ Race	Participant status	Study termination date	Primary reason for discontinuation	Any hospitalizations during study period due to COVID-19?*
xxxxx	xx/F/Bl	Completed study	ddMMMyyyy / x		No
xxxxx	xx/M/Bl	Randomized: Discontinued prior to study end	ddMMMyyyy / x	xxxxxxxxxxxxxxxxxxxx	Yes

Day 1 defined as the day of first study drug administration.
* Randomized participants only.
|M=Male, F=Female, As=Asian or Indian, Bl=Black African, Wh=White, Ot=Other.

Programming note: Sort within treatment arm by participant ID. Start a new page for each treatment arm. Derive hospitalization column by checking if participant has record in HO with HOOCCUR="Y".If yes, then populate with Yes, else with No.

Appendix 16.2.1.2 Screening failures (All screened subjects)
(Page x of y)

Participant ID	Age/ Sex/ Race	Study termination date	Primary reason for discontinuation
xxxxx	xx/F/Bl	ddMMMyyyy	
xxxxx	xx/M/Bl	ddMMMyyyy	xxxxxxxxxxxxxxxxxxxx

Only screening failures are displayed.
|M=Male, F=Female, As=Asian or Indian, Bl=Black African, Wh=White, Ot=Other.

Programming note: Sort by participant ID. Display all participants with the DS.DSTERM as primary reason for discontinuation where DS.DSSTATUS='NOT RANDOMIZED: SCREEN FAILURE'.

Appendix 16.2.2.1 Protocol deviation (Randomized Analysis Set)
(Page x of y)

Treatment arm: SOC

Participant ID	Age/ Sex/ Race	Date occurred/day	Date reported/day	Protocol deviation category	Protocol deviation description
xxxxx	xx/F/Bl	ddMMMyyyy / xx	ddMMMyyyy / xx	xxxxxx	xxxxxxx
xxxxx	xx/M/Bl				
Etc...					

Day 1 defined as the day of first study drug administration.
|M=Male, F=Female, As=Asian or Indian, Bl=Black African, Wh=White, Ot=Other.

Programming note: Sort within treatment arm by participant ID. Start a new page for each treatment arm.

Appendix 16.2.3.1 Analysis Sets (Randomized Analysis Set)
(Page x of y)

Treatment arm: SOC

Participant ID	Age/ Sex/ Race	Randomized Analysis Set	Safety Analysis Set	Modified Intention to Treat Analysis Set	As Treated Analysis Set	Pharmacokinetic Analysis Set
xxxxx	xx/F/Bl	Yes	Yes	Yes	No	No
xxxxx	xx/M/Bl	Yes	Yes	Yes	Yes	Yes

Etc...

Day 1 defined as the day of first study drug administration.
|M=Male, F=Female, As=Asian or Indian, Bl=Black African, Wh=White, Ot=Other.

Programming note: Sort within treatment arm by participant ID. Start a new page for each treatment arm.

Appendix 16.2.4.1 Demographic data and informed consent (Randomized Analysis Set)
(Page x of y)

Treatment arm: SOC

Participant ID	Age/ Sex/ Race	Language of FLU-PRO	Date/time of pre-screening informed consent	Date/time of informed consent	Date/time of enrollment informed consent	Height (cm)	Body weight (kg)	BMI (kg/m**2)	Race: Other, specify	Days of symptoms at time of enrollment
xxxx	xx/ F/ Black		ddMMMyyyy/ hh:mm	ddMMMyyyy /hh:mm	ddMMMyyyy/ hh:mm	xxx	xx.x	xx.x		
	xx/ M/ Black		ddMMMyyyy/ hh:mm	ddMMMyyyy /hh:mm	ddMMMyyyy/ hh:mm	xxx	xx.x	xx.x		

BMI = Weight (kg)/[Height (m)]**2.
|M=Male, F=Female, As=Asian or Indian, Bl=Black African, Wh=White, Ot=Other.

Programming note: Sort within treatment arm by participant ID. Start a new page for each treatment arm.

Appendix 16.2.4.2 Medical and surgical history (Randomized Analysis Set)
(Page x of y)

Treatment arm: SOC

Participant ID	Age/ Sex/ Race	Pre-specified term	Medical / surgical history as reported	Preferred term<br>Primary system organ class	Start date/day	End date/day
xxxx	xx/ F/ Black	No	xxx	Xxxxxxx<br>xxxxxx	ddMMMyyyy/xx	ddMMMyyyy/xx
	xx/ M/ Black	Yes		Xxxxxxx<br>xxxxxx	ddMMMyyyy/xx	Ongoing

Day 1 defined as the day of first study drug administration.

M=Male, F=Female, As=Asian or Indian, Bl=Black African, Wh=White, Ot=Other.

Programming note: Sort within treatment arm by participant ID. Start a new page for each treatment arm. If medical history was ticked as 'Ongoing at study end' then 'Ongoing' should be displayed instead of End date/day.

Appendix 16.2.4.3 Physical examination (Randomized Analysis Set)
(Page x of y)

Treatment arm: SOC

Participant ID	Age/ Sex/ Race	Collection date/day/ time/hours since 1st dose	Body system or part	Exam finding	If abnormal: specify
xxxx	xx/ F/ Black	ddMMMyyyy/xx/hh:mm/xx.x	General appearance		
		ddMMMyyyy/xx/hh:mm/xx.x	Skin		
xxxx	xx/ M/ White				

Day 1 defined as the day of first study drug administration.
|M=Male, F=Female, As=Asian or Indian, Bl=Black African, Wh=White, Ot=Other.

Programming note: Sort within treatment arm by participant ID, collection date/time and body system or part. Start a new page for each treatment arm.

Appendix 16.2.4.4 Randomization (Randomized Analysis Set)
(Page x of y)

Participant ID	Age/Sex/Race	Randomization date/time	Randomization number	Randomized treatment arm	Actual treatment arm	Re-screened?	Randomized manually?
xxxx	xx/F/Black	ddMMMyyyy/hh:mm	xxx	SOC	SOC		
xxxx	xx/M/White						

|M=Male, F=Female, As=Asian or Indian, Bl=Black African, Wh=White, Ot=Other.

Programming note: Sort within randomized treatment arm and participant ID. Start a new page for each treatment arm. Display re-screened column using SUPPDS.QNAM="DSRESCRN" and randomized manually column using SUPPDS.QNAM="MANYN".

Appendix 16.2.5.1 Drug accountability (Safety Analysis Set)
(Page x of y)

Treatment arm: SOC

Par. ID	Age/ Sex/ Race	Drug	Type	Date/ day	No. of tablets	Compliance*
xxxx	xx/F/ Bl	Paracetamol	Dispense	ddMMMyyyy/ xx	xx	
		xxx	Return	ddMMMyyyy/ xx	xx	
etc.						

*Compliance is only calculated for experimental treatments.
Day 1 defined as the day of first study drug administration.
|M=Male, F=Female, As=Asian or Indian, Bl=Black African, Wh=White, Ot=Other.

Programming note: Sort within treatment arm by participant ID, drug, type (first dispense, then return), date. Display compliance only for experimental treatments. If there are multiple rows or dispense/return, show compliance in last row.
Start a new page for each treatment arm.

Appendix 16.2.5.2 Pharmacokinetic concentration data (PK Analysis Set)
(Page x of y)

Treatment arm: ASAQ

Par. ID	Age/ Sex/ Race	PK parameter (unit)	Sample date/day/time	Concentration	Date/day/time of IMP dose administ. prior to blood sampling	Samples stored and processed appropriately
xxxx	xx/F/Bl	xxx (xx)	ddMMMyyyy/xx/hh:mm<br>ddMMMyyyy/xx/hh:mm	xxx	ddMMMyyyy/xx/hh:mm	Yes

etc.

Day 1 defined as the day of first study drug administration.
|M=Male, F=Female, As=Asian or Indian, Bl=Black African, Wh=White, Ot=Other.

Programming note: Sort within treatment arm by participant ID, PK parameter and date/time. Display only experimental arms as no PK parameter. Present only available data.
Start a new page for each treatment arm.

Appendix 16.2.6.1 Mid-nasal swab and saliva specimen data, viral culture and SARS-CoV-2 serology (Randomized Analysis Set)
(Page x of y)

Treatment arm: SOC

Participant ID	Age/ Sex/ Race	Visit	Category	Specimen material type	Collection date/day/ time/hours since 1st dose	Parameter	Result	Unit
xxxx	xx/ F/ Black	Day 2	SINGLE PLEX RT-PCR	NASOPHARYNGEAL SWAB	ddMMMyyyy/xx/hh:mm/xx.x		Positive	
					ddMMMyyyy/xx/hh:mm/xx.x			
xxxx	xx/ M/ White							

Day 1 defined as the day of first study drug administration.
|M=Male, F=Female, As=Asian or Indian, Bl=Black African, Wh=White, Ot=Other.

Programming note: Sort within treatment arm by participant ID, collection date/time, category, specimen and parameter. Start a new page for each treatment arm. Present data from MB domain. Present unit if available in separate column. For category column use MB.MBCAT and for specimen material type use MB.MBSPEC.

Appendix 16.2.6.2 Hospitalizations (Randomized Analysis Set)
(Page x of y)

Treatment arm: SOC

Participant ID	Age/ Sex/ Race	Date of admission/day	Date of discharge/day	Maximum WHO Ordinal Scale for Clinical Improvement during Admission period
xxxx	xx/ F/ Black	ddMMMyyyy/xx	ddMMMyyyy/xx	xx
		ddMMMyyyy/xx	ddMMMyyyy/xx	
xxxx	xx/ M/ White			

Day 1 defined as the day of first study drug administration.
|M=Male, F=Female, As=Asian or Indian, Bl=Black African, Wh=White, Ot=Other.

Programming note: Sort within treatment arm by participant ID and date of admission. Start a new page for each treatment arm.

Appendix 16.2.6.3 FLU-PRO Plus (Randomized Analysis Set)
(Page x of y)

Treatment arm: SOC

Part. ID	Age/ Sex/ Race	Date/day/ time/hours since 1st dose	Visit	Question	Item	Answer
xxxx	xx/F/Bl	ddMMMyyyy/xx/ hh:mm/xx.x	Baseline	Please rate the extent to which you had each symptom during the past 24 hours	Runny or dripping nose	Somewhat
					xxx	
					xxx	
					...	
				In the past 24 hours, how often have you had any of the following symptoms?	Sneezing	Never
					xxx	
Etc...						

Day 1 defined as the day of first study drug administration.
|M=Male, F=Female, As=Asian or Indian, Bl=Black African, Wh=White, Ot=Other.

Programming note: Sort within treatment arm by participant ID and date/time, and question. Start a new page for each treatment arm.

Appendix 16.2.6.4 FLU-PRO Plus Global additional daily diary items (Randomized Analysis Set)
(Page x of y)

Treatment arm: SOC

Part. ID	Age/ Sex/ Race	Date/day/	Visit	Question	Answer
xxxx	xx/F/Bl	ddMMMyyyy/xx		Overall, how severe were your infection symptoms today?	Mild
				xxx	
Etc...					

* Time point displays the mapped time window for analysis.
Day 1 defined as the day of first study drug administration.
|M=Male, F=Female, As=Asian or Indian, Bl=Black African, Wh=White, Ot=Other.

Programming note: Sort within treatment arm by participant ID and date/time, and question. Present all FLU-PRO Plus global additional daily diary items and also the following questions from general daily diary items: 'Have you taken your daily medication since survey', 'Have you taken any other medication?' (Show also SUPPFA.FADESC if answer is yes as "Yes, FADESC") and 'Have you observed any change or new complaint since you last completed the survey?' (Show also SUPPFA.FADESC if answer is yes as "Yes, FADESC").
Start a new page for each treatment arm.

Appendix 16.2.6.5 WHO Ordinal Scale for Clinical Improvement (Randomized Analysis Set)
(Page x of y)

Treatment arm: SOC

Participant ID	Age/Sex/Race	Date/day/ time/hours since 1st dose	Visit	Score
xxxx	xx/F/Bl	ddMMMyyyy/xx/ hh:mm/xx.x	Baseline	2
		ddMMMyyyy/xx/ hh:mm/xx.x	Day 7	1

Etc...

Day 1 defined as the day of first study drug administration.
|M=Male, F=Female, As=Asian or Indian, Bl=Black African, Wh=White, Ot=Other.

Programming note: Sort within treatment arm by participant ID and date/time. Start a new page for each treatment arm.

Appendix 16.2.7.1 Treatment emergent adverse events (Safety Analysis Set)
(Page x of y)

Treatment arm: SOC

Par. ID	Age/ Sex/ Race	Adverse event INVESTIGATOR TERM/ Preferred term/SOC	Start date/ day	Outcome date/day	Severity*	Related To study Treatment?	Action Taken**	Outcome	SAE	If SAE: Serious due to?
xxxx	xx/F/ Bl	XXXXXXXXXXXX/ xxxxxxxxxxx/xxxx	ddMMMyyyy/ xx	ddMMMyyy/ xx	Grade 1	Not related	1	Unknown	Yes	Life-threatening

etc.

Day 1 defined as the day of first study drug administration.
* The severity of AEs is assessed according to the DAIDS AE grading Table, corrected Version 2.1, July 2017.
** Action taken: 1 = Not applicable, 2 = Unknown , 3 = No change, 4 = Dose reduced, 5 = Drug interrupted, 6 = Drug withdrawn. If action taken = Drug withdrawn then the day of drug withdrawal is given in brackets.
MedDRA version xx.x has been used for coding.
|M=Male, F=Female, As=Asian or Indian, Bl=Black African, Wh=White, Ot=Other.

Programming note: Sort within treatment arm by participant ID, AE start date. If action taken = Drug withdrawn, display corresponding numerical code and in in brackets the day on which the drug was withdrawn (AEENDY).
Start a new page for each treatment arm.

Appendix 16.2.7.2 Vital signs (Safety Analysis Set)
(Page x of y)

Treatment arm: SOC

Participant ID	Age/ Sex/ Race	Date/day/ time/hours since 1st dose	Visit	Pulse (bpm)	Temperature (degree C)	Respiratory rate (breaths per minute)	SpO2 (%)	SpO2 <93%?
xxxx	xx/ F/ Black	ddMMMyyyy/xx /hh:mm/xx.x	Baseline	xx	xx.x	xx	xx	No
		ddMMMyyyy/xx /hh:mm/xx.x	Day 2					
xxxx	xx/ M/ White							

Day 1 defined as the day of first study drug administration.

M=Male, F=Female, As=Asian or Indian, Bl=Black African, Wh=White, Ot=Other.

Programming note: Sort within treatment arm by participant ID, parameter and collection date/time. Display all vital sign parameters. Start a new page for each treatment arm.

Appendix 16.2.7.3 Prior and concomitant medications (Safety Analysis Set)
(Page x of y)

Treatment arm: SOC

Part. ID	Age/ Sex/ Race	Treatment INVESTIGATOR TERM Preferred term/ATC class	Date/day started	Date/day stopped	Dose (unit)	Frequency	Route	Type
xxxx	xx/F/Bl	Xxxxxxxxxxxxxxxx xxxxxxxxxxxxxx/xxxxxxxx	ddMMMyyy /xx	ddMMMyyyy /xx	xxx	xxx	xxx	Prior
		Xxxxxxxxxxxxxxxx xxxxxxxxxxxxxx/xxxxxxxx	ddMMMyyy /xx	ddMMMyyyy /xx	xxx	xxx	xxx	Concomitant
xxxx	xx/F/Bl	Xxxxxxxxxxxxxxxx xxxxxxxxxxxxxx/xxxxxxxx	ddMMMyyyy /xx	ddMMMyyyy /xx	xxx	xxx	xxx	Prior

Etc...

Day 1 defined as the day of first study drug administration.
Medications were coded using the World Health Organizational-Drug Dictionary (WHO-DD) version xx.
|M=Male, F=Female, As=Asian or Indian, Bl=Black African, Wh=White, Ot=Other.

Programming note: Sort within treatment arm by participant ID and start date. Start a new page for each treatment arm. For Frequency and Route: if the 'Other, specify' item is used, use that for presentation.

17. Appendices

17.1 FLU-PRO total and domain scoring

The derivation of FLU-PRO Total and domain scores (including rules for handling missing data) according to the FLU-PRO user manual version 1.2 are provided in the table 2 below.

Table 2

Domain	Items	Scoring	Minimum Data
**Nose**	Runny or dripping nose<br>Congested or stuffy nose Sneezing<br>Sinus pressure	Arithmetic mean of 4 items within **Nose** domain	Daily score for 3 of 4 items must be present to calculate domain score
**Throat**	Scratchy or itchy throat<br>Sore or painful throat<br>Difficulty swallowing	Arithmetic mean of 3 items within **Throat** domain	Daily score for 2 of 3 items must be present to calculate domain score
**Eyes**	Teary or watery eyes<br>Sore or painful eyes<br>Eyes sensitive to light	Arithmetic mean of 3 items within **Eyes** domain	Daily score for 2 of 3 items must be present to calculate domain score
**Chest/Respiratory**	Trouble breathing<br>Chest congestion<br>Chest tightness<br>Dry or hacking cough<br>Wet or loose cough<br>Coughing<br>Coughed up mucus or phlegm	Arithmetic mean of 7 items within **Chest/Respiratory** domain	Daily score for 5 of 7 items must be present to calculate domain score

**Gastrointestinal**	Felt nauseous<br>Stomachache<br>How many times did you vomit? How many times did you have diarrhea?	Arithmetic mean of 4 items within **Gastrointestinal** domain	Daily score for 3 of 4 items must be present to calculate domain score
**Body/Systemic**	Headache<br>Head congestion<br>Felt dizzy<br>Lack of appetite<br>Sleeping more than usual<br>Body aches or pains<br>Weak or tired<br>Chills or shivering<br>Felt cold<br>Felt hot	Arithmetic mean of 11 items within **Body/Systemic** domain	Daily score for 8 of 11 items must be present to calculate domain score
**Total**	All above 32 items	Arithmetic mean of all 32 items within FLU-PRO	In the presence of missing data, the above conditions for the calculation of all domain scores must be met in order to calculate the FLU-PRO total score.
