# Supplementary material for: Safety and efficacy of four drug regimens versus standard-of-care for the treatment of symptomatic outpatients with COVID-19: A randomised, open-label, multi-arm, phase 2 clinical trial
Source: eBioMedicine. 2022 Nov 1;86:104322. doi: 10.1016/j.ebiom.2022.104322 (PMC9624152; doi:10.1016/j.ebiom.2022.104322)
Supplement: Supplementary Data [file mmc4.pdf]

## Supplementary data

# Safety and efficacy of four drug regimens versus standard-of-care for the treatment of symptomatic outpatients with COVID-19: a randomised, open-label, multi-arm, phase 2 clinical trial

Nomathemba Chandiwana, M.D.; Chelsea Kruger, M.D.; Hilary Johnstone, M.D.; M. Farouk Chughlay, M.D.; Chung Ju, Ph.D.; Byungsu Kim, M.Sc.; Yengiwe Dineka, B.Nurs.; Sarah Arbe-Barnes, Ph.D.; Robert Miller, M.D.; Andrew Owen, Ph.D.; Andrew Hill, M.D.; Daniel Windgassen, M.Sc.; Nada Abla, Ph.D.; Anne-Claire Marrast, M.D.; Stephan Duparc, M.D.; W.D. Francois Venter, FCP

## Contents

Table S1 Post-screening schedule of patient assessments. .... 3

Table S2 Bioanalytical methods to determine drug concentrations. .... 4

Table S3 Sensitivity analysis of the primary endpoint: incidence of SARS-CoV-2 clearance on day 7 based on qualitative RT-PCR. .... 5

Table S4 Primary analysis of the incidence of SARS-CoV-2 clearance on day 7 based on RT-PCR by subgroup (mITT population). See also Figure S1. .... 6

Table S5 Incidence of SARS-CoV-2 clearance on day 7 based on viral culture (mITT population). .... 10

Table S6 Incidence of SARS-CoV-2 clearance on days 3, 10, 14, 21, and 28 based on RT-PCR (mITT population). See also Figure S1. .... 11

Figure S1 Incidence of SARS-CoV-2 clearance on days 3, 10, 14, 21, and 28 based on RT-PCR (mITT population). See also Table S6. .... 12

Table S7 Repeated measure analysis of $log_{10}$ viral load of SARS-CoV-2 change from baseline (mITT population) .... 13

Table S8 Repeated measure analysis of $log_{10}$ viral load of SARS-CoV-2 change from baseline in high-risk patients (mITT population). .... 14

Table S9 Proportional odds model for disease progression for day 7, 14, 21, and 28 (mITT population) .... 15

Table S10 Cox regression analysis of time to first zero WHO Ordinal Scale score for Clinical Improvement (mITT population). .... 16

Table S11 Poisson regression analysis for the proportion of days with fever, $SpO_2$ values <93%, or respiratory symptoms after randomisation (mITT population). .... 17

Figure S2 FLU-PRO Plus questionnaire scores and changes from baseline (mITT population). .... 18

Table S12 Investigational drug blood or plasma concentrations (pharmacokinetic population) .... 19

Figure S3 Drug plasma or blood concentrations at day 3 and day 7 in patients with or without SARS-CoV-2 clearance based on RT-PCR at day 7 (pharmacokinetic population). .... 20

Table S13 Treatment emergent adverse events of any cause and maximum severity (safety population) .... 22

Table S13 Treatment emergent adverse events considered to be study drug related (safety population)........................................................................................................................ 28
Figure S4 Change in vital signs from baseline (safety population). ..................................................... 29
Figure S5 Shift from baseline to day 28 in serology (mITT population)............................................... 30

**Table S1 Post-screening schedule of patient assessments.**

| Procedure | Enrolment Day 1[a] | Treatment and follow-up period — days | | | | | | | | | | EOS Day 28 (±2) |
|---|---|---|---|---|---|---|---|---|---|---|---|---|
| | | 2 | 3 (+1) | 4–6 | 7 (±1) | 8–9 | 10 (±1) | 11–13 | 14 (±2) | 15–20 | 21 (±2) | |
| Study therapy[b] | ● | ● | ● | ● | ● | | | | | | | |
| Pharmacokinetic sampling[b] | | | ● | | ● | | | | | | | |
| Mid-nasal swab and saliva specimen | | | ● | | ● | | ● | | ● | | ● | ● |
| Blood for serology | ● | | | | | | | | | | | ● |
| Participant reported vital signs | ● | ● | ● | ● | ● | ● | ● | ● | ● | | ● | ● |
| Participant reported $SpO_2$ | ● | ● | ● | ● | ● | ● | ● | ● | ● | | | |
| Completion of daily survey | ● | ● | ● | ● | ● | ● | ● | ● | ● | | ● | ● |
| Completion of FLU-PRO Plus questionnaire | ● | ● | ● | ● | ● | ● | ● | ● | ● | | ● | ● |
| WHO Ordinal Scale for Clinical Improvement[c] | ● | | | | ● | | | | ● | | ● | ● |
| Adverse event review and clinician contact | ● | ● | ● | ● | ● | ● | ● | ● | ● | ● | ● | ● |

EOS, end of study; WHO, World Health Organization.

[a] Additional assessments that were conducted on day 1 and not during follow-up were: confirmation of inclusion and exclusion criteria, completion of the consent process, assessment of past and current medical conditions and concomitant medications, physical examination, vital signs, and an electrocardiograph.

[b] The duration of treatment and pharmacokinetic sampling were dependent upon the treatment arm.

[c] By 9-point Scale; 0-uninfected, 1-no limitation of activities, 2-limitation of activities, 3-hospitalized but no oxygen therapy, 4-oxygen by mask or nasal prongs, 5-non-invasive ventilation or high-flow oxygen, 6-intubation and mechanical ventilation, 7-ventilation with additional organ support, 8-death.

**Table S2 Bioanalytical methods to determine drug concentrations.**

| Analyte | Calibration range | Matrix | Internal standard |
|---|---|---|---|
| Amodiaquine | 0·156–10·0 ng/mL | Plasma | Amodiaquine-d10 |
| N-desethyl amodiaquine | 1·56–100 ng/mL | Plasma | N-desethyl amodiaquine-d5 |
| Favipiravir | 0·391–25·0 µg/mL | Plasma | Favipiravir Impurity 3 |
| Sofosbuvir | 2·50–160 ng/mL | Plasma | [$2H_6$]-Sofosbuvir |
| Artesunate | 1·56–200 ng/mL | Plasma | Artesunate-d4 |
| Dihydroartemisinin | 3·91–500 ng/mL | Plasma | Dihydroartemisinin-d4 |
| Daclatasavir | 15·6–1000 ng/mL | Plasma | Daclatasavir-d6 |
| Nitazoxanide | 100–15,000 ng/mL | Plasma | Nitazoxanide |
| Pyronaridine | 0·977–500 ng/mL | Whole blood | Pyronaridine $^{13}C_2$-d4 |
| Tizoxanide | 100–15,000 ng/mL | Plasma | Tizoxanide-d4 |

Extraction from the biological matrix used a solid phase extraction technique, with analysis by liquid chromatography with tandem mass spectrometry detection (LC-MS-MS).

Target limits for evaluable calibration standards were ±15% bias of each calibration standard point (±20% at the lower limit of quantification) and ±15% bias for each quality control sample.

**Table S3 Sensitivity analysis of the primary endpoint: incidence of SARS-CoV-2 clearance on day 7 based on qualitative RT-PCR.**

| **mITT population missing = failure** | **SOC (n=39)** | **ASAQ (n=39)** | **PA (n=36)** | **FPV+NTZ (n=37)** | **SOF-DCV (n=35)** |
|---|---|---|---|---|---|
| Covariate adjusted analysis[a] | | | | | |
| Incidence, n/N (%)[b] | 13/39 (33·3) | 15/39 (38·5) | 10/35 (28·6) | 10/37 (27·0) | 8/35 (22·9) |
| Risk ratio (95% CI) | Reference | 0·96 (0·55, 1·67) | 0·70 (0·36, 1·34) | 0·72 (0·38, 1·34) | 0·55 (0·27, 1·12) |
| *P* value | Reference | 0·87 | 0·28 | 0·30 | 0·099 |
| Crude analysis | | | | | |
| Incidence, n/N (%)[a] | 13/39 (33·3) | 15/39 (38·5) | 10/36 (27·8) | 10/37 (27·0) | 8/35 (22·9) |
| Risk ratio (95% CI) | Reference | 1·15 (0·64, 2·09) | 0·86 (0·43, 1·7) | 0·81 (0·41, 1·62) | 0·69 (0·32, 1·46) |
| *P* value | Reference | 0·64 | 0·66 | 0·55 | 0·33 |
| **As-treated population** | **SOC (n=38)** | **ASAQ (n=38)** | **PA (n=34)** | **FPV+NTZ (n=35)** | **SOF-DCV (n=33)** |
| Covariate adjusted analysis[a] | | | | | |
| Incidence, n/N (%)[b] | 13/38 (34·2) | 15/38 (39·5) | 11/33 (33·3) | 9/35 (25·7) | 8/33 (24·2) |
| Risk ratio (95% CI) | Reference | 0·87 (0·46, 1·63) | 0·72 (0·40, 1·03) | 0·55 (0·27, 1·11) | 0·47 (0·22, 1·01) |
| *P* value | Reference | 0·66 | 0·66 | 0·093 | 0·054 |
| Crude analysis | | | | | |
| Incidence, n/N (%)[a] | 13/38 (34·2) | 15/38 (39·5) | 11/34 (32·4) | 9/35 (25·7) | 8/33 (24·2) |
| Risk ratio (95% CI) | Reference | 1·15 (0·64, 2·08) | 0·97 (0·51, 1·87) | 0·75 (0·37, 1·54) | 0·71 (0·34, 1·5) |
| *P* value | Reference | 0·64 | 0·94 | 0·43 | 0·37 |

[a] The covariate adjusted regression model contained treatment arm, age at baseline (years), sex, baseline BMI, baseline comorbidities, baseline viral load category and days of symptoms at time of enrolment as covariates. The crude analysis repeated the regression model without any covariate adjustment.

[b] n/N is number of patients with clearance / number of patients evaluable at day 7.

mITT, modified intention-to-treat; SOC, standard-of-care; ASAQ, artesunate-amodiaquine; PA, pyronaridine-artesunate; FPV+NTZ, favipiravir plus nitazoxanide; SOF-DCV, sofosbuvir-daclatasvir.

**Table S4 Primary analysis of the incidence of SARS-CoV-2 clearance on day 7 based on RT-PCR by subgroup (mITT population). See also Figure S1.**

| Subgroup | Category | Treatment arm | N | n/N (%)[a] | Comparison | Risk ratio (95%CI) | *P* value |
|---|---|---|---|---|---|---|---|
| Age | Low (≤33 years) | SOC | 22 | 8/21 (38·1) | | | |
| | | ASAQ | 19 | 5/19 (26·3) | ASAQ / SOC | 0·754 (0·245, 2·324) | 0·6235 |
| | | PA | 18 | 5/18 (27·8) | PA / SOC | 0·739 (0·230, 2·382) | 0·6131 |
| | | FPV+NTZ | 19 | 7/19 (36·8) | FPV+NTZ / SOC | 0·953 (0·327, 2·780) | 0·9302 |
| | | SOF-DCV | 18 | 3/18 (16·7) | SOF-DCV / SOC | 0·387 (0·100, 1·504) | 0·1706 |
| | High (>33 years) | SOC | 17 | 5/17 (29·4) | | | |
| | | ASAQ | 20 | 10/20 (50·0) | ASAQ / SOC | 1·362 (0·458, 4·049) | 0·5779 |
| | | PA | 18 | 5/15 (33·3) | PA / SOC | 0·760 (0·216, 2·674) | 0·6686 |
| | | FPV+NTZ | 18 | 3/18 (16·7) | FPV+NTZ / SOC | 0·419 (0·097, 1·811) | 0·2444 |
| | | SOF-DCV | 17 | 5/16 (31·3) | SOF-DCV / SOC | 0·885 (0·245, 3·202) | 0·8526 |
| Sex | Male | SOC | 15 | 5/14 (35·7) | | | |
| | | ASAQ | 14 | 5/14 (35·7) | ASAQ / SOC | 0·661 (0·186, 2·344) | 0·5218 |
| | | PA | 16 | 7/14 (50·0) | PA / SOC | 0·804 (0·244, 2·649) | 0·7201 |
| | | FPV+NTZ | 22 | 5/22 (22·7) | FPV+NTZ / SOC | 0·374 (0·102, 1·365) | 0·1365 |
| | | SOF-DCV | 20 | 4/20 (20·0) | SOF-DCV / SOC | 0·300 (0·078, 1·160) | 0·0811 |
| | Female | SOC | 24 | 8/24 (33·3) | | | |
| | | ASAQ | 25 | 10/25 (40·0) | ASAQ / SOC | 1·283 (0·501, 3·289) | 0·6035 |
| | | PA | 20 | 3/19 (15·8) | PA / SOC | 0·421 (0·108, 1·643) | 0·2132 |
| | | FPV+NTZ | 15 | 5/15 (33·3) | FPV+NTZ / SOC | 1·057 (0·342, 3·272) | 0·9228 |
| | | SOF-DCV | 15 | 4/14 (28·6) | SOF-DCV / SOC | 1·035 (0·296, 3·615) | 0·9567 |
| BMI | Low (≤30 kg/m$^2$) | SOC | 28 | 10/27 (37·0) | | | |
| | | ASAQ | 24 | 10/24 (41·7) | ASAQ / SOC | 0·902 (0·372, 2·192) | 0·8205 |
| | | PA | 21 | 7/19 (36·8) | PA / SOC | 0·706 (0·259, 1·926) | 0·4963 |
| | | FPV+NTZ | 24 | 8/24 (33·3) | FPV+NTZ / SOC | 0·645 (0·243, 1·713) | 0·3787 |
| | | SOF-DCV | 20 | 4/19 (21·1) | SOF-DCV / SOC | 0·360 (0·108, 1·203) | 0·0970 |
| | High (>30 kg/m$^2$) | SOC | 11 | 3/11 (27·3) | | | |
| | | ASAQ | 15 | 5/15 (33·3) | ASAQ / SOC | 1·431 (0·339, 6·036) | 0·6258 |
| | | PA | 15 | 3/14 (21·4) | PA / SOC | 0·758 (0·150, 3·835) | 0·7376 |
| | | FPV+NTZ | 13 | 2/13 (15·4) | FPV+NTZ / SOC | 0·742 (0·119, 4·617) | 0·7489 |
| | | SOF-DCV | 15 | 4/15 (26·7) | SOF-DCV / SOC | 1·219 (0·264, 5·639) | 0·7998 |

| Subgroup | Category | Treatment arm | N | n/N (%)[a] | Comparison | Risk ratio (95%CI) | *P* value |
|---|---|---|---|---|---|---|---|
| Comorbidities | None | SOC | 28 | 11/27 (40·7) | | | |
| | | ASAQ | 22 | 9/22 (40·9) | ASAQ / SOC | 0·769 (0·312, 1·893) | 0·5674 |
| | | PA | 21 | 7/19 (36·8) | PA / SOC | 0·704 (0·262, 1·894) | 0·4869 |
| | | FPV+NTZ | 21 | 7/21 (33·3) | FPV+NTZ / SOC | 0·615 (0·229, 1·652) | 0·3347 |
| | | SOF-DCV | 19 | 4/18 (22·2) | SOF-DCV / SOC | 0·371 (0·112, 1·228) | 0·1045 |
| | ≥1 comorbidity | SOC | 11 | 2/11 (18·2) | | | |
| | | ASAQ | 17 | 6/17 (35·3) | ASAQ / SOC | 2·395 (0·478, 11·988) | 0·2879 |
| | | PA | 15 | 3/14 (21·4) | PA / SOC | 0·914 (0·150, 5·560) | 0·9225 |
| | | FPV+NTZ | 16 | 3/16 (18·8) | FPV+NTZ / SOC | 0·991 (0·157, 6·263) | 0·9923 |
| | | SOF-DCV | 16 | 4/16 (25·0) | SOF-DCV / SOC | 1·417 (0·253, 7·945) | 0·6919 |
| Viral load | Low (<176,145 copies/mL) | SOC | 17 | 10/16 (62·5) | | | |
| | | ASAQ | 18 | 11/18 (61·1) | ASAQ / SOC | 0·666 (0·327, 1·355) | 0·2623 |
| | | PA | 20 | 9/19 (47·4) | PA / SOC | 0·666 (0·349, 1·269) | 0·2162 |
| | | FPV+NTZ | 18 | 9/18 (50·0) | FPV+NTZ / SOC | 0·560 (0·268, 1·169) | 0·1226 |
| | | SOF-DCV | 19 | 6/18 (33·3) | SOF-DCV / SOC | 0·379 (0·160, 0·901) | 0·0280 |
| | High (≥175,145 copies/mL) | SOC | 22 | 3/22 (13·6) | | | |
| | | ASAQ | 21 | 4/21 (19·0) | ASAQ / SOC | 1·672 (0·421, 6·632) | 0·4648 |
| | | PA | 15 | 1/14 (7·1) | PA / SOC | 0·558 (0·065, 4·814) | 0·5957 |
| | | FPV+NTZ | 19 | 1/19 (5·3) | FPV+NTZ / SOC | 0·429 (0·048, 3·805) | 0·4475 |
| | | SOF-DCV | 16 | 2/16 (12·5) | SOF-DCV / SOC | 1·005 (0·190, 5·314) | 0·9949 |
| Days of symptoms | Low (≤ 3 days) | SOC | 39 | 13/38 (34·2) | | | |
| | | ASAQ | 37 | 15/37 (40·5) | ASAQ / SOC | 1·071 (0·502, 2·285) | 0·8600 |
| | | PA | 34 | 9/31 (29·0) | PA / SOC | 0·718 (0·300, 1·719) | 0·4568 |
| | | FPV+NTZ | 34 | 9/34 (26·5) | FPV+NTZ / SOC | 0·647 (0·264, 1·582) | 0·3393 |
| | | SOF-DCV | 35 | 8/34 (23·5) | SOF-DCV / SOC | 0·571 (0·225, 1·452) | 0·2395 |
| | High (> days) | SOC | 0 | 0 | | | |
| | | ASAQ | 2 | 0/ 2 (0·0) | ASAQ / SOC | NE (NE, NE) | NE |
| | | PA | 2 | 1/ 2 (50·0) | PA / SOC | NE (NE, NE) | NE |
| | | FPV+NTZ | 3 | 1/ 3 (33·3) | FPV+NTZ / SOC | NE (NE, NE) | NE |
| | | SOF-DCV | 0 | 0 | SOF-DCV / SOC | | |
| Risk[b] | Low risk | SOC | 27 | 10/26 (38·5) | | | |
| | | ASAQ | 21 | 8/21 (38·1) | ASAQ / SOC | 0·750 (0·289, 1·945) | 0·5541 |

| Subgroup | Category | Treatment arm | N | n/N (%)[a] | Comparison | Risk ratio (95%CI) | *P* value |
|---|---|---|---|---|---|---|---|
| | | PA | 20 | 7/18 (38·9) | PA / SOC | 0·733 (0·268, 2·006) | 0·5451 |
| | | FPV+NTZ | 21 | 7/21 (33·3) | FPV+NTZ / SOC | 0·638 (0·234, 1·743) | 0·3810 |
| | | SOF-DCV | 19 | 4/18 (22·2) | SOF-DCV / SOC | 0·381 (0·113, 1·281) | 0·1188 |
| | High risk | SOC | 12 | 3/12 (25·0) | | | |
| | | ASAQ | 18 | 7/18 (38·9) | ASAQ / SOC | 1·971 (0·504, 7·699) | 0·3292 |
| | | PA | 16 | 3/15 (20·0) | PA / SOC | 0·773 (0·149, 4·013) | 0·7593 |
| | | FPV+NTZ | 16 | 3/16 (18·8) | FPV+NTZ / SOC | 0·817 (0·148, 4·496) | 0·8163 |
| | | SOF-DCV | 16 | 4/16 (25·0) | SOF-DCV / SOC | 1·189 (0·251, 5·629) | 0·8271 |

[a] Values are n/N (%) where n/N is number of patients with clearance / number of patients evaluable at day 7.

[b] High risk was defined as age >60 years or body mass index >30 kg/m$^2$ plus the presence of at least one comorbidity for progression to severe disease.

mITT, modified intention-to-treat; SOC, standard-of-care; ASAQ, artesunate-amodiaquine; PA, pyronaridine-artesunate; FPV+NTZ, favipiravir plus nitazoxanide; SOF-DCV, sofosbuvir-daclatasvir; NE, non-evaluable.

**Table S5 Incidence of SARS-CoV-2 clearance on day 7 based on viral culture (mITT population).**

| Treatment arm | N | n/N (%)[a] | Comparison | Risk ratio (95%CI) | *P* value |
|---|---|---|---|---|---|
| SOC | 39 | 6/6 (100) | | | |
| ASAQ | 39 | 4/4 (100) | ASAQ / SOC | 1·021 (0·269, 3·875) | 0·9759 |
| PA | 36 | 3/3 (100) | PA / SOC | 1·068 (0·238, 4·801) | 0·9317 |
| FPV+NTZ | 37 | 10/11 (90.9) | FPV+NTZ / SOC | 0·855 (0·297, 2·459) | 0·7713 |
| SOF-DCV | 35 | 5/6 (83.3) | SOF-DCV / SOC | 0·772 (0·224, 2·663) | 0·6816 |

[a] Number of patients with outcome/number of patients evaluable at day 7.

The regression model contains treatment arm, age at baseline (years), sex, baseline body mass index, baseline comorbidities, baseline viral load category and days of symptoms at time of enrolment as covariates.

mITT, modified intention-to-treat; SOC, standard-of-care; ASAQ, artesunate-amodiaquine; PA, pyronaridine-artesunate; FPV+NTZ, favipiravir plus nitazoxanide; SOF-DCV, sofosbuvir-daclatasvir.

**Table S6 Incidence of SARS-CoV-2 clearance on days 3, 10, 14, 21, and 28 based on RT-PCR (mITT population). See also Figure S1.**

| Day | Treatment arm | N | n/N (%)[a] | Comparison | Risk ratio (95%CI) | *P* value |
|---|---|---|---|---|---|---|
| 3[b] | SOC | 39 | 11/39 (28·2) | | | |
| | ASAQ | 39 | 7/39 (17·9) | ASAQ / SOC | 0·468 (0·172, 1·26) | 0·1354 |
| | PA | 36 | 6/35 (17·1) | PA / SOC | 0·335 (0·114, 0·98) | 0·0462 |
| | FPV+NTZ | 37 | 9/37 (24·3) | FPV+NTZ / SOC | 0·570 (0·218, 1·49) | 0·2517 |
| | SOF-DCV | 35 | 5/35 (14·3) | SOF-DCV / SOC | 0·315 (0·101, 0·98) | 0·0470 |
| 10[c] | SOC | 39 | 13/39 (33·3) | | | |
| | ASAQ | 39 | 13/39 (33·3) | ASAQ / SOC | 0·930 (0·507, 1·70) | 0·8158 |
| | PA | 36 | 14/35 (40·0) | PA / SOC | 0·904 (0·511, 1·59) | 0·7279 |
| | FPV+NTZ | 37 | 11/37 (29·7) | FPV+NTZ / SOC | 0·857 (0·475, 1·54) | 0·6104 |
| | SOF-DCV | 35 | 12/35 (34·3) | SOF-DCV / SOC | 1·001 (0·568, 1·76) | 0·9983 |
| 14[c] | SOC | 39 | 22/39 (56·4) | | | |
| | ASAQ | 39 | 20/39 (51·3) | ASAQ / SOC | 0·979 (0·644, 1·49) | 0·9227 |
| | PA | 36 | 16/35 (45·7) | PA / SOC | 0·792 (0·513, 1·22) | 0·2940 |
| | FPV+NTZ | 37 | 19/37 (51·4) | FPV+NTZ / SOC | 0·954 (0·626, 1·45) | 0·8258 |
| | SOF-DCV | 35 | 18/35 (51·4) | SOF-DCV / SOC | 0·909 (0·580, 1·42) | 0·6760 |
| 21[b] | SOC | 39 | 26/39 (66·7) | | | |
| | ASAQ | 39 | 21/39 (53·8) | ASAQ / SOC | 0·775 (0·434, 1·38) | 0·3888 |
| | PA | 36 | 24/35 (68·6) | PA / SOC | 0·973 (0·548, 1·72) | 0·9259 |
| | FPV+NTZ | 37 | 24/37 (64·9) | FPV+NTZ / SOC | 0·948 (0·533, 1·68) | 0·8549 |
| | SOF-DCV | 35 | 24/35 (68·6) | SOF-DCV / SOC | 0·952 (0·535, 1·69) | 0·8679 |
| 28[c] | SOC | 39 | 28/39 (71·8) | | | |
| | ASAQ | 39 | 26/39 (66·7) | ASAQ / SOC | 0·963 (0·704, 1·31) | 0·8134 |
| | PA | 36 | 24/35 (68·6) | PA / SOC | 1·018 (0·726, 1·42) | 0·9188 |
| | FPV+NTZ | 37 | 27/37 (73·0) | FPV+NTZ / SOC | 1·150 (0·838, 1·57) | 0·3863 |
| | SOF-DCV | 35 | 24/35 (68·6) | SOF-DCV / SOC | 0·989 (0·724, 1·35) | 0·9459 |

[a] Number of patients with outcome/number of patients evaluable at each time point.

[b] Log link and Poisson distribution.

[c] Log link and binomial distribution.

mITT, modified intention-to-treat; SOC, standard-of-care; ASAQ, artesunate-amodiaquine; PA, pyronaridine-artesunate; FPV+NTZ, favipiravir plus nitazoxanide; SOF-DCV, sofosbuvir-daclatasvir.

The logistic regression model contained treatment arm, age at baseline (years), sex, baseline body mass index, baseline comorbidities, baseline viral load category and days of symptoms at time of enrolment as covariates.

**Figure S1 Incidence of SARS-CoV-2 clearance on days 3, 10, 14, 21, and 28 based on RT-PCR (mITT population). See also Table S6.**

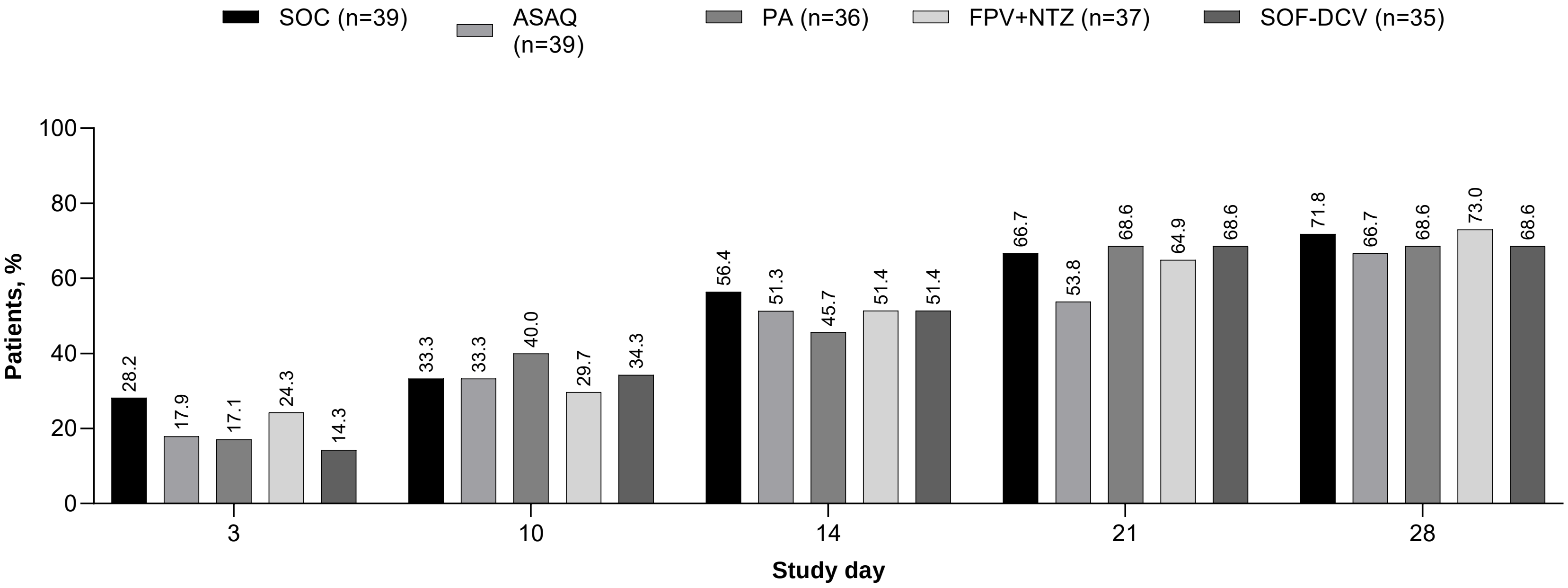


mITT, modified intention-to-treat; SOC, standard-of-care; ASAQ, artesunate-amodiaquine; PA, pyronaridine-artesunate; FPV+NTZ, favipiravir plus nitazoxanide; SOF-DCV, sofosbuvir-daclatasvir.

**Table S7 Repeated measure analysis of $log_{10}$ viral load of SARS-CoV-2 change from baseline (mITT population).**

| Treatment arm | Day | N[a] | LSmean change (SE) | 95%CI | Difference test–reference (SE) | 95%CI | 2-sided *P* value |
|---|---|---|---|---|---|---|---|
| SOC (N=39) | 3 | 37 | −1·33 (0·32) | −1·96, −0·69 | Reference | | |
| | 7 | 38 | −2·53 (0·28) | −3·09, −1·97 | | | |
| | 10 | 36 | −2·99 (0·26) | −3·50, −2·48 | | | |
| | 14 | 37 | −3·40 (0·27) | −3·94, −2·86 | | | |
| ASAQ (N=39) | 3 | 38 | −1·31 (0·31) | −1·92, −0·69 | 0·02 (0·44) | −0·85, 0·89 | 0·9642 |
| | 7 | 37 | −2·57 (0·28) | −3·12, −2·02 | −0·04 (0·39) | −0·81, 0·73 | 0·9248 |
| | 10 | 38 | −2·71 (0·25) | −3·20, −2·22 | 0·28 (0·35) | −0·42, 0·97 | 0·4293 |
| | 14 | 38 | −3·25 (0·26) | −3·76, −2·73 | 0·15 (0·37) | −0·58, 0·89 | 0·6773 |
| PA (N=36) | 3 | 32 | −1·48 (0·34) | −2·15, −0·81 | −0·16 (0·46) | −1·07, 0·76 | 0·7374 |
| | 7 | 31 | −2·42 (0·30) | −3·02, −1·82 | 0·11 (0·41) | −0·70, 0·93 | 0·7852 |
| | 10 | 31 | −3·01 (0·27) | −3·55, −2·47 | −0·02 (0·37) | −0·76, 0·72 | 0·9574 |
| | 14 | 31 | −3·48 (0·29) | −4·05, −2·91 | −0·08 (0·39) | −0·86, 0·70 | 0·8405 |
| FPV+NTZ (N=37) | 3 | 37 | −0·98 (0·31) | −1·60, −0·36 | 0·35 (0·45) | −0·54, 1·24 | 0·4393 |
| | 7 | 35 | −2·43 (0·28) | −2·99, −1·87 | 0·10 (0·40) | −0·69, 0·90 | 0·7947 |
| | 10 | 35 | −2·90 (0·25) | −3·40, −2·40 | 0·09 (0·36) | −0·63, 0·81 | 0·7981 |
| | 14 | 35 | −3·46 (0·27) | −3·99, −2·93 | −0·06 (0·38) | −0·82, 0·70 | 0·8709 |
| SOF-DCV (N=35) | 3 | 33 | −0·90 (0·33) | −1·56, −0·24 | 0·43 (0·46) | −0·48, 1·34 | 0·3546 |
| | 7 | 33 | −2·35 (0·30) | −2·94, −1·76 | 0·18 (0·41) | −0·63, 0·99 | 0·6576 |
| | 10 | 34 | −2·92 (0·27) | −3·44, −2·40 | 0·07 (0·37) | −0·66, 0·80 | 0·8526 |
| | 14 | 34 | −3·14 (0·28) | −3·69, −2·58 | 0·26 (0·39) | −0·51, 1·03 | 0·5006 |

[a] Number of patients with available data at the respective time point. mITT, modified intention-to-treat; SOC, standard-of-care; ASAQ, artesunate-amodiaquine; PA, pyronaridine-artesunate; FPV+NTZ, favipiravir plus nitazoxanide; SOF-DCV, sofosbuvir-daclatasvir. The mixed-effect linear model for repeated measures includes treatment, age at baseline (years), sex, body mass index at baseline, baseline comorbidities, baseline viral load category, days of symptoms at time of enrolment and treatment-by-visit interaction as fixed effects.

**Table S8 Repeated measure analysis of $log_{10}$ viral load of SARS-CoV-2 change from baseline in high-risk patients (mITT population).**

| Treatment arm | Day | N[a] | LSmean change (SE) | 95%CI | Difference test–reference (SE) | 95%CI | 2-sided *P* value |
|---|---|---|---|---|---|---|---|
| SOC (N=39) | 3 | 12 | −1·44 (0·68) | −2·79, −0·09 | Reference | | |
| | 7 | 12 | −3·12 (0·61) | −4·33, −1·92 | | | |
| | 10 | 12 | −3·43 (0·59) | −4·60, −2·26 | | | |
| | 14 | 11 | −3·76 (0·60) | −4·96, −2·56 | | | |
| ASAQ (N=39) | 3 | 17 | −1·40 (0·62) | −2·63, −0·18 | 0·04 (0·74) | −1·43, 1·51 | 0·9583 |
| | 7 | 16 | −3·02 (0·57) | −4·15, −1·89 | 0·10 (0·63) | −1·15, 1·35 | 0·8729 |
| | 10 | 18 | −3·39 (0·54) | −4·47, −2·31 | 0·03 (0·58) | −1·13, 1·20 | 0·9559 |
| | 14 | 17 | −3·94 (0·55) | −5·04, −2·84 | −0·18 (0·61) | −1·39, 1·03 | 0·7658 |
| PA (N=36) | 3 | 15 | −1·81 (0·64) | −3·08, −0·54 | −0·37 (0·76) | −1·89, 1·15 | 0·6316 |
| | 7 | 14 | −3·02 (0·59) | −4·18, −1·85 | 0·11 (0·65) | −1·19, 1·41 | 0·8705 |
| | 10 | 13 | −3·62 (0·57) | −4·76, −2·47 | −0·19 (0·62) | −1·43, 1·05 | 0·7617 |
| | 14 | 14 | −4·20 (0·57) | −5·34, −3·06 | −0·44 (0·64) | −1·71, 0·83 | 0·4925 |
| FPV+NTZ (N=37) | 3 | 16 | −1·16 (0·64) | −2·43, 0·10 | 0·28 (0·77) | −1·27, 1·82 | 0·7206 |
| | 7 | 16 | −3·44 (0·58) | −4·60, −2·29 | −0·32 (0·66) | −1·64, 1·00 | 0·6327 |
| | 10 | 16 | −3·07 (0·56) | −4·19, −1·95 | 0·36 (0·63) | −0·90, 1·62 | 0·5726 |
| | 14 | 14 | −4·08 (0·58) | −5·23, −2·92 | −0·31 (0·66) | −1·63, 1·00 | 0·6339 |
| SOF-DCV (N=35) | 3 | 16 | −1·30 (0·63) | −2·55, −0·04 | 0·14 (0·75) | −1·36, 1·65 | 0·8488 |
| | 7 | 15 | −3·19 (0·58) | −4·35, −2·03 | −0·06 (0·65) | −1·35, 1·23 | 0·9212 |
| | 10 | 16 | −3·64 (0·56) | −4·76, −2·52 | −0·21 (0·61) | −1·43, 1·00 | 0·7280 |
| | 14 | 16 | −4·30 (0·56) | −5·42, −3·17 | −0·54 (0·63) | −1·79, 0·71 | 0·3948 |

[a] Number of patients with available data at the respective time point.

mITT, modified intention-to-treat; SOC, standard-of-care; ASAQ, artesunate-amodiaquine; PA, pyronaridine-artesunate; FPV+NTZ, favipiravir plus nitazoxanide; SOF-DCV, sofosbuvir-daclatasvir. The mixed-effect linear model for repeated measures includes treatment, age at baseline (years), sex, body mass index at baseline, baseline comorbidities, baseline viral load category, days of symptoms at time of enrolment and treatment-by-visit interaction as fixed effects.

High risk was defined as age >60 years or body mass index >30 $kg/m^2$ plus the presence of at least one comorbidity for progression to severe disease.

**Table S9 Proportional odds model for disease progression for day 7, 14, 21, and 28 (mITT population).**

| Day | Treatment arm | N | Comparison | Adjusted odds ratio (95%CI) | *P* value |
|---|---|---|---|---|---|
| 7 | SOC | 39 | | | |
| | ASAQ | 39 | ASAQ / SOC | 0·41 (0·13, 1·31) | 0·1317 |
| | PA | 36 | PA / SOC | 0·71 (0·22, 2·36) | 0·5803 |
| | FPV+NTZ | 37 | FPV+NTZ / SOC | 1·12 (0·35, 3·61) | 0·8480 |
| | SOF-DCV | 35 | SOF-DCV / SOC | 1·59 (0·48, 5·27) | 0·4447 |
| 14 | SOC | 39 | | | |
| | ASAQ | 39 | ASAQ / SOC | 0·46 (0·11, 1·93) | 0·2906 |
| | PA | 36 | PA / SOC | 0·33 (0·07, 1·56) | 0·1613 |
| | FPV+NTZ | 37 | FPV+NTZ / SOC | 1·04 (0·28, 3·90) | 0·9528 |
| | SOF-DCV | 35 | SOF-DCV / SOC | 1·87 (0·49, 7·13) | 0·3577 |
| 21 | SOC | 39 | | | |
| | ASAQ | 39 | ASAQ / SOC | 1·88 (0·26,>9·99) | 0·5292 |
| | PA | 36 | PA / SOC | 0·90 (0·10, 8·36) | 0·9266 |
| | FPV+NTZ | 37 | FPV+NTZ / SOC | 3·18 (0·47,>9·99) | 0·2354 |
| | SOF-DCV | 35 | SOF-DCV / SOC | 2·48 (0·33,>9·99) | 0·3742 |
| 28 | SOC | 39 | | | |
| | ASAQ | 39 | ASAQ / SOC | 1·31 (0·29, 5·93) | 0·7285 |
| | PA | 36 | PA / SOC | 0·29 (0·04, 2·06) | 0·2172 |
| | FPV+NTZ | 37 | FPV+NTZ / SOC | 1·13 (0·23, 5·46) | 0·8792 |
| | SOF-DCV | 35 | SOF-DCV / SOC | 1·43 (0·29, 6·94) | 0·6597 |

WHO, world health organization; mITT, modified intention-to-treat; SOC, standard-of-care; ASAQ, artesunate-amodiaquine; PA, pyronaridine-artesunate; FPV+NTZ, favipiravir plus nitazoxanide; SOF-DCV, sofosbuvir-daclatasvir.

Disease severity was measured by WHO Ordinal Scale score for Clinical Improvement (ordered from 0<1<2<...<8). The longitudinal proportional odds model includes time point, treatment and treatment-by-time point interaction as fixed-effect factors. Baseline age (in years), sex, baseline BMI, baseline WHO Ordinal Scale score, baseline comorbidities, baseline viral load category and days of symptoms at time of enrolment are included as covariates. Participant-specific intercepts were included as random effects.

An adjusted odds ratio > 1 suggests a higher chance of more severe disease at the corresponding time point in the experimental treatment arm versus SOC.

**Table S10 Cox regression analysis of time to first zero WHO Ordinal Scale score for Clinical Improvement (mITT population).**

| Treatment arm | N | n/M (%) | Comparison | Hazard ratio (95%CI) | *P* value |
|---|---|---|---|---|---|
| SOC | 39 | 31/37 (83·8) | | | |
| ASAQ | 39 | 32/39 (82·1) | ASAQ / SOC | 1·29 (0·77, 2·16) | 0·3270 |
| PA | 36 | 31/35 (88·6) | PA / SOC | 1·45 (0·85, 2·46) | 0·1712 |
| FPV+NTZ | 37 | 31/36 (86·1) | FPV+NTZ / SOC | 0·81 (0·48, 1·37) | 0·4220 |
| SOF-DCV | 35 | 28/35 (80·0) | SOF-DCV / SOC | 0·77 (0·44, 1·34) | 0·3515 |

WHO, world health organization; mITT, modified intention-to-treat; SOC, standard-of-care; ASAQ, artesunate-amodiaquine; PA, pyronaridine-artesunate; FPV+NTZ, favipiravir plus nitazoxanide; SOF-DCV, sofosbuvir-daclatasvir; n, number of participants with WHO Ordinal Scale for Clinical Improvement; M, number of evaluable participants.

The cox regression model includes treatment arm, age at baseline (years), sex, baseline BMI, baseline comorbidities, baseline viral load category and days of symptoms at time of enrolment as covariates. A hazard ratio > 1 favors the treatment arm in the numerator of the ratio (= higher probability of having zero WHO Ordinal Scale score).

**Table S11 Poisson regression analysis for the proportion of days with fever, $SpO_2$ values <93%, or respiratory symptoms after randomisation (mITT population).**

| Treatment arm | N | N days with symptoms | N days of observation | Raw rate | Rate estimate (95%CI) | Comparison | Rate ratio (95%CI) | *P* value |
|---|---|---|---|---|---|---|---|---|
| **Days with fever** | | | | | | | | |
| SOC | 39 | 3 | 503 | 0·596 | 0·248 (0·060, 1·028) | | | |
| ASAQ | 39 | 0 | 552 | 0·000 | <0·001 (<0·001, INFTY) | ASAQ / SOC | <0·001 (<0·001, INFTY) | 0·9999 |
| PA | 36 | 8 | 462 | 1·732 | 0·695 (0·235, 2·052) | PA / SOC | 2·805 (0·718, 10·951) | 0·1379 |
| FPV+NTZ | 37 | 3 | 501 | 0·599 | 0·220 (0·055, 0·889) | FPV+NTZ / SOC | 0·890 (0·166, 4·766) | 0·8918 |
| SOF-DCV | 35 | 4 | 479 | 0·835 | 0·417 (0·114, 1·523) | SOF-DCV / SOC | 1·685 (0·340, 8·342) | 0·5226 |
| **$SpO_2$ <93%** | | | | | | | | |
| SOC | 39 | 2 | 502 | 0·398 | 0·237 (0·040, 1·412) | | | |
| ASAQ | 39 | 0 | 535 | 0·000 | <0·001 (<0·001, INFTY) | ASAQ / SOC | <0·001 (<0·001, INFTY) | 0·9999 |
| PA | 36 | 3 | 448 | 0·670 | 0·284 (0·048, 1·677) | PA / SOC | 1·198 (0·175, 8·190) | 0·8539 |
| FPV+NTZ | 37 | 1 | 500 | 0·200 | 0·129 (0·015, 1·070) | FPV+NTZ / SOC | 0·543 (0·044, 6·767) | 0·6353 |
| SOF-DCV | 35 | 1 | 480 | 0·208 | 0·116 (0·012, 1·108 | SOF-DCV / SOC | 0·491 (0·042, 5·718) | 0·5702 |
| **Days with respiratory symptoms** | | | | | | | | |
| SOC | 39 | 189 | 530 | 35·660 | 32·959 (28·252, 38·450) | | | |
| ASAQ | 39 | 164 | 585 | 28·034 | 25·934 (22·134, 30·386) | ASAQ / SOC | 0·787 (0·637, 0·972) | 0·0262 |
| PA | 36 | 164 | 490 | 33·469 | 31·037 (26·451, 36·416) | PA / SOC | 0·942 (0·759, 1·168) | 0·5838 |
| FPV+NTZ | 37 | 156 | 527 | 29·602 | 28·085 (23·947, 32·937) | FPV+NTZ / SOC | 0·852 (0·685, 1·061) | 0·1519 |
| SOF-DCV | 35 | 179 | 507 | 35·306 | 35·005 (30·099, 40·711) | SOF-DCV / SOC | 1·062 (0·858, 1·315) | 0·5807 |

mITT, modified intention-to-treat; SOC, standard-of-care; ASAQ, artesunate-amodiaquine; PA, pyronaridine-artesunate; FPV+NTZ, favipiravir plus nitazoxanide; SOF-DCV, sofosbuvir-daclatasvir.

Raw rate was calculated as the number of days with fever / number of days of observation x 100. Rate estimate was derived by means of a Poisson regression model, adjusted to a 100 day period. The Poisson regression model contained treatment arm, age at baseline (years), sex, baseline body mass index, baseline comorbidities, baseline viral load category and days of symptoms at time of enrolment as covariates. For each participant the number of days of observation (i.e. days where temperature is non-missing) was used as offset. INFTY=estimated as positive infinity.

**Figure S2 FLU-PRO Plus questionnaire scores and changes from baseline (mITT population).**

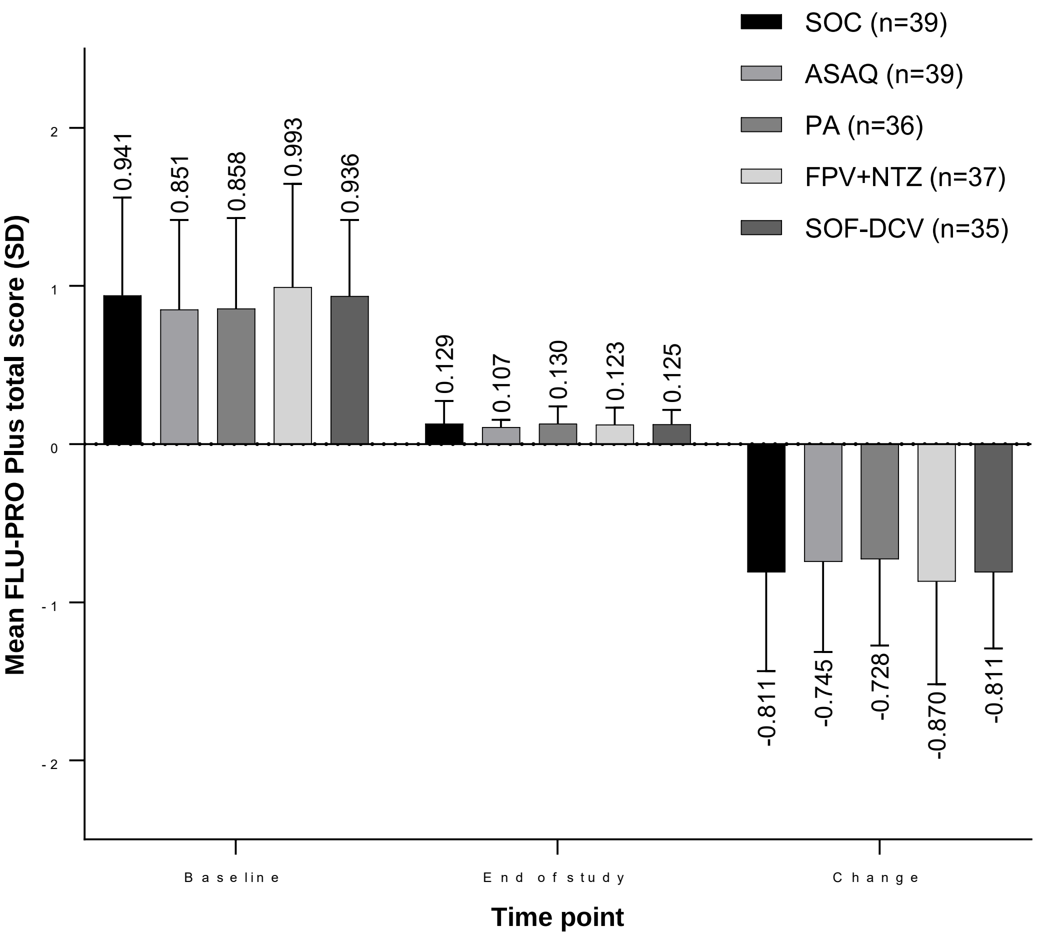


mITT, modified intention-to-treat; SOC, standard-of-care; ASAQ, artesunate-amodiaquine; PA, pyronaridine-artesunate; FPV+NTZ, favipiravir plus nitazoxanide; SOF-DCV, sofosbuvir-daclatasvir.

**Table S12 Investigational drug blood or plasma concentrations (pharmacokinetic population).**

| ASAQ (n=39) Day 3 | n (%) | Mean | Geometric mean | SD | Minimum | Q1 | Median | Q3 | Maximum |
|---|---|---|---|---|---|---|---|---|---|
| Artesunate, ng/mL | 37 (94·9) | 9·533 | 1·158 | 31·7108 | 0·78 | 0·78 | 0·78 | 0·78 | 151 |
| Dihydroartemisinin, ng/mL | 37 (94·9) | 71·369 | 3·586 | 269·9415 | 1·96 | 1·955 | 1·955 | 1·955 | 1420 |
| Amodiaquine, ng/mL | 36 (92·3) | 2·4211 | 1·6137 | 2·54236 | 0·078 | 1·21 | 1·525 | 2·225 | 11·3 |
| N-desethylamodiaquine, ng/mL | 35 (89·7) | 128·7 | 94·61 | 71·309 | 0·8 | 93·1 | 120 | 147 | 373 |
| **ASAQ (n=39) Day 7** | **n (%)** | **Mean** | **Geometric mean** | **SD** | **Minimum** | **Q1** | **Median** | **Q3** | **Maximum** |
| Artesunate, ng/mL | 38 (97·4) | 0·78 | 0·78 | 0 | 0·78 | 0·78 | 0·78 | 0·78 | 0·78 |
| Dihydroartemisinin, ng/mL | 38 (97·4) | 1·955 | 1·955 | 0 | 1·96 | 1·955 | 1·955 | 1·955 | 1·96 |
| Amodiaquine, ng/mL | 38 (97·4) | 0·2104 | 0·1826 | 0·10385 | 0·078 | 0·078 | 0·215 | 0·266 | 0·44 |
| N-desethylamodiaquine, ng/mL | 38 (97·4) | 67·24 | 53·21 | 27·734 | 0·8 | 52·00 | 69·10 | 87·20 | 142·0 |
| **PA (n=36) Day 3** | **n (%)** | **Mean** | **Geometric mean** | **SD** | **Minimum** | **Q1** | **Median** | **Q3** | **Maximum** |
| Pyronaridine, ng/mL | 35 (97·2) | 101·67 | 77·34 | 114·817 | 19·2 | 50·6 | 76·6 | 107 | 707 |
| Artesunate, ng/mL | 36 (100) | 2·747 | 0·884 | 11·8033 | 0·78 | 0·78 | 0·78 | 0·78 | 71·6 |
| Dihydroartemisinin, ng/mL | 36 (100) | 13·996 | 2·508 | 69·7875 | 1·96 | 1·955 | 1·955 | 1·955 | 421 |
| **PA (n=36) Day 7** | **n (%)** | **Mean** | **Geometric mean** | **SD** | **Minimum** | **Q1** | **Median** | **Q3** | **Maximum** |
| Pyronaridine, ng/mL | 35 (97·2) | 52·95 | 46·99 | 25·498 | 10·9 | 38 | 49·7 | 65·7 | 134 |
| Artesunate, ng/mL | 35 (97·2) | 0·78 | 0·78 | 0 | 0·78 | 0·78 | 0·78 | 0·78 | 0·78 |
| Dihydroartemisinin, ng/mL | 35 (97·2) | 1·955 | 1·955 | 0 | 1·96 | 1·955 | 1·955 | 1·955 | 1·96 |
| **FPV+NTZ (n=38) Day 7** | **n (%)** | **Mean** | **Geometric mean** | **SD** | **Minimum** | **Q1** | **Median** | **Q3** | **Maximum** |
| Favipiravir, µg/mL | 38 (100) | 9639·2 | 1593·2 | 14658·34 | 196 | 195·5 | 439·8 | 15000 | 61800 |
| Nitazoxanide, ng/mL | 38 (100) | 50 | 50 | 0 | 50 | 50 | 50 | 50 | 50 |
| Tizoxanide, ng/mL | 38 (100) | 2118·1 | 472·8 | 3708·66 | 50 | 50 | 610 | 2260 | 17400 |
| **SOF-DCV (n=33) Day 7** | **n (%)** | **Mean** | **Geometric mean** | **SD** | **Minimum** | **Q1** | **Median** | **Q3** | **Maximum** |
| Sofosbuvir, ng/mL | 33 (100) | 90·61 | 1·58 | 513·311 | 1·3 | 1·25 | 1·25 | 1·25 | 2950 |
| Daclatasvir, ng/mL | 33 (100) | 590·88 | 316·04 | 679·044 | 7·8 | 208 | 452 | 674 | 3510 |

ASAQ, artesunate-amodiaquine; PA, pyronaridine-artesunate; FPV+NTZ, favipiravir plus nitazoxanide; SOF-DCV, sofosbuvir-daclatasvir. Drug plasma or blood levels in these samples were determined at a central site (FARMOVS, Bloemfontein, South Africa) using validated protocols (Supplementary appendix Table S2).

**Figure S3 Drug plasma or blood concentrations at day 3 and day 7 in patients with or without SARS-CoV-2 clearance based on RT-PCR at day 7 (pharmacokinetic population).**

Concentrations below the lower limit of quantification (LLOQ) were imputed with LLOQ/2.

mITT, modified intention-to-treat; SOC, standard-of-care; ASAQ, artesunate-amodiaquine; PA, pyronaridine-artesunate; FPV+NTZ, favipiravir plus nitazoxanide; SOF-DCV, sofosbuvir-daclatasvir.

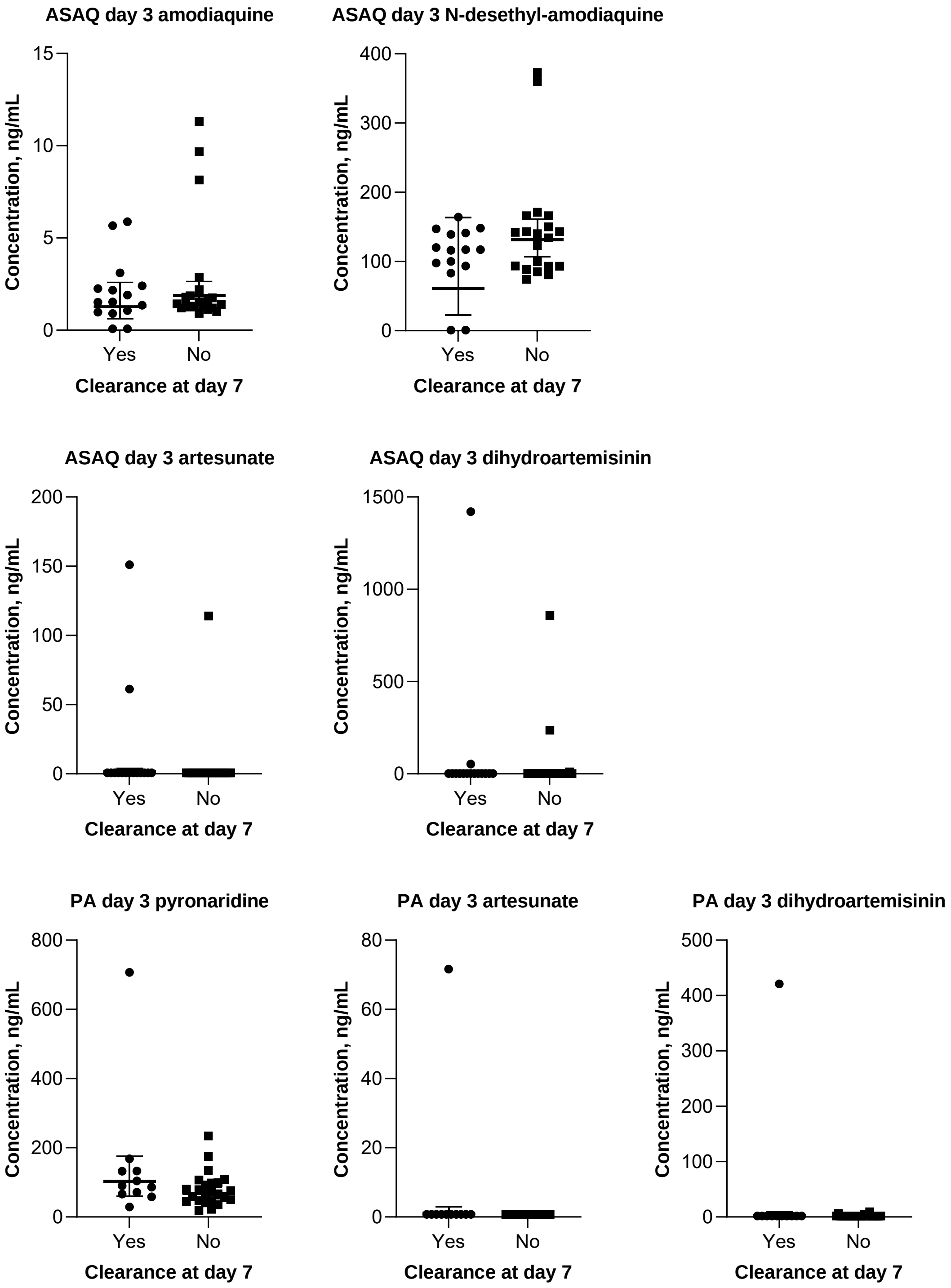

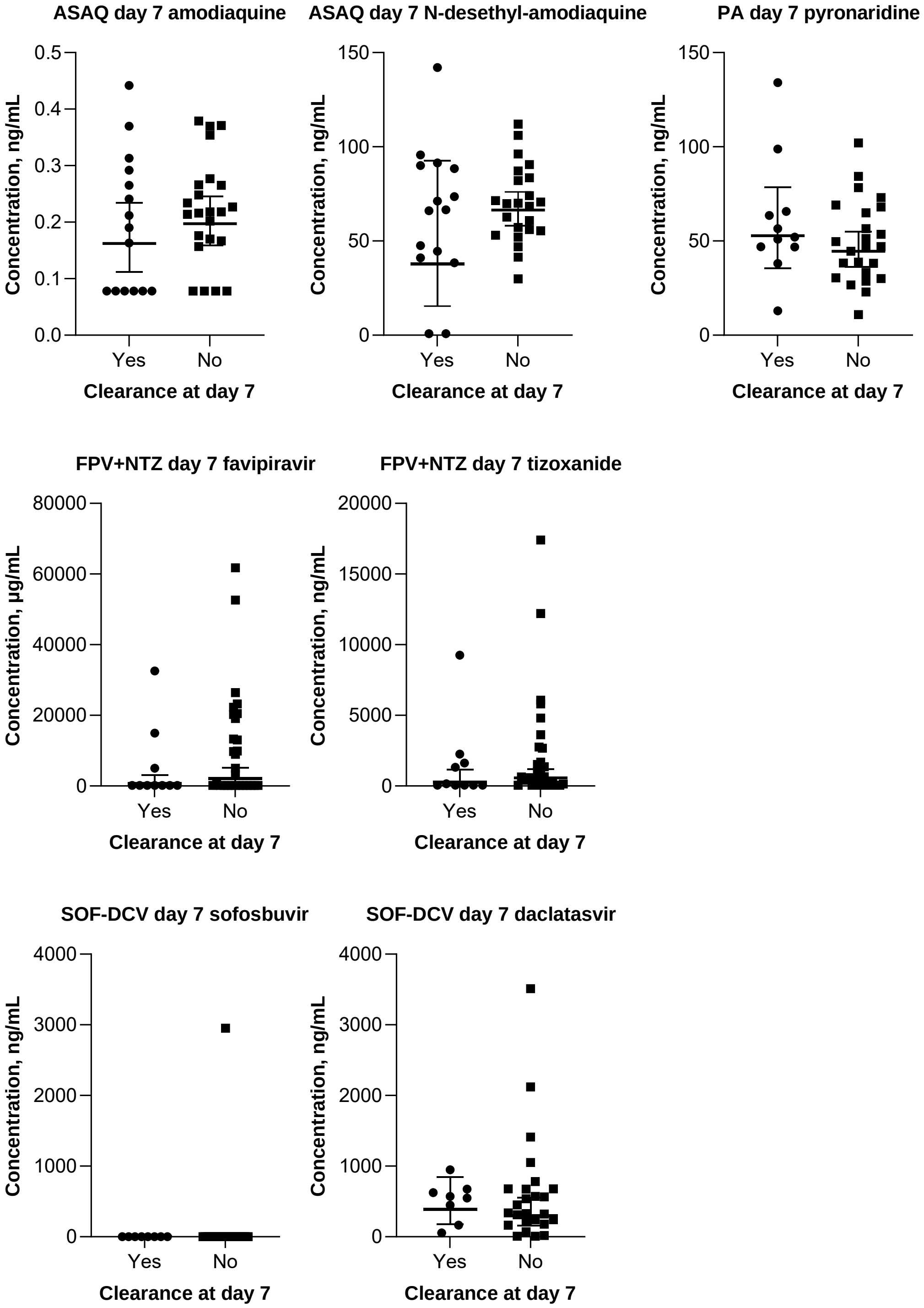
ASAQ day 7 amodiaquine
Concentration, ng/mL
0.5
0.4
0.3
0.2
0.1
0.0
Yes
No
Clearance at day 7
ASAQ day 7 N-desethyl-amodiaquine
Concentration, ng/mL
150
100
50
0
Yes
No
Clearance at day 7
PA day 7 pyronaridine
Concentration, ng/mL
150
100
50
0
Yes
No
Clearance at day 7
FPV+NTZ day 7 favipiravir
Concentration, µg/mL
80000
60000
40000
20000
0
Yes
No
Clearance at day 7
FPV+NTZ day 7 tizoxanide
Concentration, ng/mL
20000
15000
10000
5000
0
Yes
No
Clearance at day 7
SOF-DCV day 7 sofosbuvir
Concentration, ng/mL
4000
3000
2000
1000
0
Yes
No
Clearance at day 7
SOF-DCV day 7 daclatasvir
Concentration, ng/mL
4000
3000
2000
1000
0
Yes
No
Clearance at day 7

**Table S13 Treatment emergent adverse events of any cause and maximum severity (safety population).**

| Primary system organ class<br>Preferred term, n patients (%) | Severity grade | SOC (n=39) | | ASAQ (n=39) | | PA (n=38) | | FPV+NTZ (n=38) | | SOF-DCV (n=36) | |
|---|---|---|---|---|---|---|---|---|---|---|---|
| | | Patients n (%) | Events | Patients n (%) | Events | Patients n (%) | Events | Patients n (%) | Events | Patients n (%) | Events |
| Any adverse event | Any | 14 (35·9) | 32 | 18 (46·2) | 46 | 21 (55·3) | 48 | 31 (81·6) | 77 | 21 (58·3) | 35 |
| Patients may have had more than | Grade 1 | 13 (33·3) | 28 | 18 (46·2) | 39 | 20 (52·6) | 44 | 31 (81·6) | 67 | 18 (50·0) | 30 |
| one adverse event, worst adverse | Grade 2 | 1 (2·6) | 2 | 4 (10·3) | 7 | 3 (7·9) | 3 | 6 (15·8) | 9 | 4 (11·1) | 4 |
| event was counted in any category | Grade 3 | 2 (5·1) | 2 | 0 | 0 | 1 (2·6) | 1 | 0 | 0 | 1 (2·8) | 1 |
| | Grade 4 | 0 | 0 | 0 | 0 | 0 | 0 | 1 (2·6) | 1 | 0 | 0 |
| | Grade 5 | 0 | 0 | 0 | 0 | 0 | 0 | 0 | 0 | 0 | 0 |
| Blood and lymphatic system disorders | Any | 0 | 0 | 0 | 0 | 0 | 0 | 1 (2·6) | 1 | 0 | 0 |
| | Grade 4 | 0 | 0 | 0 | 0 | 0 | 0 | 1 (2·6) | 1 | 0 | 0 |
| Pancytopenia | Grade 4 | 0 | 0 | 0 | 0 | 0 | 0 | 1 (2·6) | 1 | 0 | 0 |
| Cardiac disorders | Any | 0 | 0 | 0 | 0 | 1 (2·6) | 1 | 0 | 0 | 0 | 0 |
| | Grade 1 | 0 | 0 | 0 | 0 | 1 (2·6) | 1 | 0 | 0 | 0 | 0 |
| Sinus tachycardia | Grade 1 | 0 | 0 | 0 | 0 | 1 (2·6) | 1 | 0 | 0 | 0 | 0 |
| Ear and labyrinth disorders | Any | 0 | 0 | 1 (2·6) | 1 | 0 | 0 | 0 | 0 | 0 | 0 |
| | Grade 1 | 0 | 0 | 1 (2·6) | 1 | 0 | 0 | 0 | 0 | 0 | 0 |
| Tinnitus | Grade 1 | 0 | 0 | 1 (2·6) | 1 | 0 | 0 | 0 | 0 | 0 | 0 |
| Eye disorders | Any | 1 (2·6) | 1 | 2 (5·1) | 2 | 2 (5·3) | 4 | 3 (7·9) | 3 | 4 (11·1) | 4 |
| | Grade 1 | 1 (2·6) | 1 | 2 (5·1) | 2 | 2 (5·3) | 4 | 3 (7·9) | 3 | 4 (11·1) | 4 |
| Photophobia | Grade 1 | 1 (2·6) | 1 | 0 | 0 | 1 (2·6) | 1 | 0 | 0 | 0 | 0 |
| Xerophthalmia | Grade 1 | 0 | 0 | 1 (2·6) | 1 | 1 (2·6) | 1 | 0 | 0 | 0 | 0 |
| Conjunctival discoloration | Grade 1 | 0 | 0 | 0 | 0 | 0 | 0 | 1 (2·6) | 1 | 0 | 0 |
| Conjunctivitis allergic | Grade 1 | 0 | 0 | 0 | 0 | 0 | 0 | 0 | 0 | 1 (2·8) | 1 |
| Eye pain | Grade 1 | 0 | 0 | 0 | 0 | 0 | 0 | 1 (2·6) | 1 | 0 | 0 |
| Eye pruritus | Grade 1 | 0 | 0 | 0 | 0 | 1 (2·6) | 1 | 0 | 0 | 0 | 0 |
| Eye swelling | Grade 1 | 0 | 0 | 0 | 0 | 0 | 0 | 0 | 0 | 1 (2·8) | 1 |
| Iris discoloration | Grade 1 | 0 | 0 | 0 | 0 | 0 | 0 | 1 (2·6) | 1 | 0 | 0 |
| Ocular hyperaemia | Grade 1 | 0 | 0 | 0 | 0 | 0 | 0 | 0 | 0 | 1 (2·8) | 1 |
| Orbital oedema | Grade 1 | 0 | 0 | 0 | 0 | 1 (2·6) | 1 | 0 | 0 | 0 | 0 |

| Primary system organ class<br>Preferred term, n patients (%) | Severity grade | SOC (n=39) | | ASAQ (n=39) | | PA (n=38) | | FPV+NTZ (n=38) | | SOF-DCV (n=36) | |
|---|---|---|---|---|---|---|---|---|---|---|---|
| | | Patients n (%) | Events | Patients n (%) | Events | Patients n (%) | Events | Patients n (%) | Events | Patients n (%) | Events |
| Periorbital swelling | Grade 1 | 0 | 0 | 1 (2·6) | 1 | 0 | 0 | 0 | 0 | 0 | 0 |
| Vision blurred | Grade 1 | 0 | 0 | 0 | 0 | 0 | 0 | 0 | 0 | 1 (2·8) | 1 |
| Gastrointestinal disorders | Any | 11 (28·2) | 17 | 12 (30·8) | 18 | 13 (34·2) | 19 | 22 (57·9) | 31 | 9 (25·0) | 12 |
| | Grade 1 | 10 (25·6) | 16 | 12 (30·8) | 17 | 13 (34·2) | 18 | 21 (55·3) | 29 | 8 (22·2) | 11 |
| | Grade 2 | 0 | 0 | 1 (2·6) | 1 | 1 (2·6) | 1 | 2 (5·3) | 2 | 1 (2·8) | 1 |
| | Grade 3 | 1 (2·6) | 1 | 0 | 0 | 0 | 0 | 0 | 0 | 0 | 0 |
| Nausea | Any | 2 (5·1) | 2 | 6 (15·4) | 6 | 4 (10·5) | 4 | 8 (21·1) | 8 | 4 (11·1) | 4 |
| | Grade 1 | 2 (5·1) | 2 | 6 (15·4) | 6 | 3 (7·9) | 3 | 8 (21·1) | 8 | 4 (11·1) | 4 |
| | Grade 2 | 0 | 0 | 0 | 0 | 1 (2·6) | 1 | 0 | 0 | 0 | 0 |
| Diarrhoea | Any | 5 (12·8) | 5 | 3 (7·7) | 3 | 3 (7·9) | 3 | 7 (18·4) | 7 | 4 (11·1) | 4 |
| | Grade 1 | 4 (10·3) | 4 | 3 (7·7) | 3 | 3 (7·9) | 3 | 6 (15·8) | 6 | 3 (8·3) | 3 |
| | Grade 2 | 0 | 0 | 0 | 0 | 0 | 0 | 1 (2·6) | 1 | 1 (2·8) | 1 |
| | Grade 3 | 1 (2·6) | 1 | 0 | 0 | 0 | 0 | 0 | 0 | 0 | 0 |
| Abdominal pain | Any | 2 (5·1) | 2 | 2 (5·1) | 2 | 4 (10·5) | 4 | 8 (21·1) | 8 | 2 (5·6) | 2 |
| | Grade 1 | 2 (5·1) | 2 | 2 (5·1) | 2 | 4 (10·5) | 4 | 7 (18·4) | 7 | 2 (5·6) | 2 |
| | Grade 2 | 0 | 0 | 0 | 0 | 0 | 0 | 1 (2·6) | 1 | 0 | 0 |
| Vomiting | Any | 4 (10·3) | 4 | 5 (12·8) | 5 | 5 (13·2) | 5 | 2 (5·3) | 2 | 1 (2·8) | 1 |
| | Grade 1 | 4 (10·3) | 4 | 4 (10·3) | 4 | 5 (13·2) | 5 | 2 (5·3) | 2 | 1 (2·8) | 1 |
| | Grade 2 | 0 | 0 | 1 (2·6) | 1 | 0 | 0 | 0 | 0 | 0 | 0 |
| Constipation | Grade 1 | 2 (5·1) | 2 | 0 | 0 | 0 | 0 | 1 (2·6) | 1 | 0 | 0 |
| Mouth ulceration | Grade 1 | 1 (2·6) | 1 | 0 | 0 | 2 (5·3) | 2 | 0 | 0 | 0 | 0 |
| Dyspepsia | Grade 1 | 1 (2·6) | 1 | 0 | 0 | 1 (2·6) | 1 | 0 | 0 | 0 | 0 |
| Haemorrhoids | Grade 1 | 0 | 0 | 0 | 0 | 0 | 0 | 2 (5·3) | 2 | 0 | 0 |
| Abdominal distension | Grade 1 | 0 | 0 | 0 | 0 | 0 | 0 | 0 | 0 | 1 (2·8) | 1 |
| Abdominal pain upper | Grade 1 | 0 | 0 | 1 (2·6) | 1 | 0 | 0 | 0 | 0 | 0 | 0 |
| Dry mouth | Grade 1 | 0 | 0 | 1 (2·6) | 1 | 0 | 0 | 0 | 0 | 0 | 0 |
| Flatulence | Grade 1 | 0 | 0 | 0 | 0 | 0 | 0 | 1 (2·6) | 1 | 0 | 0 |
| Gastrooesophageal reflux disease | Grade 1 | 0 | 0 | 0 | 0 | 0 | 0 | 1 (2·6) | 1 | 0 | 0 |
| Mouth swelling | Grade 1 | 0 | 0 | 0 | 0 | 0 | 0 | 1 (2·6) | 1 | 0 | 0 |

| Primary system organ class<br>Preferred term, n patients (%) | Severity grade | SOC (n=39) | | ASAQ (n=39) | | PA (n=38) | | FPV+NTZ (n=38) | | SOF-DCV (n=36) | |
|---|---|---|---|---|---|---|---|---|---|---|---|
| | | Patients n (%) | Events | Patients n (%) | Events | Patients n (%) | Events | Patients n (%) | Events | Patients n (%) | Events |
| General disorders and administration | Any | 2 (5·1) | 2 | 4 (10·3) | 4 | 1 (2·6) | 1 | 0 | 0 | 1 (2·8) | 1 |
| site conditions | Grade 1 | 2 (5·1) | 2 | 4 (10·3) | 4 | 1 (2·6) | 1 | 0 | 0 | 1 (2·8) | 1 |
| Chest discomfort | Grade 1 | 0 | 0 | 2 (5·1) | 2 | 0 | 0 | 0 | 0 | 0 | 0 |
| Chest pain | Grade 1 | 2 (5·1) | 2 | 0 | 0 | 0 | 0 | 0 | 0 | 0 | 0 |
| Axillary pain | Grade 1 | 0 | 0 | 0 | 0 | 0 | 0 | 0 | 0 | 1 (2·8) | 1 |
| Chills | Grade 1 | 0 | 0 | 1 (2·6) | 1 | 0 | 0 | 0 | 0 | 0 | 0 |
| Vessel puncture site haematoma | Grade 1 | 0 | 0 | 0 | 0 | 1 (2·6) | 1 | 0 | 0 | 0 | 0 |
| Vessel puncture site pain | Grade 1 | 0 | 0 | 1 (2·6) | 1 | 0 | 0 | 0 | 0 | 0 | 0 |
| Immune system disorders | Any | 0 | 0 | 0 | 0 | 1 (2·6) | 1 | 0 | 0 | 0 | 0 |
| | Grade 1 | 0 | 0 | 0 | 0 | 1 (2·6) | 1 | 0 | 0 | 0 | 0 |
| Seasonal allergy | Grade 1 | 0 | 0 | 0 | 0 | 1 (2·6) | 1 | 0 | 0 | 0 | 0 |
| Infections and infestations | Any | 1 (2·6) | 1 | 2 (5·1) | 2 | 3 (7·9) | 4 | 5 (13·2) | 5 | 3 (8·3) | 3 |
| | Grade 1 | 1 (2·6) | 1 | 1 (2·6) | 1 | 3 (7·9) | 3 | 4 (10·5) | 4 | 2 (5·6) | 2 |
| | Grade 2 | 0 | 0 | 1 (2·6) | 1 | 1 (2·6) | 1 | 1 (2·6) | 1 | 1 (2·8) | 1 |
| Upper respiratory tract infection | Grade 1 | 0 | 0 | 0 | 0 | 3 (7·9) | 3 | 0 | 0 | 1 (2·8) | 1 |
| Lower respiratory tract infection | Grade 2 | 0 | 0 | 1 (2·6) | 1 | 1 (2·6) | 1 | 0 | 0 | 0 | 0 |
| Vulvovaginal candidiasis | Grade 1 | 0 | 0 | 0 | 0 | 0 | 0 | 2 (5·3) | 2 | 0 | 0 |
| COVID-19 pneumonia | Grade 2 | 0 | 0 | 0 | 0 | 0 | 0 | 0 | 0 | 1 (2·8) | 1 |
| Conjunctivitis | Grade 1 | 0 | 0 | 0 | 0 | 0 | 0 | 1 (2·6) | 1 | 0 | 0 |
| Ear infection | Grade 1 | 1 (2·6) | 1 | 0 | 0 | 0 | 0 | 0 | 0 | 0 | 0 |
| Furuncle | Grade 1 | 0 | 0 | 0 | 0 | 0 | 0 | 1 (2·6) | 1 | 0 | 0 |
| Gingivitis | Grade 1 | 0 | 0 | 0 | 0 | 0 | 0 | 0 | 0 | 1 (2·8) | 1 |
| HIV infection | Grade 2 | 0 | 0 | 0 | 0 | 0 | 0 | 1 (2·6) | 1 | 0 | 0 |
| Periodontitis | Grade 1 | 0 | 0 | 1 (2·6) | 1 | 0 | 0 | 0 | 0 | 0 | 0 |
| Injury, poisoning and procedural | Any | 0 | 0 | 1 (2·6) | 1 | 0 | 0 | 0 | 0 | 1 (2·8) | 1 |
| complications | Grade 1 | 0 | 0 | 1 (2·6) | 1 | 0 | 0 | 0 | 0 | 1 (2·8) | 1 |
| Procedural dizziness | Grade 1 | 0 | 0 | 1 (2·6) | 1 | 0 | 0 | 0 | 0 | 0 | 0 |
| Soft tissue injury | Grade 1 | 0 | 0 | 0 | 0 | 0 | 0 | 0 | 0 | 1 (2·8) | 1 |
| Metabolism and nutrition disorders | Any | 0 | 0 | 1 (2·6) | 1 | 0 | 0 | 1 (2·6) | 1 | 0 | 0 |

| Primary system organ class<br>Preferred term, n patients (%) | Severity grade | SOC (n=39) | | ASAQ (n=39) | | PA (n=38) | | FPV+NTZ (n=38) | | SOF-DCV (n=36) | |
|---|---|---|---|---|---|---|---|---|---|---|---|
| | | Patients n (%) | Events | Patients n (%) | Events | Patients n (%) | Events | Patients n (%) | Events | Patients n (%) | Events |
| | Grade 1 | 0 | 0 | 1 (2·6) | 1 | 0 | 0 | 0 | 0 | 0 | 0 |
| | Grade 2 | 0 | 0 | 0 | 0 | 0 | 0 | 1 (2·6) | 1 | 0 | 0 |
| Decreased appetite | Grade 1 | 0 | 0 | 1 (2·6) | 1 | 0 | 0 | 0 | 0 | 0 | 0 |
| | Grade 2 | 0 | 0 | 0 | 0 | 0 | 0 | 1 (2·6) | 1 | 0 | 0 |
| Musculoskeletal and connective tissue disorders | Any | 0 | 0 | 3 (7·7) | 3 | 0 | 0 | 5 (13·2) | 5 | 0 | 0 |
| | Grade 1 | 0 | 0 | 2 (5·1) | 2 | 0 | 0 | 5 (13·2) | 5 | 0 | 0 |
| | Grade 2 | 0 | 0 | 1 (2·6) | 1 | 0 | 0 | 0 | 0 | 0 | 0 |
| Back pain | Any | 0 | 0 | 3 (7·7) | 3 | 0 | 0 | 2 (5·3) | 2 | 0 | 0 |
| | Grade 1 | 0 | 0 | 2 (5·1) | 2 | 0 | 0 | 2 (5·3) | 2 | 0 | 0 |
| | Grade 2 | 0 | 0 | 1 (2·6) | 1 | 0 | 0 | 0 | 0 | 0 | 0 |
| Muscle spasms | Grade 1 | 0 | 0 | 0 | 0 | 0 | 0 | 2 (5·3) | 2 | 0 | 0 |
| Pain in jaw | Grade 1 | 0 | 0 | 0 | 0 | 0 | 0 | 1 (2·6) | 1 | 0 | 0 |
| Nervous system disorders | Any | 4 (10·3) | 5 | 4 (10·3) | 6 | 12 (31·6) | 12 | 7 (18·4) | 9 | 5 (13·9) | 8 |
| | Grade 1 | 4 (10·3) | 5 | 4 (10·3) | 5 | 11 (28·9) | 11 | 6 (15·8) | 8 | 4 (11·1) | 6 |
| | Grade 2 | 0 | 0 | 1 (2·6) | 1 | 1 (2·6) | 1 | 1 (2·6) | 1 | 2 (5·6) | 2 |
| Dizziness | Any | 4 (10·3) | 4 | 2 (5·1) | 2 | 9 (23·7) | 9 | 4 (10·5) | 4 | 3 (8·3) | 4 |
| | Grade 1 | 4 (10·3) | 4 | 2 (5·1) | 2 | 8 (21·1) | 8 | 3 (7·9) | 3 | 3 (8·3) | 3 |
| | Grade 2 | 0 | 0 | 0 | 0 | 1 (2·6) | 1 | 1 (2·3) | 1 | 1 (2·8) | 1 |
| Headache | Any | 0 | 0 | 3 (7·7) | 3 | 2 (5·3) | 2 | 3 (7·9) | 3 | 3 (8·3) | 3 |
| | Grade 1 | 0 | 0 | 2 (5·1) | 2 | 2 (5·3) | 2 | 3 (7·9) | 3 | 3 (8·3) | 3 |
| | Grade 2 | 0 | 0 | 1 (2·6) | 1 | 0 | 0 | 0 | 0 | 0 | 0 |
| Paraesthesia | Grade 1 | 1 (2·6) | 1 | 0 | 0 | 1 (2·6) | 1 | 1 (2·6) | 1 | 0 | 0 |
| Somnolence | Grade 1 | 0 | 0 | 0 | 0 | 0 | 0 | 1 (2·6) | 1 | 0 | 0 |
| Syncope | Grade 2 | 0 | 0 | 0 | 0 | 0 | 0 | 0 | 0 | 1 (2·8) | 1 |
| Tremor | Grade 1 | 0 | 0 | 1 (2·6) | 1 | 0 | 0 | 0 | 0 | 0 | 0 |
| Psychiatric disorders | Any | 1 (2·6) | 2 | 4 (10·3) | 4 | 1 (2·6) | 2 | 1 (2·6) | 2 | 0 | 0 |
| | Grade 1 | 0 | 0 | 2 (5·1) | 2 | 1 (2·6) | 2 | 0 | 0 | 0 | 0 |
| | Grade 2 | 1 (2·6) | 1 | 2 (5·1) | 2 | 0 | 0 | 1 (2·6) | 2 | 0 | 2 |
| | Grade 3 | 1 (2·6) | 1 | 0 | 0 | 0 | 0 | 0 | 0 | 0 | 0 |

| Primary system organ class<br>Preferred term, n patients (%) | Severity grade | SOC (n=39) | | ASAQ (n=39) | | PA (n=38) | | FPV+NTZ (n=38) | | SOF-DCV (n=36) | |
|---|---|---|---|---|---|---|---|---|---|---|---|
| | | Patients n (%) | Events | Patients n (%) | Events | Patients n (%) | Events | Patients n (%) | Events | Patients n (%) | Events |
| Insomnia | Any | 0 | 0 | 3 (7·7) | 3 | 1 (2·6) | 1 | 0 | 0 | 0 | 0 |
| | Grade 1 | 0 | 0 | 2 (5·1) | 2 | 1 (2·6) | 1 | 0 | 0 | 0 | 0 |
| | Grade 2 | 0 | 0 | 1 (2·6) | 1 | 0 | 0 | 0 | 0 | 0 | 0 |
| Anxiety | Grade 1 | 0 | 0 | 0 | 0 | 1 (2·6) | 1 | 0 | 0 | 0 | 0 |
| | Grade 2 | 0 | 0 | 0 | 0 | 0 | 0 | 1 (2·6) | 1 | 0 | 0 |
| Panic attack | Grade 2 | 0 | 0 | 1 (2·6) | 1 | 0 | 0 | 1 (2·6) | 1 | 0 | 0 |
| Depression | Grade 2 | 1 (2·6) | 1 | 0 | 0 | 0 | 0 | 0 | 0 | 0 | 0 |
| Suicidal ideation | Grade 3 | 1 (2·6) | 1 | 0 | 0 | 0 | 0 | 0 | 0 | 0 | 0 |
| Renal and urinary disorders | Any | 0 | 0 | 0 | 0 | 1 (2·6) | 1 | 11 (28·9) | 12 | 0 | 0 |
| | Grade 1 | 0 | 0 | 0 | 0 | 1 (2·6) | 1 | 11 (28·9) | 12 | 0 | 0 |
| Chromaturia | Grade 1 | 0 | 0 | 0 | 0 | 1 (2·6) | 1 | 11 (28·9) | 12 | 0 | 0 |
| Reproductive system and breast disorders | Any | 1 (2·6) | 1 | 0 | 0 | 0 | 0 | 3 (7·9) | 4 | 0 | 0 |
| | Grade 1 | 1 (2·6) | 1 | 0 | 0 | 0 | 0 | 1 (2·6) | 2 | 0 | 0 |
| | Grade 2 | 0 | 0 | 0 | 0 | 0 | 0 | 2 (5·3) | 2 | 0 | 0 |
| Breast discoloration | Grade 2 | 0 | 0 | 0 | 0 | 0 | 0 | 1 (2·6) | 1 | 0 | 0 |
| Breast pain | Grade 1 | 1 (2·6) | 1 | 0 | 0 | 0 | 0 | 0 | 0 | 0 | 0 |
| Prostatitis | Grade 1 | 0 | 0 | 0 | 0 | 0 | 0 | 1 (2·6) | 1 | 0 | 0 |
| Semen discoloration | Grade 1 | 0 | 0 | 0 | 0 | 0 | 0 | 1 (2·6) | 1 | 0 | 0 |
| Uterine haemorrhage | Grade 2 | 0 | 0 | 0 | 0 | 0 | 0 | 1 (2·6) | 1 | 0 | 0 |
| Respiratory, thoracic and mediastinal disorders | Any | 2 (5·1) | 2 | 2 (5·1) | 3 | 3 (7·9) | 3 | 3 (7·9) | 4 | 4 (11·1) | 4 |
| | Grade 1 | 2 (5·1) | 2 | 2 (5·1) | 2 | 2 (5·3) | 2 | 3 (7·9) | 4 | 3 (8·3) | 3 |
| | Grade 2 | 0 | 0 | 1 (2·6) | 1 | 0 | 0 | 0 | 0 | 0 | 0 |
| | Grade 3 | 0 | 0 | 0 | 0 | 1 (2·6) | 1 | 0 | 0 | 1 (2·8) | 1 |
| Oropharyngeal pain | Grade 1 | 1 (2·6) | 1 | 0 | 0 | 0 | 0 | 2 (5·3) | 2 | 0 | 0 |
| Respiratory distress | Any | 0 | 0 | 1 (2·6) | 1 | 1 (2·6) | 1 | 0 | 0 | 1 (2·8) | 1 |
| | Grade 2 | 0 | 0 | 1 (2·6) | 1 | 0 | 0 | 0 | 0 | 0 | 0 |
| | Grade 3 | 0 | 0 | 0 | 0 | 1 (2·6) | 1 | 0 | 0 | 1 (2·8) | 1 |
| Epistaxis | Grade 1 | 0 | 0 | 0 | 0 | 0 | 0 | 1 (2·6) | 1 | 1 (2·8) | 1 |
| Nasal congestion | Grade 1 | 0 | 0 | 1 (2·6) | 1 | 0 | 0 | 1 (2·6) | 1 | 0 | 0 |

| Primary system organ class<br>Preferred term, n patients (%) | Severity grade | SOC (n=39) | | ASAQ (n=39) | | PA (n=38) | | FPV+NTZ (n=38) | | SOF-DCV (n=36) | |
|---|---|---|---|---|---|---|---|---|---|---|---|
| | | Patients n (%) | Events | Patients n (%) | Events | Patients n (%) | Events | Patients n (%) | Events | Patients n (%) | Events |
| Rhinitis allergic | Grade 1 | 0 | 0 | 0 | 0 | 1 (2·6) | 1 | 0 | 0 | 1 (2·8) | 1 |
| Dyspnoea | Grade 1 | 0 | 0 | 0 | 0 | 0 | 0 | 0 | 0 | 1 (2·8) | 1 |
| Hiccups | Grade 1 | 0 | 0 | 0 | 0 | 1 (2·6) | 1 | 0 | 0 | 0 | 0 |
| Rhinorrhoea | Grade 1 | 1 (2·6) | 1 | 0 | 0 | 0 | 0 | 0 | 0 | 0 | 0 |
| Throat irritation | Grade 1 | 0 | 0 | 1 (2·6) | 1 | 0 | 0 | 0 | 0 | 0 | 0 |
| Skin and subcutaneous tissue disorders | Any | 0 | 0 | 1 (2·6) | 1 | 0 | 0 | 0 | 0 | 1 (2·8) | 1 |
| | Grade 1 | 0 | 0 | 1 (2·6) | 1 | 0 | 0 | 0 | 0 | 1 (2·8) | 1 |
| Ecchymosis | Grade 1 | 0 | 0 | 1 (2·6) | 1 | 0 | 0 | 0 | 0 | 0 | 0 |
| Rash vesicular | Grade 1 | 0 | 0 | 0 | 0 | 0 | 0 | 0 | 0 | 1 (2·8) | 1 |
| Vascular disorders | Any | 1 (2·6) | 1 | 0 | 0 | 0 | 0 | 0 | 0 | 1 (2·8) | 1 |
| | Grade 1 | 0 | 0 | 0 | 0 | 0 | 0 | 0 | 0 | 1 (2·8) | 1 |
| | Grade 2 | 1 (2·6) | 1 | 0 | 0 | 0 | 0 | 0 | 0 | 0 | 0 |
| Hot flush | Grade 1 | 0 | 0 | 0 | 0 | 0 | 0 | 0 | 0 | 1 (2·8) | 1 |
| Hypertension | Grade 2 | 1 (2·6) | 1 | 0 | 0 | 0 | 0 | 0 | 0 | 0 | 0 |

SOC, standard-of-care; ASAQ, artesunate-amodiaquine; PA, pyronaridine-artesunate; FPV+NTZ, favipiravir plus nitazoxanide; SOF-DCV, sofosbuvir-daclatasvir.

MedDRA version 23·0 was used for coding adverse events.

**Table S13 Treatment emergent adverse events considered to be study drug related (safety population).**

| Primary system organ class<br>Preferred term, n patients (%) | SOC<br>(n=39) | ASAQ<br>(n=39) | PA<br>(n=38) | FPV+NTZ<br>(n=38) | SOF-DCV<br>(n=36) |
|---|---|---|---|---|---|
| Any drug-related adverse event | 0 | 11 (28·2) | 12 (31·6) | 21 (55·3) | 10 (27·8) |
| Ear and labyrinth disorders | 0 | 1 (2·6) | 0 | 0 | 0 |
| Tinnitus | 0 | 1 (2·6) | 0 | 0 | 0 |
| Eye disorders | 0 | 0 | 0 | 1 (2·6) | 1 (2·8) |
| Conjunctival discoloration | 0 | 0 | 0 | 1 (2·6) | 0 |
| Vision blurred | 0 | 0 | 0 | 0 | 1 (2·8) |
| Gastrointestinal disorders | 0 | 9 (23·1) | 9 (23·7) | 16 (42·1) | 8 (22·2) |
| Nausea | 0 | 5 (12·8) | 3 (7·9) | 7 (18·4) | 4 (11·1) |
| Diarrhoea | 0 | 3 (7·7) | 2 (5·3) | 6 (15·8) | 2 (5·6) |
| Abdominal pain | 0 | 1 (2·6) | 2 (5·3) | 6 (15·8) | 2 (5·6) |
| Vomiting | 0 | 3 (7·7) | 4 (10·5) | 2 (5·3) | 1 (2·8) |
| Abdominal distension | 0 | 0 | 0 | 0 | 1 (2·8) |
| Abdominal pain upper | 0 | 1 (2·6) | 0 | 0 | 0 |
| Gastrooesophageal reflux disease | 0 | 0 | 0 | 1 (2·6) | 0 |
| Nervous system disorders | 0 | 2 (5·1) | 7 (18·4) | 4 (10·5) | 2 (5·6) |
| Dizziness | 0 | 2 (5·1) | 6 (15·8) | 3 (7·9) | 2 (5·6) |
| Headache | 0 | 1 (2·6) | 1 (2·6) | 1 (2·6) | 2 (5·6) |
| Psychiatric disorders | 0 | 2 (5·1) | 0 | 0 | 0 |
| Insomnia | 0 | 2 (5·1) | 0 | 0 | 0 |
| Renal and urinary disorders | 0 | 0 | 0 | 11 (28·9) | 0 |
| Chromaturia | 0 | 0 | 0 | 11 (28·9) | 0 |
| Reproductive and breast system disorders | 0 | 0 | 0 | 1 (2·6) | 0 |
| Semen discolouration | 0 | 0 | 0 | 1 (2·6) | 0 |
| Vascular disorders | 0 | 0 | 0 | 0 | 1 (2·8) |
| Hot flush | 0 | 0 | 0 | 0 | 1 (2·8) |

Data are number of patients with an adverse event / total number of patients (%). Patients may have had more than one adverse event.

SOC, standard-of-care; ASAQ, artesunate-amodiaquine; PA, pyronaridine-artesunate; FPV+NTZ, favipiravir plus nitazoxanide; SOF-DCV, sofosbuvir-daclatasvir.

MedDRA version 23.0 was used for coding adverse events.

**Figure S4 Change in vital signs from baseline (safety population).**

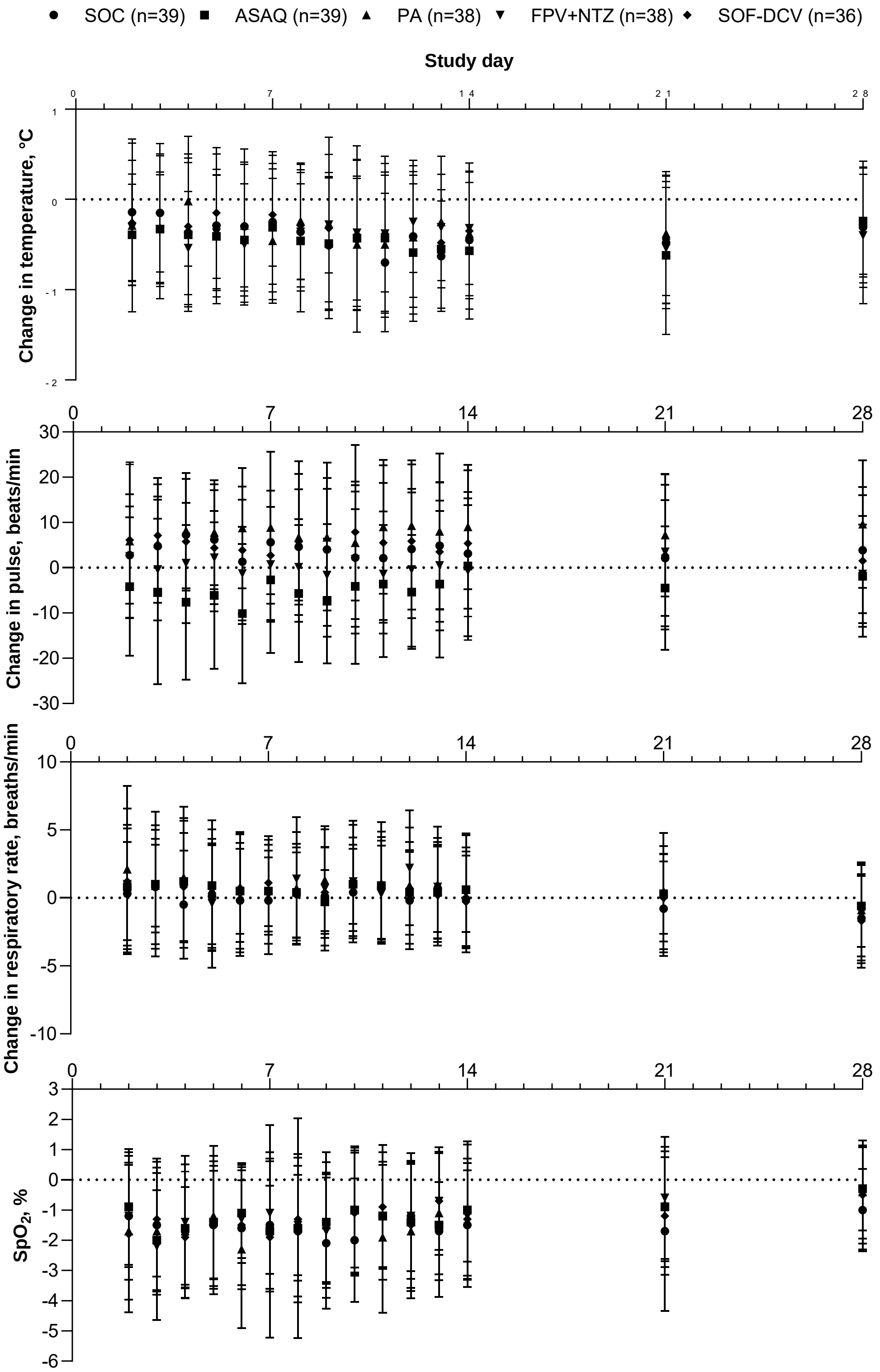


SOC, standard-of-care; ASAQ, artesunate-amodiaquine; PA, pyronaridine-artesunate; FPV+NTZ, favipiravir plus nitazoxanide; SOF-DCV, sofosbuvir-daclatasvir.

**Figure S5 Shift from baseline to day 28 in serology (mITT population).**

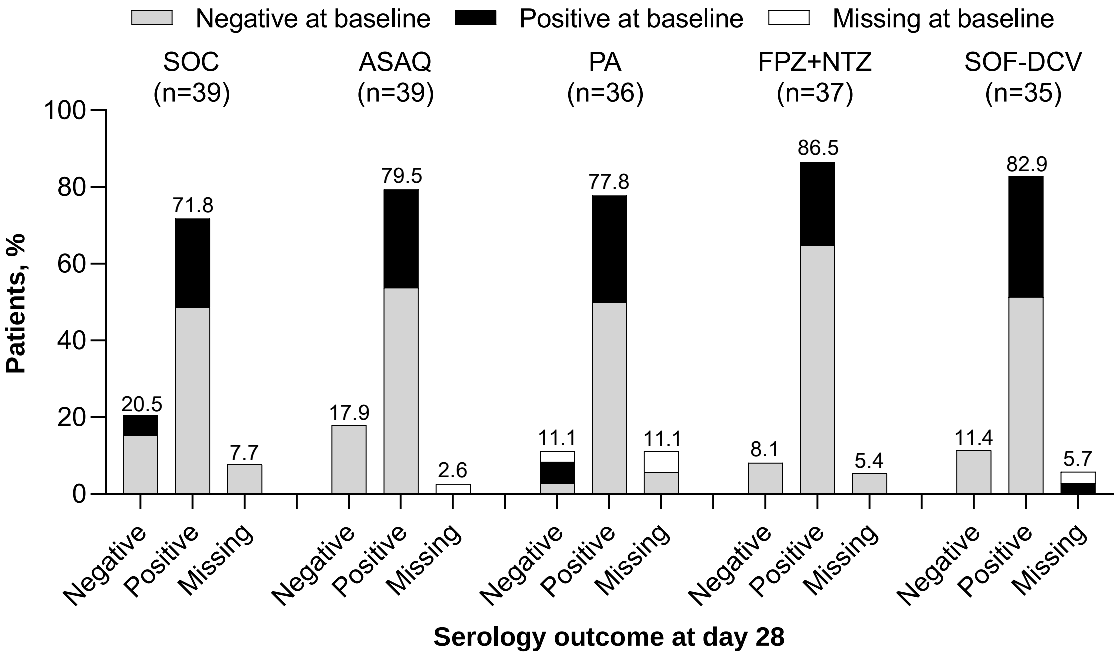


mITT, modified intention-to-treat; SOC, standard-of-care; ASAQ, artesunate-amodiaquine; PA, pyronaridine-artesunate; FPV+NTZ, favipiravir plus nitazoxanide; SOF-DCV, sofosbuvir-daclatasvir.
